# Basicity-Tuned Reactivity: *diaza*-[1,2]-Wittig versus *diaza*-[1,3]-Wittig Rearrangements of 3,4-Dihydro-2*H*-1,2,3-benzothiadiazine 1,1-Dioxides

**DOI:** 10.1021/acs.joc.0c02512

**Published:** 2020-12-31

**Authors:** Imre Gyűjtő, Márta Porcs-Makkay, Gergő Szabó, Zsolt Kelemen, Gyöngyvér Pusztai, Gábor Tóth, András Dancsó, Judit Halász, Gyula Simig, Balázs Volk, László Nyulászi

**Affiliations:** † Directorate of Drug Substance Development, Egis Pharmaceuticals Plc., P.O. Box 100, H-1475 Budapest, Hungary; ‡ Department of Inorganic and Analytical Chemistry, Budapest University of Technology and Economics, and MTA-BME Computation Driven Chemistry Research Group, Szt. Gellért tér 4, H-1111 Budapest, Hungary

## Abstract

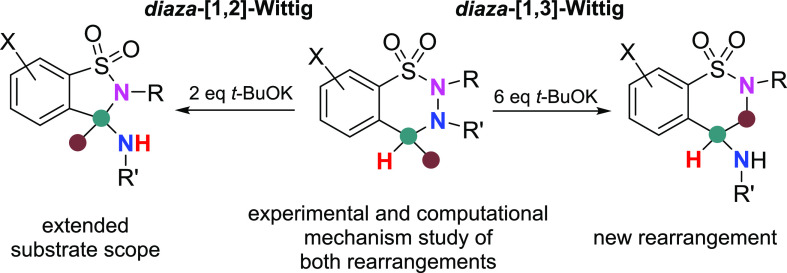

The base-induced (*t*-BuOK) rearrangement reactions of 3,4-dihydro-2*H*-1,2,3-benzothiadiazine 1,1-dioxides result in a ring opening along the N–N bond, followed by ring closure with the formation of new C–N bonds. The position of the newly formed C–N bond can selectively be tuned by the amount of the base, providing access to new, pharmacologically interesting ring systems with high yield. While with 2 equiv of *t*-BuOK 1,2-benzisothiazoles can be obtained in a *diaza*-[1,2]-Wittig reaction, with 6 equiv of the base 1,2-benzothiazine 1,1-dioxides can be prepared in most cases as the main product, in a *diaza*-[1,3]-Wittig reaction. DFT calculations and detailed NMR studies clarified the mechanism, with a mono- or dianionic key intermediate, depending on the amount of the reactant base. Also, the role of an enamide intermediate formed during the workup of the highly basic (6 equiv of base) reaction was clarified. The substrate scope of the reaction was also explored in detail.

## Introduction

**Scheme 1:**
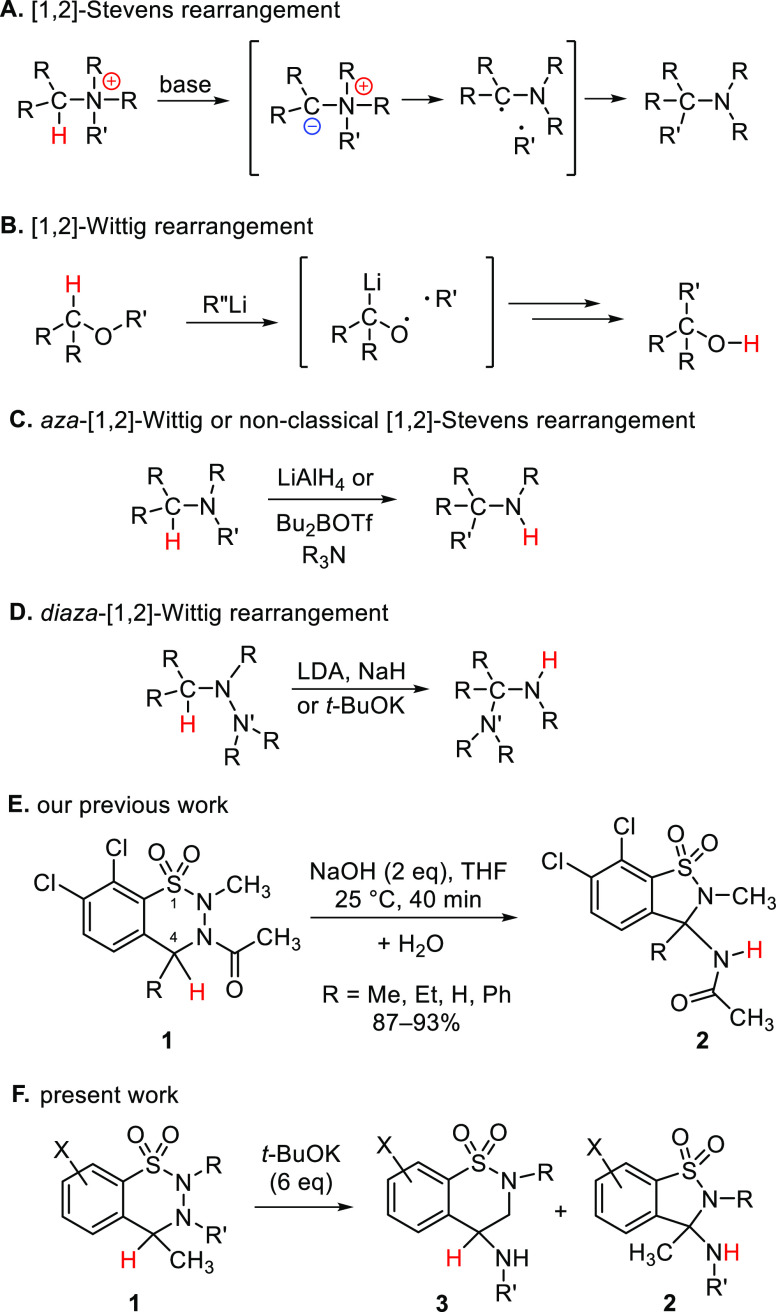
Stevens, Wittig, and Related Rearrangements; Our Previous Work, All Involving α-Deprotonation and [1,2]-Migration Steps; and the Present Work

Carbon, nitrogen, and oxygen atoms are the main constituents of organic frameworks, thus the selective formation of their bonds belongs to the most important organic transformations. [1,2]-Stevens reaction (Scheme 1A) is a well investigated example,^1^ where the ylide (formed after a base-induced deprotonation of one of the α-carbon atoms of a quaternary ammonium salt) takes part in a rearrangement via a [1,2]-shift of a nitrogen substituent. During this reaction step, a tight radical pair is usually formed after the cleavage of the C–N bond, but with certain substituents a concerted mechanism is proposed.^2,3^ [1,2]-Wittig rearrangements (Scheme 1B)^4^ with oxygen as the heteroatom and *aza*-[1,2]-Wittig rearrangements (Scheme 1C) are related transformations. This latter reaction can be considered as a nonclassical Stevens rearrangement, since here the reactant is a neutral species. While Wittig reactions including their variants^5^ are well investigated, likewise the Stevens reaction, the *aza*-[1,2]-Wittig transformation is rare^6−8^ and is often a side reaction, competing with *aza*-[2,3]-Wittig rearrangement.^9,10^ In the case of tetrasubstituted hydrazines, the N–N bond is breaking and the terminal amino unit is shifting, resulting in a *diaza*-[1,2]-Wittig rearrangement (Scheme 1D).^11−16^ Exposure of these derivatives to bases can also result in imines and amines as possible intermediates,^16,17^ indicating that with the breaking of the N–N bond during the reaction, the formation of new N–H bonds becomes also feasible. *Diaza*-1,4-Wittig rearrangement is also precedented;^18^ nonetheless, the *diaza*-[1,3]-Wittig reaction is a missing link.

Recently, we have reported a facile and (at the 4-position) substituent tolerant *diaza*-[1,2]-Wittig rearrangement of 3-acetyl-7,8-dichloro-2-methyl-3,4-dihydro-2*H*-1,2,3-benzothiadiazine 1,1-dioxides (**1**, Scheme 1E) to the corresponding 3-acetamido substituted 2,3-dihydro-1,2-benzisothiazole 1,1-dioxides (**2**), representatives of a compound family of biological relevance,^19−25^ in the presence of a suspension of 2 equiv of NaOH (or *t*-BuOK) in THF.^26^ While in our previous work we tentatively proposed an ionic pathway for this *diaza*-[1,2]-Wittig reaction yielding the benzisothiazole dioxides (**2**, Scheme 1E),^26^ the mechanism was not investigated in detail. During these investigations we now surprisingly found that, in the presence of a larger amount of base, the reaction gave also rise to the formation of an unexpected benzothiazine derivative (**3**, Scheme 1F). These new results are reported below.

## Results and Discussion

**Scheme 2:**
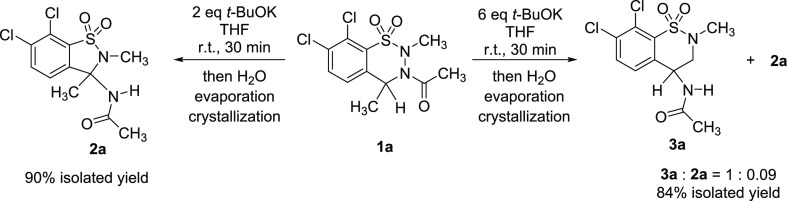
Discovery of the Basicity-Dependent Rearrangement Reactions

When seeking mechanistic information on the reaction starting from compound **1a**, we realized that depending on the basicity of the system (i.e., on the amount of *t*-BuOK applied), the reaction could result either in the previously described benzisothiazole **2a**(26) or in an unexpected benzothiazine derivative **3a** (Scheme 2), the latter being again a molecular framework in the focus of recent pharmacological interest.^27−35^ Hereby we explore the mechanism of this highly complex reaction and demonstrate that it is possible to selectively influence the outcome of the reaction toward any of the two different products. The corresponding experimental and computational studies are described below, with further details disclosed in the Supporting Information (SI).

### Computational Examination of the Deprotonation of Benzothiadiazine Dioxide **1a**, As the Initial Step of the Rearrangement

**Scheme 3:**
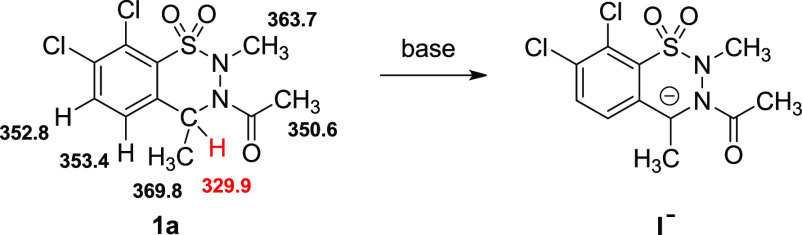
Evaluation of the Gas-Phase Acidity of **1a** at Different Positions and the Anion (**I**^**–**^) Obtained after Deprotonation Deprotonation Gibbs free energies are given in kcal/mol.

Having observed the *diaza*-[1,2]-Wittig reaction leading to 1,2-benzisothiazole **2a**(26) and considering the lack of mechanistic studies on this transformation, first we aimed to investigate the mechanism of the ring contraction of 4-methyl derivative **1a**. Since the presumable first elementary step of the rearrangement is the deprotonation of **1a**, we studied the effect of various bases (2 equiv) on the reaction shown in Scheme 2 (left). If the basicity was sufficient for deprotonation (*t*-BuOK, NaOH, NaOMe, and DBU), **2a** could be isolated in excellent yields (Table S1 in the SI), otherwise (DIPEA, NaOAc) **1a** was recovered. The possible positions for gas-phase proton abstraction were considered computationally (Scheme 3). As expected, the far most favored deprotonation site is the C(4) atom yielding anion **I**^**–**^, which is the likely starting point of the [1,2]-rearrangement. (For gas-phase acidity of some other systems at the same level of theory, see Table S2 in the SI). For further investigations, we decided to use *t*-BuOK as the deprotonating agent, since 2 equiv of this base gave 1,2-benzisothiazole dioxide **2a** selectively and with excellent isolated yield (Scheme 2).^26^

### NMR Study of the Effect of the Amount of *t*-BuOK on the Reaction Mechanism

**Figure 1:**
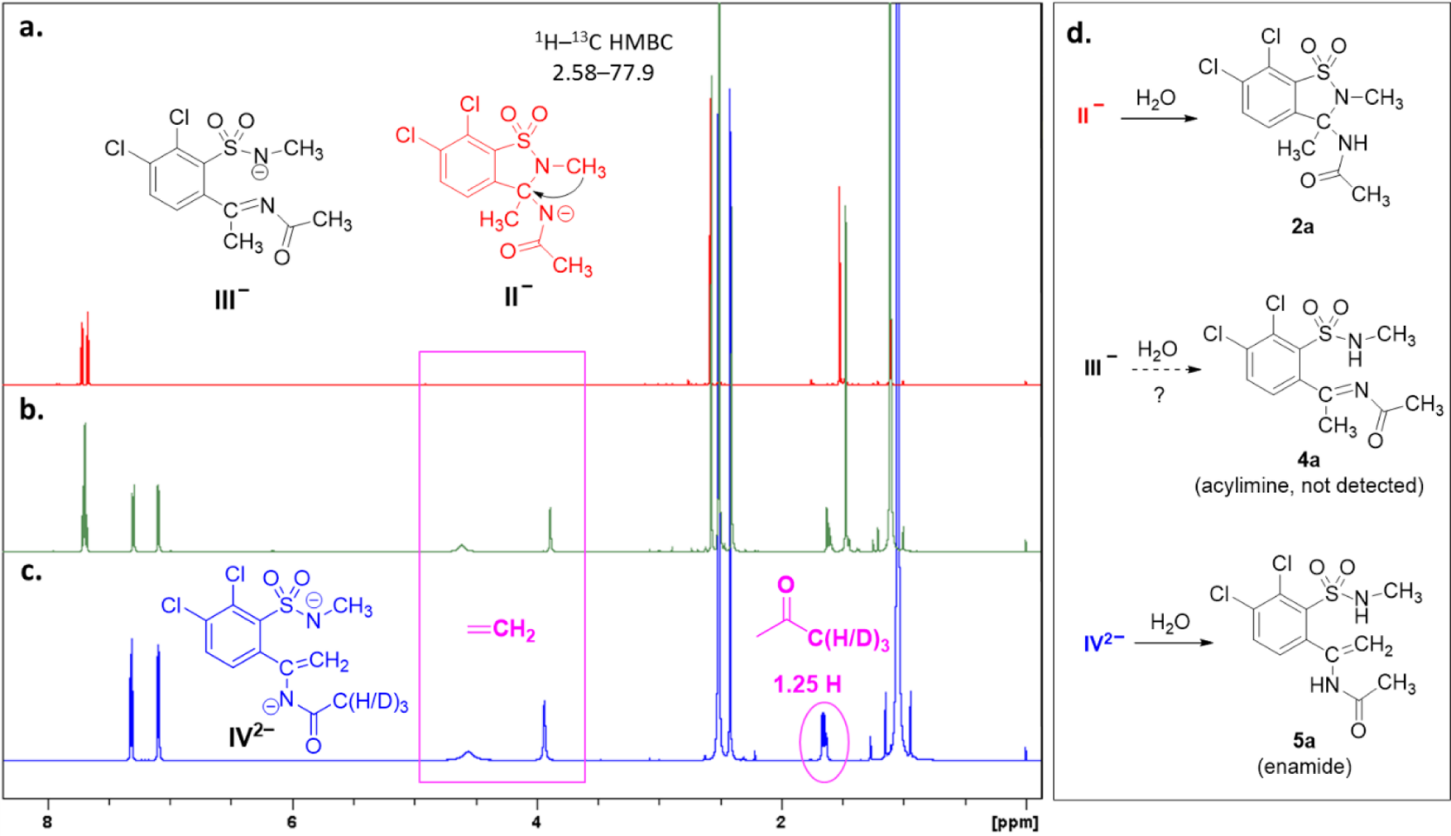
^1^H NMR analysis of reaction mixtures measured immediately after mixing **1a** (20 mg, 6.2 mmol) and 1 equiv (a, red), 2 equiv (b, green), or 6 equiv (c, blue) of *t*-BuOK (6.2, 12.4, or 37.1 mmol, respectively) in [D_6_]DMSO (800 μL) at 22 °C; and the corresponding neutral products obtained after quenching with water (d).

Aiming to obtain information on the reaction mechanism, the ^1^H NMR spectrum of the reaction mixture of **1a** and 2 equiv of *t*-BuOK was investigated in [D_6_]DMSO, immediately after mixing the reactants (Figure 1b). The detected signals corresponded to two compounds present in comparable amounts (ca. 1.1:1.0 molar ratio). When decreasing the amount of *t*-BuOK to 1 equiv, the signals marked by red (Figure 1a) become dominant. On the basis of the appearance of the characteristic 2.58/77.9 three-bond HMBC cross-peak (see the SI, page S26) caused by the vicinal ^3^*J*(NCH_3_/C-4) coupling, the compound can unequivocally be assigned to structural formula **II**^**–**^. Thus, the presence of **III**^**–**^, which would be indistinguishable by simple ^1^H NMR, can be excluded. The presence of intermediate **II**^**–**^ featuring the 1,2-benzisothiazole ring is also in accordance with the formation of **2a** upon protonation (i.e., quenching by water) and subsequent crystallization. Furthermore, it has to be noted that the formation of acylimine **4a** (the apparent protonation product of **III**^**–**^) was neither here, nor in our further experiments (see below) observed.

In the presence of 6 equiv *t*-BuOK, however, the peaks marked by blue (Figure 1/c) including the characteristic signals of a methylidene H_2_C= group (δ 4.56/3.94 ppm) become dominant. This dependence on the concentration of the base indicates that a dianionic compound, which was assigned as enamide dianion **IV**^**2–**^, was formed in the reaction mixture after removal of a second proton by the large excess of base. (For a detailed discussion of the ^1^H NMR and LC-MS spectra of the reaction mixture containing **IV**^**2–**^, see page S3 in the SI.) After quenching this reaction mixture with water and immediate extraction with DCM, not only product **3a** (47%) and the minor component **2a** (8%) could be identified by their ^1^H NMR spectra, but also enamide (**5a**, Figure 1/d, 45%) could be detected (Figure S3 in the SI). However, after our usual workup (evaporation of THF from the quenched mixture in vacuo) no **5a** was present in the precipitated product, which was obtained in 84% overall isolated yield, consisting mainly (92%) 1,2-benzothiazine 1,1-dioxide **3a**, with **2a** as the minor product (8%). The structure of compound **3a** was confirmed by detailed NMR studies and by single-crystal X-ray measurement (as a monohydrate). Structural details of compounds **1a**(26) and **3a·H**_**2**_**O** are given in the SI (Figures S11 and S12 and Tables S4 and S5).

### Effect of the Reaction Conditions on the Selectivity of the Rearrangements

It became obvious that the formation of the rearranged products **2a** and/or **3a** largely depended on the amount and strength of the base applied, providing a simple and convenient way to selectively tune the outcome of the reaction. As the next step, the conversion of compound **1a** has been investigated under various basic conditions (Table 1). The use of up to 2 equiv of *t*-BuOK in THF gave **2a** selectively in high yields (Table 1, entries 1 and 2). Upon increasing the amount of *t*-BuOK gradually to 8 equiv (entries 3–6), mixtures of **2a** and **3a** were isolated, and the selectivity could be shifted to **3a** (best with 6 equiv, entry 5). Change of the counterion to Na^+^ (entry 7) gave similar results. Use of 6 equiv of other bases in THF led to the formation of **2a** in high yields (entries 8–10). When applying 6 equiv of *t*-BuOK in other ether-type solvents or DMF, **3a** was obtained again as the major product (entries 11–14). Most importantly, from DMSO pure **3a** could be crystallized in 80% yield (entry 15), thereby making this reaction variant a practical synthesis of benzothiazine dioxide **3a**. On the contrary, using the protic *t*-BuOH, where the formation of the dianion intermediate is unlikely, **2a** (entry 16) was the sole product obtained.

**Table 1:**
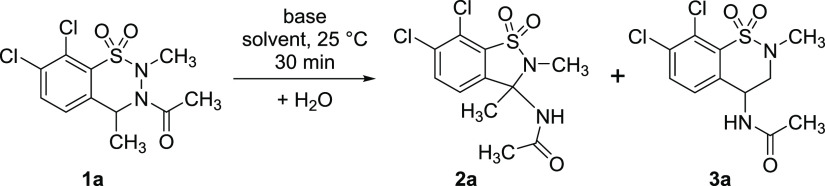
Effect of the Reaction Conditions on the Formation of Rearranged Products **2a** and **3a**

entry^a^	base	amount of base (eq)	solvent	**2a**:**3a** ratio^b^	yield (%)
1	*t*-BuOK	1	THF	1:0	83 (**2a**)
2	*t*-BuOK	2	THF	1:0	90 (**2a**)
3	*t*-BuOK	3	THF	1.00:0.88	87 (**2a**+**3a**)
4	*t*-BuOK	4	THF	0.31:1.00	80 (**2a**+**3a**)
5	*t*-BuOK	6	THF	0.09:1.00	84 (**2a**+**3a**)
6	*t*-BuOK	8	THF	0.13:1.00	88 (**2a**+**3a**)
7	*t*-BuONa	6	THF	0.09:1.00	85 (**2a**+**3a**)
8	KOH	6	THF	1:0	89 (**2a**)
9	NaOMe	6	THF	1:0	76 (**2a**)
10	NaNH_2_	6	THF	1:0	74 (**2a**)
11	*t*-BuOK	6	2-Me-THF	0.14:1.00	88 (**2a**+**3a**)
12	*t*-BuOK	6	1,4-dioxane	0.10:1.00	87 (**2a**+**3a**)
13	*t*-BuOK	6	DME	0.14:1.00	79 (**2a**+**3a**)
14	*t*-BuOK	6	DMF^c^	0.12:1.00	93 (**2a**+**3a**)
15	*t*-BuOK	6	DMSO^d^	0:1	84 (**3a**)
16	*t*-BuOK	6	*t*-BuOH	1:0	88 (**2a**)

a Reagents and reaction conditions: **1a** (0.6 mmol), base, solvent (3 mL), 25 °C, 30 min, then quenching with H_2_O, evaporation and crystallization.

b The product ratio of the isolated mixtures was determined by ^1^H NMR.

c H_2_O was added after evaporation.

d Without evaporation.

### DFT Calculations on the Ring Transformations

**Scheme 4:**
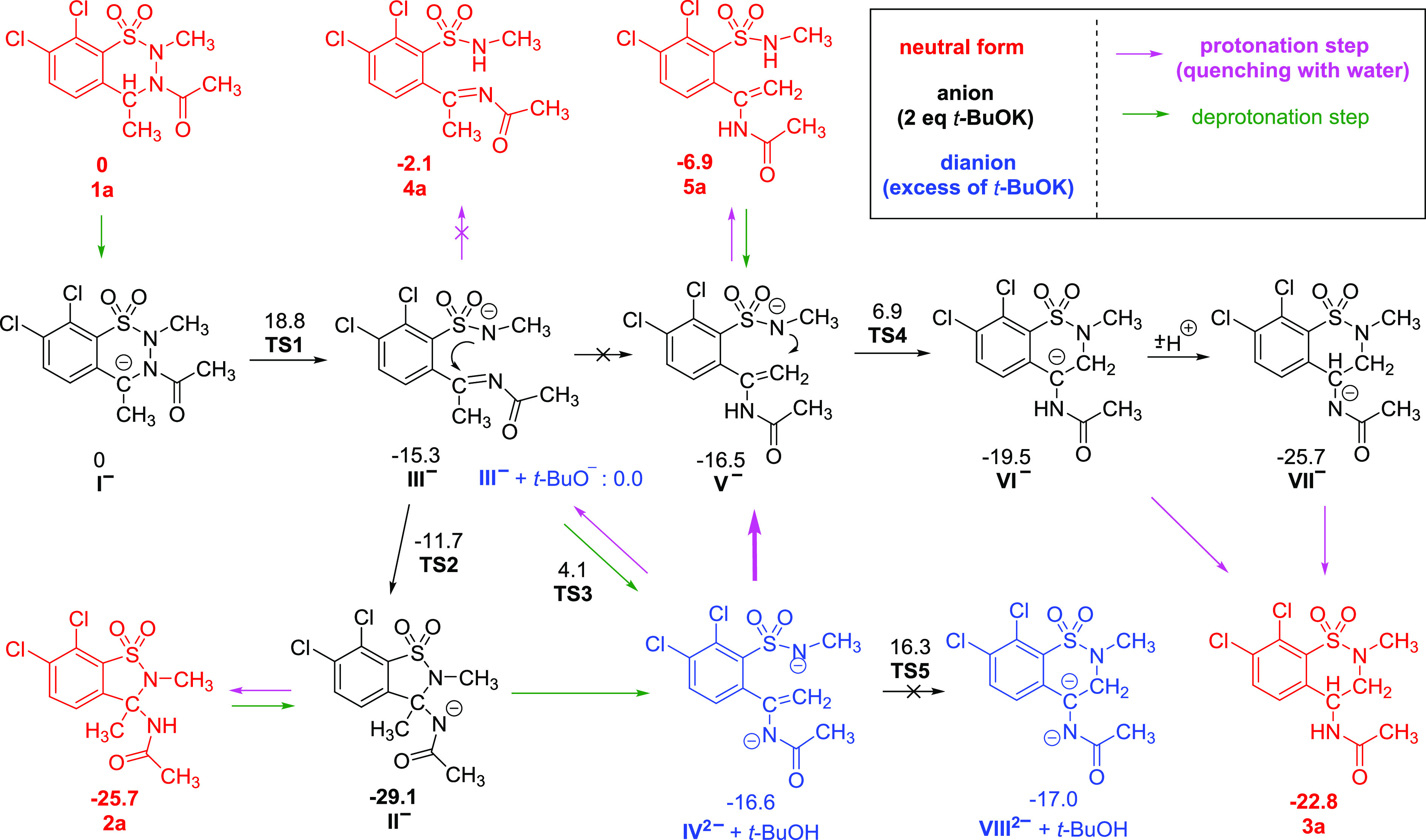
DFT Study of the Reaction Mechanism at M06-2X/6-31+G* (smd: THF) Level of Theory Reaction Gibbs free energy values are presented in kcal/mol.

In order to provide a reasonable mechanism for the rearrangement of **1a** to **2a** and **3a**, density functional theory (DFT) calculations have been carried out (Scheme 4, for the energy profile diagram see Figure S5 in the SI). Based on the investigation of the Kohn-Sahm molecular orbitals of **I**^**–**^, the concerted mechanism furnishing 1,2-benzisothiazole anion **II**^**–**^ in one step can be excluded. Though the HOMO is localized at C(4) (see Figure S4 in the SI), N(2) has no significant contribution to the LUMO. In accordance with this, we were not able to find any transition structure for a concerted pathway of the [1,2]-shift yielding **II**^**–**^ in one step. On the contrary, we could locate the transition state **TS1** corresponding to the N–N bond cleavage with a barrier of 18.8 kcal/mol leading to the formation of acylimine anion **III**^**–**^, which is significantly (by 15.3 kcal/mol) more stable than **I**^**–**^. The closed shell wave function turned out to be stable for both **III**^**–**^ and the corresponding transition structure. This is remarkable since the analogous step was claimed to have a biradical character in some cases of related Stevens rearrangements involving ylides,^2^ but it is in agreement with the observed lack of any electron spin resonance (ESR) signal or chemically induced dynamic nuclear polarization (CIDNP) signal intensity enhancement in the NMR during our experiments. In **III**^**–**^ the HOMO is at the N(2) atom, and the LUMO has a significant contribution at the C=N double bond (see Figure S4 in the SI), indicating a subsequent facile ring closure to form **II**^**–**^. Indeed, this step (**TS2**) has only a 3.6 kcal/mol activation barrier facilitating the rapid conversion (note that no NMR signal was observed for **III**^**–**^) to the thermodynamically more (by 13.8 kcal/mol) stable **II**^**–**^. It should be mentioned that the alternative pathway, i.e., the direct tautomerization of acylimine anion **III**^**–**^ to enamide anion **V**^**–**^, has a high reaction barrier furthermore **V**^**–**^ is by 12.6 kcal/mol less stable than **II**^**–**^. Thus, even if the formation of **V**^**–**^ from **III**^**–**^ might be possible by a base-catalyzed reversible deprotonation/protonation procedure via **IV**^**2–**^, the thermodynamic sink at the monoanionic level is **II**^**–**^, in accordance with the NMR results (see above).

Altogether, the selective formation of **2a** after reprotonation of **II**^**–**^ (during the workup with water) from the reaction of **1a** with 1–2 equiv of *t*-BuOK via the monoanionic pathway is fully justified on thermodynamic as well as on kinetic grounds.

When applying an excess of base, the formation of dianion **IV**^**2–**^ can easily be achieved by two alternative ways. First is by deprotonation of **III**^**–**^ with an excess of *t*-BuOK at the CH_3_ group via a barrier of 4.1 kcal/mol, calculated with respect to an incoming *t*-BuO^–^ and **III**^**–**^ as shown in Scheme 4. This barrier is comparable to that leading to the formation of **II**^**–**^ from **III**^**–**^ (3.6 kcal/mol). Alternatively, we can consider the deprotonation of the experimentally detected **II**^**–**^ at the C(3)-methyl group. Since any attempted optimization of the dianion derived by deprotonation of **II**^**–**^ resulted in a barrierless ring opening and finally in the formation of **IV**^**2–**^, and this pathway is also viable apart from the direct deprotonation of **III**^**–**^ discussed above.

Upon quenching the reaction mixture with excess of H_2_O, the protonation of **IV**^**2–**^ yields **V**^**–**^ thermodynamically somewhat more favorably than **III**^**–**^. Nevertheless, the energy difference between **III**^**–**^ and **V**^**–**^ is small (1.2 kcal/mol), in agreement with the presence of a small amount of **2a** (that can be derived from **III**^**–**^ as discussed above) in the isolated product. From **V**^**–**^, enamide **5a** can easily be obtained by further protonation, and this product was indeed observed after an immediate extraction of the reaction mixture (carried out with 6 equiv *t*-BuOK) with DCM, as discussed above. The neutral form of acylimine (**4a**, Scheme 4) is less stable than **5a**, and indeed, it remained experimentally unobserved. Since after quenching the solution is still highly basic, enamide **5a** can rearranged in a base-catalyzed reaction sequence, where the first step is a deprotonation. Thus, we should consider the reactivity of anion **V**^**–**^, which can cyclize in a thermodynamically downhill process via a reasonable 23.4 kcal/mol barrier to carbanion **VI**^**–**^,^37^ which might lead, after a proton exchange, to 1,2-benzothiazine anion **VII**^**–**^. Either **VI**^**–**^ or **VII**^**–**^ can be transformed to **3a** by protonation. Product **3a** could in principle be obtained directly from dianion **VIII**^**2–**^ as well, but the direct ring closure of **IV**^**2–**^ to dianion **VIII**^**2–**^ is unlikely, since this transformation has a high (32.9 kcal/mol) activation barrier (**TS5**). Indeed, no other major ^1^H NMR signals than those of **IV**^**2–**^ could be seen in the presence of 6 equiv of base (Figure 1).

### Experimental Studies on the Formation of Benzothiazine Dioxide Derivative **3a**

**Scheme 5:**
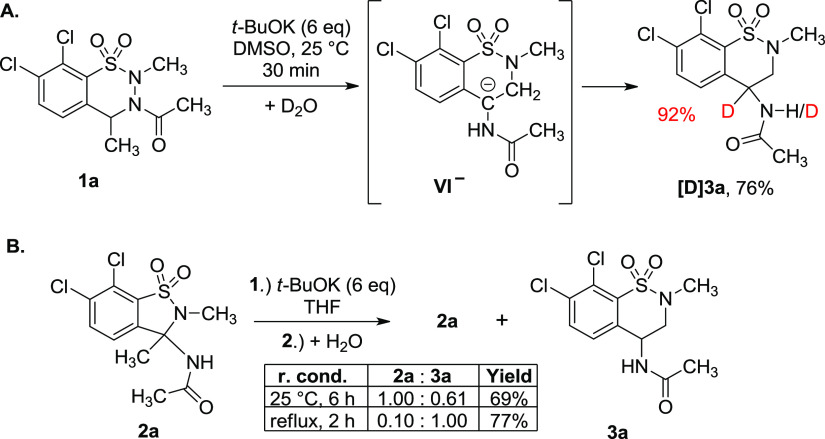
Experimental Studies on the Formation of the Benzothiazine Ring: (A) Partial Deuteration Observed after Quenching the Reaction Mixture with D_2_O and (B) Ring Expansion of Benzisothiazole **2a** to Benzothiazine **3a**

When the reaction of **1a** was carried out with 6 equiv of *t*-BuOK in DMSO, but quenched with D_2_O instead of water, **[D]3a** was obtained in 76% yield with a 92% deuteration ratio at position 4 (Scheme 5A and Figure S6 in the SI). This finding supports the suggested mechanism involving the formation of intermediate **VI**^**–**^. Clearly, in the CH_2_ unit of the thiazine ring no deuterium exchange was observed, showing that this methylene unit remains intact during the series of transformations, in accordance with our proposed mechanism starting from **IV**^**2–**^.

The question arose whether 1,2-benzisothiazole **2a** could be rearranged to 1,2-benzothiazine **3a** in a sufficiently basic medium. Treatment of 1,2-benzisothiazole **2a** with 6 equiv of *t*-BuOK at 25 °C for 6 h and subsequent quenching with water and crystallization afforded a mixture of 1,2-benzisothiazole **2a** and 1,2-benzothiazine **3a** (in 1.00:0.61 ratio), proving the occurrence of the **2a** → **3a** rearrangement (Scheme 5B). Furthermore, 2 h reflux and subsequent workup provided the “pseudoequilibrial” product ratio (0.10:1.00, Scheme 5B), in accordance with that obtained from the reaction of *t*-BuOK (6 equiv) and **1a** (Table 1, entry 5). It is noteworthy that this product ratio corresponds to the Boltzmann population of **III**^**–**^ and **V**^**–**^ as derived from their calculated 1.2 kcal/mol energy difference (Scheme 4).

While the reaction of **1a** with 6 equiv *t*-BuOK with the standard aqueous workup procedure led to **3a** as the main product (Table 1, entry 5), it is noteworthy that, with acidic workup, 1,2-benzisothiazole **2a** was obtained in excellent (92%) yield (see pages S13–14 in the SI). Thus, in the selective tuning of the outcome of the reaction, not only the amount of the base, but also the conditions of the workup are of high importance. Clearly, the thermodynamic sink is the formation of 1,2-benzisothiazole **2a** (see Scheme 4).

### Extension of the Substrate Scope of the Rearrangement of Benzothiadiazine 1,1-Dioxides (**1**) Leading to Benzisothiazole 1,1-Dioxides (**2**) Using 2 equiv of *t*-BuOK

**Scheme 6:**
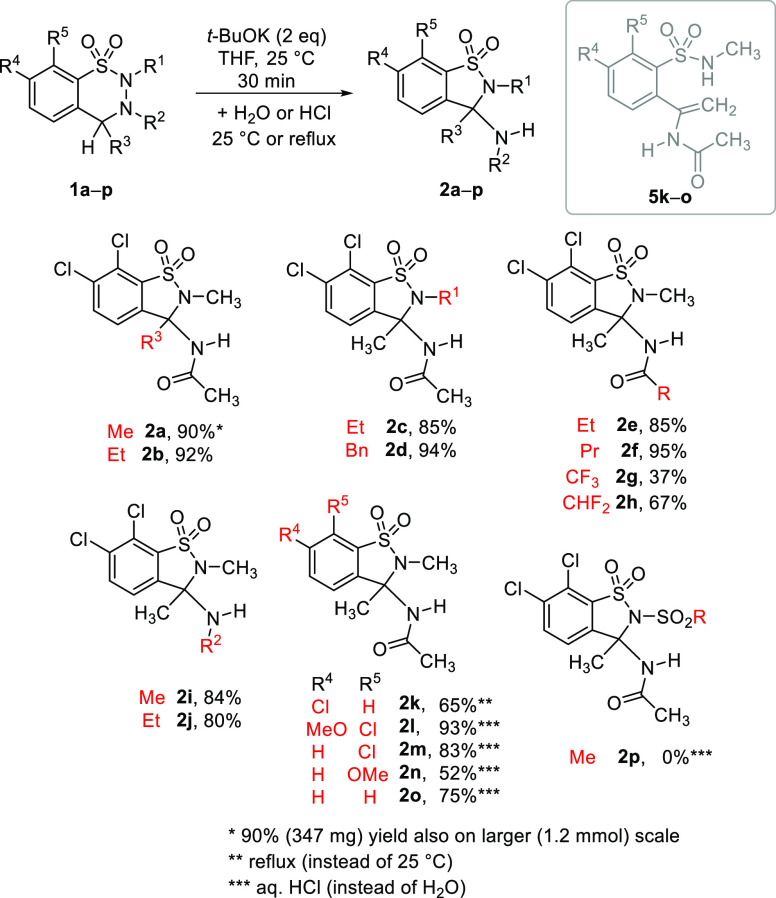
Study on the Substituent Effect on the Preparation of 1,2-Benzisothiazoles **2**, Carried out with 2 equiv of *t*-BuOK

With the useful information in hand on how to control the outcome of the rearrangements in the case of 3-acetyl-7,8-dichloro-2,4-dimethyl substituted derivative, we turned our attention to the experimental evaluation of the substituent effects. Therefore, variously substituted benzothiadiazine 1,1-dioxides **1** were synthesized as starting materials (for synthetic methods, see the SI) using the procedures described earlier.^26,38^ The ring contraction of derivatives **1** to 1,2-benzisothiazoles **2** was conducted in the presence of 2 equiv of *t*-BuOK in THF (Scheme 6), and in our standard procedure, the reaction mixture was quenched with water. Under these conditions, compounds **1a**–**j** (i.e., substrates bearing various 2-alkyl substituents, various acyl or alkyl groups at position 3, and chlorine atoms both at positions 7 and 8) were transformed smoothly and effectively to **2a**–**j** (in case of **1g**, the hydrolysis of the trifluoroacetylamino group lowered the yield). The synthesis of **2a** was successfully scaled up to 1.2 mmol of starting material **1a** with an unchanged yield (90%). Modifications in the aromatic substitution pattern (**1k**–**o**), however, have significantly reduced the formation of the precipitated 1,2-benzisothiazole (**2k**–**o**) in our usual workup procedure, and the corresponding enamides (**5k**–**o**) became the main products. For example, when the reaction mixture of **1o** was quenched with water and extracted with DCM, the isolated mixture contained the corresponding enamide (**5o**) predominantly, according to ^1^H NMR (Figures S7 and S8 in the SI), while benzisothiazole dioxide **2o** was only a minor product (12%). In case of 7-chloro-8-unsubstituted analogue **1k**, cyclization to **2k** did not take place under these conditions, it could only be forced at elevated temperature. For derivatives **1l**–**o**, cyclization with good to excellent yield could be fostered also at room temperature, by quenching the reaction mixture with aqueous (1 w/w%) hydrochloric acid instead of water. Benzothiazines **3** were not present in these experiments.

**Scheme 7:**
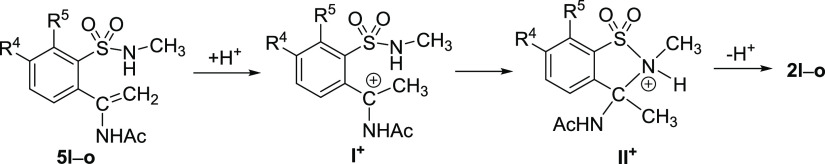
Proposed Mechanism for the Formation of 1,2-Benzisothiazoles **2l**–**o** with Acidic Work-Up

In the presence of the acid catalyst, the transformation of enamides **5l**–**o** to **2l**–**o** is easily understandable, considering that after protonation at the most basic site, i.e., at the methylidene group of **5**, carbocation **I**^**+**^ forms, which can be attacked by the nitrogen lone pair of the sulfonamide moiety to give **II**^**+**^, losing finally a proton from this nitrogen atom to result in neutral products **2l**–**o** (Scheme 7). Our DFT calculations on enamide **5o** fully supported the above mechanism, as shown in Figure S9 in the SI. The calculated proton affinity of the neutral **5o** is as high as 270.2 kcal/mol, and the resulting cation **Io**^**+**^ undergoes a ring closure via a tiny (2.3 kcal/mol) barrier (for more details see Figure S9 in the SI) to the thermodynamically more stable (by 8.9 kcal/mol) **IIo**^**+**^. In case of **5a**, similar proton affinity (261.8 kcal/mol) and reaction energy (−12.9 kcal/mol) were obtained, and scan calculations indicated a barrierless ring closure (Figure S10 in the SI). Altogether the high proton affinity and the small barrier indicate that the proton-catalyzed ring closure is a robust and generally applicable route for the formation of **2** for a wide range of substituents, in accordance with the above experimental observations.

**Scheme 8:**
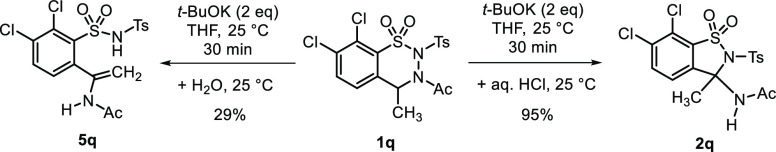
Dependence of the Product on the Workup Conditions in the Reaction of 3-Acetyl-2-tosyl Derivative **1q**

When starting from compound **1p** bearing a 2-mesyl group, a hydrolysis occurred under the usual reaction conditions, and no product (**2p**) was isolated (Scheme 6). In the case of 2-tosyl derivative **1q**, our standard procedure resulted in the precipitation of enamide **5q** (Scheme 8), isolated in 29% yield. If the same reaction mixture was treated with aqueous (1 w/w%) hydrochloric acid, again the thermodynamic sink, i.e., benzisothiazole **2q**, was obtained in excellent yield via the acid-catalyzed mechanism.

### Elaboration of the Targeted Synthesis of Benzothiazine 1,1-Dioxides (**3**) Using 6 equiv of *t*-BuOK

When studying the effect of the substituents on the pathway leading to benzothiazine **3a**, the effects stabilizing the key intermediates (dianion **IV**^**2–**^ and anion **V**^**–**^) are of importance, clearly benefiting from the delocalization along the C=C—N—C=O unit. Accordingly, when reacting 6 equiv of *t*-BuOK with compounds **1a**–**f** (all having an acyl moiety at position 3), a mixture of products **2** and **3** could be isolated in high overall yields (Table 2). A good selectivity was observed toward benzothiazine dioxides **3a**–**f** (entries 1–5), the only exception being 4-ethyl derivative **1b**, where benzothiazine **3b** was obtained as a mixture of diastereomers, in a ratio of 1.00:0.16 (entry 2), in accord with the expected effect of the 3-acyl group. In contrast, 2,3-dimethyl compound **1i** underwent even with 6 equiv *t*-BuOK a ring contraction to give **2i** in a virtually identical yield (85%, entry 6) as with 2 equiv of *t*-BuOK (see Scheme 6). The reactions of 3-acetyl-2-methyl derivatives bearing various substitution patterns (other than 7,8-dichloro) on the aromatic ring, likewise with 2 equiv of base, stopped at the enamide level. These reactions could be forced at reflux temperature to cyclization, however, in these cases again **2** was formed instead of **3** (entries 7–9).

**Table 2:**
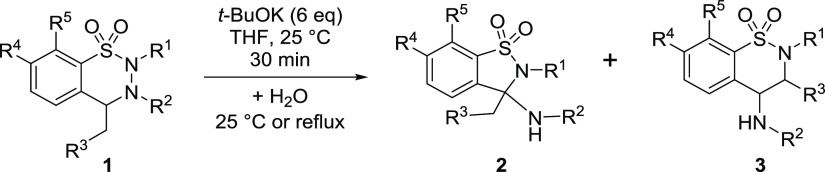
Study on the Substituent Effect in the Reaction of Compounds **1** Carried out with 6 equiv of *t*-BuOK

entry^a^	1–3	R^1^	R^2^	R^3^	R^4^	R^5^	2:3 ratio^b^	yield (%)
1	**a**	Me	C(O)Me	H	Cl	Cl	0.09:1.00	84^c^ (**2a**+**3a**)
2	**b**	Me	C(O)Me	Me	Cl	Cl	1.00:0.88^d^	54^c^ (**2b**+**3b**)
3	**c**	Et	C(O)Me	H	Cl	Cl	0.13:1.00	90^c^ (**2c**+**3c**)
4	**d**	Bn	C(O)Me	H	Cl	Cl	0.25:1.00	62^c^ (**2d**+**3d**)
5	**f**	Me	C(O)Pr	H	Cl	Cl	0.18:1.00	86^c^ (**2f**+**3f**)
6	**i**	Me	Me	H	Cl	Cl	1:0	85^c^ (**2i**)
7	**l**	Me	C(O)Me	H	OMe	Cl	1:0	74^e^ (**2l**)
8	**n**	Me	C(O)Me	H	H	OMe	1:0	35^e^ (**2n**)
9	**o**	Me	C(O)Me	H	H	H	1:0	59^e^ (**2o**)

a Reagents and reaction conditions: substrate (0.6 mmol), *t*-BuOK (3.6 mmol), THF (3 mL), 30 min, then quenching with H_2_O.

b The product ratio of the isolated mixtures was determined by ^1^H NMR.

c The reaction was carried out at 25 °C.

d dr = 1.00:0.16.

e The reaction was carried out at reflux temperature.

**Scheme 9:**
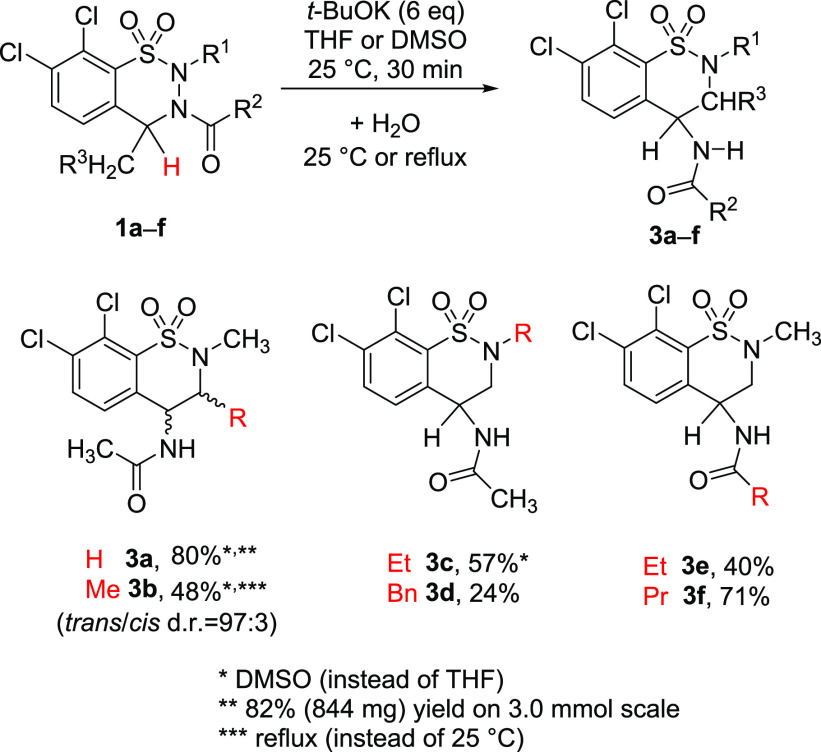
Targeted Synthesis of Variously Substituted 1,2-Benzothiazine 1,1-Dioxides **3**

The formation of compounds **3** is particularly noteworthy since [1,3]-rearrangements involving a N–N bond cleavage and inclusion of a C_2_ unit are very rare in the literature and occur with a different mechanism.^39−41^ The targeted synthesis of 1,2-benzothiazine 1,1-dioxides **3a**–**f** was finally conducted with 6 equiv of *t*-BuOK (Scheme 9), by crystallization either in DMSO (**3a**–**c**) or in THF (**3d**–**f**), yielding the products in a pure form even without chromatographic purification. Contrary to the other reactions taking place at room temperature, the quenched reaction mixture of 4-ethyl derivative **1b** had to be heated to reflux to cyclize to **3b** (trans–cis diastereomeric ratio = 97:3).

## Computations

DFT calculations were carried out with the Gaussian 09 software package.^42^ The level of theory was validated (Table S3), and the M06-2X^43^ functional was used in conjunction with the 6-31+G* basis set for conformational analysis on all reactants, transition states, and intermediates to identify the most plausible conformers (unless otherwise stated). For frequency calculations, ultrafine grid was used, and free energies are reported in kcal/mol at 1 atm and 25 °C. Free energies for gas-phase acidity were calculated with fine grid and G(H^+^(gas)) = −6.28 kcal/mol^44^ was used. Normal mode analysis has been performed, as well as intrinsic reaction coordinate (IRC) calculations to verify the transition state geometries. The possible pathways under study were modeled using the solvation model based on density (SMD)^45^ in THF. Further calculations on **I**^**–**^ were carried out using the B3LYP^46^ and the ωB97X-D^47^ functionals giving similar results as shown in the SI (Table S3). Stability of the wave functions was checked and the energies of anions and the corresponding radicals were compared to evade unnoticed electron loss. The Avogadro software^48^ was used for the visualization of PMOs and CYLview^49^ for geometries.

## Conclusion

In this paper we presented new, switchable base-catalyzed C–N bond forming reactions proceeding through a sequence of rearrangements. The ring contraction of variously substituted 3,4-dihydro-2*H*-1,2,3-benzothiadiazine 1,1-dioxides (**1**) to 1,2-benzisothiazole 1,1-dioxides (**2**) could be achieved in a *diaza*-[1,2]-Wittig reaction using 2 equiv of *t*-BuOK, followed by quenching with water and subsequent crystallization. With certain substitution patterns, the formation of enamide intermediates **5** was observed; however, when heating or using a 1% HCl solution for quenching, the selective formation of the thermodynamically most stable compounds **2** could be forced. The mechanism of the facile proton-catalyzed transformation of enamides **5** to benzisothiazoles **2** was explored by DFT calculations. When applying a larger excess (6 equiv) of *t*-BuOK in the reaction of compounds **1**, a hitherto unknown *diaza*-[1,3]-Wittig rearrangement providing 1,2-benzothiazines (**3**) was identified, and in a noteworthy way this reaction also turned out to be a practical synthetic method (80% isolated yield for **3a**). The mechanism of these reactions was explored in a combined experimental and theoretical study. NMR studies proved that when starting from **1a** with 2 equiv of *t*-BuOK base, the monoanionic cyclic intermediate **II**^**–**^ (the deprotonated form of **2a**) formed predominantly. DFT calculations clearly described the thermodynamics and the kinetics for the formation of **II**^**–**^ via a closed-shell ionic pathway with the involvement of the ring-opened short-lived intermediate **III**^**–**^. The formation of the thermodynamically stable **2** from its deprotonated anion **II**^**–**^ upon reaction with water is apparent. With a large excess (6 equiv) of the base, enamide dianion **IV**^**2–**^ could be obtained according to ^1^H NMR studies, and again DFT studies supported this pathway. When quenching the reaction mixture with water, **3a** and enamide **5a** were predominantly formed, together with some **2a**. Under the basic conditions present, **5a** transformed during the workup to **3a**, in accordance with the DFT calculations on a base-catalyzed mechanism, which was also supported by deuterium labeling. In a noteworthy way, the final **3a**:**2a** ratio represents the Boltzmann population of **V**^**–**^ to **III**^**–**^ in agreement with the calculations. In addition, the substituent effects of the base-induced (6 equiv) rearrangement of 1,2-benzothiadiazine dioxides (**1**) leading to 1,2-benzisothiazole dioxides (**2**) and to 1,2-benzothiazine dioxides (**3**) was explored, as well. The investigation of the substituent tolerance of these reactions revealed that while the formation of derivatives **2** could be achieved with most substitution patterns, the reaction leading to the formation of compounds **3** is more sensitive. For example, replacement of the N(3)-acyl group to N(3)-alkyl in the starting materials **1** destabilizes the enamide dianion **IV**^**2–**^, preventing the formation of **3**.

## Experimental Section

**Figure 2:**
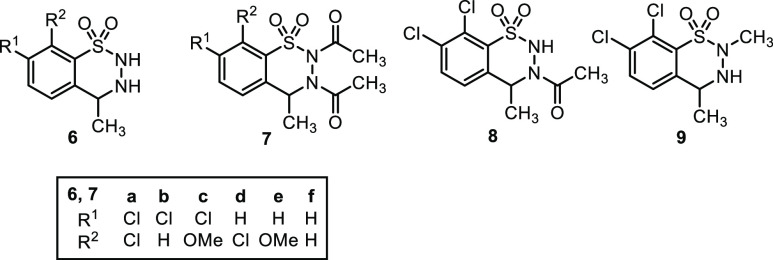
Further starting materials and intermediates used in this study.

Melting points were determined using either a Leica Galen III melting point apparatus; IR spectra were obtained on a Bruker ALPHA FT-IR spectrometer in KBr pellet, and ν̃ was reported in cm^–1^. ^1^H NMR and ^13^C NMR spectra were recorded on a Bruker Avance III 400 (400/100 MHz) or a Bruker Avance III HD 600 (600/150 MHz) spectrometer equipped with a Prodigy cryo-probehead. CDCl_3_ or [D_6_]DMSO was used as the solvent and tetramethylsilane (TMS) as the internal standard, and δ was reported in ppm. Structural assignments were made with additional information from gHSQC and gHMBC experiments. A Waters Acquity UPLC equipment coupled with a Thermo Scientific LTQ XL iontrap MS was used to obtain mass spectroscopic data. High resolution mass spectra were recorded on a micromass GCT or on a Bruker O-TOF MAXIS Impact mass spectrometer coupled with a Dionex Ultimate 3000 RS system with a diode array detector. Single crystal X-ray diffraction measurements were carried out on a Rigaku R-AXIS SPIDER diffractometer using image plate detection and monochromated Cu Kα radiation. The reactions were followed by analytical thin layer chromatography on silica gel 60 F_254_ and HPLC-MS on a Shimadzu LC-20 HPLC utilizing a SPD-M20A diode array detector and a LCMS-2020 spectrometer. Flask heating blocks were used for reactions that required heating. All unspecified reagents were purchased from commercial sources. Compounds **1a**,**b**,^26^**1i**,**j**,^39^**6b**–**f**,^39^**7a**,^26^**8**,^26^ and **9**(39) (Figure 2) were obtained as described previously.

### Synthesis of Starting Materials **7**

#### 2,3-Diacetyl-7-chloro-4-methyl-3,4-dihydro-2*H*-1,2,3-benzothiadiazine 1,1-Dioxide (**7b**)

A mixture of 7-chloro-4-methyl-3,4-dihydro-2*H*-1,2,3-benzothiadiazine 1,1-dioxide (**6b**, 3.69 g, 15.9 mmol) and acetic anhydride (28 mL) was refluxed at 140 °C under stirring for 19 h. Then it was poured onto ice water (70 g). The precipitated product was filtered and washed with H_2_O (2 × 10 mL), cold EtOH (2 × 5 mL), and hexane (2 × 10 mL). Yield 4.49 g (89%); colorless crystals; mp 152.5–153.5 °C (EtOH); ^1^H NMR (600 MHz, CDCl_3_): δ = 7.87 (d, *J* = 2.1 Hz, 1H; 8-H), 7.57 (dd, *J* = 8.4, 2.1 Hz, 1H; 6-H), 7.26 (d, *J* = 8.4 Hz, 1H; 5-H), 6.02 (q, *J* = 7.0 Hz, 1H; 4-H), 2.63 (s, 3H; O=C–CH_3_), 2.13 (s, 3H; O=C–CH_3_), 1.42 (d, *J* = 7.0 Hz, 3H; 4-CH_3_); ^13^C{^1^H} NMR (150 MHz, CDCl_3_): δ = 172.0 (O=C), 167.2 (O=C), 137.2, 136.5, 134.3, 133.6, 128.9, 123.9, 49.3 (C4), 24.3, 20.6, 19.6; IR (KBr): ν̃ = 1741 (s; C=O), 1687 (vs; C=O), 1352 (vs; SO_2_), 1151 (s; SO_2_); HRMS (ESI) *m*/*z*: [M + H]^+^ Calcd for C_12_H_14_ClN_2_O_4_S 317.0357; Found 317.0362.

#### 2,3-Diacetyl-8-chloro-7-methoxy-4-methyl-3,4-dihydro-2*H*-1,2,3-benzothiadiazine 1,1-Dioxide (**7c**)

A mixture of 8-chloro-7-methoxy-4-methyl-3,4-dihydro-2*H*-1,2,3-benzothiadiazine 1,1-dioxide (**6c**, 800 mg, 3.0 mmol) and acetic anhydride (4.5 mL) was refluxed at 140 °C under stirring for 24 h. Then it was poured onto ice water (50 g). The precipitated product was filtered and washed with H_2_O (2 × 10 mL) and hexane (2 × 10 mL). Yield 925 mg (88%); colorless crystals; mp 201–204 °C (EtOH); ^1^H NMR (600 MHz, CDCl_3_): δ = 7.17 (d, *J* = 8.5 Hz, 1H; Ar–H), 7.14 (d, *J* = 8.5 Hz, 1H; Ar–H), 5.93 (q, *J* = 6.9 Hz, 1H; 4-H), 3.95 (s, 3H; OCH_3_), 2.67 (s, 3H; O=C–CH_3_), 2.13 (s, 3H; O=C–CH_3_), 1.40 (d, *J* = 6.9 Hz, 3H; 4-CH_3_); ^13^C{^1^H} NMR (150 MHz, CDCl_3_): δ = 171.8 (O=C), 167.6 (O=C), 134.9 (C), 132.6 (C), 126.6 (CH), 120.0 (C), 115.9 (CH), 56.8 (OCH_3_), 49.6 (C4), 24.8 (O=C–CH_3_), 20.3 (4-CH_3_), 19.5 (O=C-*CH*_*3*_); IR (KBr): ν̃ = 1731 (s; C=O), 1689 (vs; C=O), 1377 (vs; SO_2_), 1157 (s; SO_2_); HRMS (ESI) *m*/*z*: [M + H]^+^ Calcd for C_13_H_16_ClN_2_O_5_S 347.0463; Found 347.0464.

#### 2,3-Diacetyl-8-chloro-4-methyl-3,4-dihydro-2*H*-1,2,3-benzothiadiazine 1,1-Dioxide (**7d**)

A mixture of 8-chloro-4-methyl-3,4-dihydro-2*H*-1,2,3-benzothiadiazine 1,1-dioxide (**6d**, 6.37 g, 27.4 mmol) and acetic anhydride (41 mL) was refluxed at 140 °C under stirring for 17 h. Then it was poured onto ice water (50 g). The precipitated product was filtered and washed with H_2_O (3 × 40 mL). Yield 7.76 g (90%); colorless crystals; mp 171–173 °C (EtOH); ^1^H NMR (600 MHz, CDCl_3_): δ = 7.52–7.46 (m, 2H; Ar–H), 7.22 (dd, *J* = 7.3, 1.7 Hz, 1H; Ar–H), 6.00 (q, *J* = 7.1 Hz, 1H; 4-H), 2.67 (s, 3H; O=C–CH_3_), 2.13 (s, 3H; O=C–CH_3_), 1.44 (d, *J* = 7.1 Hz, 3H; 4-CH_3_); ^13^C{^1^H} NMR (150 MHz, CDCl_3_): δ = 171.8 (C=O), 167.5 (C=O), 142.1 (C), 133.9 (C),133.3 (CH), 131.4 (C), 130.7 (CH), 126.0 (CH), 50.1 (C4), 24.8 (O=C–CH_3_), 20.1 (4-CH_3_), 19.5 (O=C-*CH*_*3*_); IR (KBr): ν̃ = 1741 (s; C=O), 1686 (vs; C=O), 1354 (vs; SO_2_), 1158 (s; SO_2_); HRMS (ESI) *m*/*z*: [M + H]^+^ Calcd for C_12_H_14_ClN_2_O_4_S: 317.0357; Found 317.0360.

#### 2,3-Diacetyl-8-methoxy-4-methyl-3,4-dihydro-2*H*-1,2,3-benzothiadiazine 1,1-Dioxide (**7e**)

A mixture of 8-methoxy-4-methyl-3,4-dihydro-2*H*-1,2,3-benzothiadiazine 1,1-dioxide (**6e**, 3.00 g, 13.1 mmol) and acetic anhydride (19 mL) was refluxed at 140 °C under stirring for 19 h. Then it was poured onto ice water (50 g). The precipitated product was filtered and washed with H_2_O (2 × 20 mL) and hexane (10 mL). Yield 3.80 g (93%); colorless crystals; mp 167–169 °C (EtOH); ^1^H NMR (600 MHz, CDCl_3_): δ = 7.51 (t, *J* = 8.0 Hz, 1H; 6-H), 6.92 (d, *J* = 8.0 Hz, 1H; Ar–H), 6.85 (d, *J* = 8.0 Hz, 1H; Ar–H), 5.94 (q, *J* = 7.0 Hz, 1H; 4-H), 4.01 (s, 3H; OCH_3_), 2.66 (s, 3H; O=C–CH_3_), 2.13 (s, 3H; O=C–CH_3_), 1.41 (d, *J* = 7.0 Hz, 3H; 4-CH_3_); ^13^C{^1^H} NMR (150 MHz, CDCl_3_): δ = 171.9 (C=O), 167.7 (C=O), 157.1 (C), 141.5 (C), 134.1 (CH), 124.2 (C), 119.1 (CH), 111.0 (CH), 56.8 (OCH_3_), 49.6 (C4), 24.8 (O=C–CH_3_), 19.9 (4-CH_3_), 19.5 (O=C-*CH*_*3*_); IR (KBr): ν̃ = 1736 (s; C=O), 1681 (s; C=O), 1347 (s; SO_2_), 1161 (s; SO_2_); HRMS (ESI) *m*/*z*: [M + H]^+^ Calcd for C_13_H_17_N_2_O_5_S 313.0853; Found 313.0858.

#### 2,3-Diacetyl-4-methyl-3,4-dihydro-2*H*-1,2,3-benzothiadiazine 1,1-Dioxide (**7f**)

A mixture of 4-methyl-3,4-dihydro-2*H*-1,2,3-benzothiadiazine 1,1-dioxide (**6f**, 1.86 g, 9.4 mmol) and acetic anhydride (15 mL) was refluxed at 140 °C under stirring for 24 h. Then it was quenched with EtOH (20 mL), evaporated in vacuo, crystallized from hexane (15 mL), filtered and washed with H_2_O (10 mL) and EtOH (5 mL). Yield 2.32 g (87%); colorless crystals; mp 155–157 °C (EtOH); ^1^H NMR (600 MHz, CDCl_3_): δ = 7.89 (d, *J* = 7.7 Hz, 1H; Ar–H), 7.61 (td, *J* = 7.7, 1.3 Hz, 1H; Ar–H), 7.49 (t, *J* = 7.7 Hz, 1H; Ar–H), 7.31 (d, *J* = 7.7 Hz, 1H; Ar–H), 6.04 (q, *J* = 7.1 Hz, 1H; 4-H), 2.64 (s, 3H; O=C–CH_3_), 2.14 (s, 3H; O=C–CH_3_), 1.44 (d, *J* = 7.1 Hz, 3H; 4-CH_3_); ^13^C{^1^H} NMR (150 MHz, CDCl_3_): δ = 172.1 (C=O), 167.4 (C=O), 138.9 (C), 135.3 (C), 133.3 (CH), 128.1 (CH), 127.5 (CH), 123.9 (CH), 49.6 (C4), 24.4 (O=C–CH_3_), 20.6 (4-CH_3_), 19.7 (O=C–CH_3_); IR (KBr): ν̃ = 1745 (s; C=O), 1690 (vs; C=O), 1347 (s; SO_2_); HRMS (ESI): *m*/*z* calcd for C_12_H_15_N_2_O_4_S 283.0747; Found 283.0752.

### Synthesis of 2,3-Disubstituted Benzothiadiazine Dioxides (**1**)

#### 3-Acetyl-7,8-dichloro-2-ethyl-4-methyl-3,4-dihydro-2*H*-1,2,3-benzothiadiazine 1,1-Dioxide (**1c**)

To a suspension of *t*-BuOK (449 mg, 4.0 mmol) in DMF (4 mL) was added a solution of 2,3-diacetyl-7,8-dichloro-4-methyl-3,4-dihydro-2*H*-1,2,3-benzothiadiazine 1,1-dioxide (**7a**, 702 mg, 2.0 mmol) in DMF (8 mL) at room temperature. After 30 min, EtI (485 μL, 936 mg, 6.0 mmol) was added dropwise. The mixture was stirred for 4 h. It was poured into ice water (60 g). The precipitated product was filtered. Yield 620 mg (92%); colorless crystals; mp 146–147 °C (EtOH); ^1^H NMR (600 MHz, [D_6_]DMSO): δ = 8.00 (d, *J* = 8.7 Hz, 1H; 6-H), 7.69 (d, *J* = 8.7 Hz, 1H; 5-H), 5.69 (q, *J* = 7.0 Hz, 1H; 4-H), 3.84 (dq, 1H, *J* = 13.9, 7.1 Hz; N–CH), 3.22 (dq, 1H, *J* = 13.9, 7.1 Hz; N–CH), 2.22 (s, 3H; O=C–CH_3_), 1.67 (d, *J* = 7.0 Hz, 4-CH_3_), 1.28 (t, *J* = 7.1 Hz, 3H, CH_2_–C*H*_*3*_); ^13^C{^1^H} NMR (150 MHz, [D_6_]DMSO): δ = 172.8 (C=O), 139.5 (C4a), 134.3 (C6), 132.8 (C8a*), 132.7(C7*), 128.8 (C5), 128.6 (C8), 48.2 (N–CH_2_), 46.8 (C4), 21.5 (4-CH_3_), 20.6 (O=C-*CH*_*3*_), 12.5 (CH_2_–*CH*_*3*_); IR (KBr): ν̃ = 1672 (vs; C=O), 1366 (vs; SO_2_), 1187 (s; SO_2_); HRMS (ESI) *m*/*z*: [M + H]^+^ Calcd for C_12_H_15_Cl_2_N_2_O_3_S 337.0175; Found 337.0179.

#### 3-Acetyl-2-benzyl-7,8-dichloro-4-methyl-3,4-dihydro-2*H*-1,2,3-benzothiadiazine 1,1-Dioxide (**1d**)

To a suspension of *t*-BuOK (449 mg, 4.0 mmol) in DMF (4 mL) was added a solution of **7a** (702 mg, 2.0 mmol) in DMF (8 mL) at room temperature. After 30 min, BnBr (713 μL, 1026 mg, 6.0 mmol) was added dropwise. The mixture was stirred for 4 h. It was poured into ice water (60 g). The precipitated product was filtered, washed with hexane (3 × 5 mL). Yield 722 mg (90%); colorless crystals; mp 187–189 °C (iPrOH); ^1^H NMR (400 MHz, [D_6_]DMSO): δ = 8.04 (d, *J* = 8.6 Hz, 1H; 6-H); 7.73 (d, *J* = 8.6 Hz, 1H; 5-H); 7.47 (d, *J* = 7.5 Hz, 2H; *o*-H); 7.43 (t, *J* = 7.5 Hz, 2H; *m*-H); 7.39 (t, *J* = 7.5 Hz, 1H; *p*-H); 5.72 (q, *J* = 6.9 Hz, 1H; 4-H); 5.00 (d, *J* = 14.5 Hz, 1H; N–CH); 4.42 (d, *J* = 14.5 Hz, 1H; N–CH); 1.82 (d, *J* = 6.9 Hz, 3H; 4-CH_3_); 1.80 (s, 3H; O=C–CH_3_); ^13^C{^1^H} NMR (100 MHz, [D_6_]DMSO): δ = 172.7 (C=O), 139.7 (C), 134.8 (C), 134.4 (CH), 133.4 (C), 132.8 (C), 129.9 (CH), 128.91 (CH), 128.88 (CH), 128.6 (CH), 128.5 (C), 57.0 (2-CH_2_), 47.5 (C4), 22.3 (4-CH_3_), 20.2 (O=C-*CH*_*3*_); IR (KBr): ν̃ = 1685 (vs; C=O), 1365 (s; SO_2_), 1178 (s; SO_2_); HRMS (ESI) *m*/*z*: [M + H]^+^ Calcd for C_17_H_17_Cl_2_N_2_O_3_S 399.0331; Found 399.0332.

#### 7,8-Dichloro-2,4-dimethyl-3-propionyl-3,4-dihydro-2*H*-1,2,3-benzothiadiazine 1,1-Dioxide (**1e**)

A mixture of 7,8-dichloro-2,4-dimethyl-3,4-dihydro-2*H*-1,2,3-benzothiadiazine 1,1-dioxide (**9**, 700 mg, 2.5 mmol) and propionic anhydride (16 mL) was heated at 130 °C under stirring for 16 h. Then it was quenched with H_2_O (20 mL), extracted with EtOAc (20 mL), the organic layer was washed with H_2_O (20 mL), aq NaHCO_3_ (saturated, 20 mL) and brine (10 mL), dried over MgSO_4_ and evaporated. The residue was treated with H_2_O (15 mL), decanted, stirred in hexane–diethyl ether (1:1, 10 mL) under cooling, filtered, and washed with hexane. Yield 498 mg (59%); light yellow crystals; mp 135–137 °C (EtOH); ^1^H NMR (600 MHz, [D_6_]DMSO): δ = 8.00 (d, *J* = 8.7 Hz, 1H; 6-H), 7.69 (d, *J* = 8.7 Hz, 1H; 5-H), 5.67 (q, *J* = 7.0 Hz, 1H; 4-H), 3.26 (s, 3H; NCH_3_), 2.69 (dq, *J* = 17.4, 7.4, 1H; O=C–CH), 2.56 (dq, *J* = 17.4, 7.4, 1H; O=C–CH), 1.67 (d, *J* = 7.0 Hz, 3H; 4-CH_3_), 1.04 (t, *J* = 7.4 Hz, 3H; CH_2_–C*H*_*3*_); ^13^C{^1^H} NMR (150 MHz, [D_6_]DMSO): δ = 175.0 (C=O), 139.8 (C4a), 134.2 (C6), 132.8 (C7), 132.5(C8a), 128.9 (C5), 128.6 (C8), 47.0 (C4), 41.2 (NCH_3_), 24.8 (4-CH_3_), 22.1 (O=C-*CH*_*2*_), 8.7 (CH_2_–*CH*_*3*_); IR (KBr): ν̃ = 1678 (vs; C=O), 1358 (s; SO_2_); HRMS (ESI) *m*/*z*: [M + H]^+^ Calcd for C_12_H_15_Cl_2_N_2_O_3_S 337.0175; Found 337.0179.

#### 3-Butyryl-7,8-dichloro-2,4-dimethyl-3,4-dihydro-2*H*-1,2,3-benzothiadiazine 1,1-Dioxide (**1f**)

A mixture of **9** (703 mg, 2.5 mmol) and butyric anhydride (20 mL) was heated at 130 °C under stirring for 15 h. Then it was quenched with H_2_O (20 mL), extracted with EtOAc (20 mL), the organic layer was washed with H_2_O (20 mL), aq NaHCO_3_ (saturated, 20 mL) and brine (10 mL), dried over MgSO_4_, and evaporated. The residue was treated and washed with diethyl ether (4, then 2 × 2 mL). Yield 545 mg (62%); light yellow crystals; mp 141–143 °C (EtOH); ^1^H NMR (600 MHz, [D_6_]DMSO): δ = 8.00 (d, *J* = 8.6 Hz, 1H; Ar–H), 7.69 (dd, *J* = 8.8, 0.8 Hz, 1H; Ar–H), 5.70–5.65 (m, 1H; 4-H), 3.26 (s, 3H; 2-CH_3_), 2.64 (dt, *J* = 16.7, 7.5, 1H; O=C–CH), 2.54 (dt, *J* = 16.7, 7.5, 1H; O=C–CH), 1.67 (d, *J* = 7.1 Hz, 3H; 4-CH_3_), 1.61–1.53 (m, 2H; *CH*_*2*_-CH_3_), 0.92 (t, *J* = 7.3 Hz, 3H; CH_2_–*CH*_*3*_); ^13^C{^1^H} NMR (150 MHz, [D_6_]DMSO): δ = 174.2 (C=O), 139.8, 134.2, 132.8, 132.5, 128.9, 128.6, 47.0, 41.3, 33.2, 22.1, 17.5, 13.8; IR (KBr): ν̃ = 1680 (vs; C=O), 1359 (vs; SO_2_), 1183 (s; SO_2_); HRMS (ESI) *m*/*z*: [M + H]^+^ Calcd for C_13_H_17_Cl_2_N_2_O_3_S 351.0331; Found 351.0336.

#### 7,8-Dichloro-3-trifluoroacetyl-2,4-dimethyl-3,4-dihydro-2*H*-1,2,3-benzothiadiazine 1,1-Dioxide (**1g**)

A suspension of **9** (703 mg, 2.5 mmol) and trifluoroacetic anhydride (20 mL) was stirred at room temperature for 14 h. The precipitated product was filtered and washed with H_2_O. Yield 743 mg (79%); colorless crystals; mp 151–152 °C (EtOH); ^1^H NMR (600 MHz, [D_6_]DMSO): δ = 8.08 (d, *J* = 8.7 Hz, 1H; Ar–H), 7.73 (d, *J* = 8.7 Hz, 1H; Ar–H), 5.68 (q, *J* = 6.9 Hz, 1H; 4-H), 3.27 (s, 3H; 2-CH_3_), 1.80 (d, *J* = 6.9 Hz, 3H; 4-CH_3_); ^13^C{^1^H} NMR (150 MHz, [D_6_]DMSO): δ = 156.7 (q, *J* = 36.9 Hz; C=O), 137.7, 134.7, 133.4, 131.2, 129.0, 128.8, 115.9 (q, *J* = 288.8 Hz; CF_3_); 49.1 (4C), 42.1 (2-CH_3_), 21.4 (4-CH_3_); IR (KBr): ν̃ = 1721 (s; C=O), 1373 (vs; SO_2_), 1187 (vs; SO_2_); HRMS (ESI) *m*/*z*: [M + NH_4_]^+^ Calcd for C_11_H_13_F_3_Cl_2_N_3_O_3_S 394.0001; Found 394.0001.

#### 7,8-Dichloro-3-difluoroacetyl-2,4-dimethyl-3,4-dihydro-2*H*-1,2,3-benzothiadiazine 1,1-Dioxide (**1h**)

To **9** (463 mg, 1.65 mmol) was added difluoroacetic anhydride (3 mL) under ice water cooling, and then it was stirred at room temperature for 2.5 h. The mixture was poured into ice water (40 g), the precipitated product was filtered and washed with water. Yield 564 mg (95%); colorless crystals; mp 162–163 °C (EtOH); ^1^H NMR (600 MHz, CDCl_3_): δ = 7.73 (d, *J* = 8.6 Hz, 1H; 6-H), 7.29 (d, *J* = 8.6 Hz, 1H; 5-H), 6.65 (dd, *J* = 54.8, 51.6 Hz, 1H; CHF_2_), 5.62 (q, *J* = 7.2 Hz, 1H; 4-H), 3.28 (s, 3H; 2-CH_3_), 1.82 (d, *J* = 7.2 Hz, 3H; 4-CH_3_); ^13^C{^1^H} NMR (150 MHz, CDCl_3_): δ = 163.2 (dd, *J* = 30.1, 24.2 Hz; C=O), 136.8 (C4a), 135.1 (C7), 134.1 (C6), 132.6 (C8a), 131.4 (C8), 126.4 (C5), 105.8 (dd, *J* = 244.2, 246.9 Hz; CHF_2_); 47.9 (4C), 41.7 (2-CH_3_), 22.2 (4-CH_3_); IR (KBr): ν̃ = 1716 (s; C=O), 1368 (s; SO_2_), 1065 (m; SO_2_); HRMS (ESI) *m*/*z*: [M + Na] C_11_H_10_F_2_Cl_2_N_2_O_3_S 380.8649; Found 380.8652.

#### 3-Acetyl-7-chloro-2,4-dimethyl-3,4-dihydro-2*H*-1,2,3-benzothiadiazine 1,1-Dioxide (**1k**)

To a mixture of **7b** (1.58 g, 5.0 mmol) and DMF (10 mL) was added *t*-BuOK (1.12 g, 10.0 mmol) and DMF (20 mL) at room temperature. After 10 min, MeI (934 μL, 2.13 g, 15.0 mmol) was added dropwise. The mixture was stirred for 4.5 h. It was poured into ice water (160 g). The precipitated product was filtered. Yield 697 mg (48%); beige crystals; mp 156–157 °C (EtOH); ^1^H NMR (600 MHz, [D_6_]DMSO): δ = 7.98 (d, *J* = 2.2 Hz, 1H; H-8), 7.84 (dd, *J* = 8.6, 2.2 Hz, 1H; H-6), 7.72 (d, *J* = 8.6 Hz, 1H; H-5), 5.61 (q, *J* = 7.0 Hz, 1H; 4-H), 3.13 (s, 3H; NCH_3_), 2.21 (s, 3H; O=C–CH_3_), 1.63 (d, *J* = 7.0 Hz, 3H; 4-CH_3_); ^13^C{^1^H} NMR (150 MHz, [D_6_]DMSO): δ = 172.4 (C=O), 136.6, 133.6, 133.0, 132.6, 130.7, 124.0, 46.1 (C4), 40.4 (NCH_3_), 22.4 (4-CH_3_), 20.9 (O=C-*CH*_*3*_); IR (KBr): ν̃ = 1686 (vs; C=O), 1343 (vs; SO_2_), 1164 (s; SO_2_); HRMS (ESI) *m*/*z*: [M + H]^+^ Calcd for C_11_H_14_ClN_2_O_3_S 289.0414; Found 289.0413.

#### 3-Acetyl-8-chloro-7-methoxy-2,4-dimethyl-3,4-dihydro-2*H*-1,2,3-benzothiadiazine 1,1-Dioxide (**1l**)

To a suspension of *t*-BuOK (324 mg, 2.9 mmol) in DMF (3 mL) was added a solution of **7c** (500 mg, 1.4 mmol) in DMF (6.5 mL) at room temperature. After 30 min, MeI (269 μL, 614 mg, 4.3 mmol) was added dropwise. The mixture was stirred for 2.5 h. It was poured into ice water (50 g). The precipitated product was filtered. Yield 415 mg (90%); colorless crystals; mp 176–177 °C; ^1^H NMR (400 MHz, [D_6_]DMSO): δ = 7.62 (d, *J* = 8.7 Hz, 1H; Ar–H), 7.52 (d, *J* = 8.7 Hz, 1H; Ar–H), 5.56 (q, *J* = 7.0 Hz, 1H; 4-H), 3.94 (s, 3H; OCH_3_), 3.23 (s, 3H; NCH_3_), 2.21 (s, 3H; O=C–CH_3_), 1.65 (d, *J* = 7.0 Hz, 3H; 4-CH_3_); ^13^C{^1^H} NMR (100 MHz, [D_6_]DMSO): δ = 172.1 (C=O), 154.6, 131.4, 131.2, 128.3, 118.4, 117.2, 57.1 (OCH_3_), 46.5 (C4), 41.0 (NCH_3_), 22.4 (4-CH_3_), 20.6 (O=C-*CH*_*3*_); IR (KBr): ν̃ = 1678 (s; C=O), 1349 (vs; SO_2_), 1292; HRMS (ESI) *m*/*z*: [M + H]^+^ Calcd for C_12_H_16_ClN_2_O_4_S 319.0514; Found 319.0518.

#### 3-Acetyl-8-chloro-2,4-dimethyl-3,4-dihydro-2*H*-1,2,3-benzothiadiazine 1,1-Dioxide (**1m**)

To a suspension of *t*-BuOK (1.06 g, 9.5 mmol) in DMF (10 mL) was added a solution of **7d** (1.50 g, 4.7 mmol) in DMF (20 mL) at room temperature. After 10 min, MeI (884 μL, 2.02 g, 14.2 mmol) was added dropwise. The mixture was stirred for 85 min. It was poured into ice water (150 g). The precipitated product was filtered. Yield 1.21 g (89%); colorless crystals; mp 133–134 °C (EtOH); ^1^H NMR (600 MHz, [D_6_]DMSO): δ = 7.70 (t, *J* = 7.9 Hz, 1H; 6-H), 7.67–7.63 (m, 2H; Ar–H), 5.65 (q, *J* = 7.0 Hz, 1H; 4-H), 3.26 (s, 3H; NCH_3_), 2.22 (s, 3H; O=C–CH_3_), 1.68 (d, *J* = 7.0 Hz, 3H; 4-CH_3_); ^13^C{^1^H} NMR (150 MHz, [D_6_]DMSO): δ = 172.2 (C=O), 141.0 (C), 133.9 (C), 130.6 (CH), 130.5 (C), 127.5 (CH), 46.8 (C4), 40.9 (NCH_3_), 22.3 (4-CH_3_), 20.6 (O=C–CH_3_) (one quaternary C signal not resolved); IR (KBr): ν̃ = 1686 (vs; C=O), 1351 (vs; SO_2_), 1166 (m; SO_2_); HRMS (ESI) *m*/*z*: [M + H]^+^ Calcd for C_11_H_14_ClN_2_O_3_S 289.0408; Found 289.0412.

#### 3-Acetyl-8-methoxy-2,4-dimethyl-3,4-dihydro-2*H*-1,2,3-benzothiadiazine 1,1-Dioxide (**1n**)

To a suspension of *t*-BuOK (1.80 g, 1.60 mmol) in DMF (1.5 mL) was added a solution of **7e** (200 mg, 0.64 mmol) in DMF (2.5 mL) at room temperature. After 30 min, MeI (140 μL, 318 mg, 2.24 mmol) was added dropwise. The mixture was stirred for 2 h. It was poured into ice water (20 g). The precipitated product was filtered. Yield 140 mg (77%); colorless crystals; mp 189–190 °C (EtOH); ^1^H NMR (400 MHz, [D_6_]DMSO): δ = 7.89–7.47 (m, 1H; Ar–H), 7.47–6.88 (m, 2H; Ar–H), 5.82–5.27 (m, 1H; 4-H), 3.90 (s, 3H; OCH_3_), 3.17 (s, 3H; NCH_3_), 2.19 (s, 3H; O=C–CH_3_), 1.88–1.31 (m, 3H; 4-CH_3_) (broad signals); ^13^C{^1^H} NMR (100 MHz, [D_6_]DMSO): δ = 172.2 (C=O), 157.2 (C), 139.8 (C), 134.2 (CH), 120.8 (C), 119.6 (CH), 111.7 (CH), 56.8 (OCH_3_), 46.2 (C4), 40.8 (NCH_3_), 22.3 (4-CH_3_), 20.7 (O=C-*CH*_*3*_); IR (KBr): ν̃ = 1688 (vs; C=O), 1345 (vs; SO_2_), 1159 (m; SO_2_); HRMS (ESI) *m*/*z*: [M + H]^+^ Calcd for C_12_H_17_N_2_O_4_S 285.0904; Found 285.0904.

#### 3-Acetyl-2,4-dimethyl-3,4-dihydro-2*H*-1,2,3-benzothiadiazine 1,1-Dioxide (**1o**)

To a suspension of *t*-BuOK (898 mg, 8.0 mmol) in DMF (7 mL) was added a solution of **7f** (1129 mg, 4.0 mmol) in DMF (10 mL) at room temperature. After 30 min, MeI (747 μL, 1703 mg, 12.0 mmol) was added dropwise. The mixture was stirred for 2 h. It was poured into ice water (100 g). The precipitated product was filtered. Yield 861 mg (85%); colorless crystals; mp 115–117 °C (EtOH); ^1^H NMR (600 MHz, [D_6_]DMSO): δ = 7.88 (dd, 1H, *J* = 7.8, 1.1 Hz; Ar–H), 7.75 (td, 1H, *J* = 7.8, 1.1 Hz; Ar–H), 7.68 (d, 1H, *J* = 7.8 Hz; Ar–H) 7.59 (t, 1H, *J* = 7.8 Hz; Ar–H), 5.60 (q, *J* = 7.0 Hz, 1H; 4-H), 3.11 (s, 3H; NCH_3_), 2.22 (s, 3H; O=C–CH_3_), 1.65 (d, *J* = 7.0 Hz, 3H; 4-CH_3_); ^13^C{^1^H} NMR (150 MHz, [D_6_]DMSO): δ = 172.4 (C=O), 137.6, 133.6, 130.9, 128.6, 128.3, 124.4, 46.2 (C4), 40.4 (NCH_3_), 22.5 (4-CH_3_), 20.9 (O=C-*CH*_*3*_); IR (KBr): ν̃ = 1677 (vs; C=O), 1342 (vs; SO_2_), 1183 (m; SO_2_); HRMS (ESI) *m*/*z*: [M + H]^+^ Calcd for C_11_H_15_N_2_O_3_S 255.0798; Found 255.0802.

#### 3-Acetyl-7,8-dichloro-2-(methylsulfonyl)-4-methyl-3,4-dihydro-2*H*-1,2,3-benzothiadiazine 1,1-Dioxide (**1p**)

To a stirred mixture of **8** (500 mg, 1.6 mmol) and TEA (0.5 mL, 3.6 mmol) in DCM (6.5 mL) was added MsCl in two portions (250 μL, 3.2 mmol and after 2 h 125 μL, 1.6 mmol) at room temperature. After 4 h stirring, it was poured into ice water (20 g), and the precipitated product was filtered. Yield 425 mg (68%); colorless crystals; mp 226–227 °C (EtOH); ^1^H NMR (600 MHz, CDCl_3_): δ = 7.69 (d, *J* = 8.5 Hz, 1H; Ar–H), 7.24 (dd, *J* = 8.5, 0.7 Hz, 1H; Ar–H), 5.88 (∼qd, *J* = 7.1, 0.7 Hz, 1H; 4-H), 3.66 (s, 3H; SO_2_CH_3_), 3.11 (s, 3H; O=C–CH_3_), 1.72 (d, *J* = 7.1 Hz, 3H; 4-CH_3_); ^13^C{^1^H} NMR (150 MHz, CDCl_3_): δ = 172.3 (C=O), 138.6 (C), 134.8 (C), 134.33 (C), 134.31 (CH), 130.0 (C), 127.1 (CH), 49.6 (C4), 45.1 (CH_3_), 20.7 (CH_3_), 19.9 (CH_3_); IR (KBr): ν̃ = 1705 (s; C=O), 1372 (vs; SO_2_), 1158 (s; SO_2_); HRMS (ESI) *m*/*z*: [M + H]^+^ Calcd for C_11_H_13_Cl_2_N_2_O_5_S_2_ 386.9637; Found 386.9636.

#### 3-Acetyl-7,8-dichloro-4-methyl-2-(4-toluenesulfonyl)-3,4-dihydro-2*H*-1,2,3-benzothiadiazine 1,1-Dioxide (**1q**)

To a stirred mixture of 3-acetyl-7,8-dichloro-4-methyl-3,4-dihydro-2*H*-1,2,3-benzothiadiazine 1,1-dioxide (**8**, 500 mg, 1.6 mmol) and TEA (0.5 mL, 3.6 mmol) in DCM (8.5 mL) was added TsCl (617 mg, 3.2 mmol) at room temperature. After 1.5 h stirring, it was poured into ice water (30 g), and the precipitated product was filtered. Yield 490 mg (65%); colorless crystals; mp 243–244 °C (EtOAc); ^1^H NMR (600 MHz, CDCl_3_): δ = 7.96 (d, *J* = 8.3 Hz, 2H; *o*-H), 7.67 (d, *J* = 8.6 Hz, 1H; 6-H), 7.42 (d, *J* = 8.3 Hz, 2H; *m*-H), 7.25 (d, *J* = 8.6 Hz, 1H; 5-H), 5.92 (q, *J* = 7.1 Hz, 1H; 4-H), 2.50 (s, 3H; *p*-CH_3_), 2.36 (s, 3H; O=C–CH_3_), 1.85 (d, *J* = 7.1 Hz, 3H; 4-CH_3_); ^13^C{^1^H} NMR (150 MHz, CDCl_3_): δ = 172.9 (C=O), 147.1 (*p*-C), 138.7 (C4a), 135.3 (C8a), 134.4 (C7), 134.1 (ipso-C), 134.0 (C6), 130.0 (4C; *o*-C and *m*-C), 129.9 (C8), 126.9 (C5), 49.6 (C4), 21.9 (*p*-CH_3_), 20.8 (4-CH_3_), 20.2 (O=C-*CH*_*3*_); IR (KBr): ν̃ = 1698 (vs; C=O), 1376 (vs; SO_2_), 1169 (s; SO_2_); HRMS (ESI) *m*/*z*: [M + H]^+^ Calcd for C_17_H_17_Cl_2_N_2_O_5_S_2_ 462.9950; Found 462.9956.

### Preparation of 1,2-Benzisothiazole 1,1-Dioxides (**2**)

#### General Procedure for the Synthesis of Compounds **2a**–**f**,**i**,**j**

Method A. To the mixture of **1a**–**d**,**f**,**i** (0.60 mmol) in THF (3 mL) was added *t*-BuOK (1.2 mmol, 135 mg) at 25 °C and stirred for 30 min. Then, it was quenched with water (10 mL), and THF was evaporated. After stirring and cooling with ice water bath for 1 h, the precipitated product was filtered and washed with water to give **2a**–**d**,**f**,**i**. Method B. To the solution of **1e**,**j** (0.30 mmol) in THF (1.5 mL) was added *t*-BuOK (0.60 mmol, 67 mg) at 25 °C and stirred for 30 min. Then, it was quenched with water (5 mL), and THF was evaporated. After stirring and cooling with an ice water bath for 1 h, the precipitated product was filtered and washed with water to give **2e**,**j**.

#### *N*-(6,7-Dichloro-2,3-dimethyl-1,1-dioxo-2,3-dihydro-1,2-benzisothiazol-3-yl)acetamide (**2a**)

Using **1a** (194 mg). Yield 174 mg (90%). Identical with our previous report;^26^ mp 212 °C (EtOH); ^1^H NMR (600 MHz, [D_6_]DMSO): δ = 8.79 (s, 1H; NH), 7.94 (d, *J* = 8.4 Hz, 1H; 5-H), 7.57 (d, *J* = 8.4 Hz; 4-H), 2.67 (s, 3H; NCH_3_), 1.82 (s, 3H; O=C–CH_3_), 1.56 (s, 3H; 3-CH_3_); ^13^C{^1^H} NMR (600 MHz, [D_6_]DMSO): δ = 168.6 (O=C), 143.4 (C3a), 135.0 (C5), 132.8 (C7a), 132.5 (C6), 124.7 (C7), 123.3 (C4), 71.5 (C3), 25.2 (3-CH_3_), 23.0 (O=C-*CH*_*3*_), 22.7 (NCH_3_). Scaled-up experiment with modified workup: To the mixture of **1a** (1.2 mmol, 389 mg) in THF (6 mL) was added *t*-BuOK (2.4 mmol, 269 mg) at 25 °C and stirred for 30 min. Then, THF was evaporated and aq HCl (5 w/w%, 10 mL) was added to the residue. Upon cooling with ice water, the precipitated product was filtered and washed with water to give **2a** (347 mg, 90%).

#### *N*-(6,7-Dichloro-3-ethyl-2-methyl-1,1-dioxo-2,3-dihydro-1,2-benzisothiazol-3-yl)acetamide (**2b**)

Using **1b** (202 mg). Yield 187 mg (92%). Identical with our previous report;^26^ mp 209–210 °C (EtOH); ^1^H NMR (600 MHz, [D_6_]DMSO): δ = 8.67 (s, 1H; NH), 7.94 (d, *J* = 8.3 Hz, 1H; 5-H), 7.52 (d *J* = 8.3 Hz, 1H; 4-H), 2.63 (s, 3H; NCH_3_), 2.13–1.94 (m, 2H; 3-CH_2_), 1.81 (s, 3H; O=C–CH_3_), 0.48 (t, *J* = 7.2 Hz, 3H; CH_2_–*CH*_*3*_); ^13^C{^1^H} NMR (150 MHz, [D_6_]DMSO): δ = 168.8 (O=C), 141.0 (C3a), 134.9 (C5), 134.2 (C7a), 132.5 (C6), 124.7 (C7), 123.2 (C4), 75.0 (C3), 29.5 (CH_2_), 22.9 (O=C-*CH*_*3*_), 22.4 (NCH_3_), 6.9 (CH_2_–*CH*_*3*_).

#### *N*-(6,7-Dichloro-2-ethyl-3-methyl-1,1-dioxo-2,3-dihydro-1,2-benzisothiazol-3-yl)acetamide (**2c**)

Using **1c** (202 mg). Yield 172 mg (85%); colorless crystals; mp 221–222 °C (EtOH); ^1^H NMR (600 MHz, [D_6_]DMSO): δ = 8.83 (s, 1H; NH), 7.92 (d, *J* = 8.4 Hz, 1H; 5-H), 7.54 (d, *J* = 8.4 Hz, 1H; 4-H), 3.31–3.15 (m, 2H; 2-CH_2_), 1.82 (s, 3H; O=C–CH_3_), 1.59 (s, 3H; 3-CH_3_), 1.26 (t, *J* = 7.2 Hz, 3H; CH_2_–*CH*_*3*_); DEPTQ (150 MHz, [D_6_]DMSO): δ = 168.1 (C=O), 143.4 (C3a), 134.9 (C5), 133.0 (C7a), 132.4 (C6), 124.6 (C7), 123.2 (C4), 71.9 (C3), 33.6 (2-CH_2_), 26.6 (3-CH_3_), 23.0 (O=C–CH_3_), 15.3 (CH_2_–*CH*_*3*_); IR (KBr): ν̃ = 3265 (m; NH), 1672 (s; C=O), 1305 (vs; SO_2_), 1191 (vs; SO_2_); HRMS (ESI) *m*/*z*: [M + H]^+^ Calcd for C_12_H_15_Cl_2_N_2_O_3_S 337.0175; Found 337.0175.

#### *N*-(2-Benzyl-6,7-dichloro-3-methyl-1,1-dioxo-2,3-dihydro-1,2-benzisothiazol-3-yl)acetamide (**2d**)

Using **1d** (240 mg). Yield 226 mg (94%); colorless crystals; mp 225–227 °C (EtOH); ^1^H NMR (600 MHz, [D_6_]DMSO): δ = 8.71 (s, 1H; NH), 7.94 (d, *J* = 8.3 Hz, 1H; 5-H), 7.52 (d, *J* = 8.4 Hz, 1H; 4-H), 7.43 (d, *J* = 7.3 Hz, 2H; *o*-H), 7.33 (t, *J* = 7.3 Hz, 2H; *m*-H), 7.27 (t, *J* = 7.3 Hz, 1H; *p*-H), 4.39 (s, 2H; 2-CH_2_), 1.54 (s, 3H; 3-CH_3_), 1.53 (s, 3H; O=C–CH_3_); ^13^C{^1^H} NMR (150 MHz, [D_6_]DMSO): δ = 168.8 (O=C), 143.5 (C3a), 137.1 (ipso-C), 135.1 (C5), 132.6 (C7a*), 132.5 (C6*), 128.3 (2C; *o*-C), 128.2 (2C; *m*-C), 127.4 (*p*-C), 124.8 (C7), 123.2 (C4), 72.1 (C3), 41.6 (2-CH_2_), 26.6 (3-CH_3_), 22.6 (O=C-*CH*_*3*_); IR (KBr): ν̃ = 3268 (m; NH), 3067 (m; C = C–H), 1673 (s; C=O), 1300 (vs; SO_2_), 1174 (vs; SO_2_); HRMS (ESI) *m*/*z*: [M + H]^+^ Calcd for C_17_H_17_Cl_2_N_2_O_3_S: 339.0331; Found 339.0332.

#### *N*-(6,7-Dichloro-2,3-dimethyl-1,1-dioxo-2,3-dihydro-1,2-benzisothiazol-3-yl)propionamide (**2e**)

Using **1e** (101 mg). Yield 86 mg (85%); colorless crystals; mp 224–225 °C (EtOH); ^1^H NMR (600 MHz, [D_6_]DMSO): δ = 8.71 (s, 1H; NH), 7.94 (d, *J* = 8.3 Hz, 1H; 5-H), 7.55 (d, *J* = 8.3 Hz, 1H; 4-H), 2.66 (s, 3H; 2-CH_3_), 2.19–2.06 (m, 2H; *CH*_*2*_-CH_3_), 1.56 (s, 3H), 0.89 (t, *J* = 7.5 Hz, 3H; CH_2_–*CH*_*3*_); ^13^C{^1^H} NMR (150 MHz, [D_6_]DMSO): δ = 172.5 (C=O), 143.5 (C3a), 135.0 (C5), 132.8 (C7a), 132.5 (C6), 124.7 (C7), 123.2 (C4), 71.5 (C3), 28.4 (O=C-*CH*_*2*_), 25.2 (3-CH_3_), 22.7 (2-CH_3_), 9.5 (CH_2_–*CH*_*3*_); IR (KBr): ν̃ = 3262 (w; NH), 1669 (m; C=O), 1306 (s; SO_2_), 1155 (s; SO_2_); HRMS (ESI) *m*/*z*: [M + H]^+^ Calcd for C_12_H_15_Cl_2_N_2_O_3_S 337.0175; Found 337.0174.

#### *N*-(6,7-Dichloro-2,3-dimethyl-1,1-dioxo-2,3-dihydro-1,2-benzisothiazol-3-yl)butyramide (**2f**)

Using **1f** (211 mg). Yield 200 mg (95%); colorless crystals; mp 258–260 °C (EtOH); ^1^H NMR (600 MHz, [D_6_]DMSO): δ = 8.73 (s, 1H; NH), 7.95 (d, *J* = 8.3 Hz, 1H; 5-H), 7.54 (d, *J* = 8.3 Hz, 1H; 4-H), 2.66 (s, 3H; 2-CH_3_), 2.14–2.03 (m, 2H; O=C–CH_2_), 1.56 (3-CH_3_), 1.46–1.37 (m, 2H; *CH*_*2*_-CH_3_), 0.80 (t, *J* = 7.4 Hz, 3H; CH_2_–*CH*_*3*_); ^13^C{^1^H} NMR (150 MHz, [D_6_]DMSO): δ = 171.6 (C=O), 143.5 (C3a), 135.0 (C5), 132.9 (C7a), 132.5 (C6), 124.7 (C7), 123.2 (C4), 71.5 (C3), 37.1 (O=C-*CH*_*2*_*)*, 25.2 (3-CH_3_), 22.7 (2-CH_3_), 18.4 (CH_2_–*CH*_*2*_-CH_3_), 13.7 (CH_2_–*CH*_*3*_); IR (KBr): ν̃ = 3258 (m; NH), 3205 (m; NH), 3067 (C=C–H), 1663 (s; C=O), 1301 (s; SO_2_), 1159 (s; SO_2_); HRMS (ESI) *m*/*z*: [M + H]^+^ Calcd for C_13_H_17_Cl_2_N_2_O_3_S 351.0332; Found 351.0331.

#### *N*-(6,7-Dichloro-2,3-dimethyl-1,1-dioxo-2,3-dihydro-1,2-benzisothiazol-3-yl)trifluoroacetamide (**2g**)

To the solution of **1g** (226 mg) in THF (3 mL) was added *t*-BuOK (1.2 mmol, 135 mg) at 25 °C and stirred for 30 min. Then, it was quenched with water (10 mL), and THF was evaporated. After stirring and cooling with ice water bath, the precipitated side product was filtered. The filtrate was extracted with DCM (3 × 15 mL), the combined organic layer was dried over MgSO_4_ and evaporated. Yield 83 mg (37%); colorless crystals; mp 198–199 °C; ^1^H NMR (600 MHz, [D_6_]DMSO): δ = 10.26 (s, 1H; NH), 8.03 (d, *J* = 8.3 Hz, 1H; 5-H), 7.73 (d, *J* = 8.3 Hz, 1H; 4-H), 2.73 (s, 3H; 2-CH_3_), 1.72 (s, 3H; 3-CH_3_); ^13^C{^1^H} NMR (150 MHz, [D_6_]DMSO): δ = 155.4 (q, *J* = 37.5 Hz; O=C), 141.1 (C4a), 135.7 (C5), 133.5 (C7a*), 132.8 (C6*), 125.1 (C7), 123.5 (C4), 115.2 (q, *J* = 289.5 Hz; CF_3_), 72.1 (C3), 24.5 (3-CH_3_), 22.9 (2-CH_3_); IR (KBr): ν̃ = 3354 (w; NH), 1727 (m; C=O), 1550 (m, O=C-NH), 1304 (m; SO_2_), 1167 (m; SO_2_); HRMS (ESI) *m*/*z*: [M + H]^+^ Calcd for C_11_H_10_Cl_2_F_3_N_2_O_3_S 376.9736; Found 376.9735.

#### *N*-(6,7-Dichloro-2,3-dimethyl-1,1-dioxo-2,3-dihydro-1,2-benzisothiazol-3-yl)difluoroacetamide (**2h**)

To the solution of **1h** (108 mg) in THF (1.5 mL) was added *t*-BuOK (0.60 mmol, 67 mg) at 25 °C and stirred for 30 min. Then, it was quenched with water (5 mL), and THF was evaporated. After stirring and cooling with ice water bath, the precipitated byproduct was filtered. The filtrate was extracted with DCM (3 × 15 mL), the combined organic layer was dried over MgSO_4_ and evaporated. Yield 71 mg (67%); colorless crystals; mp 90–91 °C; ^1^H NMR (600 MHz, [D_6_]DMSO): δ = 9.67 (s, 1H; NH), 8.02 (d, *J* = 8.3 Hz, 1H; 5-H), 7.64 (d, *J* = 8.3 Hz, 1H; 4-H), 6.19 (t, *J* = 53.5 Hz, 1H; CHF_2_), 2.77 (s, 3H; 2-CH_3_), 1.67 (s, 3H; 3-CH_3_); ^13^C{^1^H} NMR (150 MHz, [D_6_]DMSO): δ = 161.4 (t, *J* = 26.0 Hz; O=C), 141.8 (C3a), 135.4 (C5), 133.2 (C7a*), 132.8 (C6), 124.9 (C7), 123.5 (C4), 107.9 (t, *J* = 247.3 Hz; CHF_2_), 71.6 (C3), 24.7 (3-CH_3_), 22.8 (2-CH_3_); IR (KBr): ν̃ = 3330 (w; NH), 1714 (m; C=O), 1548 (w, O=C-NH), 1303 (m; SO_2_), 1162 (m; SO_2_); HRMS (ESI) *m*/*z*: [M + H]^+^ Calcd for C_11_H_11_Cl_2_F_2_N_2_O_3_S 358.9830; Found 358.9830.

#### 6,7-Dichloro-2,3-dimethyl-3-(methylamino)-2,3-dihydro-1,2-benzisothiazole 1,1-Dioxide (**2i**)

Using **1i** (177 mg). Yield 148 mg (84%); colorless crystals; mp 187–189 °C (EtOH); ^1^H NMR (600 MHz, [D_6_]DMSO): δ = 8.03 (d, *J* = 8.3 Hz, 1H; 5-H), 7.62 (d, *J* = 8.3 Hz, 1H; 4-H), 3.64 (q, *J* = 5.7 Hz, 1H; NH), 2.68 (s, 3H; 2-CH_3_), 1.77 (d, *J* = 5.7 Hz, 3H; HN-*CH*_*3*_), 1.46 (s, 3H; 3-CH_3_); ^13^C{^1^H} NMR (150 MHz, [D_6_]DMSO): δ = 142.9 (C3a), 135.4 (C5), 134.4 (C7a*), 133.3 (C6*), 124.9 (C6), 124.5 (C4), 79.1 (C3), 28.1 (HN–CH_3_), 25.3 (3-CH_3_), 21.5 (2-CH_3_); IR (KBr): ν̃ = 3350 (m; NH), 1287 (vs; SO_2_), 1160 (vs; SO_2_); HRMS (ESI) *m*/*z*: [M + H]^+^ Calcd for C_10_H_13_Cl_2_N_2_O_2_S 295.0069; Found 295.0068.

#### 6,7-Dichloro-3-(ethylamino)-2,3-dimethyl-2,3-dihydro-1,2-benzisothiazole 1,1-Dioxide (**2j**)

Using **1j** (93 mg). Yield 74 mg (80%); colorless crystals; mp 112–113 °C; ^1^H NMR (600 MHz, [D_6_]DMSO): δ = 8.02 (d, *J* = 8.2 Hz, 1H; 5-H), 7.64 (d, *J* = 8.2 Hz, 1H; 4-H), 3.58 (q, *J* = 4.2 Hz, 1H; NH), 2.69 (s, 3H; 2-CH_3_), 2.25–2.15 (m, 1H; N–CH), 1.83–1.72 (m, 1H; N–CH), 1.47 (s, 3H; 3-CH_3_), 0.91 (t, *J* = 7.1 Hz, 3H; CH_2_–*CH*_*3*_); ^13^C{^1^H} NMR (150 MHz, [D_6_]DMSO): δ = 143.5 (C3a), 135.4 (C5), 134.2 (C7a*), 133.2 (C6*), 124.9 (C7), 124.4 (C4), 78.6 (C3), 36.2 (N–CH_2_), 25.4 (3-CH_3_), 21.6 (2-CH_3_), 15.0 (CH_2_–*CH*_*3*_); IR (KBr): ν̃ = 3348 (m; NH), 1291 (s; SO_2_), 1156 (s; SO_2_); HRMS (ESI) *m*/*z*: [M + H]^+^ Calcd for C_11_H_15_Cl_2_N_2_O_2_S 309.0226; Found 309.0227.

#### *N*-(6-Chloro-2,3-dimethyl-1,1-dioxo-2,3-dihydro-1,2-benzisothiazol-3-yl)acetamide (2k)

To the solution of **1k** (87 mg, 0.30 mmol) in THF (1.5 mL) was added *t*-BuOK (0.60 mmol, 67 mg) at 25 °C and stirred for 30 min. Then, it was quenched with water (5 mL), and THF was evaporated. Then, the aqueous mixture was refluxed for 1 h. After stirring and cooling with ice water bath for 1 h, the precipitated product was filtered and washed with water. Yield 56 mg (65%); colorless crystals; mp 277–278 °C; ^1^H NMR (600 MHz, [D_6_]DMSO): δ = 8.71 (s, 1H; NH), 8.05 (d, *J* = 1.8 Hz, 1H; 7-H), 7.73 (dd, *J* = 8.5, 1.8 Hz, 1H; 5-H), 7.57 (d, *J* = 8.5 Hz, 1H; 4-H), 2.65 (s, 3H; 2-CH_3_), 1.82 (s, 3H; O=C–CH_3_), 1.55 (s, 3H; 3-CH_3_); ^13^C{^1^H} NMR (150 MHz, [D_6_]DMSO): δ = 168.7 (O=C), 140.7 (C3a), 135.1 (C6*), 134.0 (C7a*), 133.4 (C5), 125.1 (C4), 120.8 (C7), 72.6 (C3), 25.2 (3-CH_3_), 23.1 (O=C-*CH*_*3*_), 22.6 (2-CH_3_); IR (KBr): ν̃=, 3265 (m; NH), 1669 (m; O=C), 1298 (m; SO_2_), 1157 (m; SO_2_); HRMS (ESI) *m*/*z*: [M + H]^+^ Calcd for C_11_H_14_ClN_2_O_3_S: 289.0408; Found 289.0408.

### General Procedure for the Synthesis of Compounds **2l**–**o**,**q**

To the mixture of **1l**–**o**, **1q** (0.3 mmol) in THF (1.5 mL) was added *t*-BuOK (0.60 mmol, 67 mg) at 25 °C and stirred for 30 min. Then, it was quenched with aq HCl (1 w/w%, 2.5 mL), and THF was evaporated. Upon cooling with ice water, the precipitated product was filtered and washed with water to give compounds **2l**–**o**,**q**.

#### *N*-(7-Chloro-6-methoxy-2,3-dimethyl-1,1-dioxo-2,3-dihydro-1,2-benzisothiazol-3-yl)acetamide (**2l**)

Using **1l** (96 mg). Yield 89 mg (93%); colorless crystals; mp 251–252 °C (EtOH); ^1^H NMR (600 MHz, [D_6_]DMSO): δ = 8.63 (s, 1H; NH), 7.48 (d, *J* = 8.5 Hz, 1H; 4-H), 7.45 (d, *J* = 8.5 Hz, 1H; 5-H), 3.94 (s, 3H; OCH_3_), 2.65 (s, 3H; 2-CH_3_), 1.81 (s, 3H; O=C–CH_3_), 1.55 (s, 3H; 3-CH_3_); ^13^C{^1^H} NMR (150 MHz, [D_6_]DMSO): δ = 168.8 (O=C), 155.2 (C6), 135.4 (C3a), 132.2 (C7), 122.7 (C4), 117.6 (C5), 114.0 (C7a), 71.5 (C3), 57.3 (OCH_3_), 25.6 (3-CH_3_), 23.2 (O=C-*CH*_*3*_), 22.7 (2-CH_3_); IR (KBr): ν̃ = 3452 (m; NH), 3264 (w; NH), 1649 (m; O=C), 1282 (w; SO_2_), 1151 (s; SO_2_); HRMS (ESI) *m*/*z*: [M + NH_4_]^+^ Calcd for C_12_H_19_ClN_3_O_4_S 336.0779; Found 336.0784.

#### *N*-(7-Chloro-2,3-dimethyl-1,1-dioxo-2,3-dihydro-1,2-benzisothiazol-3-yl)acetamide (**2m**)

Using **1m** (87 mg). Yield 72 mg (83%); colorless crystals; mp 257–258 °C (EtOH); ^1^H NMR (600 MHz, [D_6_]DMSO): δ = 8.73 (s, 1H; NH), 7.68 (t, *J* = 7.9 Hz, 1H; 5-H), 7.63 (d, *J* = 7.9 Hz, 1H; 6-H), 7.51 (d, *J* = 7.9 Hz, 1H; 4-H), 2.66 (s, 3H; 2-CH_3_), 1.82 (s, 3H; O=C–CH_3_), 1.55 (s, 3H; 3-CH_3_); ^13^C{^1^H} NMR (150 MHz, [D_6_]DMSO): δ = 168.7 (O=C), 144.7 (C3a), 134.8 (C5), 131.0 (C7a), 130.0 (C6), 126.4 (C7), 122.0 (C4), 71.9 (C3), 25.4 (3-CH_3_), 23.0 (O=C-*CH*_*3*_), 22.5 (2-CH_3_); IR (KBr): ν̃ = 3267 (m; NH), 1671 (s; O=C), 1294 (vs; SO_2_), 1158 (s; SO_2_); HRMS (ESI) *m*/*z*: [M + H]^+^ Calcd for C_11_H_14_ClN_2_O_3_S 289.0408; Found 289.0408.

#### *N*-(7-Methoxy-2,3-dimethyl-1,1-dioxo-2,3-dihydro-1,2-benzisothiazol-3-yl)acetamide (**2n**)

Using **1n** (85 mg). Yield 44 mg (52%); colorless crystals; mp 189–190 °C (EtOH); ^1^H NMR (600 MHz, [D_6_]DMSO): δ = 8.58 (s, 1H; NH), 7.60 (t, *J* = 8.1 Hz, 1H; 5-H), 7.14 (d, *J* = 8.1 Hz, 1H; 6-H), 7.04 (d, *J* = 7.9 Hz, 1H; 4-H), 3.93 (s, 3H; OCH_3_), 2.60 (s, 3H; 2-CH_3_), 1.81 (s, 3H; O=C–CH_3_), 1.51 (s, 3H; 3-CH_3_); ^13^C{^1^H} NMR (150 MHz, [D_6_]DMSO): δ = 168.5 (O=C), 153.9 (C7), 144.2 (C3a), 135.1 (C5), 120.9 (C7a), 114.4 (C4), 111.5 (C6), 72.1 (C3), 56.3 (OCH_3_), 25.5 (3-CH_3_), 23.2 (O=C-*CH*_*3*_), 22.5 (2-CH_3_); IR (KBr): ν̃=, 3306 (w; NH), 1673 (m; O=C), 1291 (s; SO_2_), 1155 (m; SO_2_); HRMS (ESI) *m*/*z*: [M + H]^+^ Calcd for C_12_H_17_N_2_O_4_S 285.0904; Found 285.0903.

#### *N*-(2,3-Dimethyl-1,1-dioxo-2,3-dihydro-1,2-benzisothiazol-3-yl)acetamide (**2o**)

Using **1o** (76 mg). Yield 57 mg (75%); colorless crystals; mp 193–194 °C; ^1^H NMR (600 MHz, [D_6_]DMSO): δ = 8.64 (s, 1H; NH), 7.82 (d, *J* = 7.7 Hz, 1H; 7-H), 7.68 (t, *J* = 7.7 Hz, 1H; 5-H), 7.58 (t, *J* = 7.7 Hz, 1H; 6-H), 7.54 (d, *J* = 7.7 Hz, 1H; 4-H), 2.65 (s, 3H; 2-CH_3_), 1.82 (s, 3H; O=C–CH_3_), 1.56 (s, 3H; 3-CH_3_); ^13^C{^1^H} NMR (150 MHz, [D_6_]DMSO): δ = 168.6 (O=C), 141.8 (C3a), 133.4 (C7a), 133.2 (C7), 129.4 (C4), 123.1 (C6), 120.7 (C5), 72.9 (C3), 25.4 (3-CH_3_), 23.2 (O=C–CH_3_), 22.5 (2-CH_3_); IR (KBr): ν̃ = 3370 (w; NH), 1670 (m; O=C), 1284 (m; SO_2_), 1159 (m; SO_2_); HRMS (ESI) *m*/*z*: [M + H]^+^ Calcd for C_11_H_15_N_2_O_3_S 255.0797; Found 255.0799.

#### *N*-(6,7-Dichloro-3-methyl-2-tosyl-1,1-dioxo-2,3-dihydro-1,2-benzisothiazol-3-yl)acetamide (**2q**)

Using **1q** (139 mg). Yield 132 mg (95%); colorless crystals; mp 199.5–200.5 °C (EtOH); ^1^H NMR (600 MHz, [D_6_]DMSO): δ = 9.30 (s, 1H; NH), 7.98 (d, *J* = 8.5 Hz, 1H; 5-H), 7.88 (d, *J* = 7.9 Hz, 2H; *o*-H), 7.50 (d, *J* = 8.5 Hz, 1H; 4-H), 7.43 (d, *J* = 7.9 Hz, 2H; *m*-H), 2.39 (s, 3H; *p*-CH_3_), 1.95 (s, 3H; 3-CH_3_), 1.21 (s, 3H; O=C–CH_3_); ^13^C{^1^H} NMR (150 MHz, [D_6_]DMSO): δ = 168.6 (O=C), 145.2 (*p*-C), 141.4 (C3a), 136.1 (C5), 135.9 (ipso-C), 133.1 (C6), 131.6, (C7a), 129.7 (2C; *m*-CH), 128.3 (2C; *o*-CH), 124.9 (C7), 122.9 (C4), 75.7 (C3), 29.8 (3-CH_3_), 22.2 (O=C-*CH*_*3*_), 21.3 (*p*-CH_3_); IR (KBr): ν̃ = 3250 (w; NH), 3200 (w; NH), 1672 (m; O=C), 1370 (m; SO_2_), 1196 (m; SO_2_); HRMS (ESI) *m*/*z*: [M + H]^+^ Calcd for C_17_H_17_Cl_2_N_2_O_5_S_2_ 462.9950; Found 462.9950.

#### Synthesis of *N*-{1-[3,4-dichloro-2-(*N*-tosylsulfamoyl)phenyl]vinyl}acetamide (**5q**)

To the suspension of **1q** (0.60 mmol) in THF (3 mL) was added *t*-BuOK (1.2 mmol, 135 mg) at 25 °C and stirred for 30 min. Then, it was quenched with water (10 mL), and THF was evaporated. After stirring and cooling with ice water bath for 1 h, the precipitated product was filtered and washed with water. Yield 80 mg (29%); colorless crystals, mp 264–265 °C (EtOH); ^1^H NMR (600 MHz, [D_6_]DMSO): δ = 9.11 (s, 1H; NH), 7.67 (d, *J* = 8.3 Hz, 1H), 7.48 (d, *J* = 7.9 Hz, 2H), 7.26–7.08 (m, 3H), 5.75 (s, 1H; = CH), 4.44 (s, 1H; = CH), 2.33 (s, 3H), 1.88 (s, 3H), (sulfonimide NH is under the noise level); ^13^C{^1^H} NMR (150 MHz, [D_6_]DMSO): δ = 167.8, 143.4, 142.5, 142.4, 140.7, 138.3, 132.3, 131.7, 131.0, 128.7 (2C), 125.8 (2C), 101.0 (=CH_2_), 24.2, 21.1; IR (KBr): ν̃ = 3328 (m; NH), 1668 (s; O=C), 1526 (s; O=C-NH), 1311 (s; SO_2_), 1089 (vs; SO_2_); HRMS (ESI) *m*/*z*: [M + H]^+^ Calcd for C_17_H_17_Cl_2_N_2_O_5_S_2_ 462.9950; Found 462.9959.

### Synthesis of 1,2-Benzothiazine 1,1-Dioxides (**3**)

#### *N*-(7,8-Dichloro-2-methyl-1,1-dioxo-3,4-dihydro-2*H*-1,2-benzothiazin-4-yl)acetamide Monohydrate (**3a**·**H**_**2**_**O**)

To the solution of **1a** (0.60 mmol, 194 mg) in DMSO (3 mL) was added *t*-BuOK (3.6 mmol, 404 mg) at 25 °C and stirred for 30 min. Then, it was quenched with water (15 mL), and after intensive stirring for 1 h, the precipitated product was filtered and washed with water to give **3a·H**_**2**_**O** (163 mg, 80%); colorless crystals; mp 186–187 °C (EtOH); ^1^H NMR (600 MHz, [D_6_]DMSO): δ = 8.53 (d, *J* = 8.5 Hz, 1H; NH), 7.90 (d, *J* = 8.5 Hz, 1H; 6-H), 7.38 (dd, *J* = 8.7, 0.7 Hz, 1H; 5-H), 5.38 (ddd, *J* = 10.2, 8.5, 5.4 Hz, 1H; 4-H), 3.88 (dd, *J* = 14.7, 10.2 Hz, 1H; 3-H_ax_), 3.66 (dd, *J* = 14.7, 5.4 Hz, 1H; 3-H_eq_), 2.98 (s, 3H; 2-CH_3_), 1.92 (s, 3H; O=C–CH_3_); ^13^C{^1^H} NMR (150 MHz, [D_6_]DMSO): δ = 170.0 (O=C), 139.3 (C4a), 135.7 (C8a), 133.7 (C6), 133.0 (C7), 129.8 (C5), 128.4 (C8), 50.5 (C3), 40.7 (C4), 36.2 (2-CH_3_), 22.9 (O=C-*CH*_*3*_); IR (KBr): ν̃ = 3451 (m; NH), 3258 (m; NH), 1644 (vs; O=C), 1329 (vs; SO_2_), 1172 (s; SO_2_); HRMS (ESI) *m*/*z*: [M + H]^+^ Calcd for C_11_H_13_Cl_2_N_2_O_3_S 323.0018; Found 323.0017; Elemental analysis Calcd (%) for C_11_H_12_Cl_2_N_2_O_3_S·H_2_O C 38.72, H 4.14, Cl 20.78, N 8.21, S 9.40; Found C 38.53, H 4.20, Cl 21.27, N 8.14, S 9.55. Scaled-up experiment: To the solution of **1a** (3.00 mmol, 970 mg) in DMSO (15 mL) was added *t*-BuOK (18.0 mmol, 2020 mg) at 25 °C and stirred for 30 min. Then, it was quenched with water (75 mL), the precipitated product was filtered and washed with water to give **3a·H**_**2**_**O** (844 mg, 82%).

#### *N*-(7,8-Dichloro-2,3-dimethyl-1,1-dioxo-3,4-dihydro-2*H*-1,2-benzothiazin-4-yl)acetamide (**3b**)

To the solution of **1b** (0.60 mmol, 202 mg) in DMSO (3 mL) was added *t*-BuOK (3.6 mmol, 404 mg) at 25 °C and stirred for 30 min. Then, it was quenched with water (15 mL), and refluxed at 98 °C for 2 h. After cooling to room temperature and intensive stirring, the product precipitated, then it was filtered and washed with water. Yield 97 mg (48%); trans–cis mixture, d.r. 97:3; gray crystals. Another reaction with sequential crystallization gave an analytical sample of pure trans racemate; colorless crystals; mp 217–218 °C; ^1^H NMR (600 MHz, [D_6_]DMSO): δ = 8.53 (d, *J* = 8.9 Hz, 1H; NH), 7.88 (d, *J* = 8.7 Hz, 1H; 6-H), 7.33 (d, *J* = 8.7 Hz, 1H; 5-H), 5.15 (t, *J* = 10.0 Hz, 1H; 4-H_ax_), 4.29 (dq, *J* = 10.0, 6.7 Hz, 1H; 3-H_ax_), 2.80 (s, 3H; 2-CH_3_), 1.94 (s, 3H; O=C–CH_3_); 1.29 (t, *J* = 6.7 Hz, 3H; 3-CH_3_); DEPTQ (150 MHz, [D_6_]DMSO): δ = 170.3 (O=C), 139.7 (C4a), 135.2 (C8a), 133.8 (C6), 132.9 (C7), 129.6 (C5), 128.3 (C8), 54.6 (C3), 46.1 (C4), 29.8 (2-CH_3_), 22.9 (O=C-*CH*_*3*_), 15.8 (3-CH_3_); IR (KBr): ν̃ 3249 (w; NH), 1661 (m; O=C), 1553 (m; O=C-NH), 1344 (m; SO_2_), 1153 (m; SO_2_); HRMS (ESI) *m*/*z*: [M + H]^+^ Calcd for C_12_H_15_Cl_2_N_2_O_3_S 337.0175; Found 337.0176.

#### *N*-(7,8-Dichloro-2-ethyl-1,1-dioxo-3,4-dihydro-2*H*-1,2-benzothiazin-4-yl)acetamide monohydrate (**3c**)

To the solution of **1c** (0.30 mmol, 101 mg) in DMSO (1.5 mL) was added *t*-BuOK (1.8 mmol, 202 mg) at 25 °C and stirred for 30 min. Then, it was quenched with water (7.5 mL), and after intensive stirring for 1 h, the precipitated product was filtered and washed with water to give **3c** (61 mg, 57%); colorless crystals; mp 85–86 °C; ^1^H NMR (600 MHz, [D_6_]DMSO): δ = 8.54 (d, *J* = 8.5 Hz, 1H; NH), 7.89 (d, *J* = 8.6 Hz, 1H; 6-H), 7.38 (d, *J* = 8.6 Hz, 1H; 5-H), 5.29 (ddd, *J* = 9.9, 8.5, 5.4 Hz, 1H; 4-H), 3.83 (dd, *J* = 14.8, 9.9 Hz, 1H; 3-H_ax_), 3.70 (dd, *J* = 14.8, 5.4 Hz, 1H; 3-H_eq_), 3.45 (dq, *J* = 13.7, 7.1 Hz, 1H; *CH*-CH_3_), 3.26 (dq, *J* = 13.7, 7.1 Hz, 1H; *CH*-CH_3_), 1.91 (s, 3H; O=C–CH_3_), 1.19 (t, *J* = 7.1 Hz, 1H; CH_2_–*CH*_*3*_); ^13^C{^1^H} NMR (150 MHz, [D_6_]DMSO): δ = 169.9 (O=C), 139.3 (C4a), 136.8 (C8a), 133.6 (C6), 133.0 (C7), 129.7 (C5), 128.1 (C8), 47.4 (C3), 43.2 (2-CH_2_), 41.7 (C4), 22.8 (O=C-*CH*_*3*_), 14.4 (CH_2_–*CH*_*3*_); IR (KBr): ν̃ = 3457 (m; NH), 3265 (m; NH), 1646 (vs; O=C), 1318 (s; SO_2_), 1169 (m; SO_2_); HRMS (ESI) *m*/*z*: [M + H]^+^ Calcd for C_12_H_15_Cl_2_N_2_O_3_S: 337.0175; Found 337.0175; Elemental analysis Calcd (%) for C_12_H_14_Cl_2_N_2_O_3_S·H_2_O C 40.57, H 4.54, Cl 19.96, N 7.89, S 9.03; Found C 40.58, H 4.51, Cl 20.67, N 7.89, S 9.12.

#### *N*-(2-Benzyl-7,8-dichloro-1,1-dioxo-3,4-dihydro-2*H*-1,2-benzothiazin-4-yl)acetamide (**3d**)

To the suspension of **1d** (0.45 mmol) in THF (2.25 mL) was added *t*-BuOK (2.7 mmol, 303 mg) at 25 °C and stirred for 30 min. Then, it was quenched with water (7.5 mL), and THF was evaporated. After stirring and cooling with ice water bath, then precipitate was filtered as a mixture of **2d** and **3d** (0.25:1, 110 mg, 62%). Upon dissolving in DMSO (4 mL), MeOH (20 mL) and water (38 mL) was added, and the precipitated product was filtered off to give pure **3d**. Yield 43 mg (24%); colorless crystals; mp 178–180 °C; ^1^H NMR (600 MHz, [D_6_]DMSO): δ = 8.52 (d, *J* = 8.3 Hz, 1H; NH), 7.91 (d, *J* = 8.5 Hz, 1H; 6-H), 7.46–7.37 (m, 5H), 7.37–7.29 (m, 1H), 5.33 (ddd, *J* = 9.9, 8.5, 5.4 Hz, 1H; 4-H), 4.61 (d, *J* = 14.7 Hz, 1H; 2-CH), 4.46 (d, *J* = 14.7 Hz, 1H; 2-CH), 3.77 (dd, *J* = 14.9, 9.6 Hz, 1H; 3-H_ax_), 3.47 (dd, *J* = 14.9, 5.2 Hz, 1H; 3-H_eq_), 1.86 (s, 3H; O=C–CH_3_); ^13^C{^1^H} NMR (150 MHz, [D_6_]DMSO): δ = 169.7 (O=C), 139.3 (C4a), 136.9 (C8a), 136.0 (ipso-C), 133.7 (C6), 133.0 (C7), 130.0 (C5), 128.9 (2C; *m*-C), 128.6 (2C; *o*-C), 128.1 (C8), 128.0 (*p*-C), 51.3 (2-CH_2_), 47.3 (C3), 41.9 (C4), 22.8 (O=C-*CH*_*3*_); IR (KBr): ν̃ = 3364 (m; NH), 1683 (s; O=C), 1296 (m; SO_2_), 1153 (m; SO_2_); HRMS (ESI) *m*/*z*: [M + H]^+^ Calcd for C_17_H_17_Cl_2_N_2_O_3_S 399.0331; Found 339.0331.

#### *N*-(7,8-Dichloro-2-methyl-1,1-dioxo-3,4-dihydro-2*H*-1,2-benzothiazin-4-yl)propionamide (**3e**)

To the solution of **1e** (202 mg, 0.60 mmol) in THF (3 mL) was added *t*-BuOK (3.6 mmol, 404 mg) at 25 °C and stirred for 30 min. Then, it was quenched with water (10 mL), and THF was evaporated. After stirring and cooling with ice water bath, precipitate was filtered as a mixture of **2e** and **3e** (160 mg, 79%). Upon dissolving in DMSO (5 mL), MeOH (25 mL) and water (47.5 mL) was added, and the precipitated product was filtered off to give pure **3e**. Yield 80 mg (40%); colorless crystals; mp 200–201 °C; ^1^H NMR (600 MHz, [D_6_]DMSO): δ = 8.45 (d, *J* = 8.6 Hz, 1H; NH), 7.90 (d, *J* = 8.5 Hz, 1H; 6-H), 7.35 (d, *J* = 8.5 Hz, 1H; 5-H), 5.38 (∼ddd, *J* = 10.5, 8.6, 5.5 Hz, 1H; 4-H), 3.90 (dd, *J* = 14.7, 10.5 Hz, 1H; 3-H_ax_), 3.65 (dd, *J* = 14.7, 5.5 Hz, 1H; 3-H_eq_), 2.98 (s, 3H; 2-CH_3_), 2.24–2.15 (m, 2H; O=C–CH_2_), 1.05 (t, *J* = 7.7 Hz, 3H; CH_2_–*CH*_*3*_); ^13^C{^1^H} NMR (150 MHz, [D_6_]DMSO): δ = 173.6 (O=C), 139.4 (C4a), 135.7 (C8a), 133.7 (C6), 133.0 (C7), 129.7 (C5), 128.4 (C8), 50.4 (C3), 40.5 (C4), 36.2 (2-CH_3_), 28.7 (O=C-*CH*_*2*_); 9.9 (CH_2_–*CH*_*3*_); IR (KBr): ν̃ = 3265 (w; NH), 1655 (m; O=C), 1349 (m; SO_2_), 1166 (m; SO_2_); HRMS (ESI) *m*/*z*: [M + H]^+^ Calcd for C_12_H_15_Cl_2_N_2_O_3_S 377.0175; Found 377.0175.

#### *N*-(7,8-Dichloro-2-methyl-1,1-dioxo-3,4-dihydro-2*H*-1,2-benzothiazin-4-yl)butyramide (**3f**)

To the solution of **1f** (211 mg, 0.60 mmol) in THF (3 mL) was added *t*-BuOK (3.6 mmol, 404 mg) at 25 °C and stirred for 30 min. Then, it was quenched with water (10 mL), and THF was evaporated. After stirring and cooling with ice water bath, the precipitate was filtered as a mixture of **2f** and **3f** (182 mg, 86%). Upon dissolving in DMSO (7.4 mL), MeOH (37 mL) and water (70 mL) was added, and the precipitated product was filtered off to give pure **3f**. Yield 149 mg (71%); colorless crystals; mp 205–206 °C; ^1^H NMR (600 MHz, [D_6_]DMSO): δ = 8.48 (d, *J* = 8.7 Hz, 1H; NH), 7.91 (d, *J* = 8.7 Hz, 1H; 6-H), 7.34 (d, *J* = 8.7 Hz, 1H; 5-H), 5.38 (∼ddd, *J* = 10.4, 8.7, 5.5 Hz, 1H; 4-H), 3.90 (dd, *J* = 14.8, 10.4 Hz, 1H; 3-H_ax_), 3.65 (dd, *J* = 14.8, 5.5 Hz, 1H; 3-H_eq_), 2.98 (s, 3H; 2-CH_3_), 2.21–2.08 (m, 2H; O=C–CH_2_), 1.62–1.52 (m, 2H; CH_2_–*CH*_*2*_-CH_3_), 0.89 (t, *J* = 7.4 Hz, 3H; CH_2_–*CH*_*3*_); ^13^C{^1^H} NMR (150 MHz, [D_6_]DMSO): δ = 172.7 (O=C), 139.4 (C4a), 135.7 (C8a), 133.7 (C6), 133.0 (C7), 129.7 (C5), 128.4 (C8), 50.4 (C3), 40.6 (C4), 37.5 (O=C-*CH*_*2*_), 36.2 (2-CH_3_), 18.8 (CH_2_–*CH*_*2*_-CH_3_), 13.8 (CH_2_–*CH*_*3*_); IR (KBr): ν̃ = 3274 (w; NH), 1652 (m; O=C), 1347 (s; SO_2_), 1161 (m; SO_2_); HRMS (ESI) *m*/*z*: [M + H]^+^ Calcd for C_13_H_17_Cl_2_N_2_O_3_S 351.0331; Found 351.0331.

## Supporting Information Available

Additional experimental and theoretical mechanism studies; detailed NMR structure eludication (including ^1^H and ^13^C NMR, DEPTQ, HSQC, HMBC, selective NOE, ROE, and TOCSY spectra) of new compounds; coordinates and energy values of the computed structures; conditions of X-ray diffraction measurements, cif files, and ORTEP diagrams (PDF)
Structure report files of **1a** (CCDC 1995298) (PDF)
Structure report files of **3a**·**H**_**2**_**O** (CCDC 1995299) (PDF)

## Accession Codes

CCDC 1995298–1995299 contain the supplementary crystallographic data for this paper. These data can be obtained free of charge via www.ccdc.cam.ac.uk/data_request/cif, or by emailing data_request@ccdc.cam.ac.uk, or by contacting The Cambridge Crystallographic Data Centre, 12 Union Road, Cambridge CB2 1EZ, UK; fax: +44 1223 336033.
