# Supplementary material for: Basicity-Tuned Reactivity: diaza-[1,2]-Wittig versus diaza-[1,3]-Wittig Rearrangements of 3,4-Dihydro-2H-1,2,3-benzothiadiazine 1,1-Dioxides
Source: J Org Chem. 2020 Dec 31;86(2):1685–700. doi: 10.1021/acs.joc.0c02512 (PMC8021225; doi:10.1021/acs.joc.0c02512)
Supplement: Supplementary file 1 — jo0c02512_si_001.pdf [file jo0c02512_si_001.pdf]

Basicity-Tuned Reactivity: *diaza*-[1,2]-Wittig *versus* *diaza*-[1,3]-Wittig Rearrangements of 3,4-Dihydro-2*H*-1,2,3-benzothiadiazine 1,1-Dioxides

Imre Gyűjtő,[a,b] Márta Porcs-Makkay,[a] Gergő Szabó,[b] Zsolt Kelemen,[b] Gyöngyvér Pusztai,[a] Gábor Tóth,[a] András Dancsó,[a] Judit Halász,[a] Gyula Simig,[a] Balázs Volk,[a,*] László Nyulászi[b,*]

Table of content

Additional tables and figures.......... S2

Detailed NMR structure elucidation of compounds **2a**, **3a**, *trans*-**3b** and *cis*-**3b**, compounds present in *t*-BuOK/[D_6]DMSO reaction mixtures, and structure elucidation of **[D]3a** S13

NMR spectra.......... S29

Calculated total energies and XYZ coordinates.......... S70

Single crystal X-ray diffraction data S109

Additional tables and figures

Table S1. Experimental study of various bases used for the deprotonation of **1a**

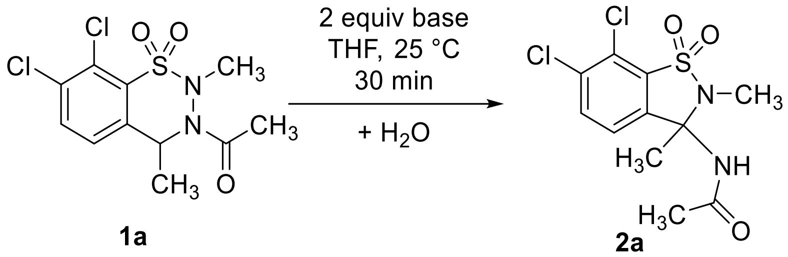

Base	Product	Yield (%)
t-BuOK	**2a**	90
NaOH	**2a**	90
NaOMe	**2a**	76
DBU	**2a**	95
DIPEA	**1a**	95
NaOAc	**1a**	80
EMIM*-OAc	**1a**	91**

Reagents and reaction conditions: **1a** (0.6 mmol), base (2 equiv), THF (3 mL), 25 °C, 30 min, quenching with water, evaporation of THF, crystallization.
* EMIM stands for 1-ethyl-3-methylimidazolium cation.
** With ^{1}H NMR, traces of **2a** could be detected in the crude product.

Table S2. Comparison of the M06-2X/6-31+G* gas-phase acidity of **1a** at position C(4) with that of other weak acids

HB	ΔG_{gas} [kcal/mol]
H_2O	382.7
MeOH	370.3
t-BuOH	365.1
**1a** at C(4) atom	329.9
AcOH	329.2

Gas-phase acidity values in Scheme 3 and Table S2 were obtained as follows: neutral and deprotonated forms of **1a** (at all possible positions), H_2O, MeOH, *t*-BuOH and AcOH were optimized at M06-2X/6-31+G* level of theory. Then free energies were calculated at the same level with fine grid and $G(H^+(gas))=-6.28$ kcal/mol. Deprotonation Gibbs free energies were calculated according to the following equation:

$$\Delta G_{gas} = G(B^-(gas)) + G(H^+(gas)) - G\big(HB(gas)\big)$$

Noteworthy features in the ^{1}H NMR spectrum of **IV^{2-}** depicted in Figure 1 in the manuscript: (i) the partially hindered rotation of the amide moiety results in a characteristic line broadening; (ii) the intensity of the –C(=O)–C<u>H</u>$_3$ signal is only ca. 1.25H (instead of 3H), revealing a H–D exchange with [D$_6$]DMSO under these strongly basic conditions, also supported by LC-MS (ESI) measurements (Figure S1). When a sample of **1a** / 6 equiv *t*-BuOK / DMSO reaction mixture was analyzed with ^{1}H NMR in [D$_6$]DMSO, the –C(=O)–C<u>H</u>$_3$ signal intensity remained about 3H in the ^{1}H NMR spectrum (Figure S2).

Procedure: To a mixture of **1a** (0.6 mmol, 194 mg) dissolved in DMSO (3 mL), *t*-BuOK (3.6 mmol, 404 mg) was added at 25 °C and stirred for 10 min. A sample was analyzed by ^{1}H NMR (Figure S2), which contained **IV^{2-}** dominantly. The residue was quenched with water (10 mL), extracted with DCM (1 × 50, 3 × 25 mL), the combined organic layer was dried over $MgSO_4$ and evaporated to give 159 mg (85%) product identified as a mixture of **2a**, **3a** and **5a** in a ratio of ca. 0.17:1.05:1.00 (Figure S3).

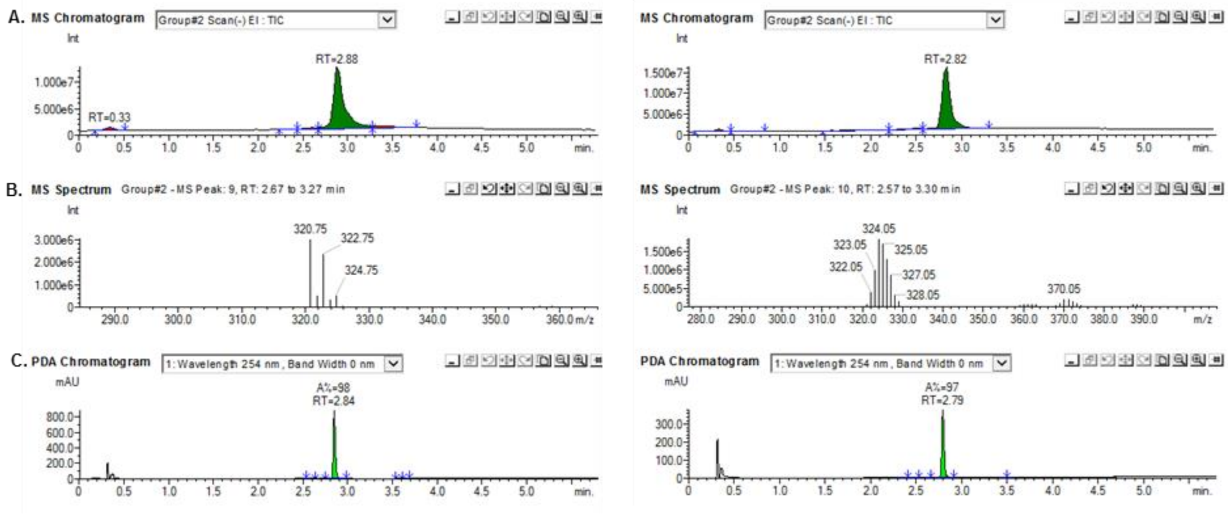

Figure S1. LC-MS analysis of the reaction of **1a** with 6 equiv *t*-BuOK in DMSO (left) or in [D$_6$]DMSO (right)

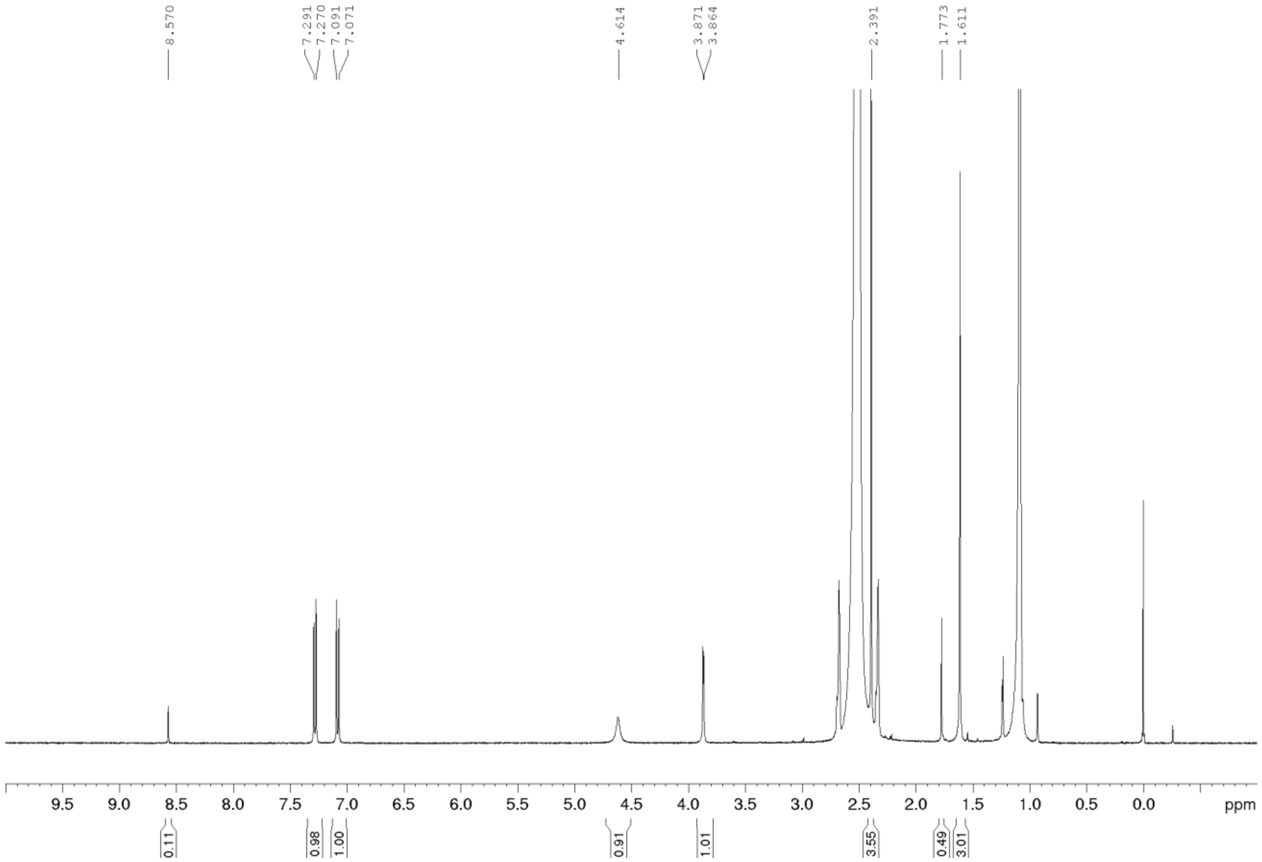

Figure S2. ^{1}H NMR analysis of a sample from **1a** / 6 equiv *t*-BuOK / DMSO reaction mixture, measured in [D$_6$]DMSO

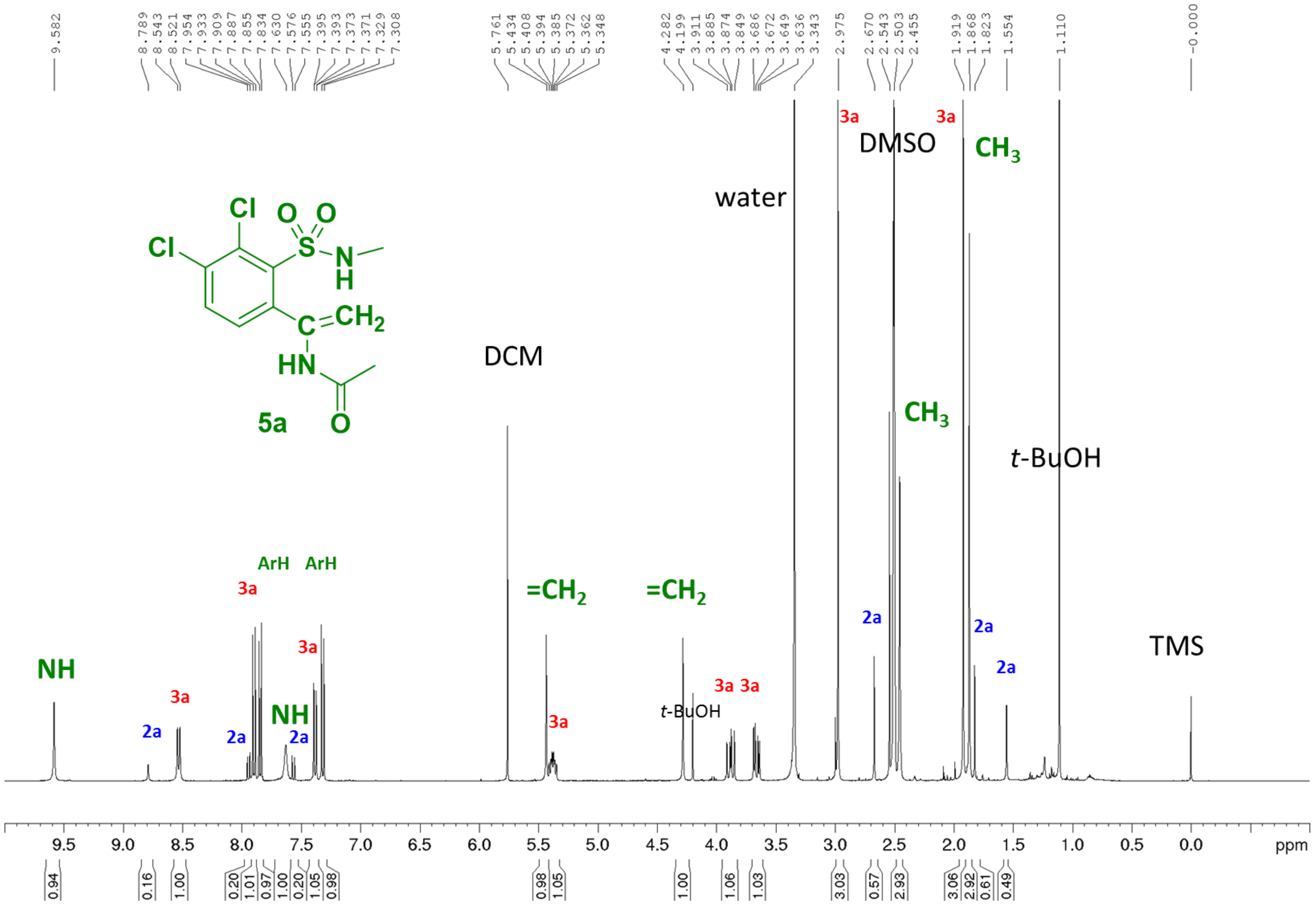

Figure S3. ¹H NMR spectrum of an extracted mixture containing enamide **5a**

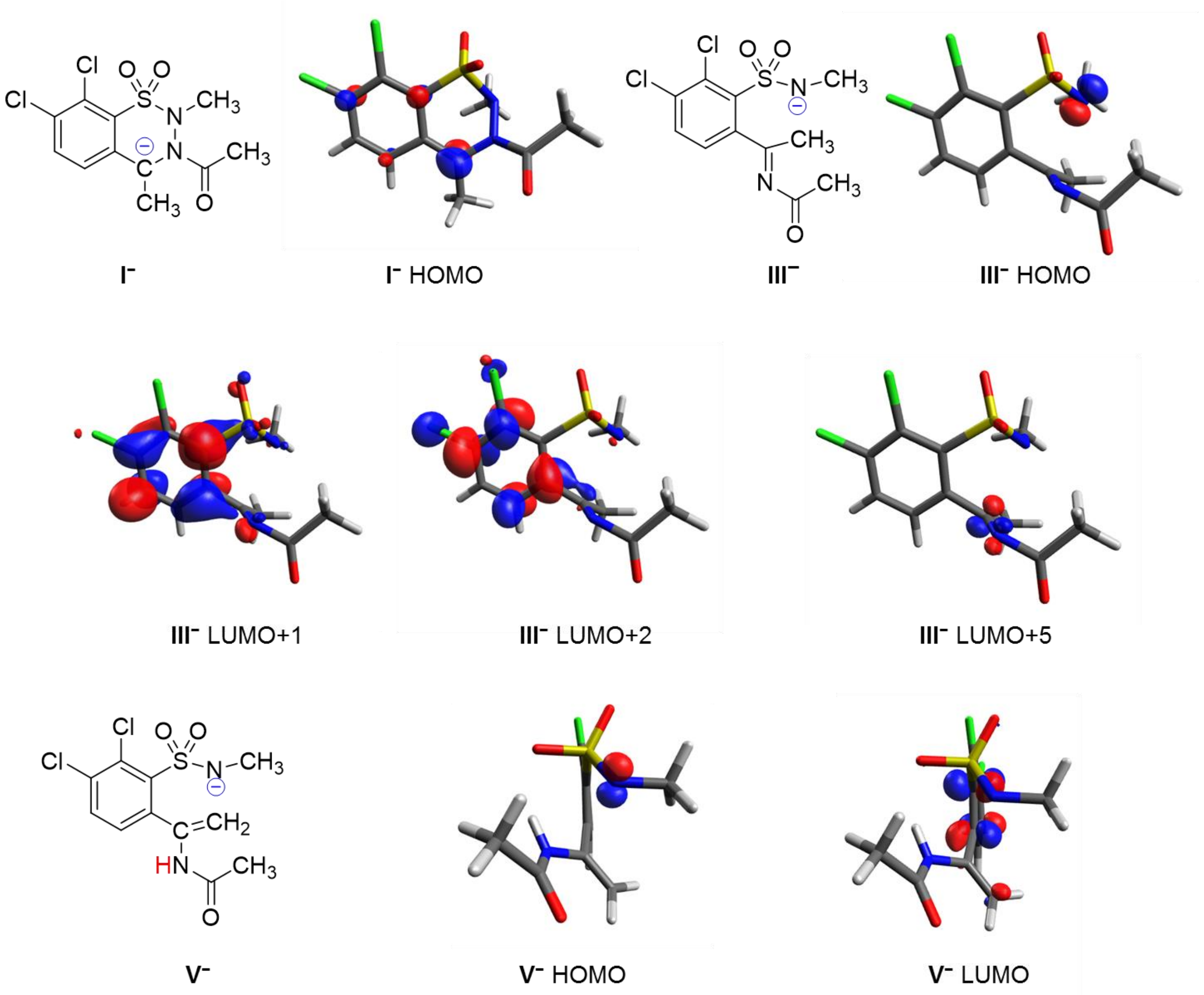

Figure S4. Selected Kohn-Sahm molecular orbitals of **I**⁻, **III** and **V**⁻ at M06-2X/6-31+G* level of theory

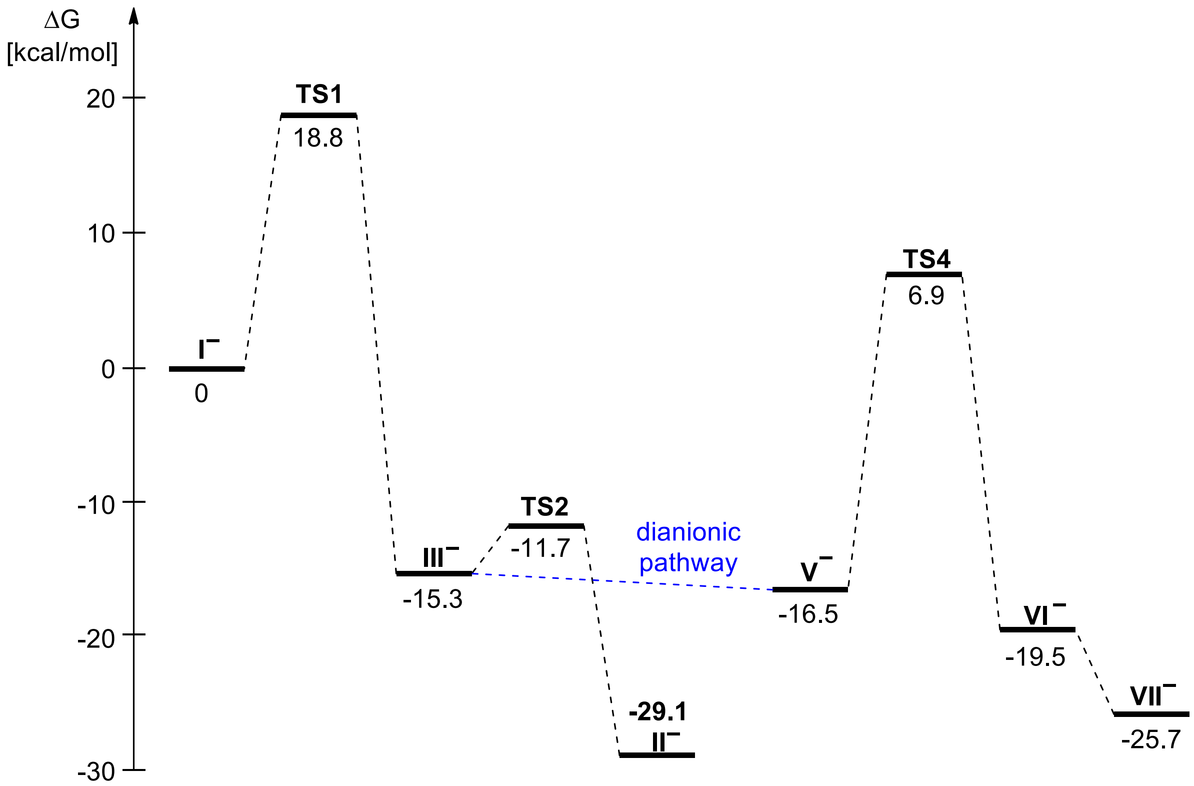

Figure S5. Gibbs free energy profile diagram of the reaction mechanism at M06-2X/6-31+G* (smd: THF) level of theory depicted in Scheme 4

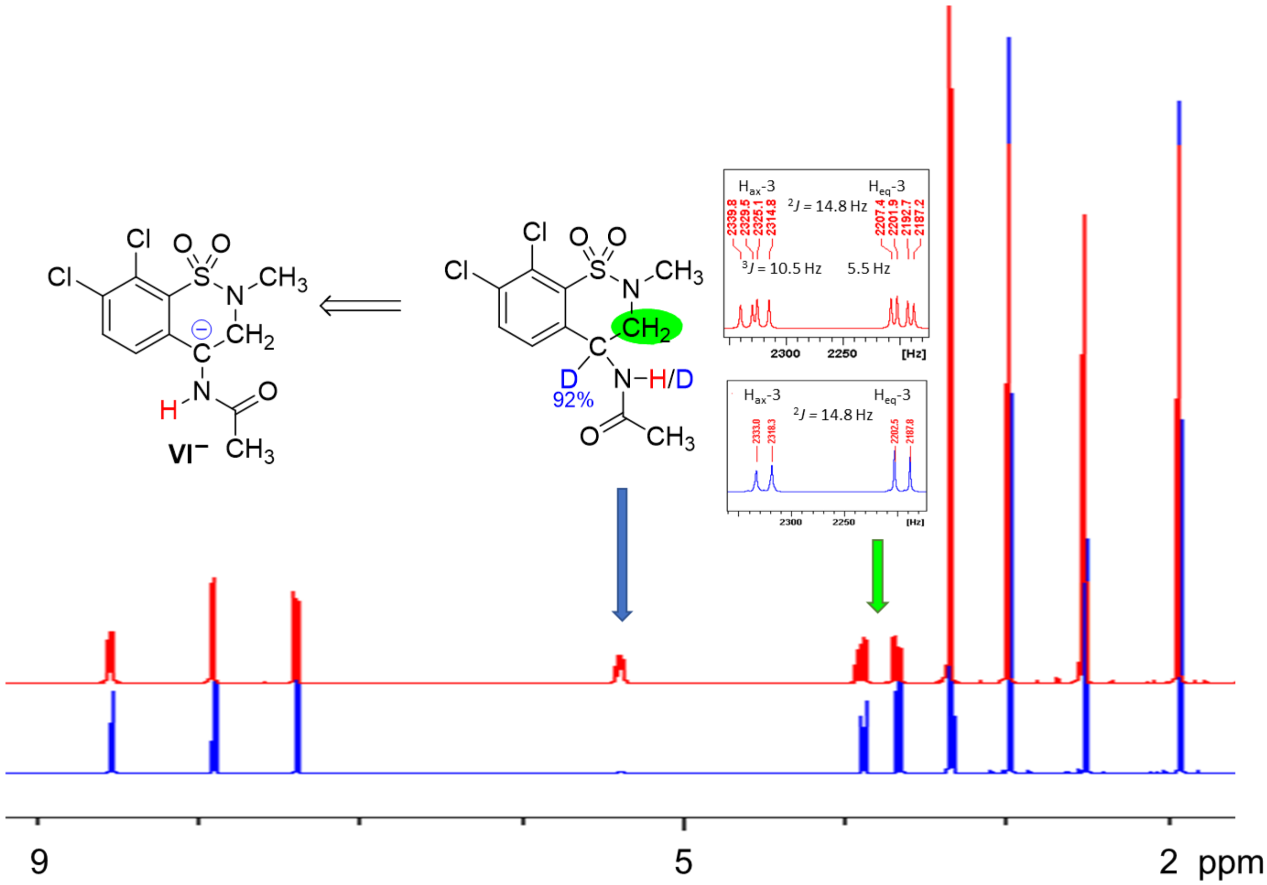

Figure S6. ^{1}H NMR spectra in [D_6]DMSO of **3a** (red) and **[D]3a** (blue) after trapping carbanion **VI⁻** with D_2O

To the solution of **1o** (0.6 mmol, 153 mg) in THF (3 mL) was added *t*-BuOK (1.2 mmol, 2 equiv, 135 mg) at 25 °C and stirred for 30 min. Then it was quenched with water (10 mL), THF was removed in vacuo, and extracted with DCM (2 × 30 mL). The combined organic layer was dried over $MgSO_4$ and evaporated to give 133 mg (87%) product, identified as a mixture of **2o** and the corresponding enamide **5o** in a molar ratio of ca. 0.13:1.00 (Figures S7 and S8).

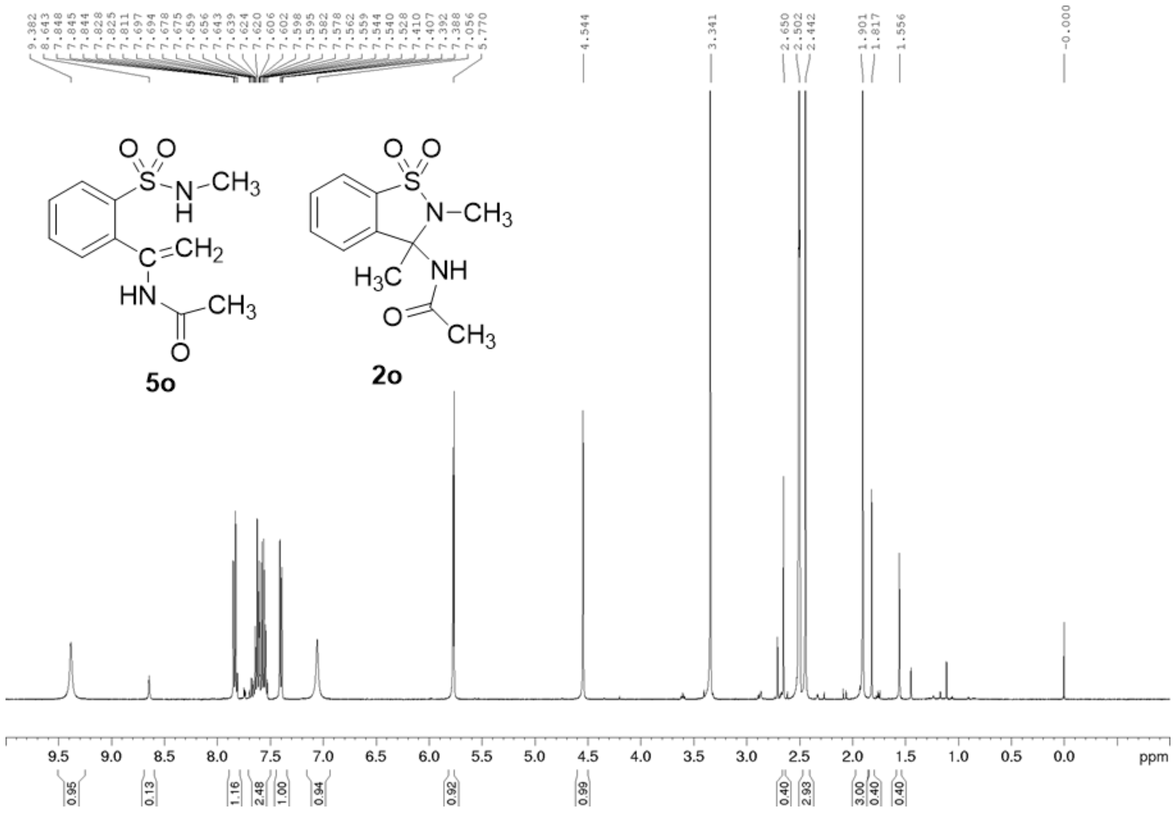

Figure S7. ^{1}H NMR spectrum of **2o** (minor product) besides the corresponding enamide **5o** (major product)

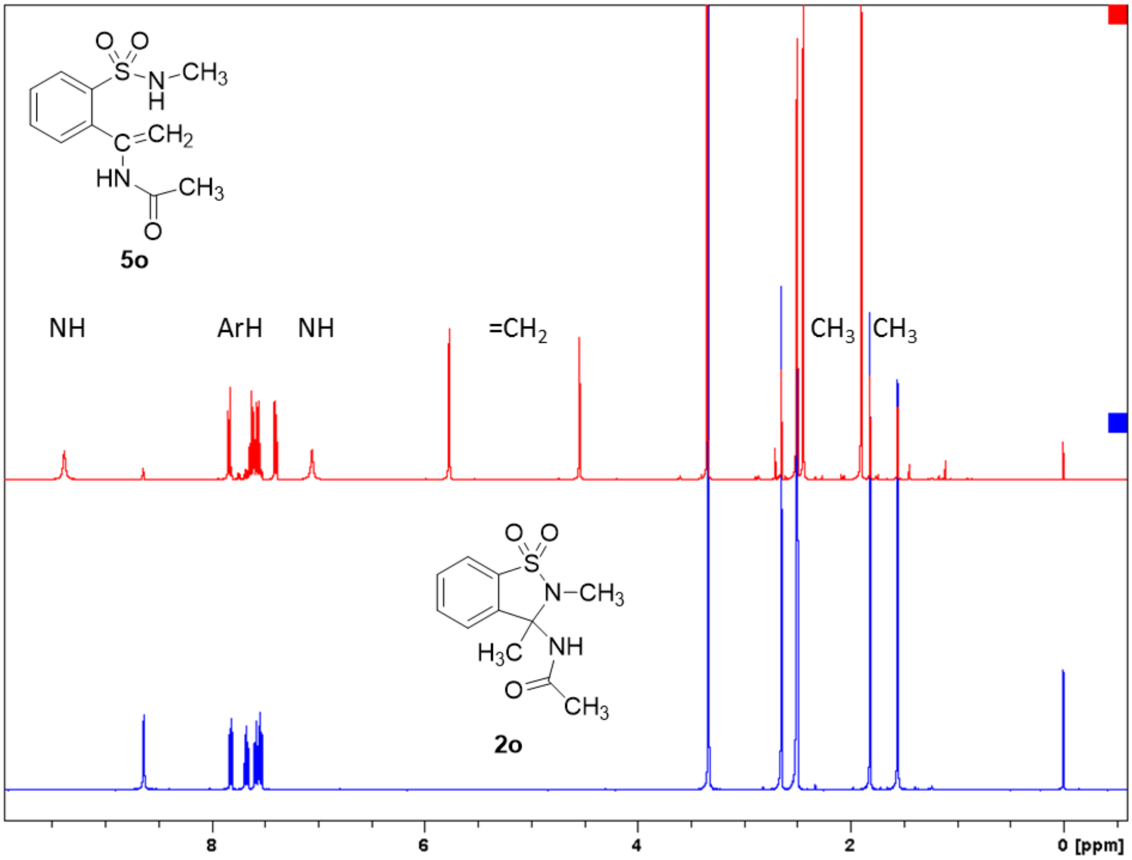

Figure S8. ^{1}H NMR spectrum of **2o** in a mixture with enamide **5o** (above) and in its pure form (below)

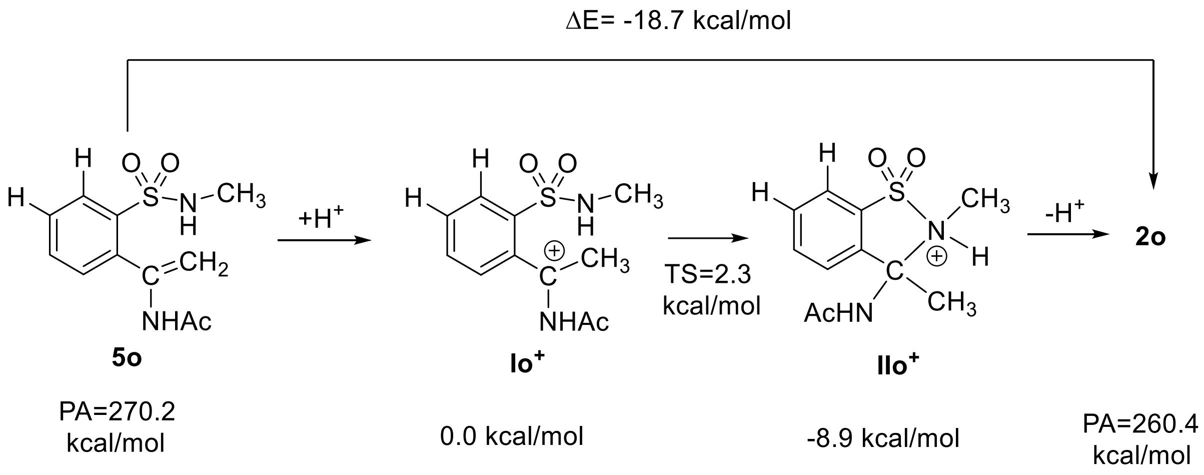

Figure S9. Calculated reaction mechanism of the proton-catalyzed transformation of enamide **5o** to **2o** (at M06-2X/6-31+G* SMD:THF level of theory)

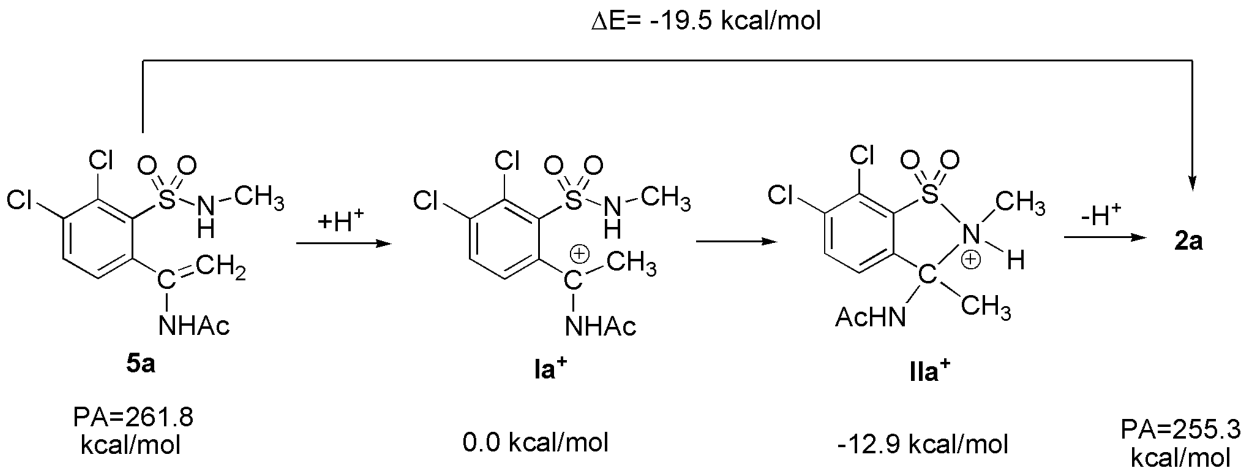

Figure S10. Calculated reaction mechanism of the proton-catalyzed transformation of enamide **5a** to **2a** (at M06-2X/6-31+G* SMD:THF level of theory)

Unfortunately, all attempts to localize the transition state between **Ia**$^+$ and **IIa**$^+$ failed in our hand. In accordance with this, scan calculations (by decreasing the N–C distance stepwise by 0.1 Å) showed that the energy of the system decreased continuously, indicating no significant barrier during this process. In case of **5o**, however, we could localize the corresponding TS and the process exhibited a tiny barrier of 2.3 kcal/mol. Altogether, it is easily understandable that upon protonation the reaction proceeds with a wide range of substituents easily to the thermodynamically stable product **2**.

Experimental evidence to support the mechanism above: acidification of the enamide containing reaction mixture (**IV**$^{2-}$ / **V**$^-$ / **5a**) gave **2a**:

To the solution of **1a** (0.6 mmol, 194 mg) in THF (3 mL) was added *t*-BuOK (3.6 mmol, 404 mg) at 25 °C and stirred for 30 min. After that, THF was evaporated, and the residue was quenched with aq HCl (5 w/w%, 5 mL). Then it was cooled to 5 °C, the precipitated product was filtered and washed with water to give **2a**. Yield: 178 mg (92%).

Table S3. Testing of various methods and levels of theory

**E [kcal/mol]**	optimization (smd: THF)	single point calculation (smd: THF)			
**method level of theory**	M06-2X 6-31+G*	B3LYP 6-31+G*	ωB97X-D 6-31+G*	M06-2X aug-cc-pVTZ	M06-2X 6-311++G**
**I^-**	0.0	0.0	0.0	0.0	0.0
**TS1**	19.6	13.2	19.1	20.0	20.0
**III^-**	-13.2	-18.1	-16.0	-14.9	-13.6
**TS2**	-9.9	-15.8	-12.3	-10.9	-10.0
**II^-**	-29.5	-30.9	-32.6	-29.7	-28.7
**V^-**	-16.4	-18.1	-16.4	-20.1	-18.6
**TS3**	8.2	2.4	7.6	6.7	6.8
**VI^-**	-20.0	-20.8	-20.5	-22.2	-21.5
**VII^-**	-27.6	-28.7	-29.6	-27.7	-26.9
**IV^{2-}**	0.0	0.0	0.0	0.0	0.0
**TS4**	29.8	27.7	31.0	31.6	30.2
**VIII^{2-}**	-2.1	1.8	-0.8	-0.7	-1.9

Optimization was performed at M06-2X/6-31+G* (THF: smd). Single point calculation at the same level employing B3LYP showed moderate differences at **TS1**, whereas ωB97X-D gave similar results. Using larger basis sets (aug-cc-pVTZ or 6-311++G**) afforded only slight changes in the relative energy values, thus we used the 6-31+G* basis.

Direct tautomerization of **III^-** to **V^-** was initially investigated at B3LYP/6-31+G*, and gave a barrier of $\Delta G^{\#}$=79.2 kcal/mol.

Detailed NMR structure elucidation of compounds 2a, 3a, *trans*-3b and *cis*-3b, compounds present in *t*-BuOK/[D6]DMSO reaction mixtures, and structure elucidation of [D]3a

Compound **2a**, ^{1}H and DEPTQ

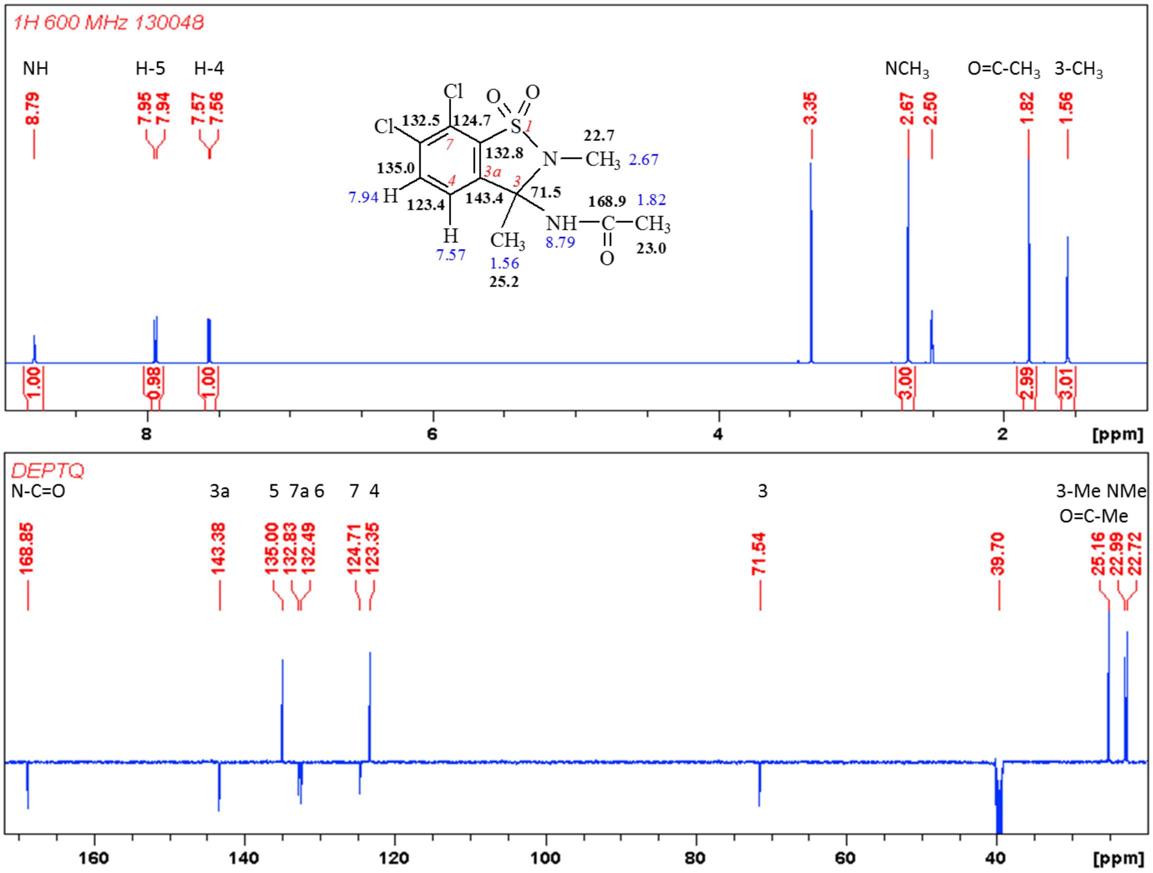

Compound **2a**, HSQC and HMBC

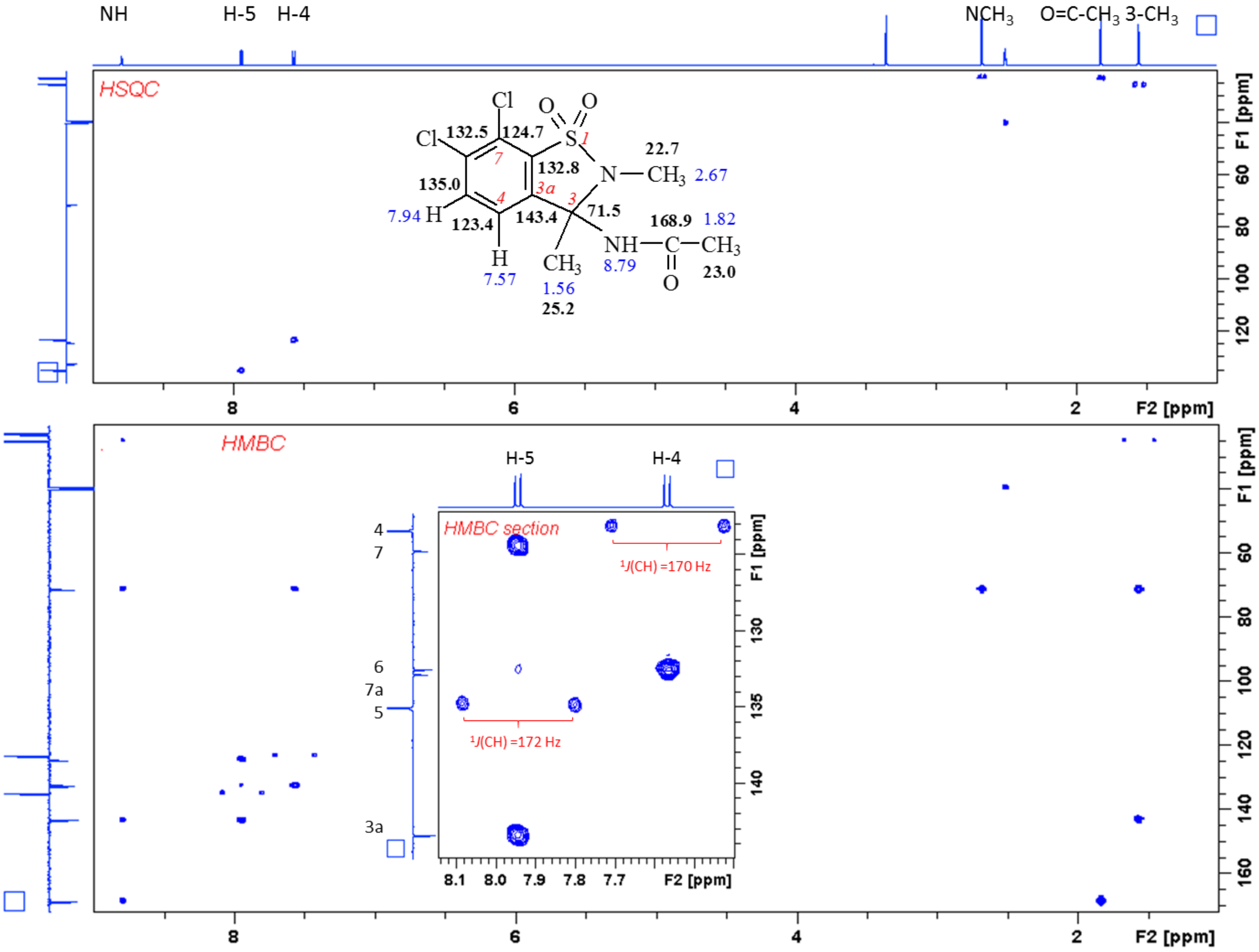

Compound **3a**, ^{1}H and HSQC

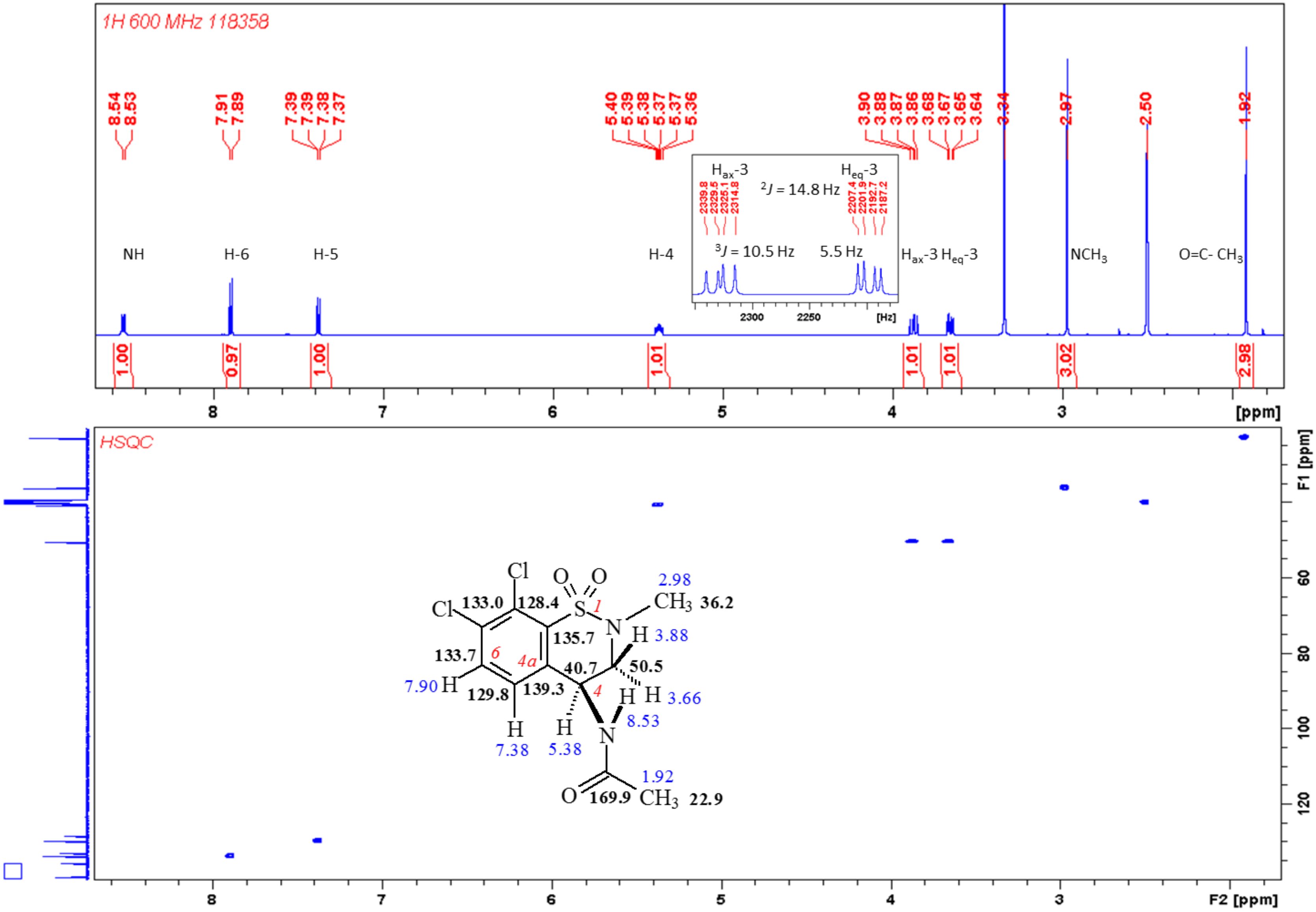

Compound **3a**, ^{13}C and HMBC

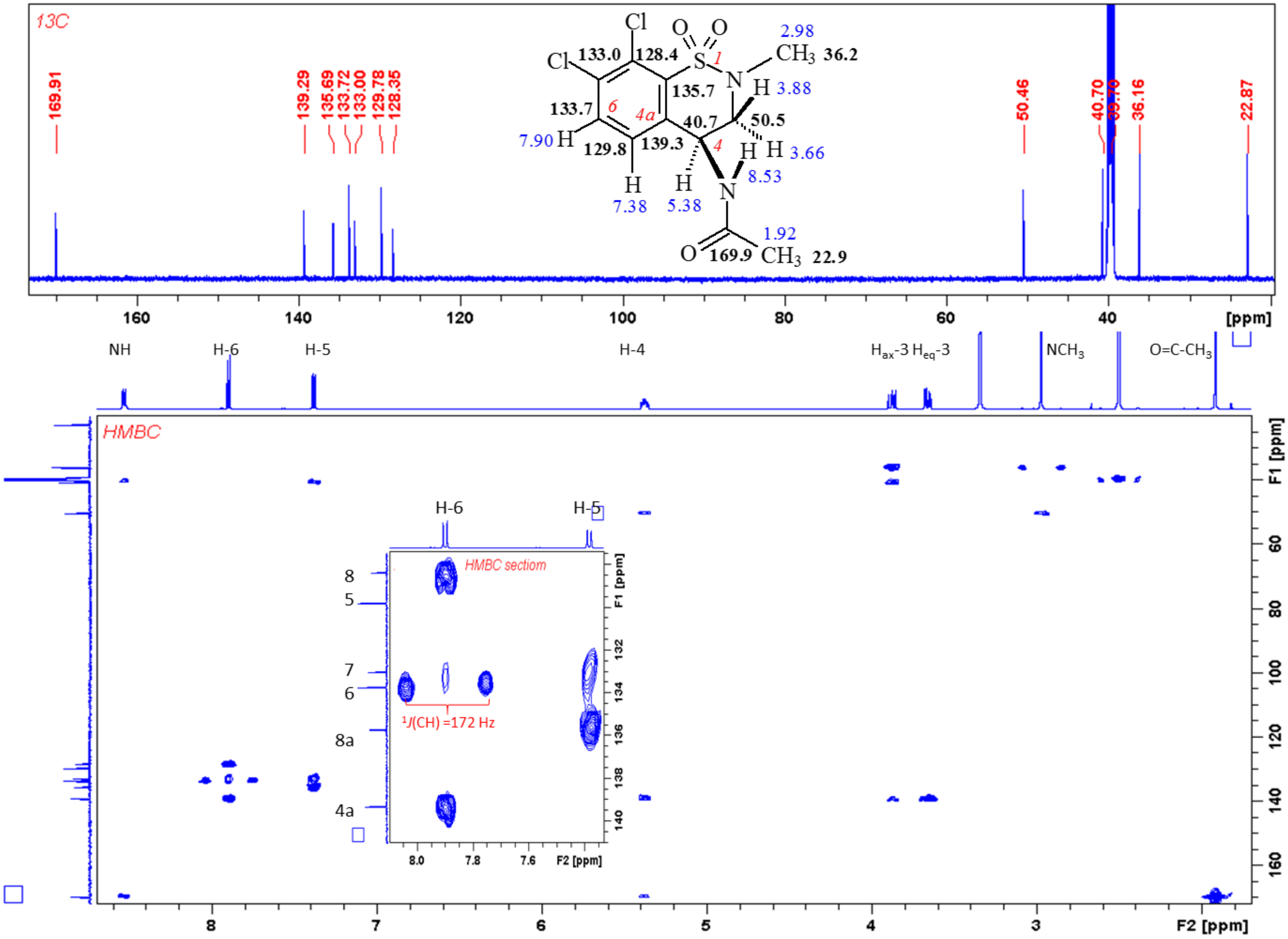

Compound ***trans*-3b**, ^{1}H and edHSQC

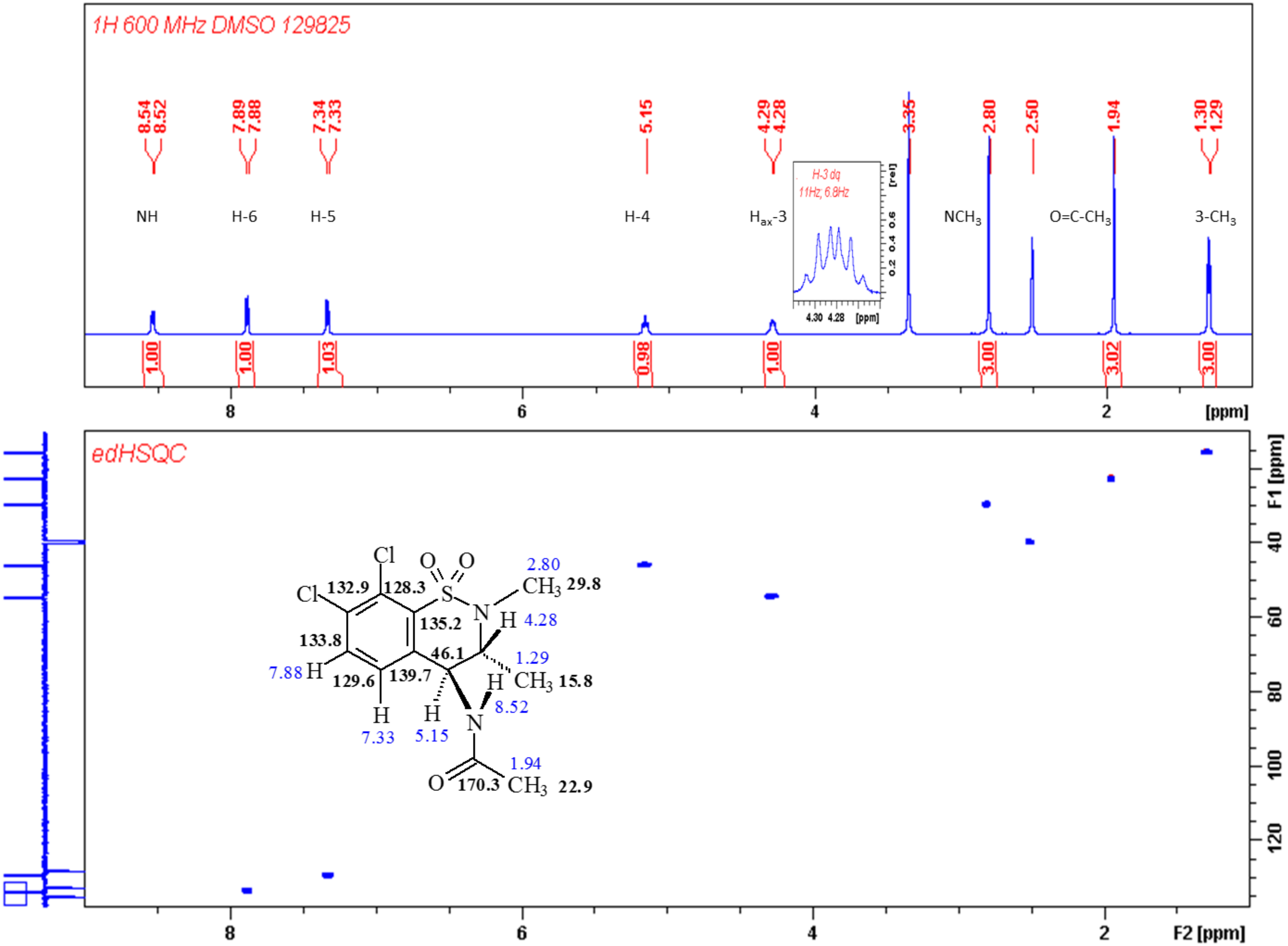

Compound ***trans*-3b**, DEPTQ and HMBC

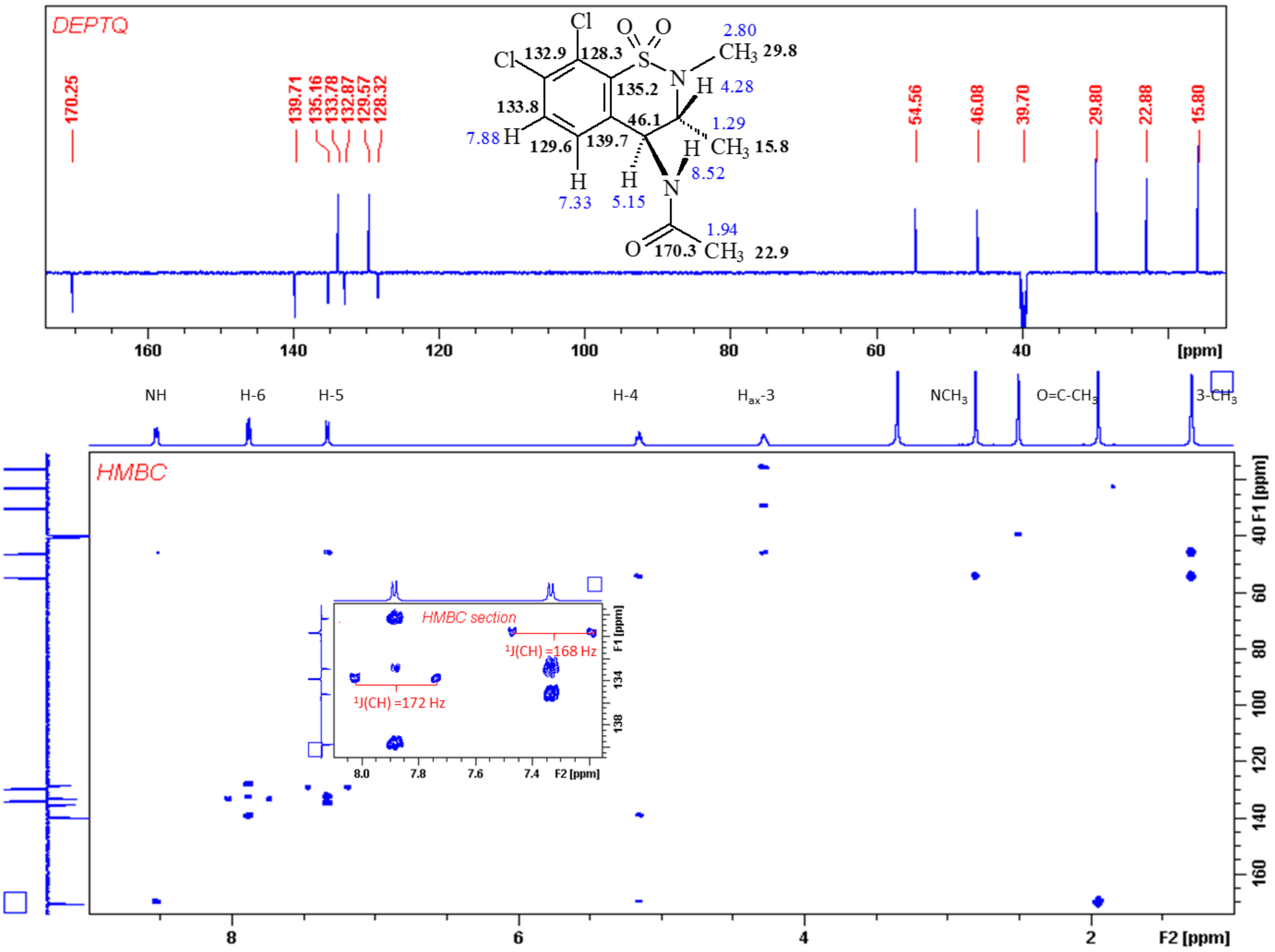

Compound ***trans*-3b** and stereostructure supported by ^{1}H, selNOE on H-4, NCH_3, 3-CH_3, selROE on H-3 and DFT geometry optimalization (from bottom)

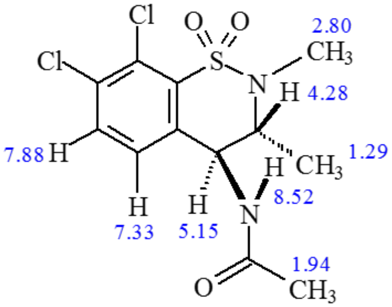

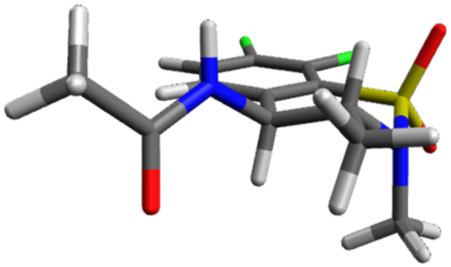

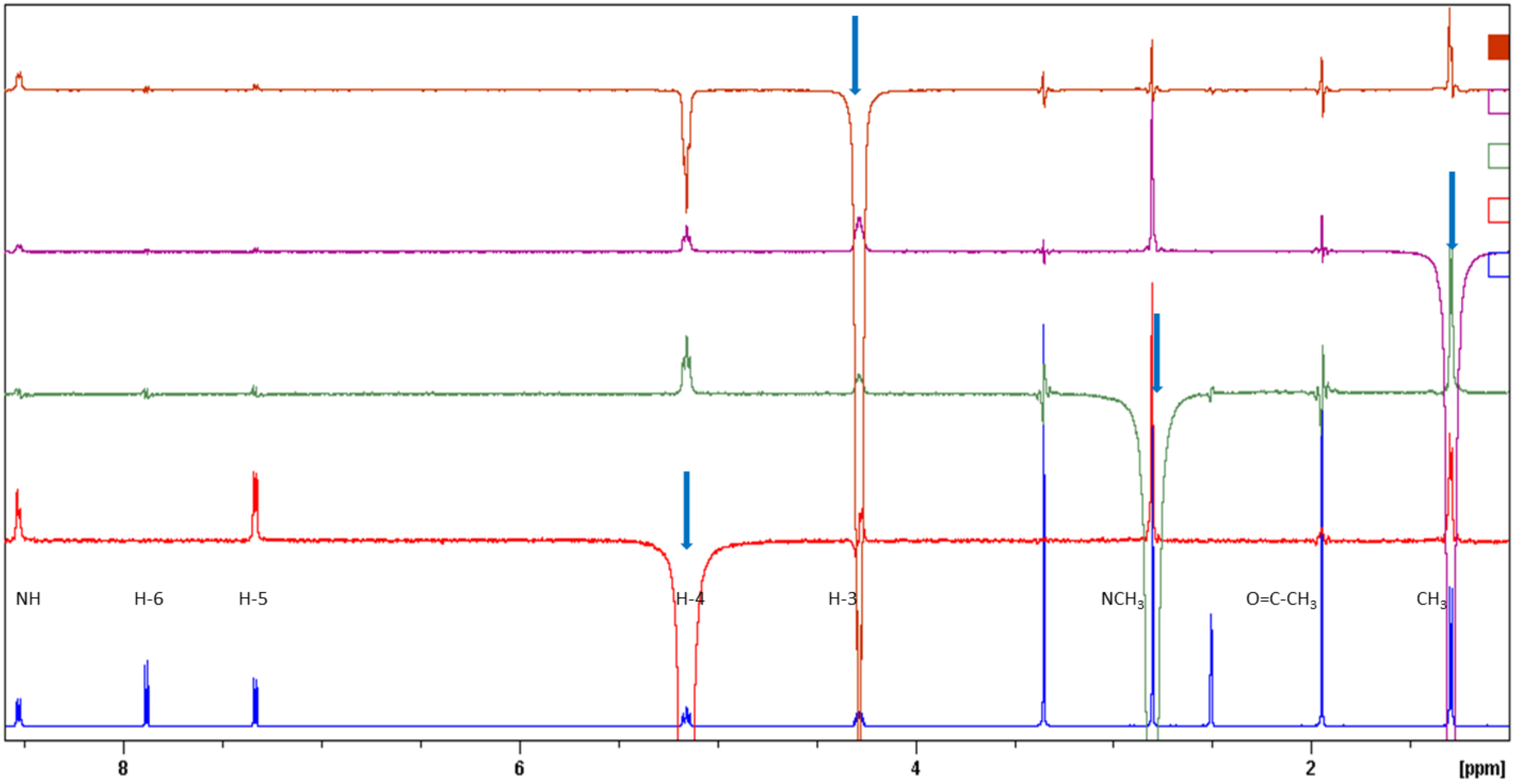

In the 85%/15% mixture of **3b** diastereomers, identification of the ^{1}H signals of the minor ***cis*-3b** by selTOCSY and selNOE on δNH: 8.28 ppm, stereostructure supported also with DFT geometry optimization

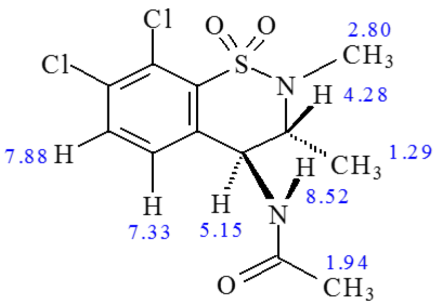

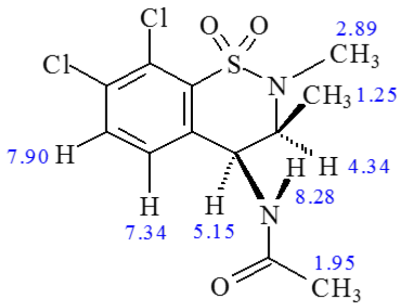

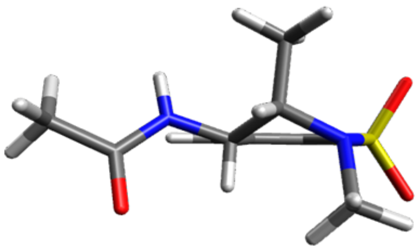

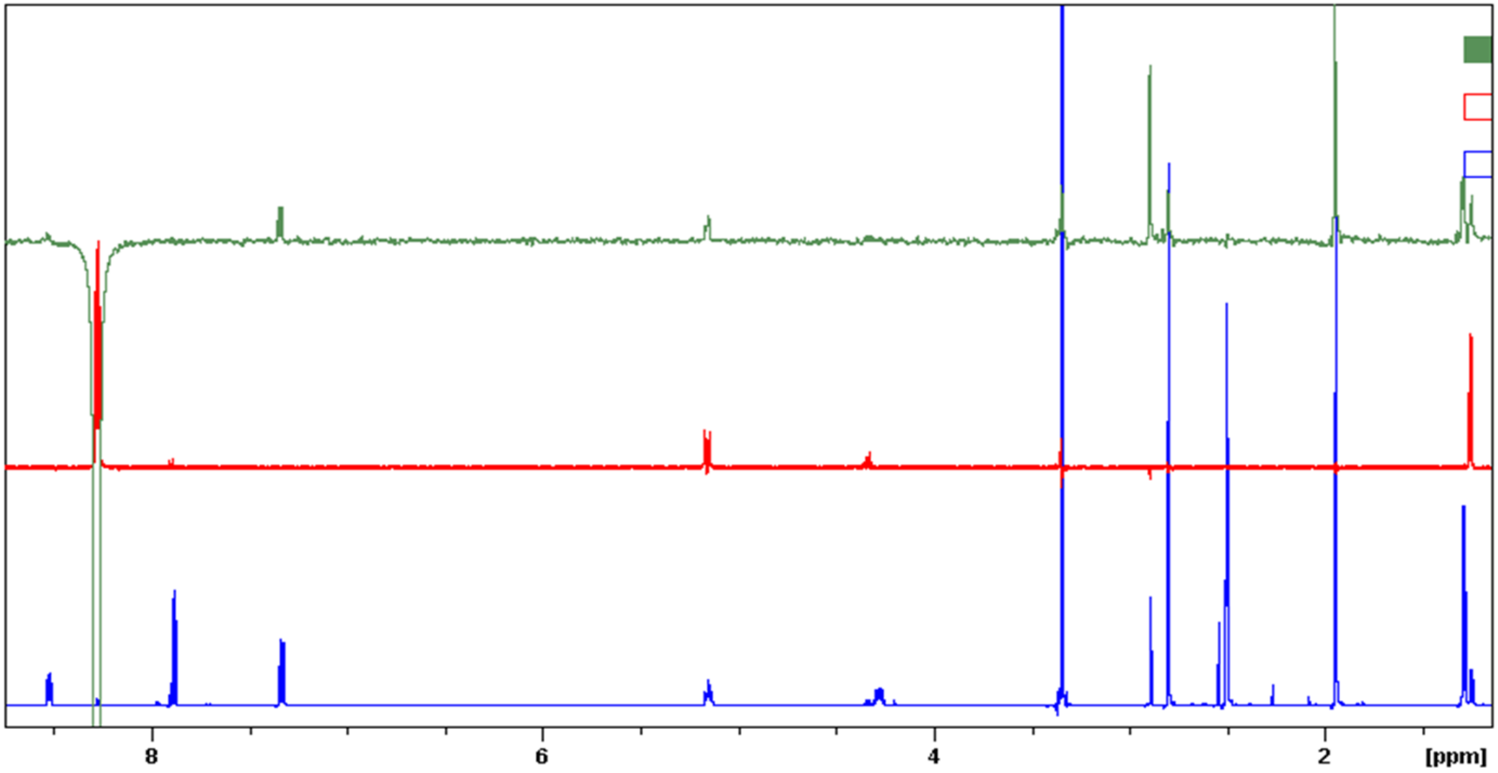

Investigation of the H-4 and H-3 signals detected in the 85%/15% mixture of **3b** diastereomers

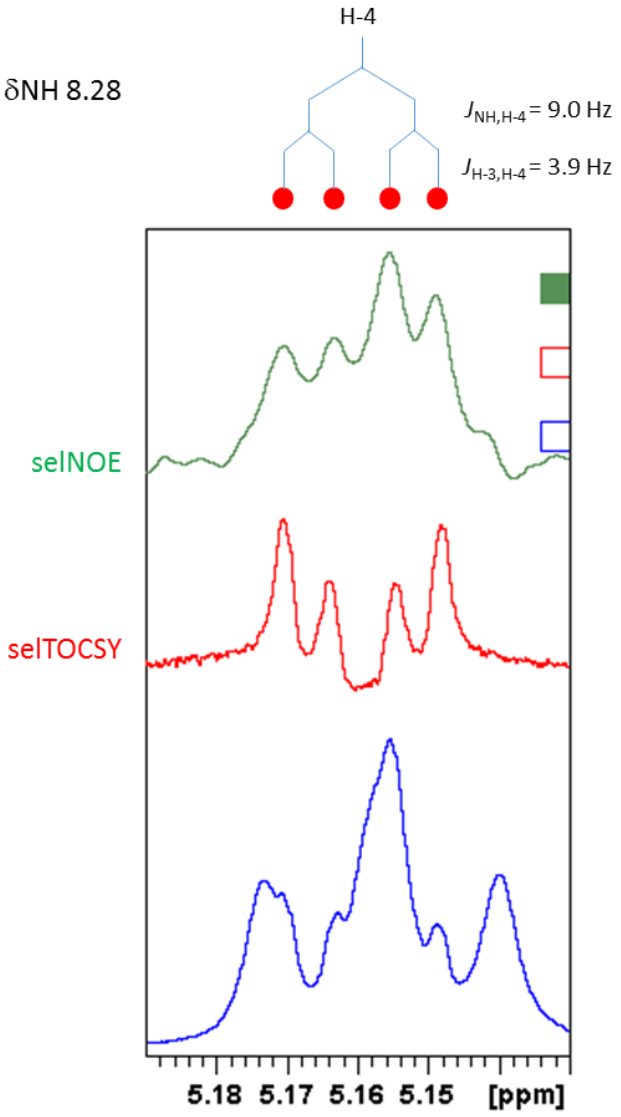

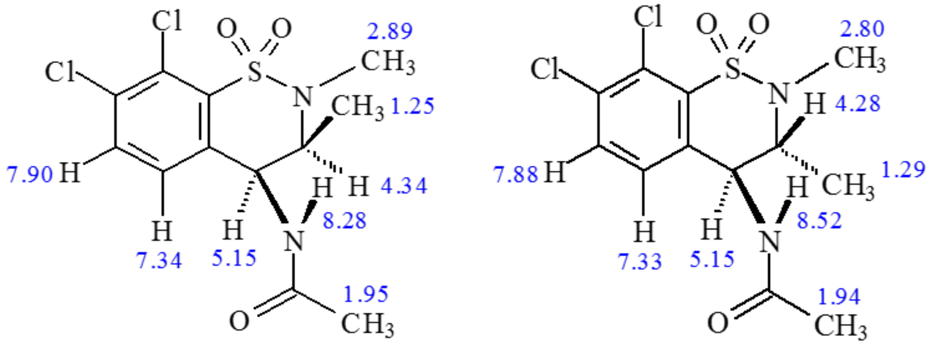

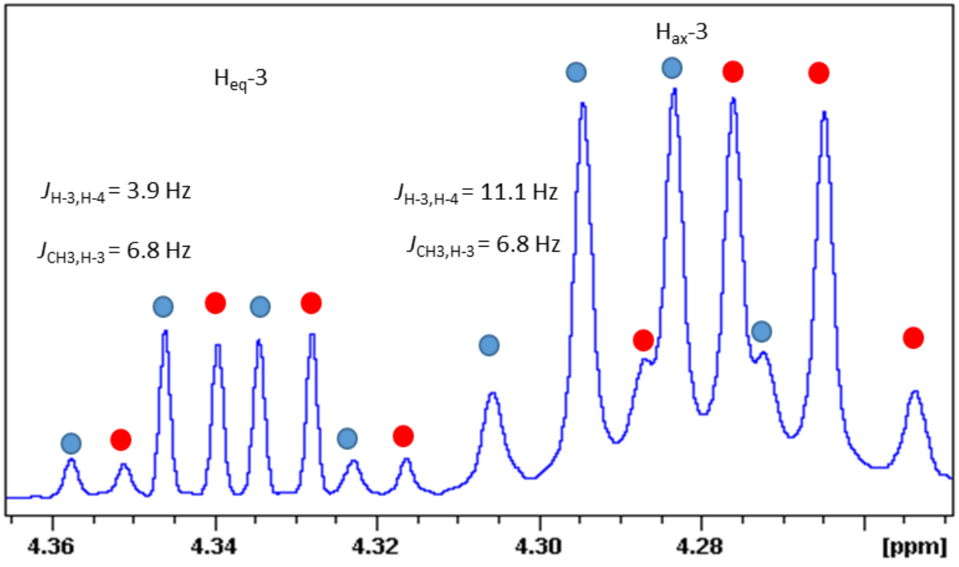

In the 85%/15% mixture of **3b** isomers, identification of the ^{13}C signals of the minor ***cis*-3b** by DEPTQ and edHSQC

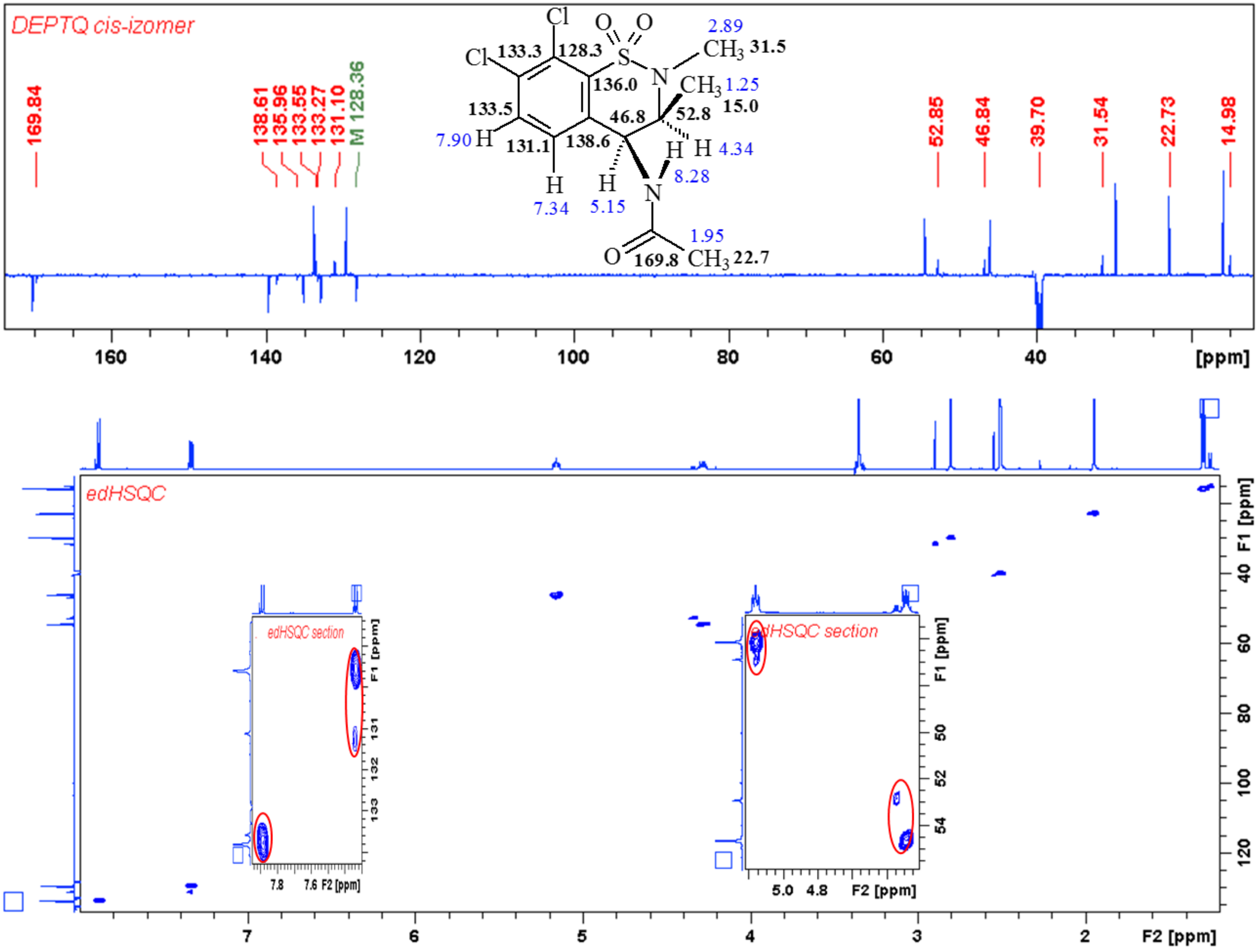

Reaction mixture of **1a** and 1 equiv ***t*-BuOK** in [D$_6$]DMSO: compound **II**$^-$ and ***t*-BuO**$^-$, ^{1}H NMR and DEPTQ

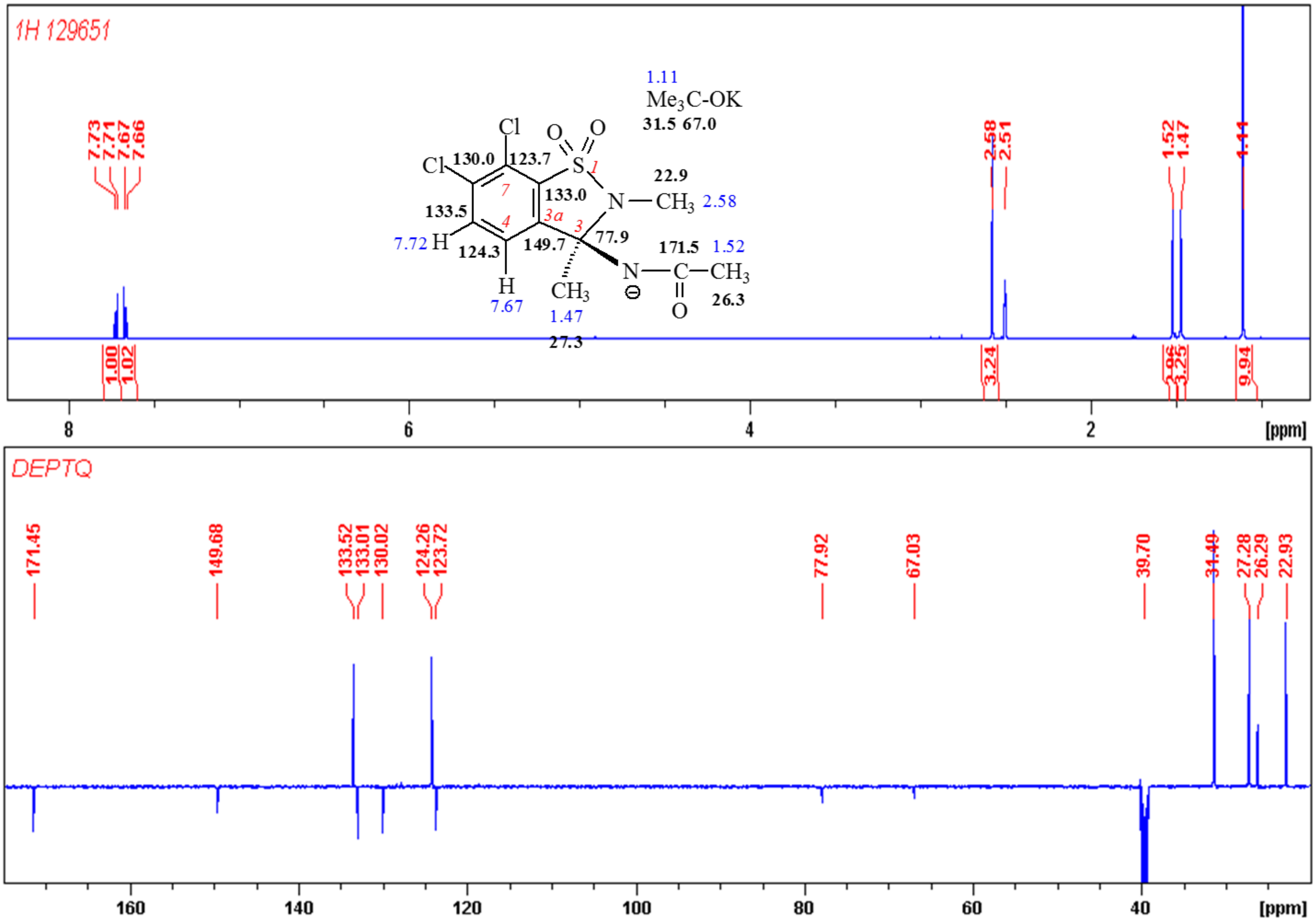

Reaction mixture of **1a** and 1 equiv ***t*-BuOK** in [D6]DMSO: compound **II**⁻ and ***t*-BuO**⁻, HSQC and HMBC

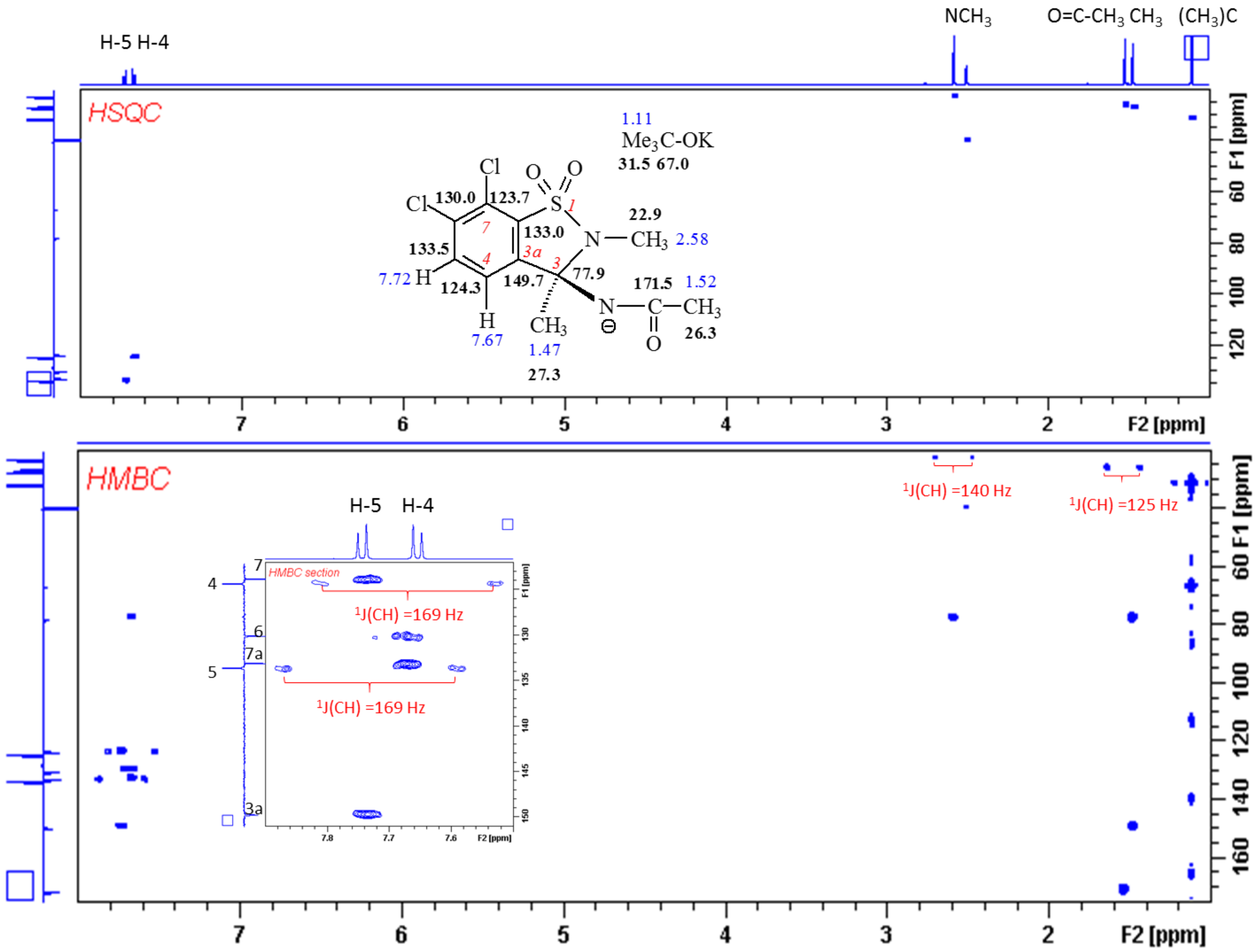

Reaction mixture of **1a** and 2 equiv ***t*-BuOK** in [D$_6$]DMSO: compound **II**$^-$, **IV**$^{2-}$and ***t*-BuO**$^-$, HSQC and HMBC

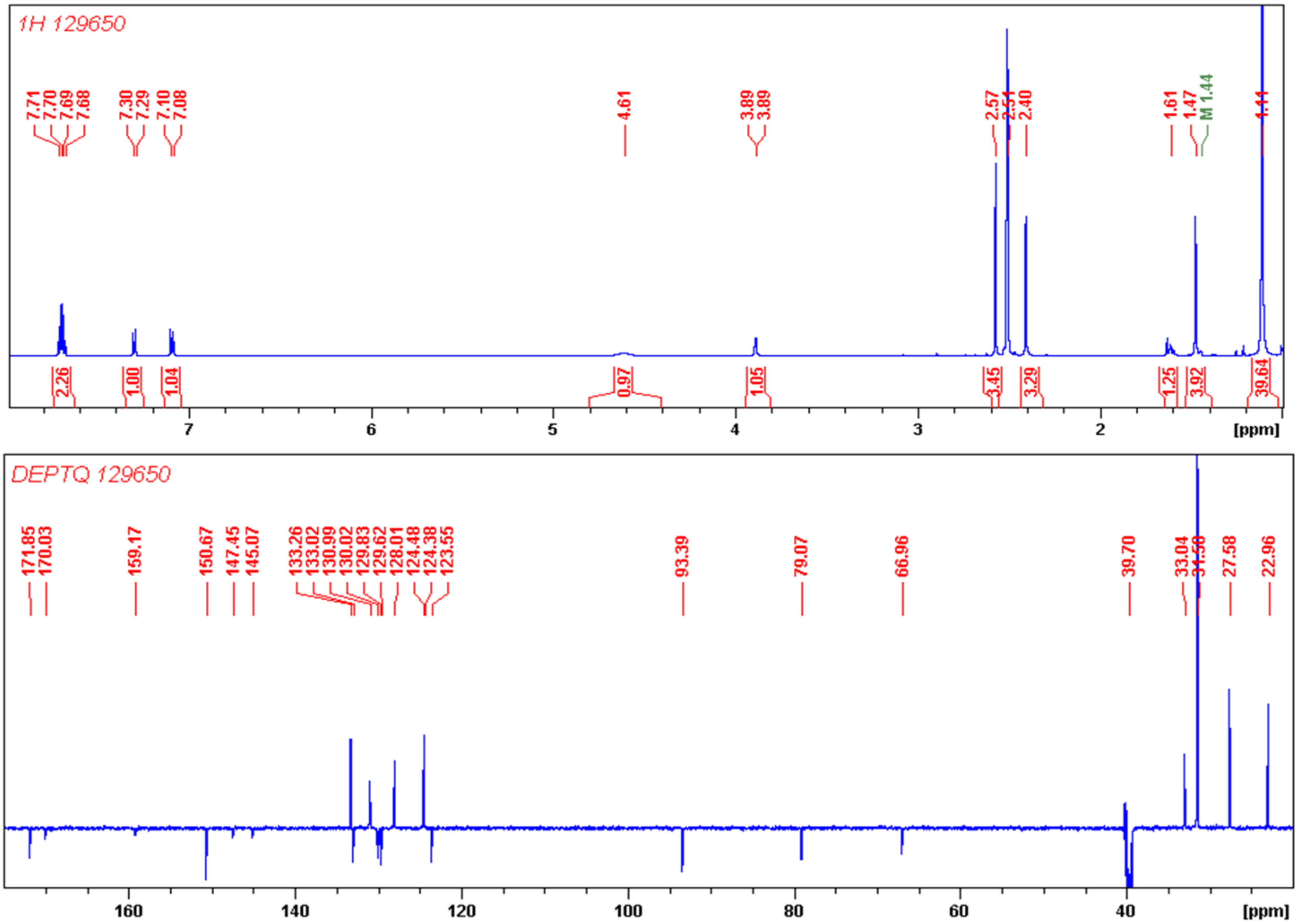

Reaction mixture of **1a** and 6 equiv ***t*-BuOK** in [D6]DMSO: compound **IV**$^{2-}$ and ***t*-BuO**$^{-}$, ^{1}H, DEPTQ and ^{13}C NMR

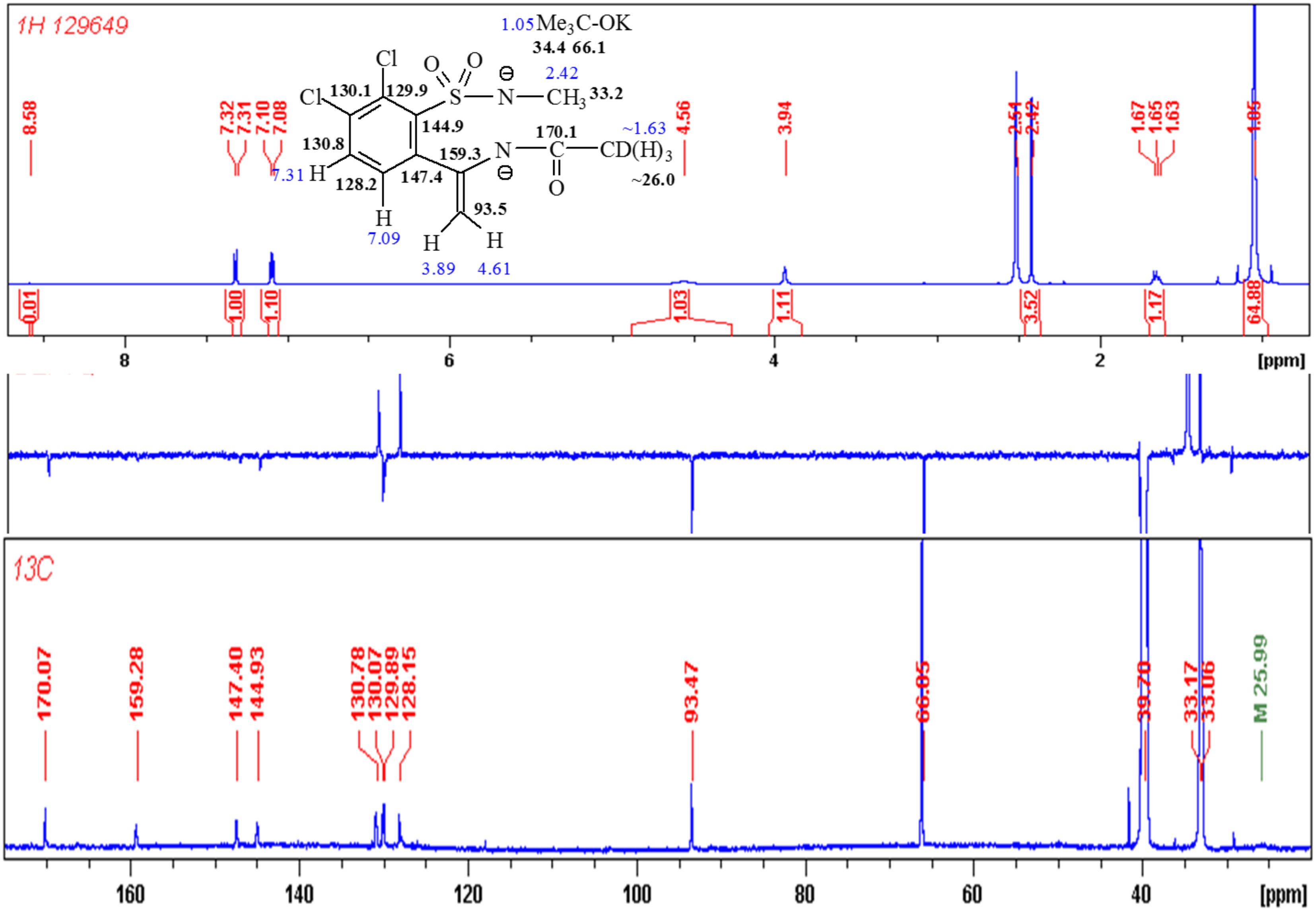

Reaction mixture of **1a** and 6 equiv ***t*-BuOK** in [D6]DMSO]DMSO: compound **IV**$^{2-}$ and ***t*-BuO**$^{-}$, HSQC and HMBC

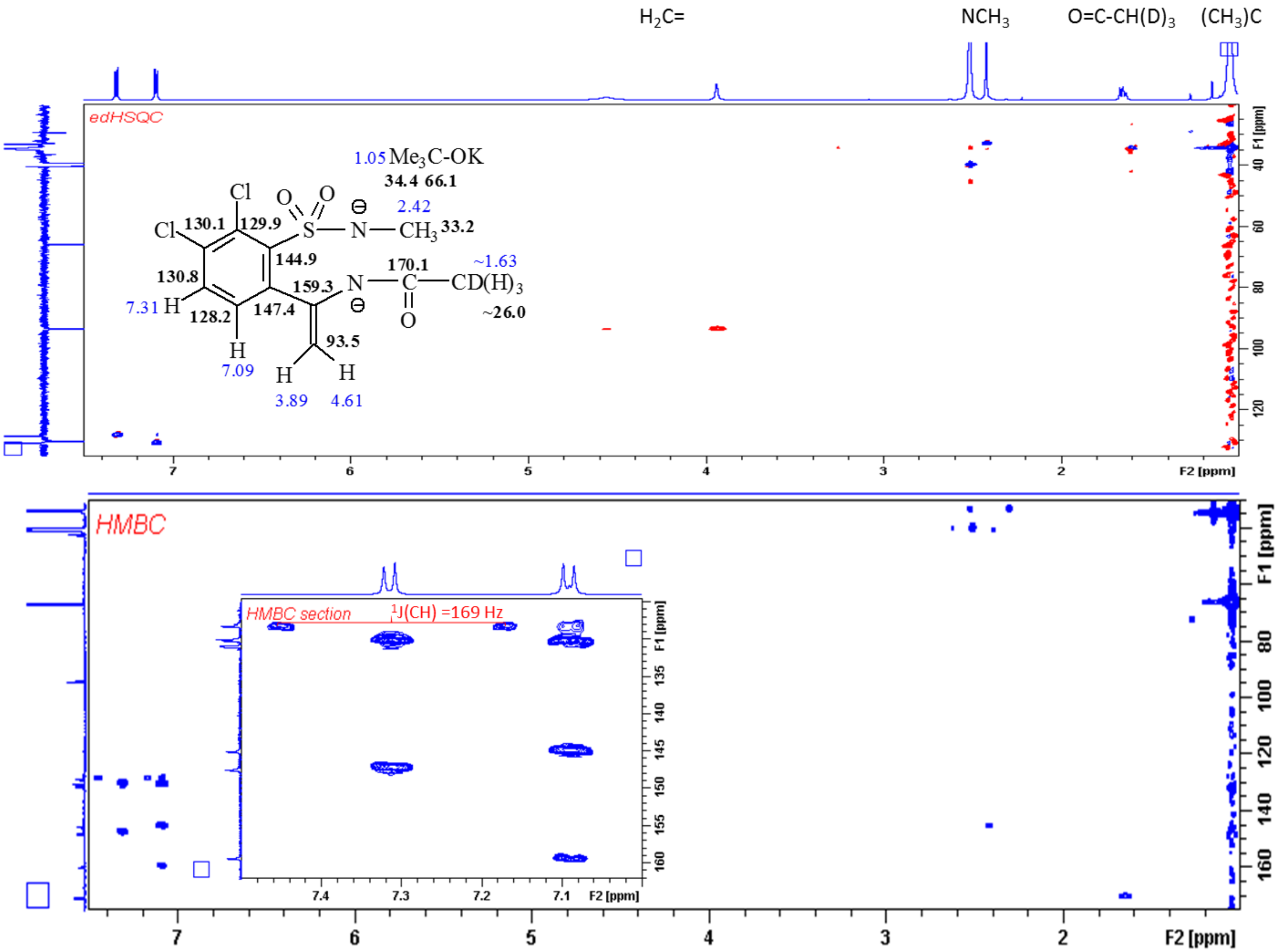

Compound **[D]3a**, ^{1}H, HSQC and ^{13}C NMR

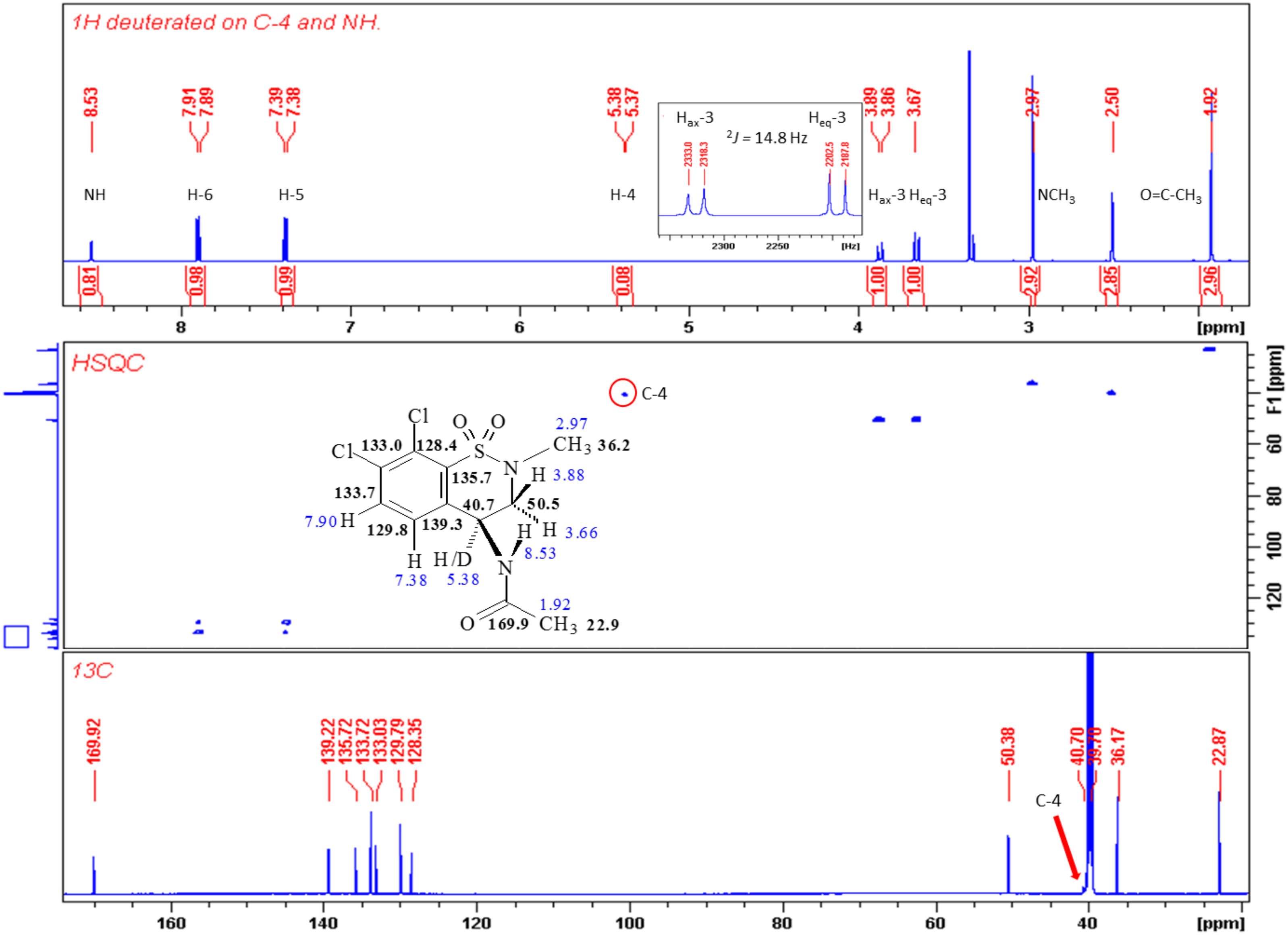

NMR spectra

Compound **1c**, ^{1}H and ^{13}C NMR (600/150 MHz, [D$_6$]DMSO)

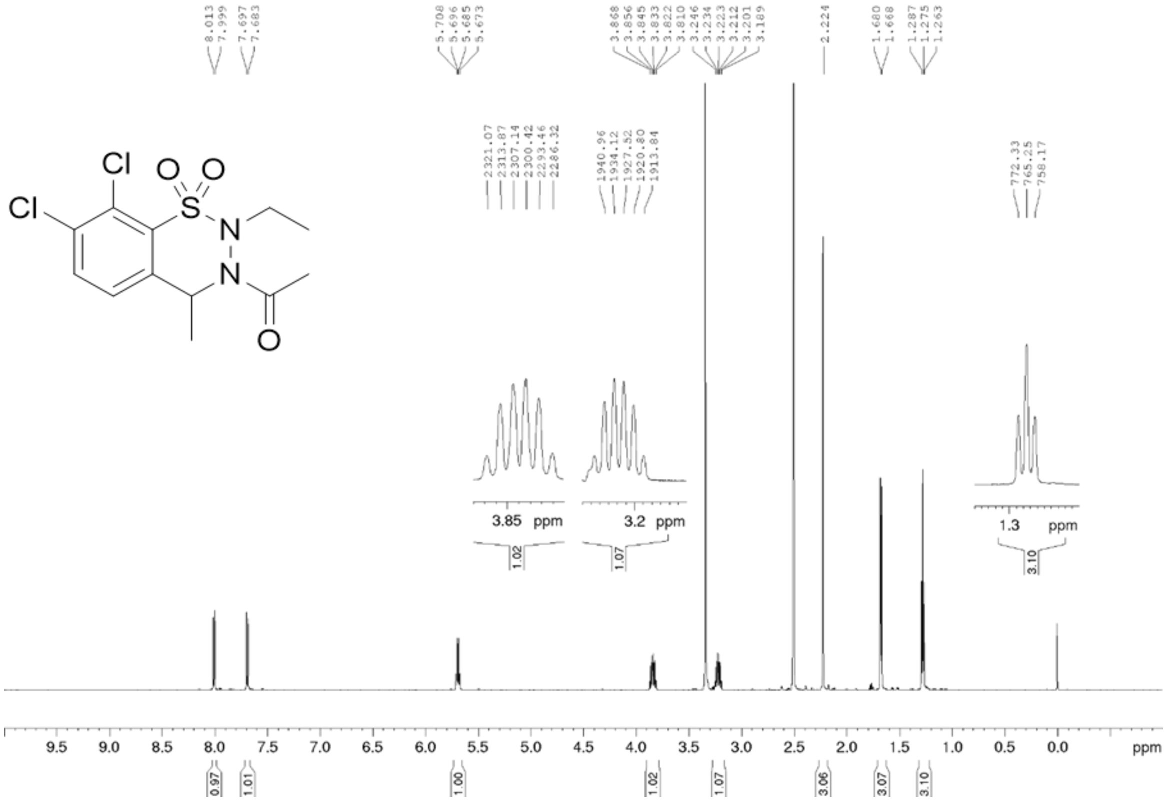

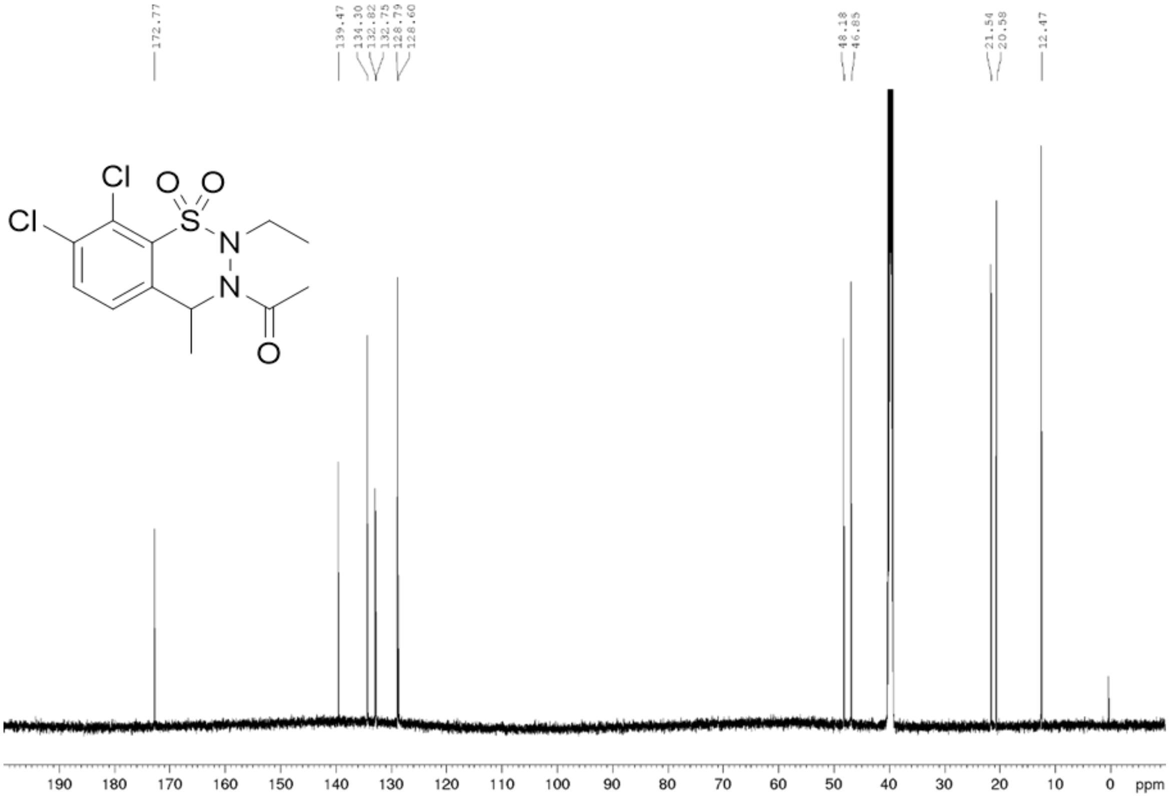

Compound **1d**, ^{1}H and ^{13}C NMR (600/150 MHz, [D$_6$]DMSO)

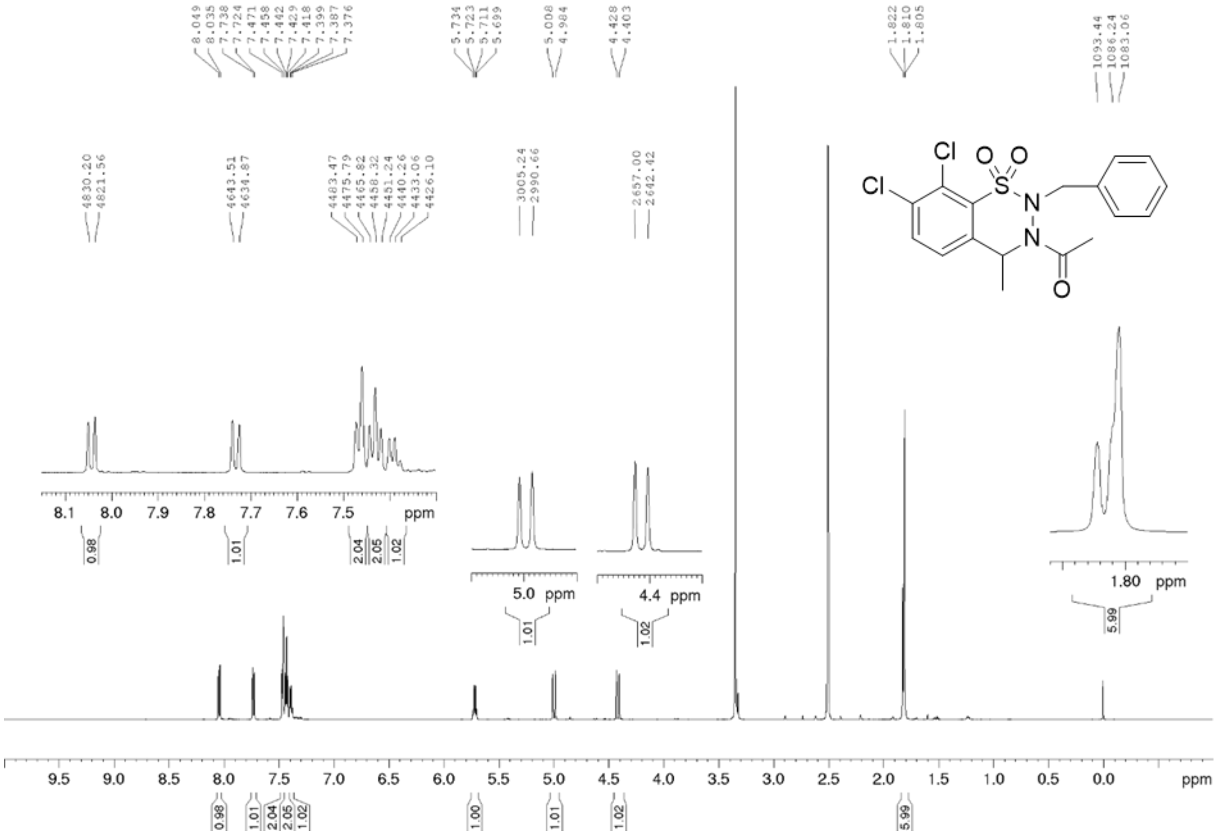

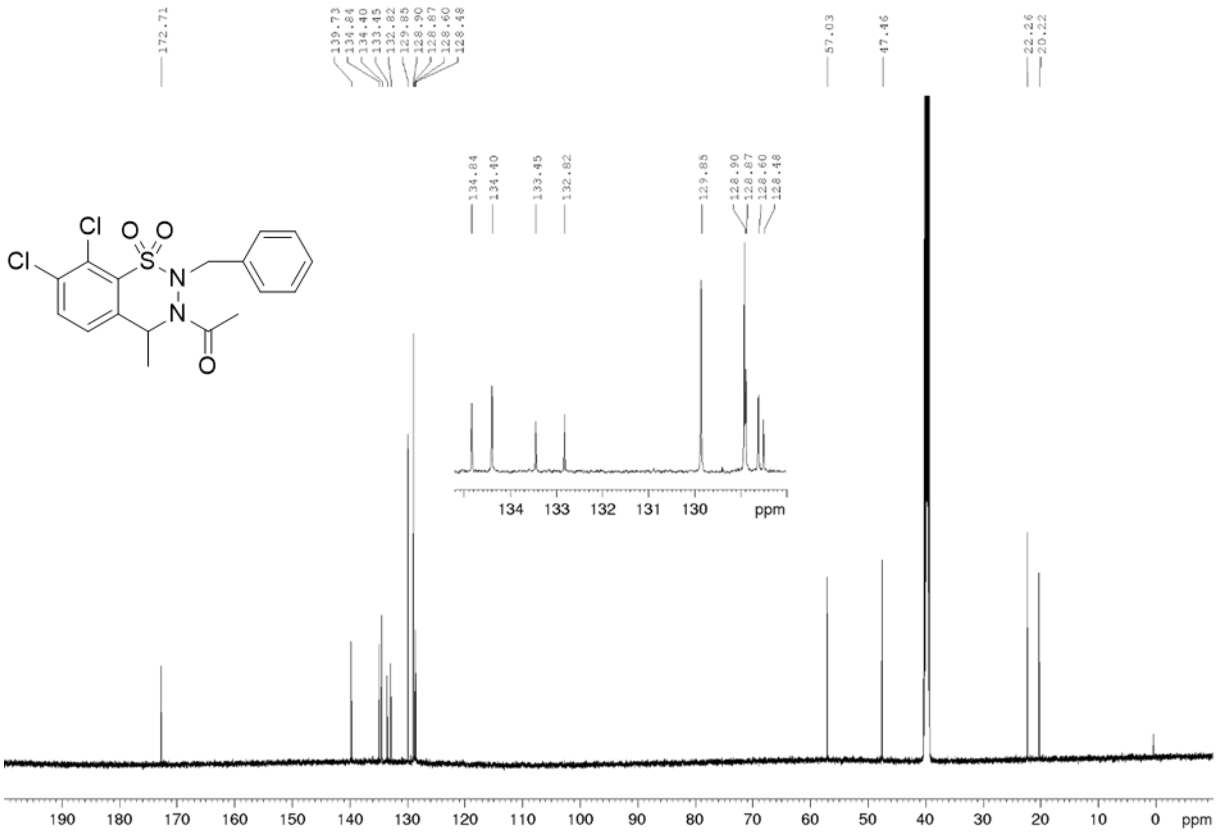

Compound **7b**, ^{1}H and ^{13}C NMR (600/150 MHz, $CDCl_3$)

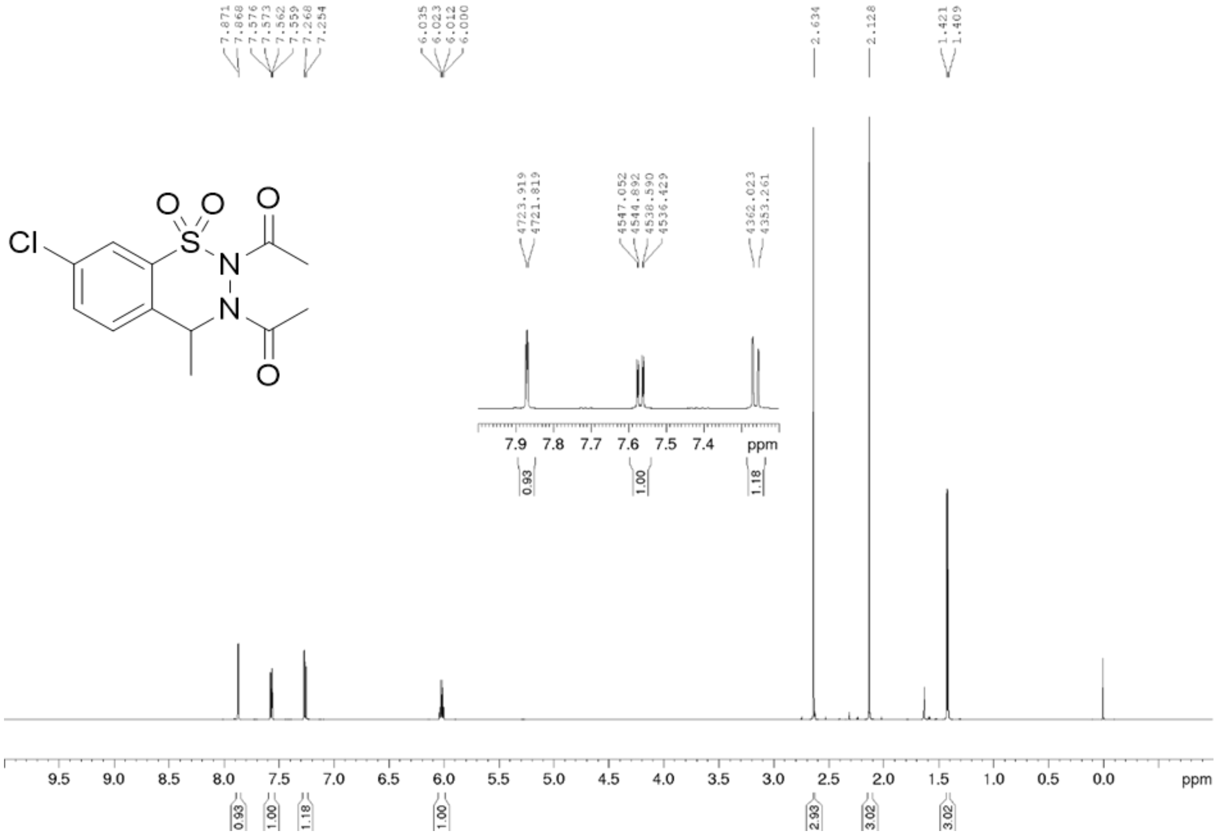

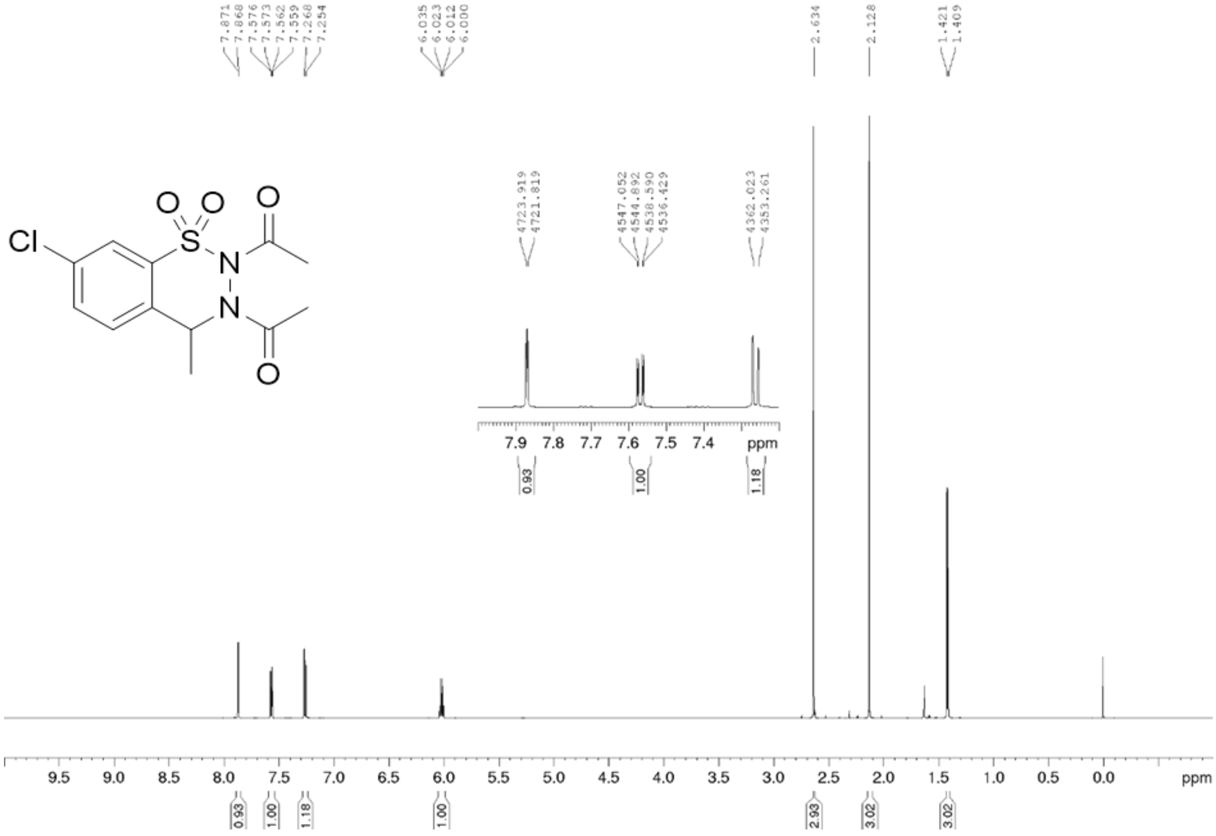

Compound **7c**, ^{1}H and ^{13}C NMR (600/150 MHz, $CDCl_3$)

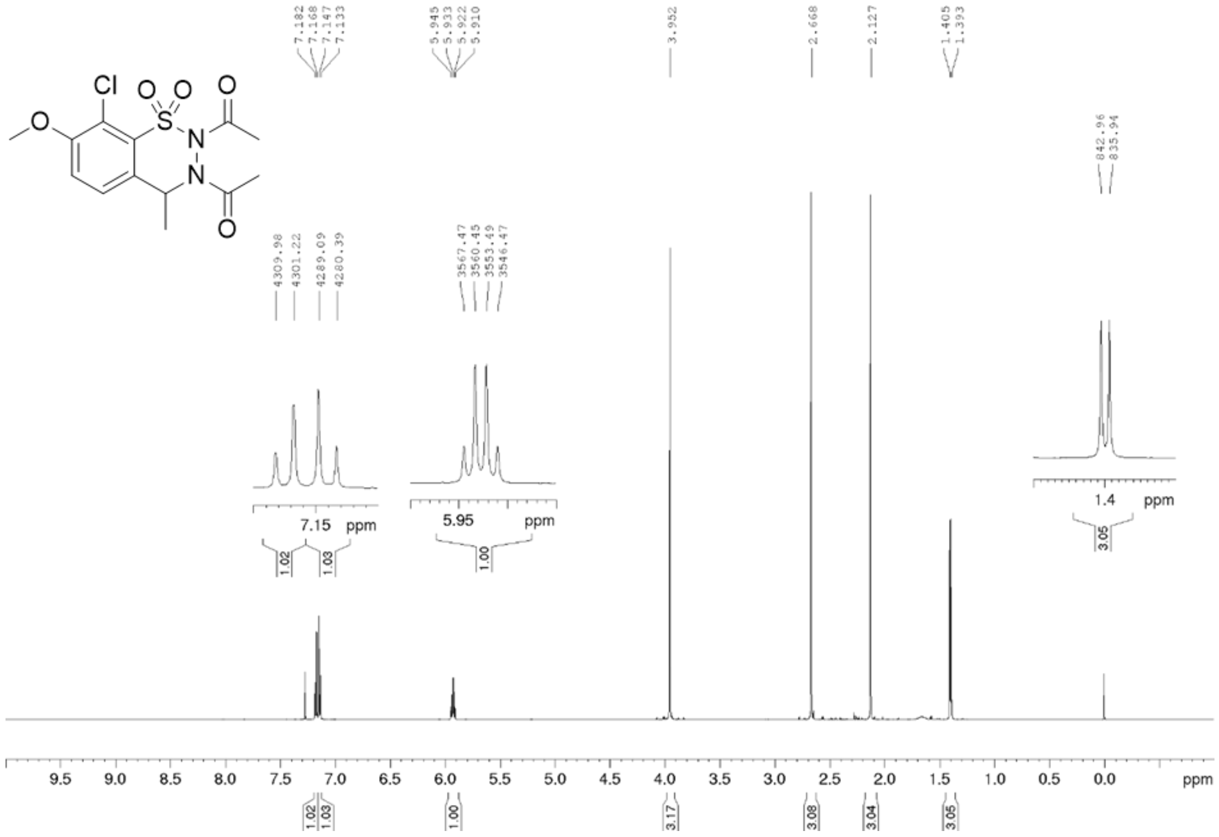

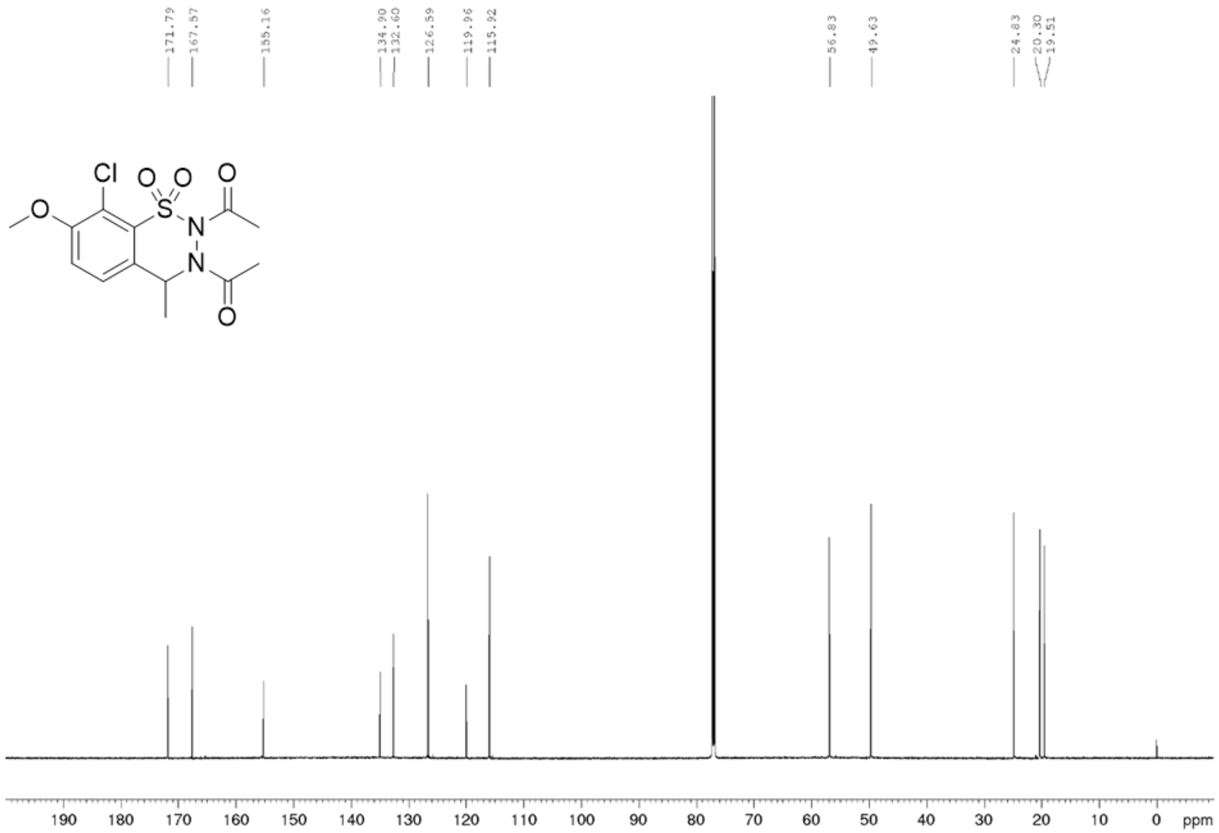

Compound **7d**, ^{1}H and ^{13}C NMR (600/150 MHz, $CDCl_3$)

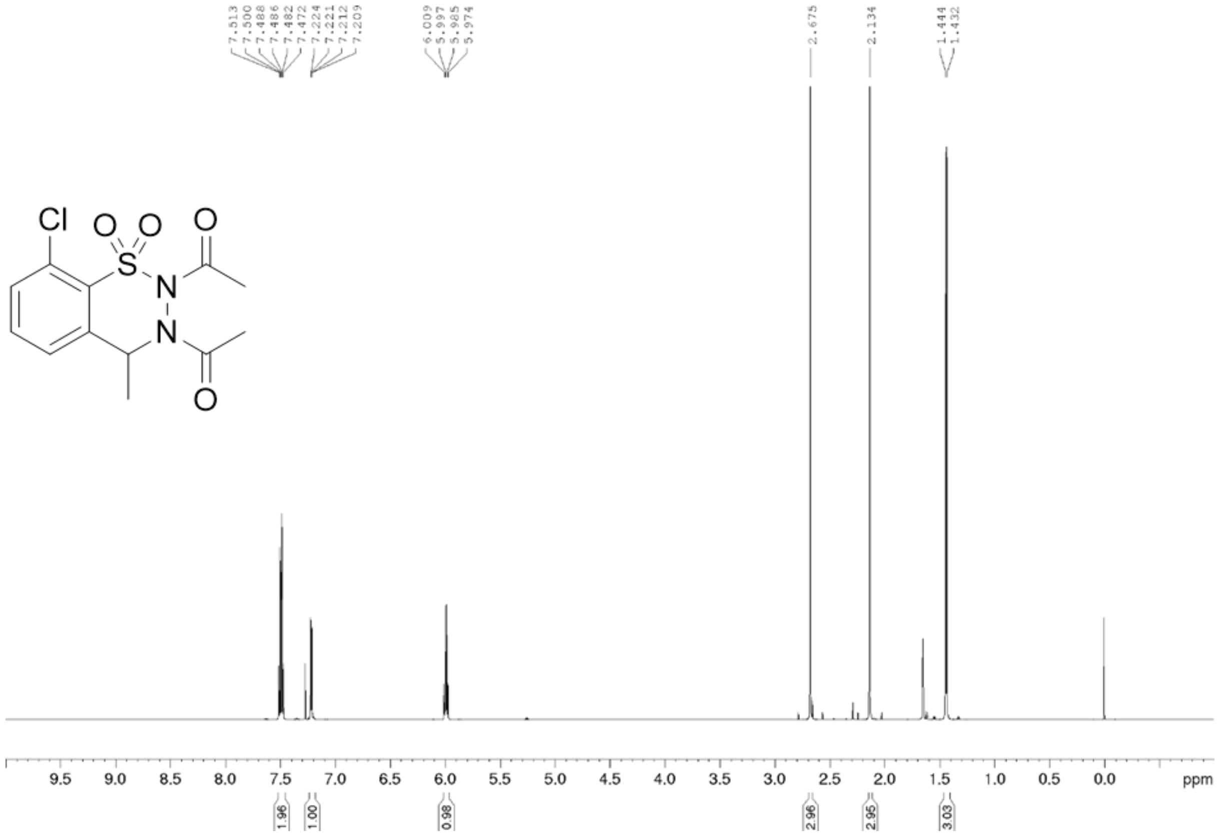

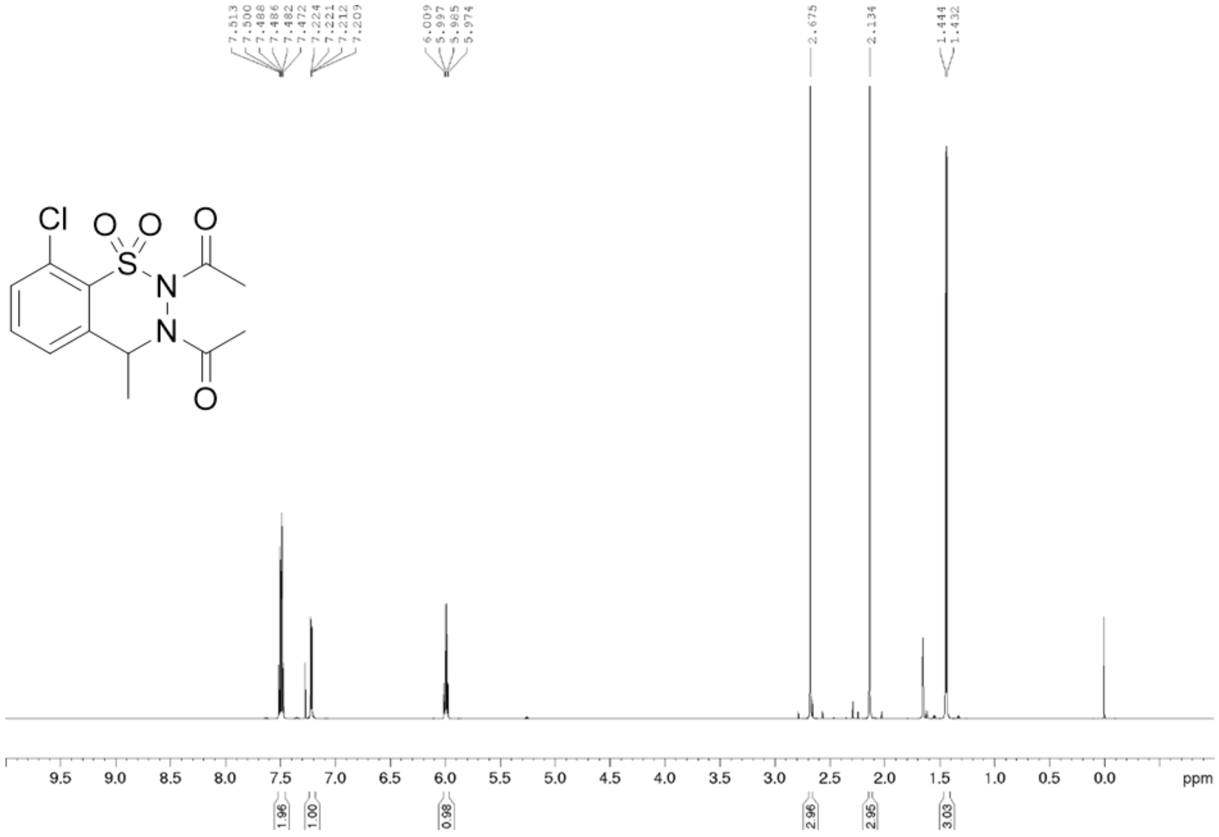

Compound **7e**, ^{1}H and ^{13}C NMR (600/150 MHz, $CDCl_3$)

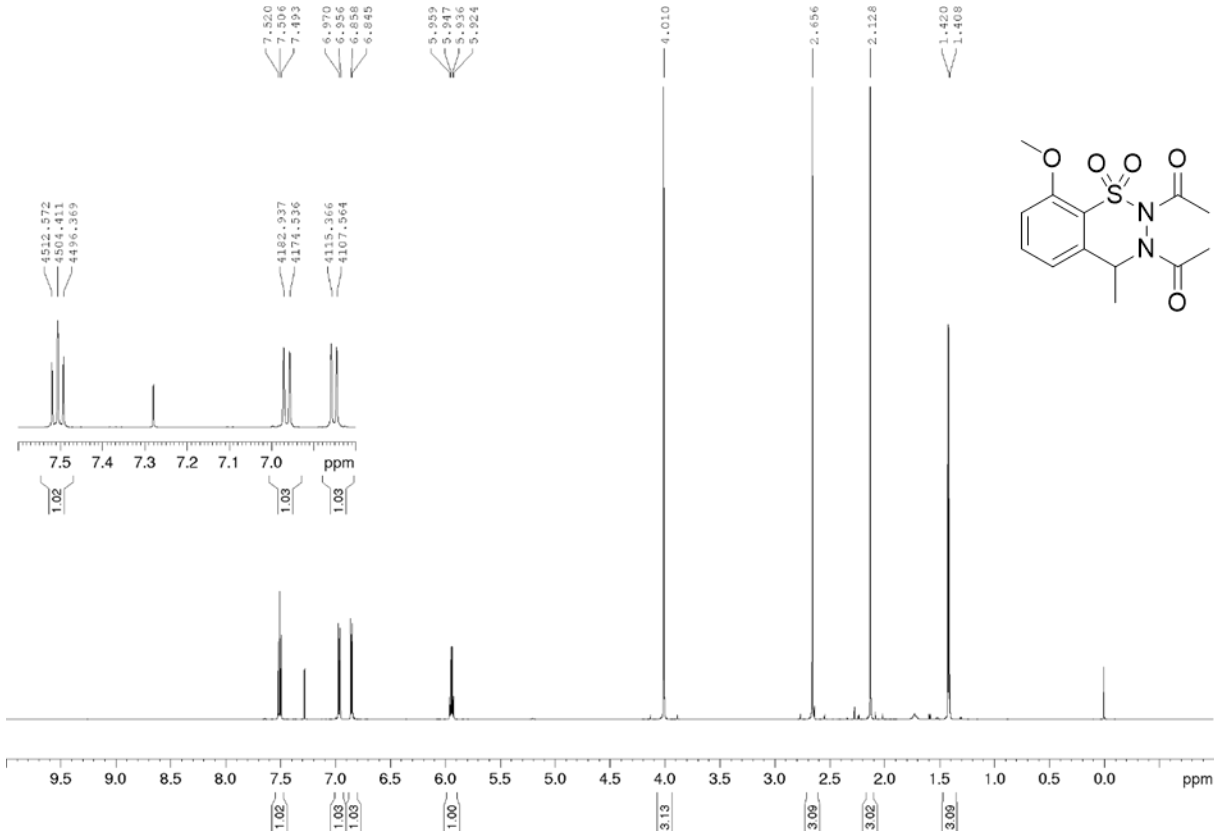

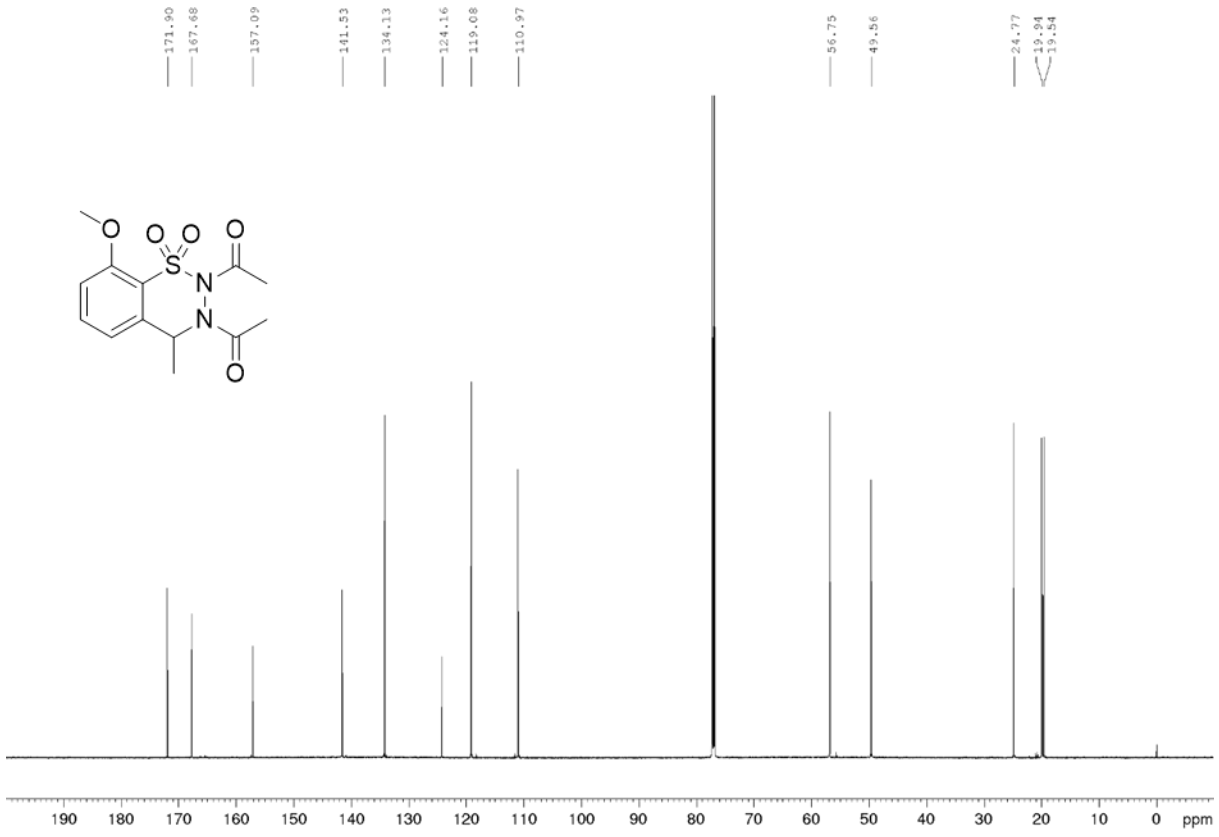

Compound **7f**, ^{1}H and ^{13}C NMR (600/150 MHz, $CDCl_3$)

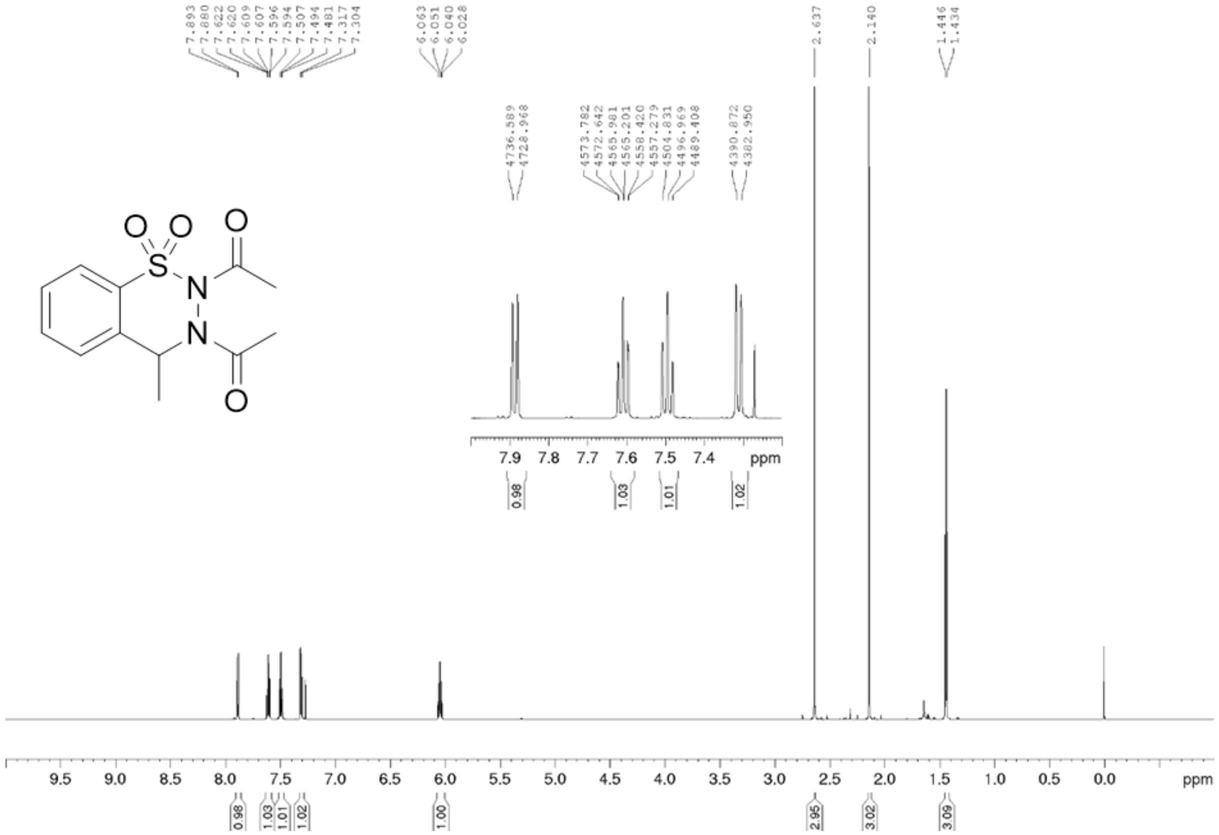

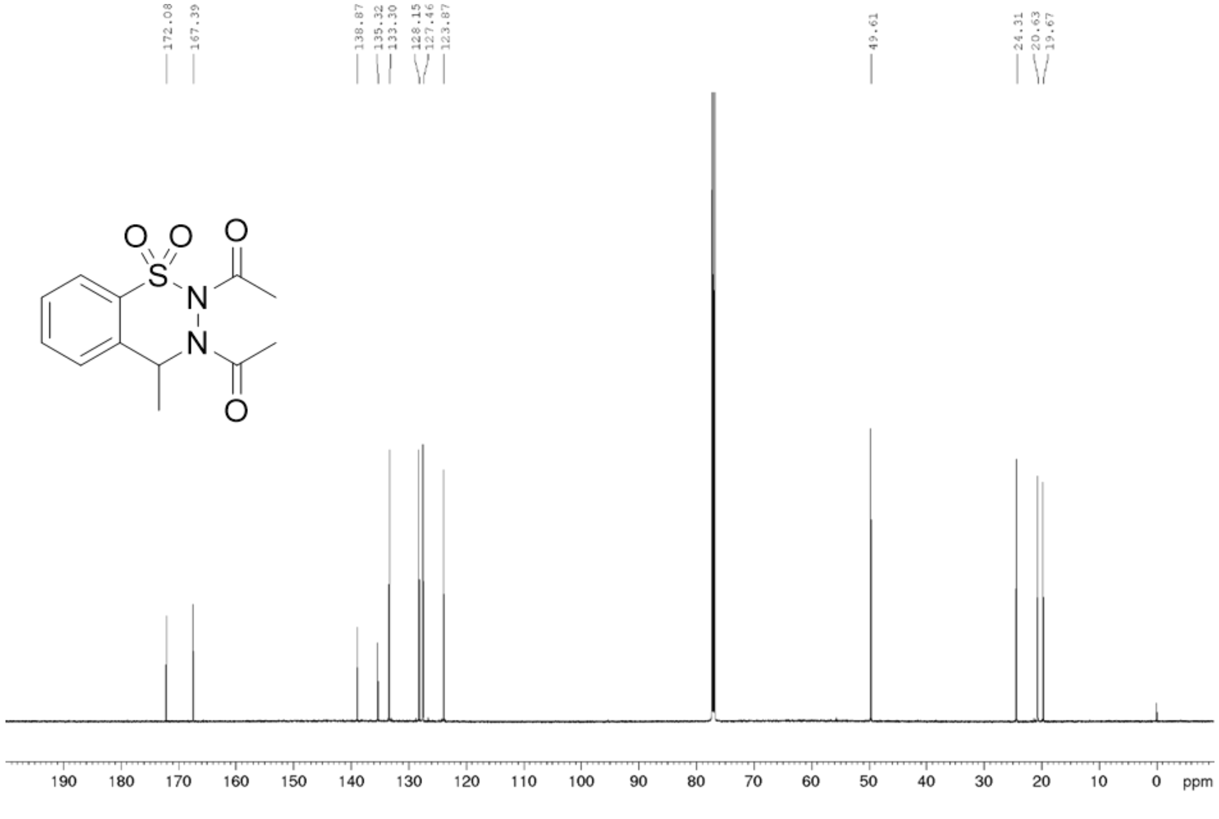

Compound **1k**, ^{1}H and ^{13}C NMR (600/150 MHz, [D$_6$]DMSO)

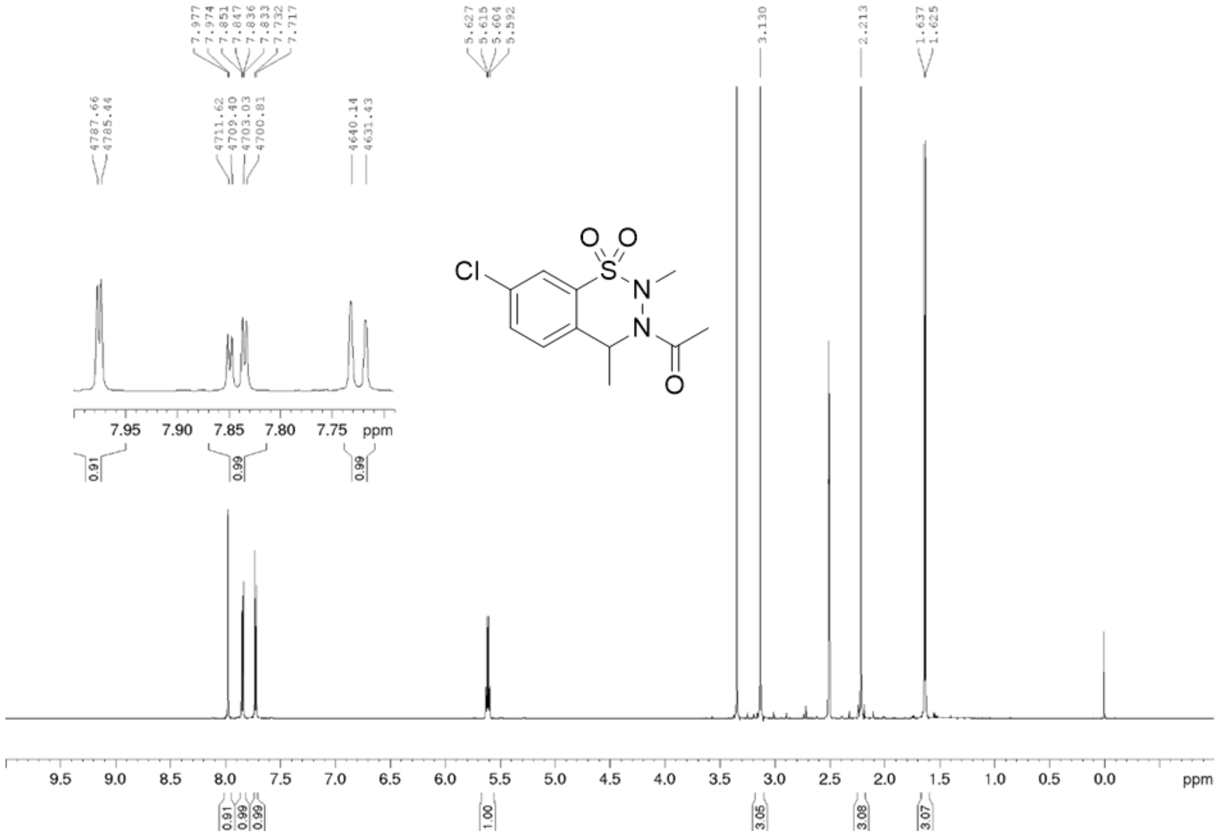

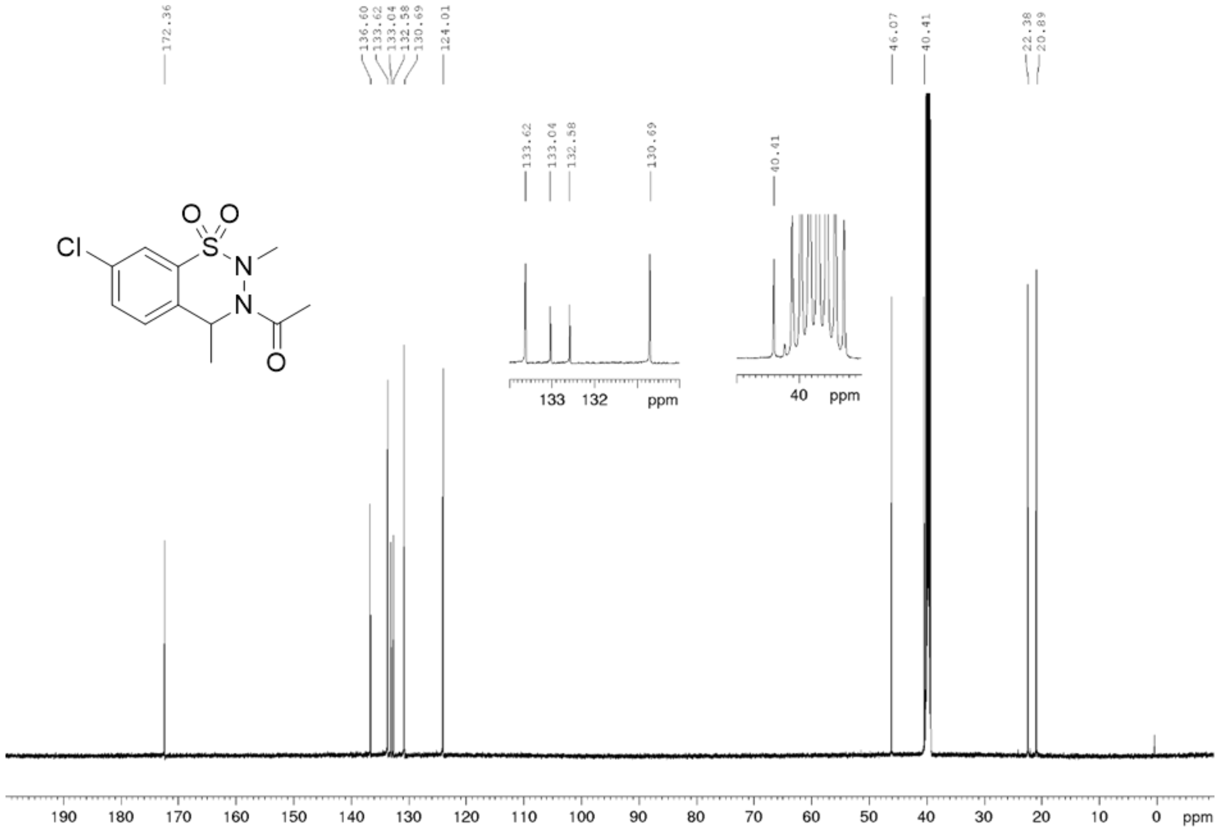

Compound **1l**, ^{1}H and ^{13}C NMR (400/100 MHz, [D$_6$]DMSO)

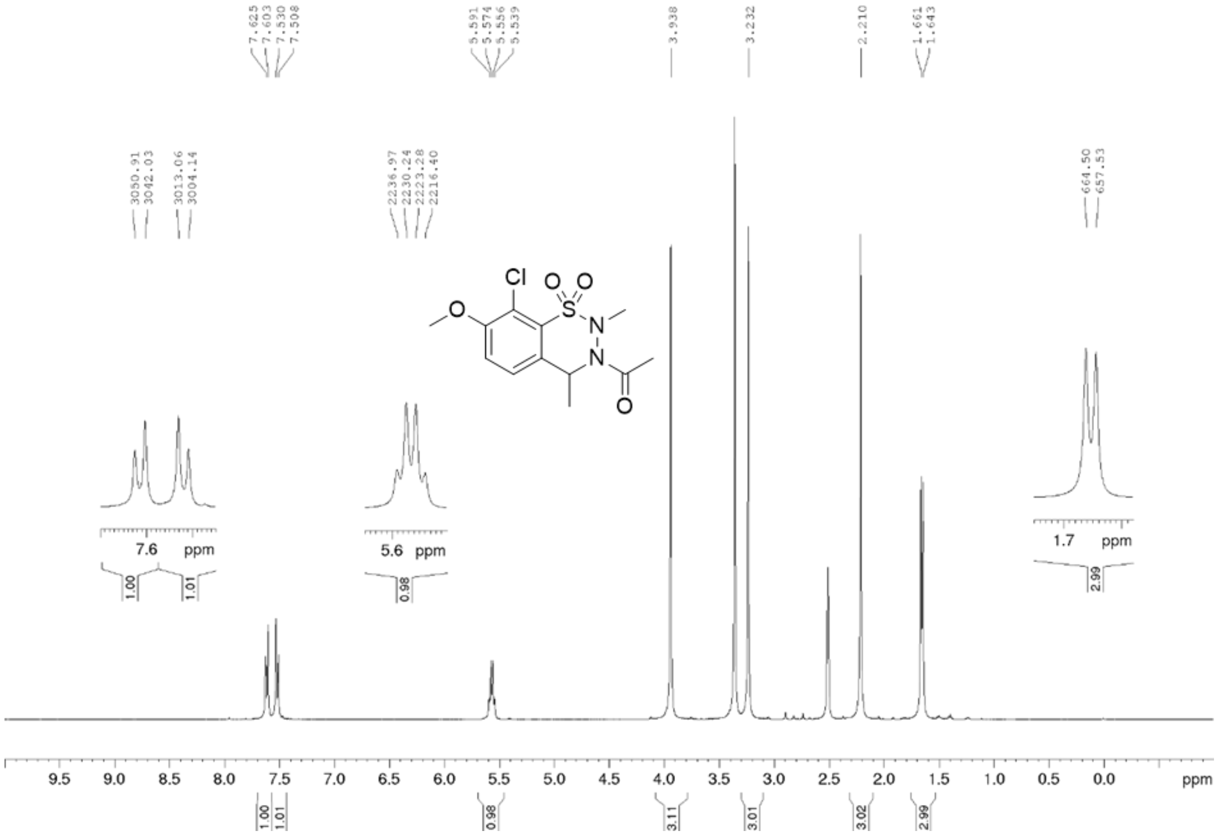

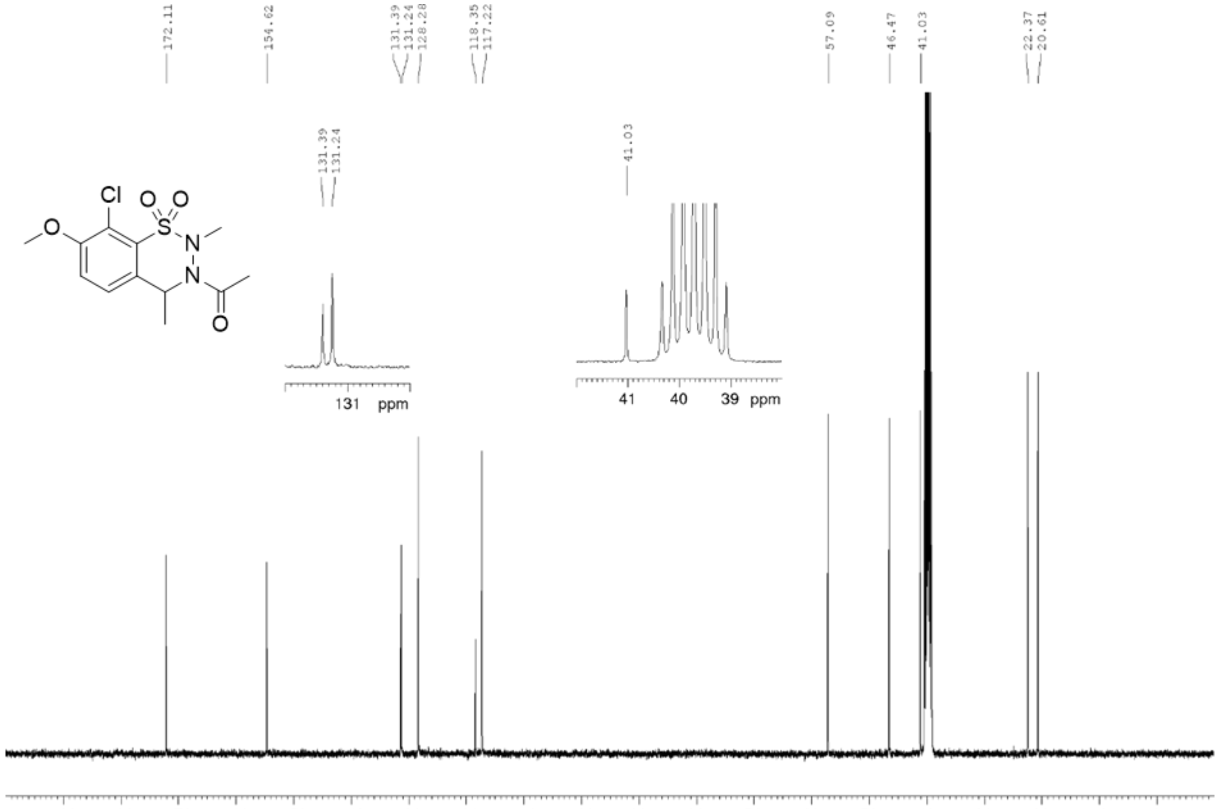

Compound **1m**, ^{1}H and ^{13}C NMR (600/150 MHz, [D$_6$]DMSO)

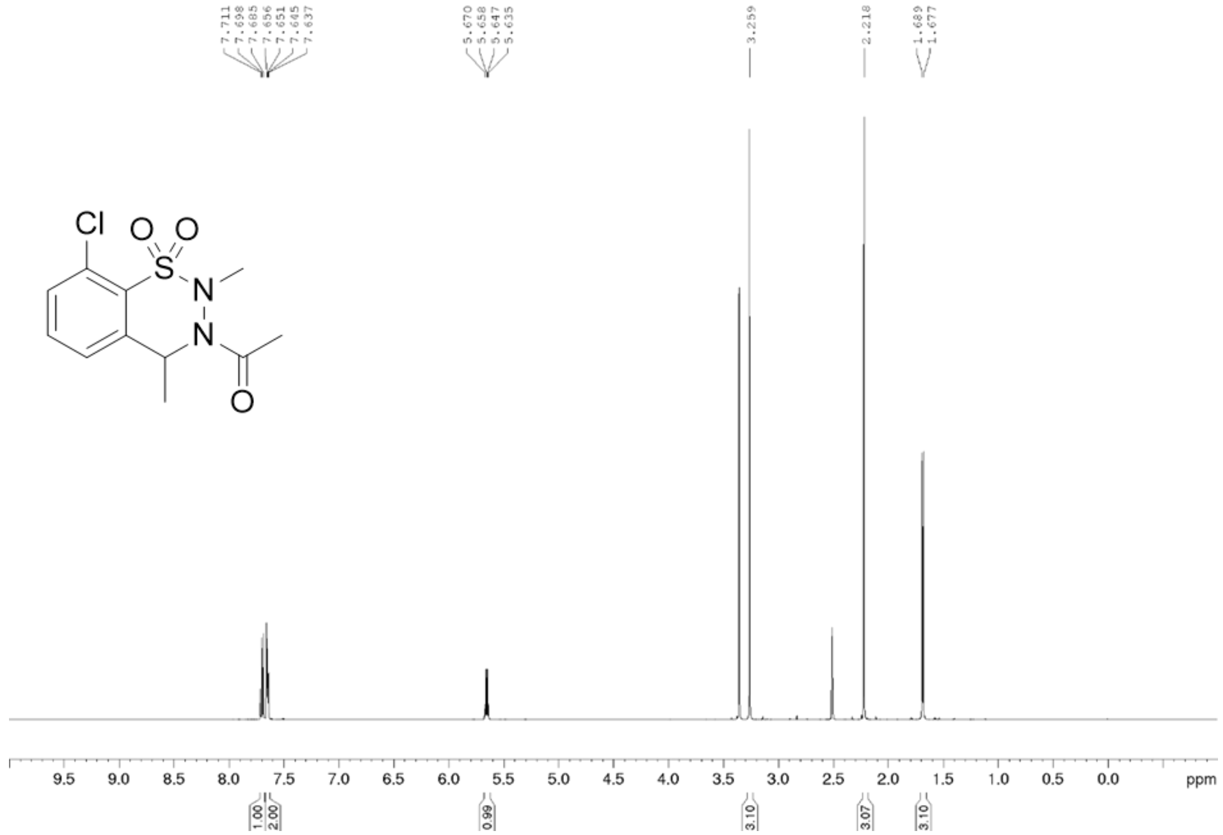

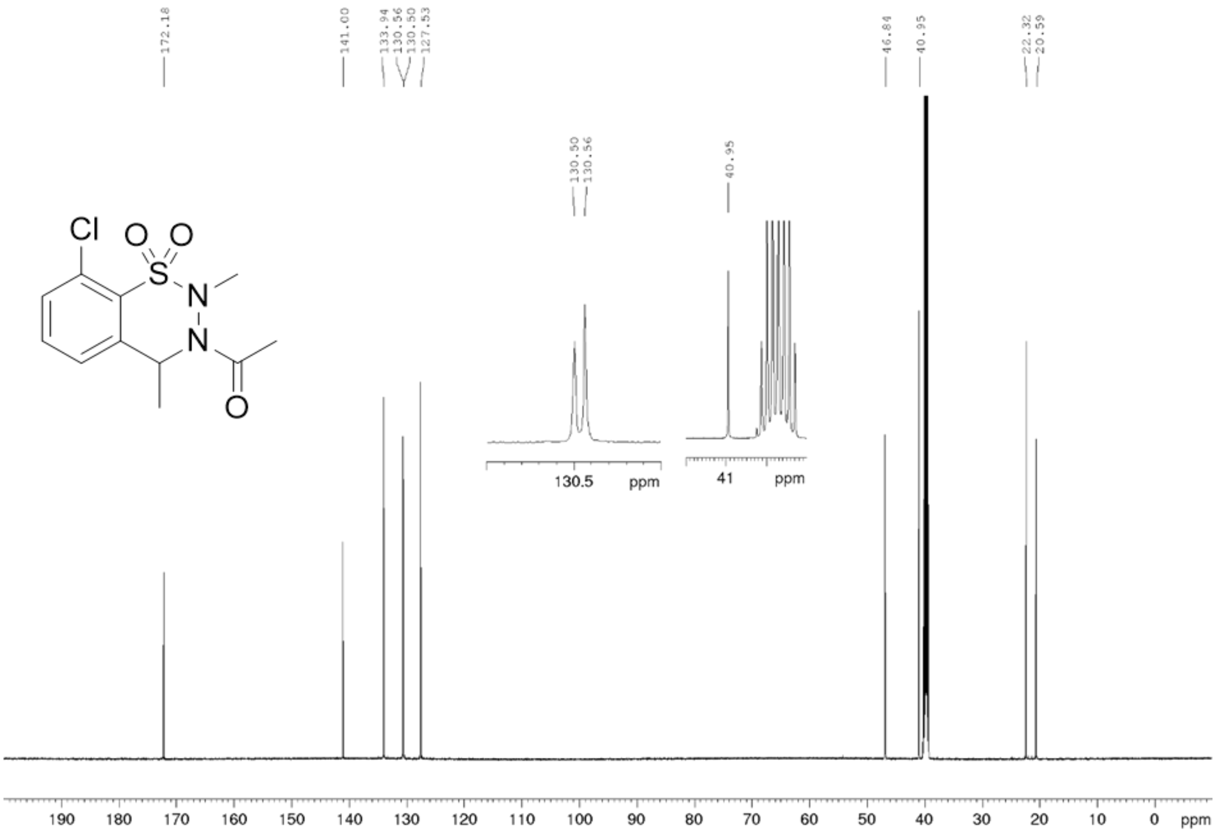

Compound **1n**, ^{1}H and ^{13}C NMR (400/100 MHz, [D_6]DMSO)

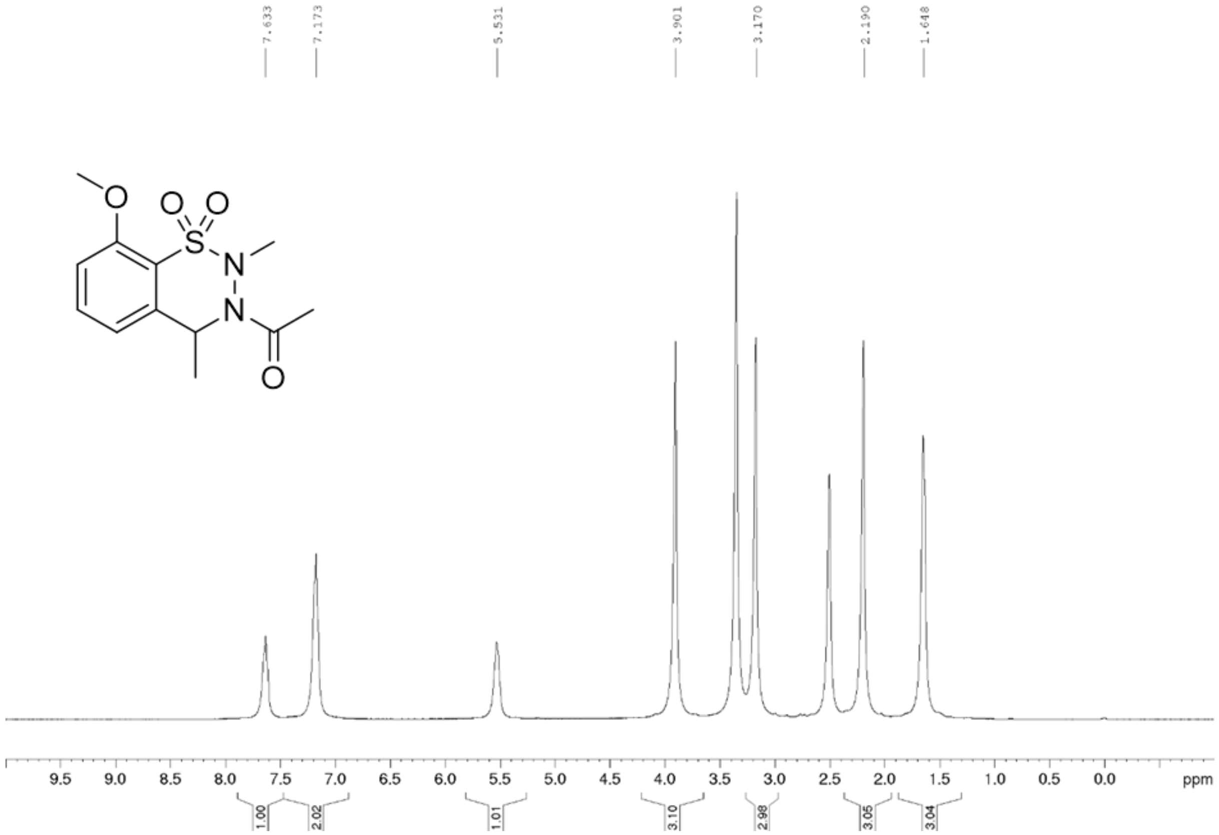

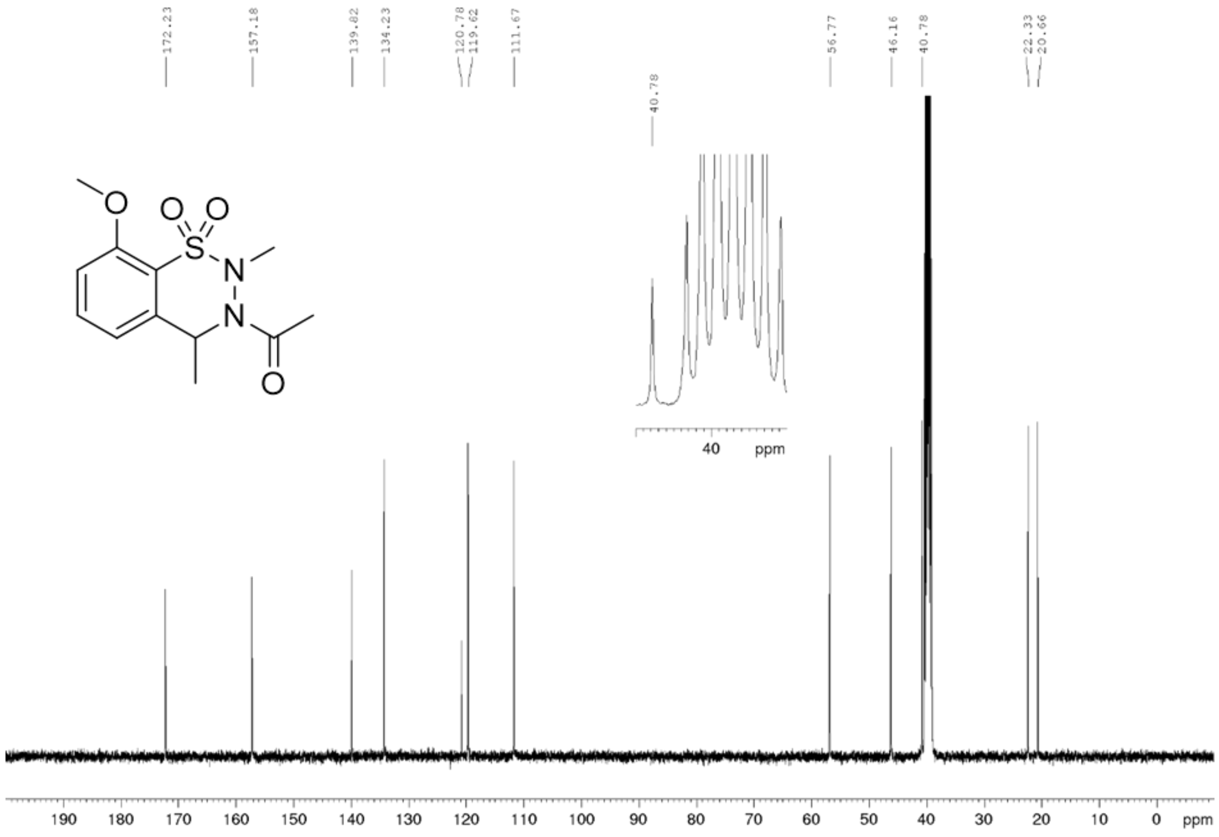

Compound **1o**, ^{1}H and ^{13}C NMR (600/150 MHz, [D$_6$]DMSO)

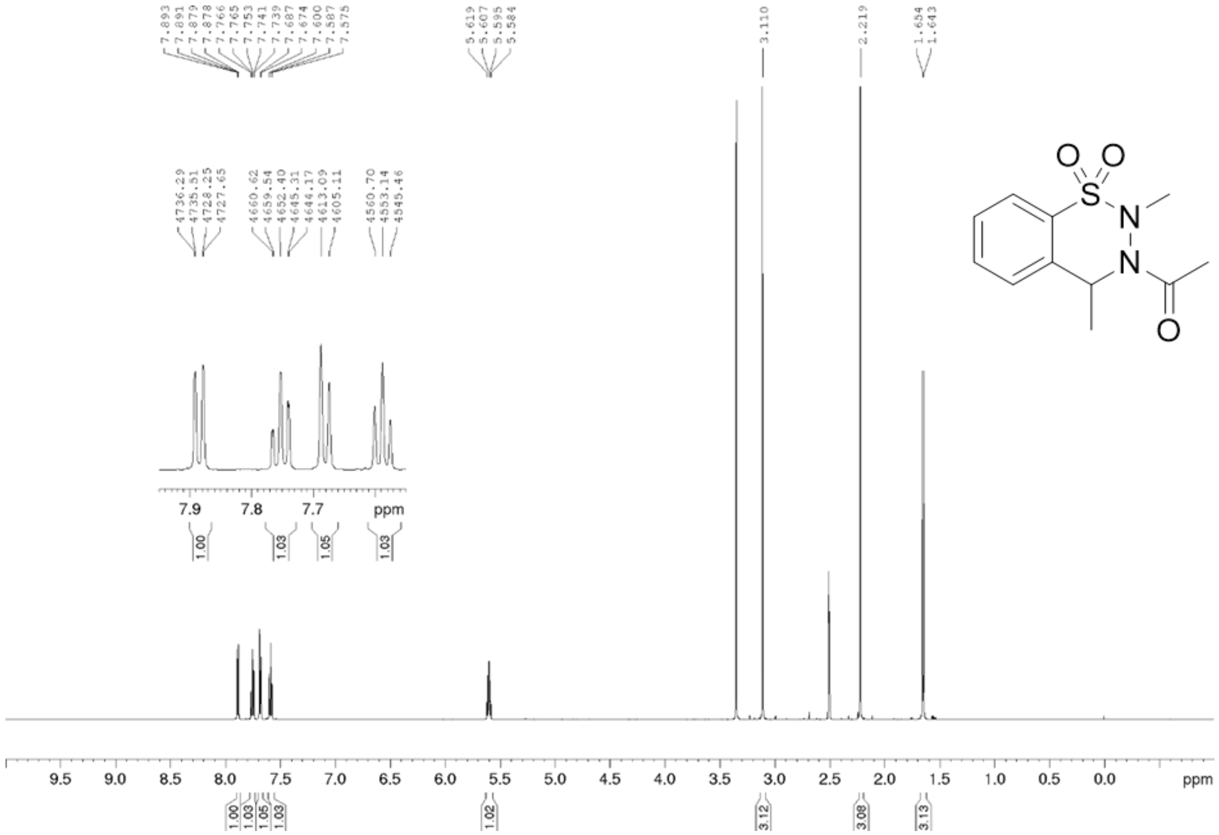

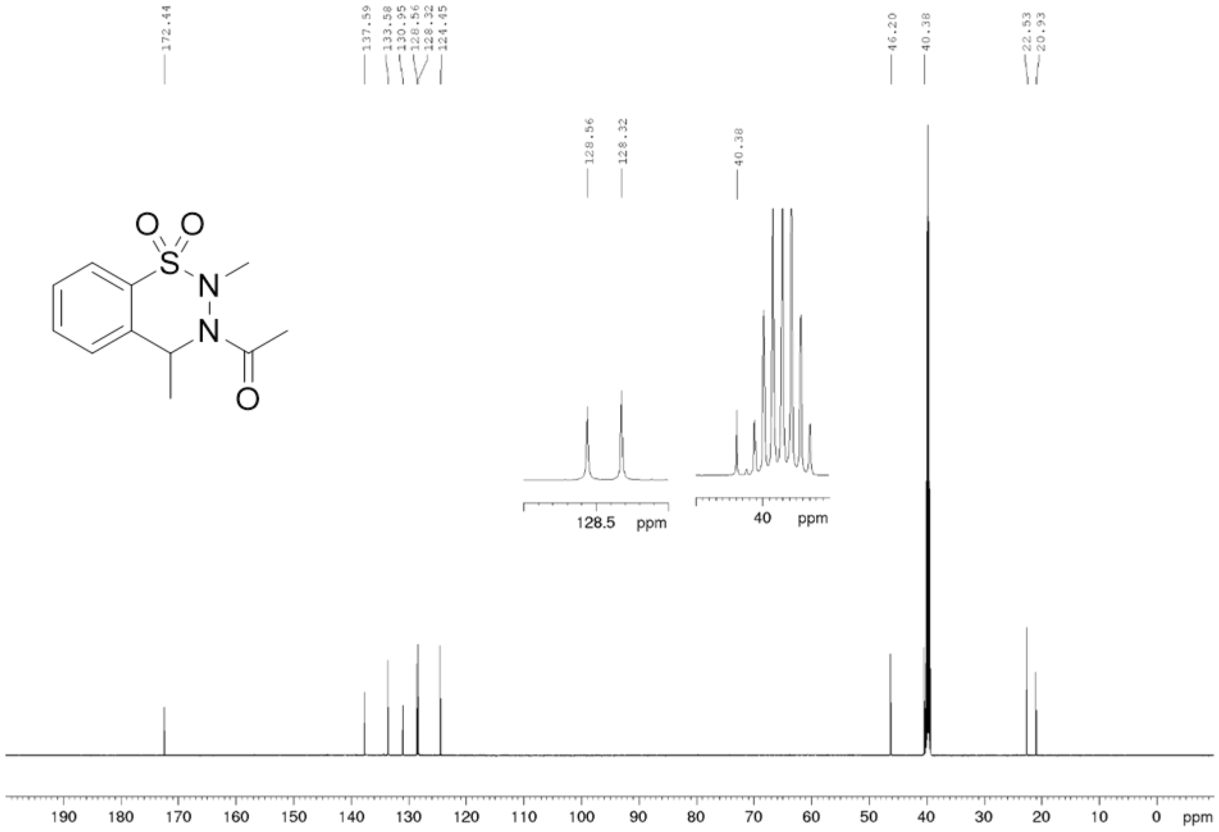

Compound **1e**, ^{1}H and ^{13}C NMR (600/150 MHz, [D_6]DMSO)

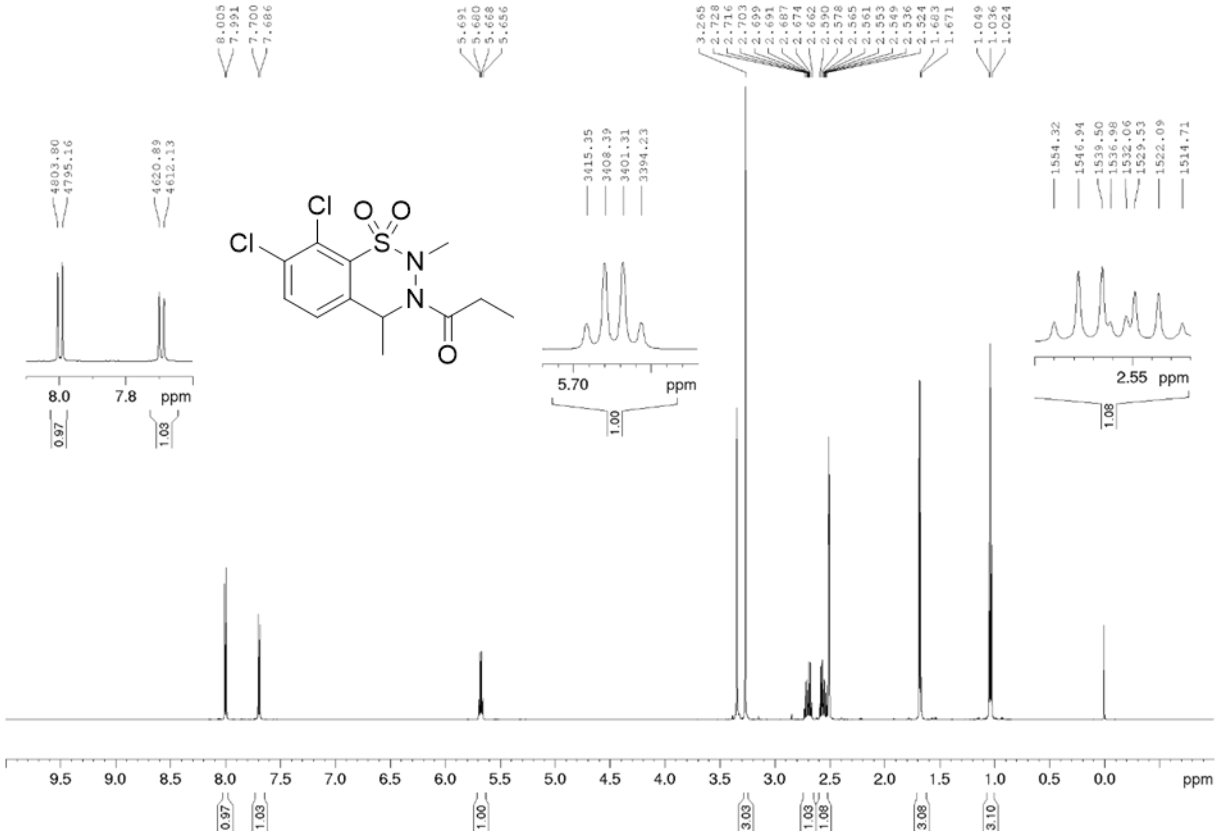

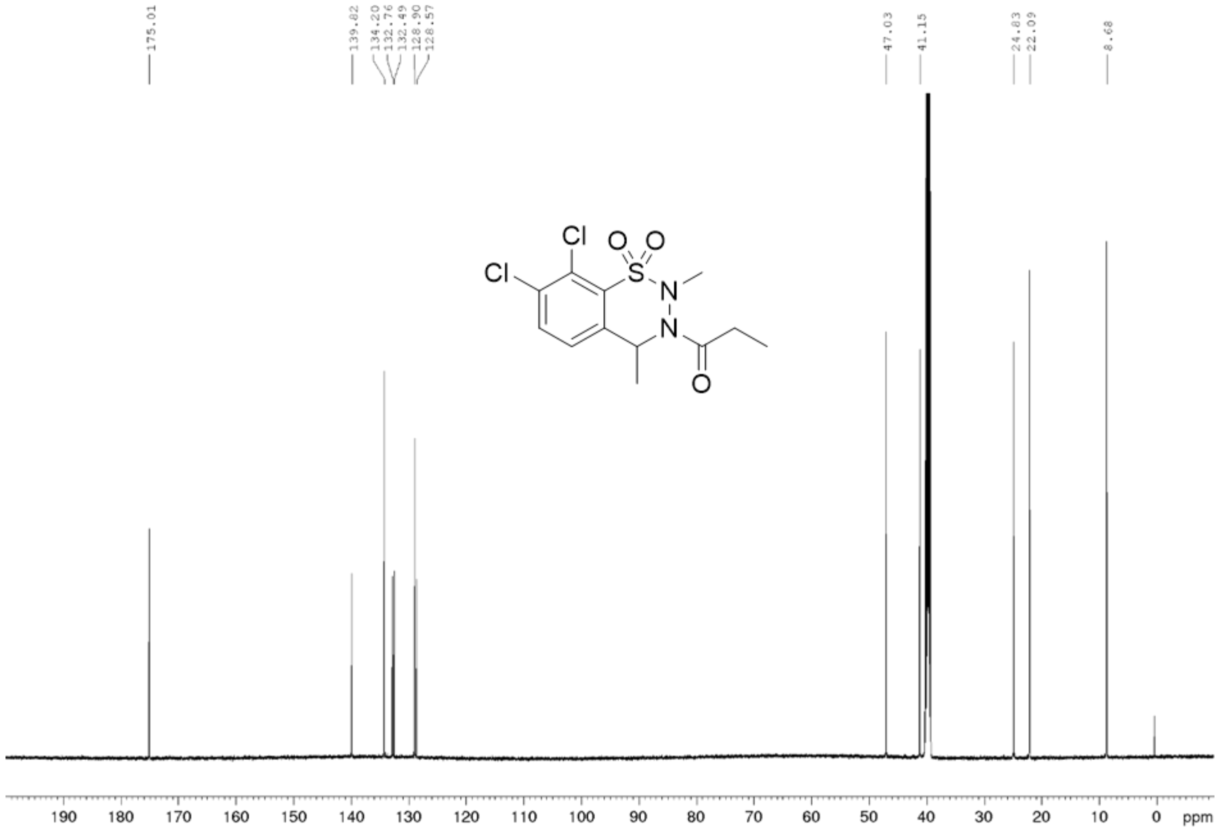

Compound **1f**, ^{1}H and ^{13}C NMR (600/150 MHz, [D$_6$]DMSO)

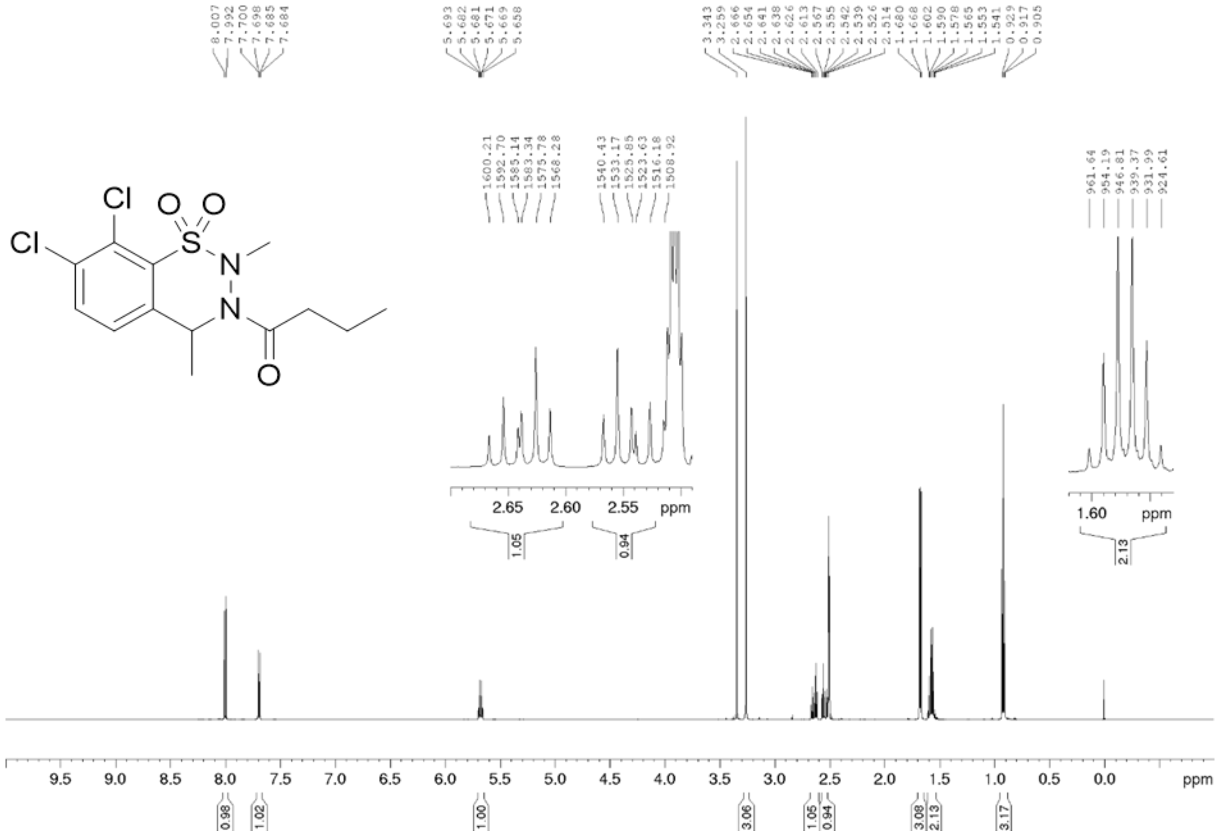

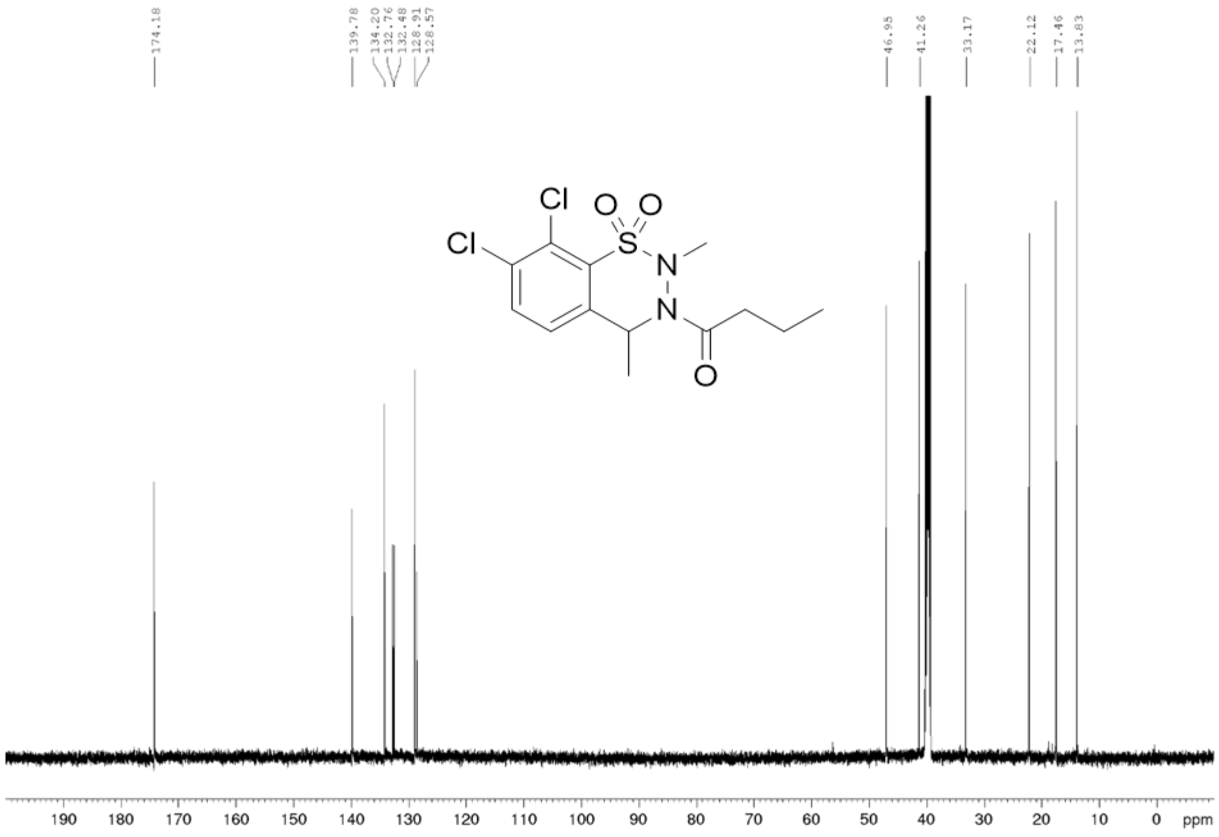

Compound **1g**, ^{1}H and ^{13}C NMR (600/150 MHz, $[D_6]DMSO$)

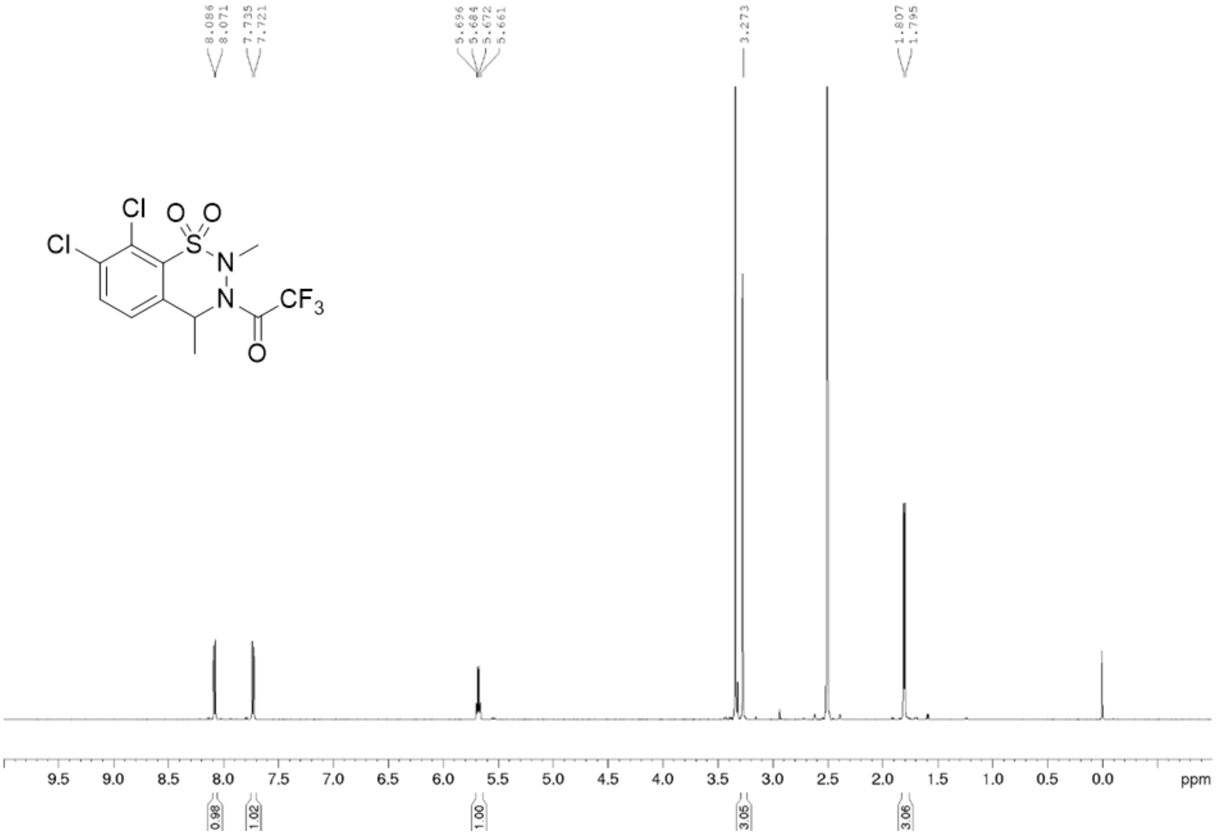

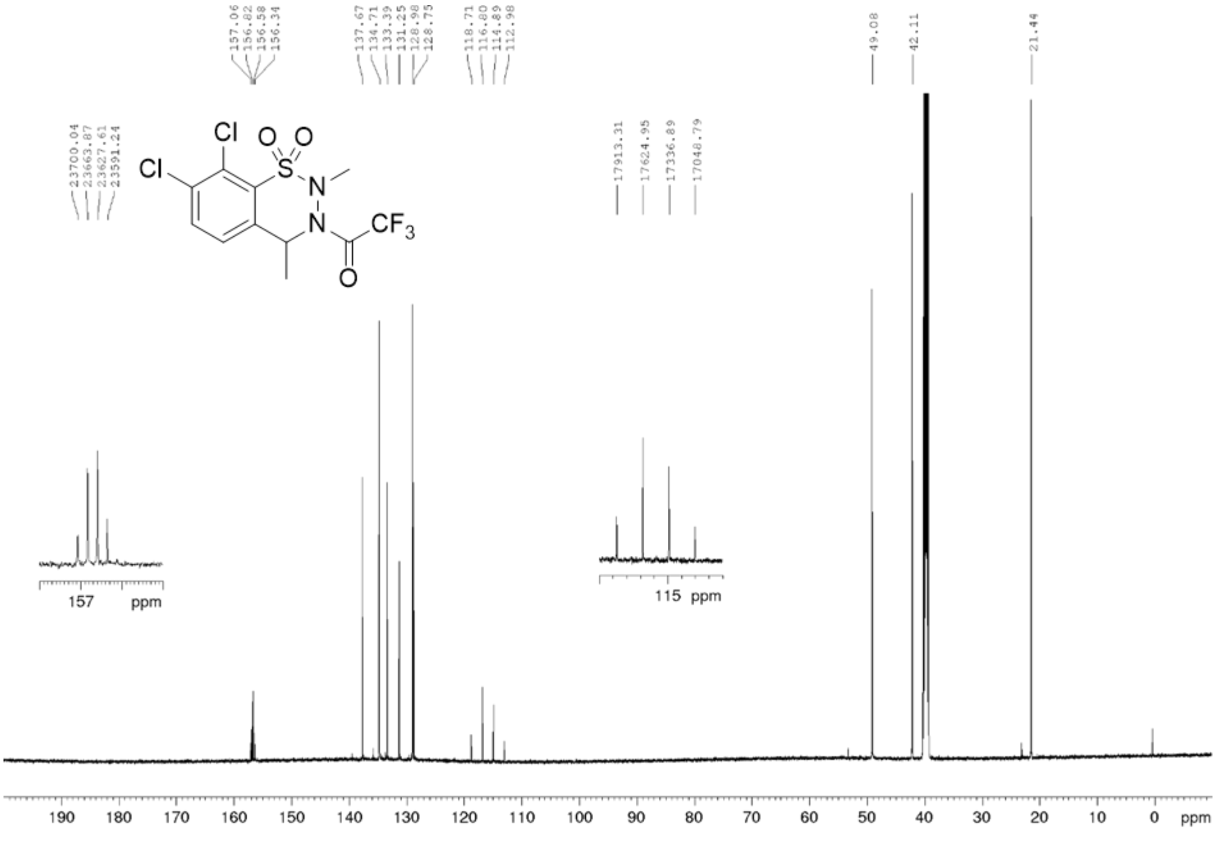

Compound **1h**, ^{1}H and ^{13}C NMR (600/150 MHz, $CDCl_3$)

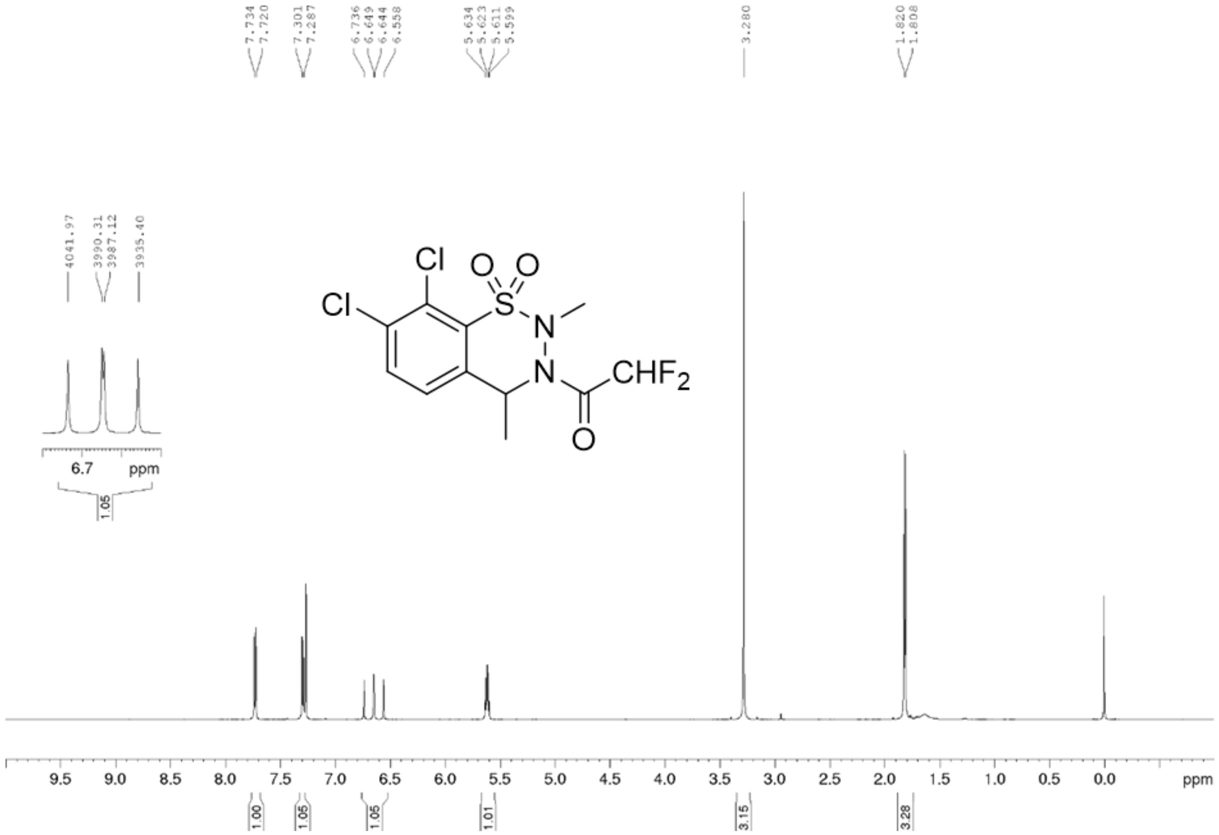

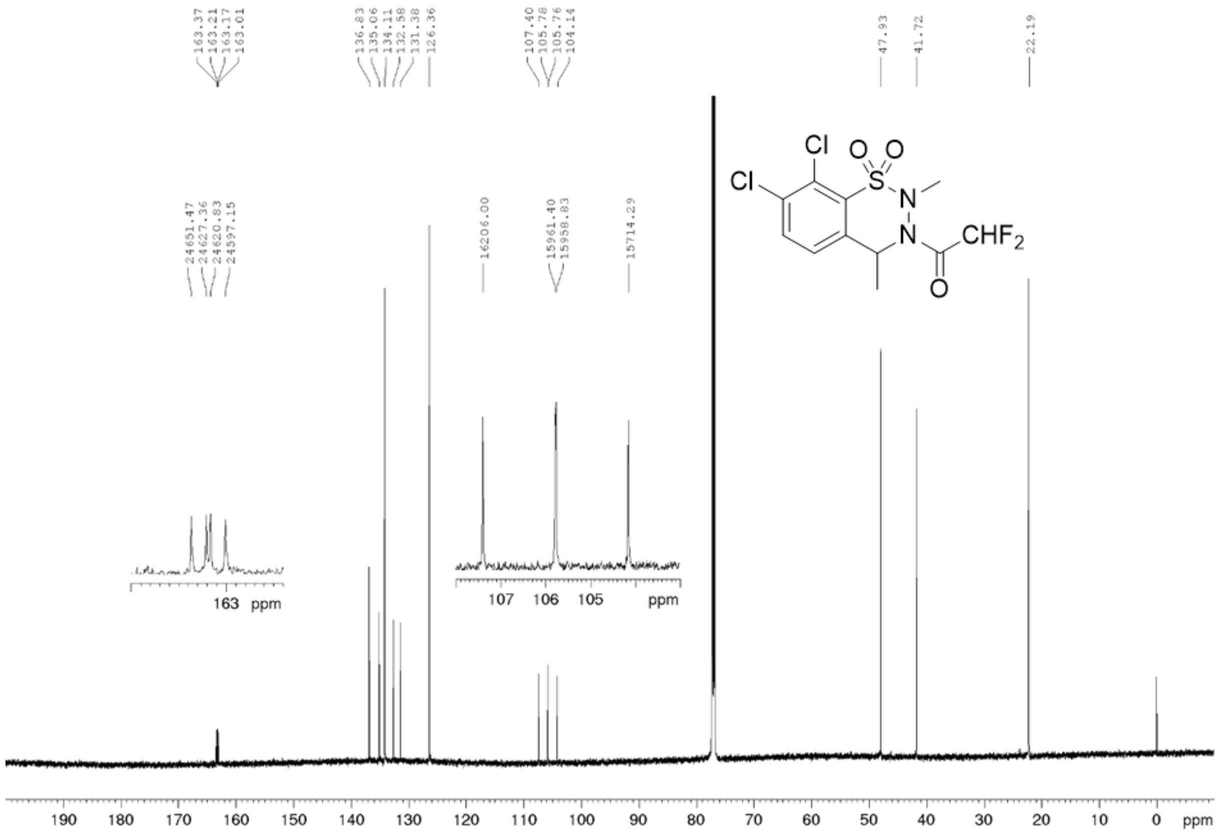

Compound **1p**, ^{1}H and ^{13}C NMR (600/150 MHz, $CDCl_3$)

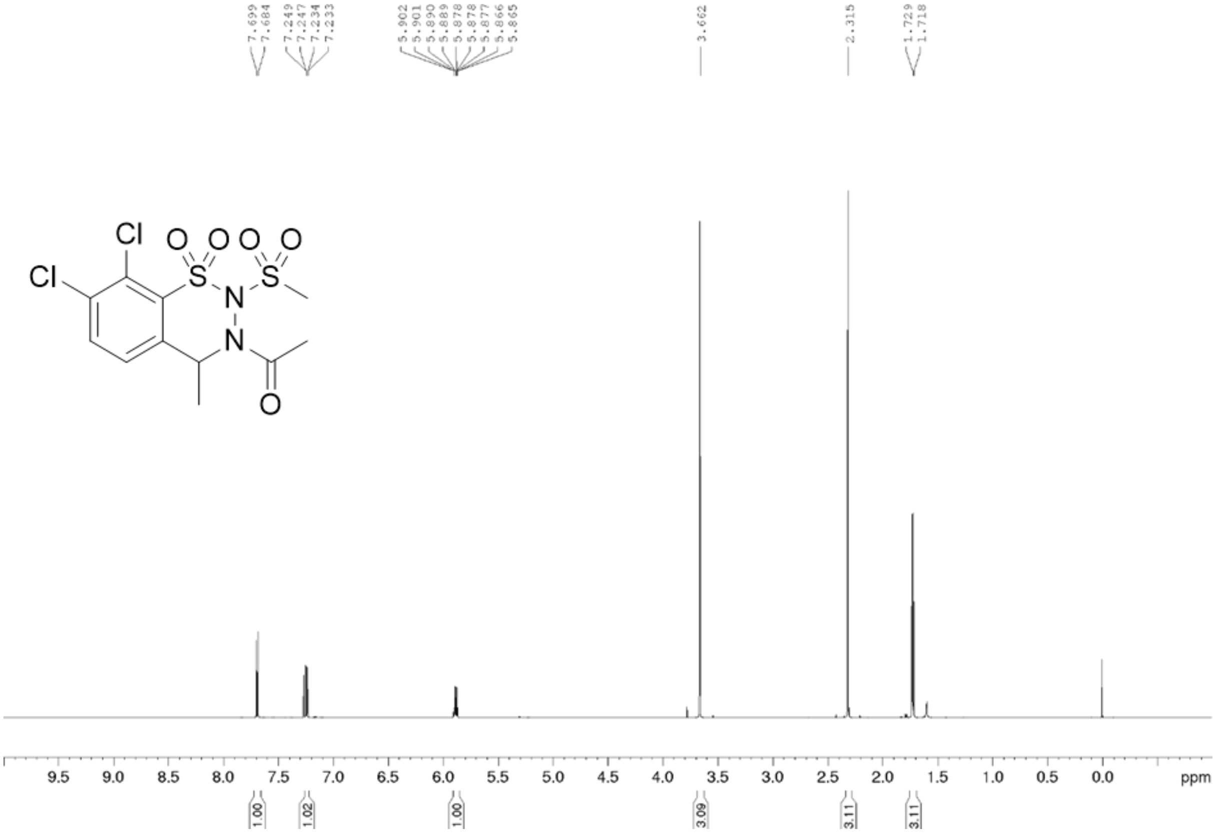

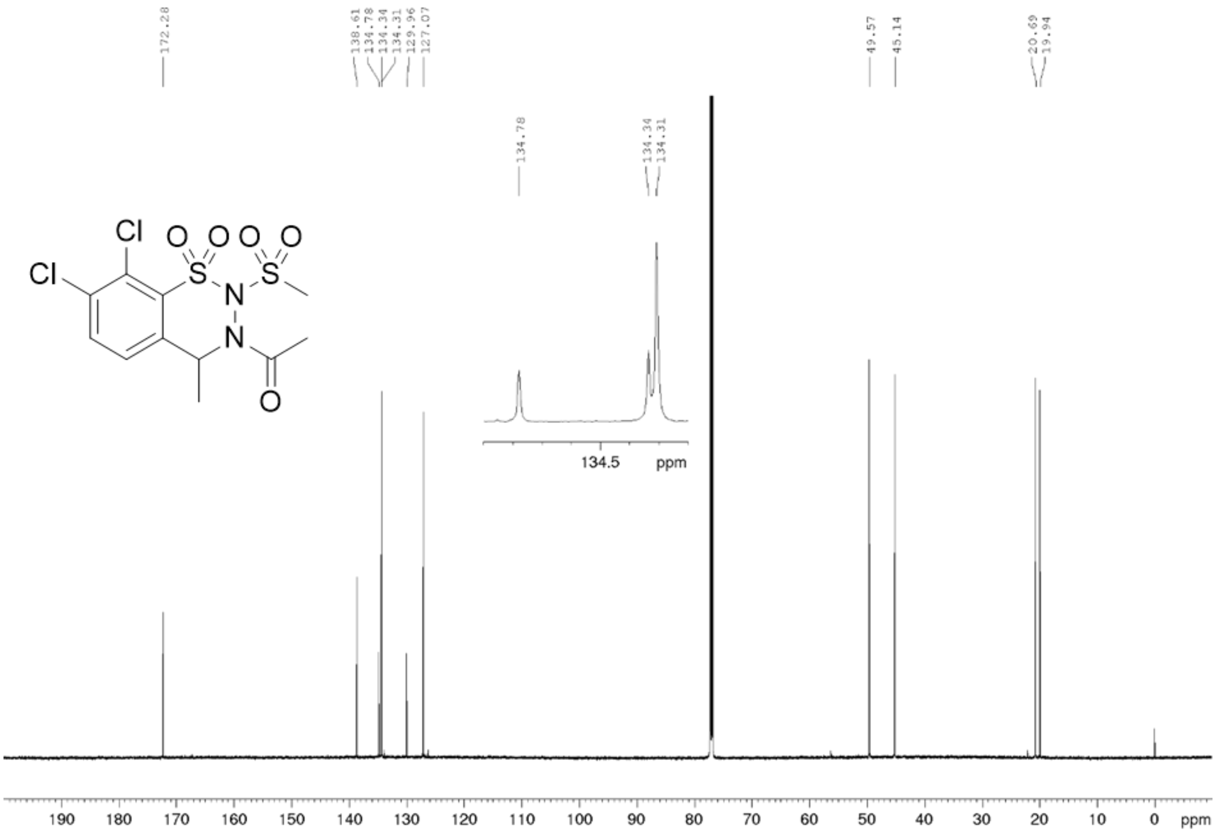

Compound **1q**, ^{1}H and ^{13}C NMR (600/150 MHz, $CDCl_3$)

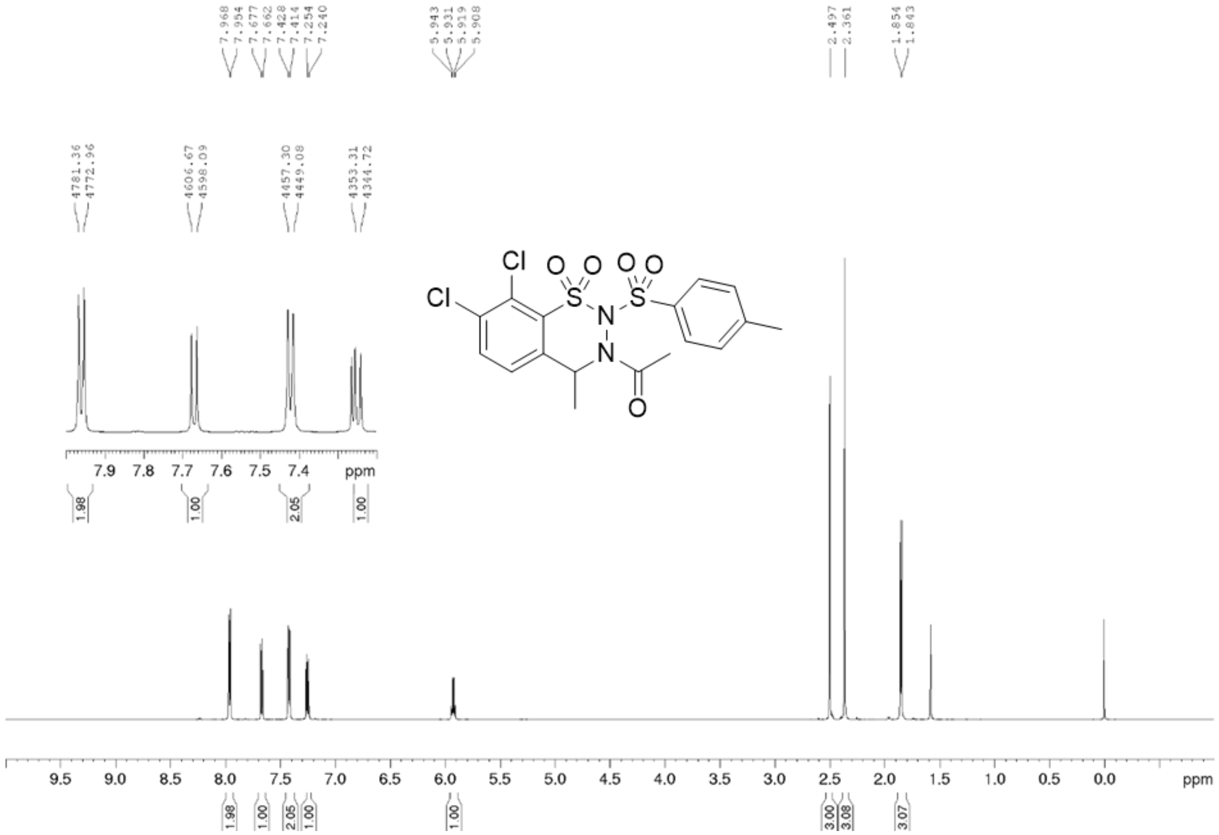

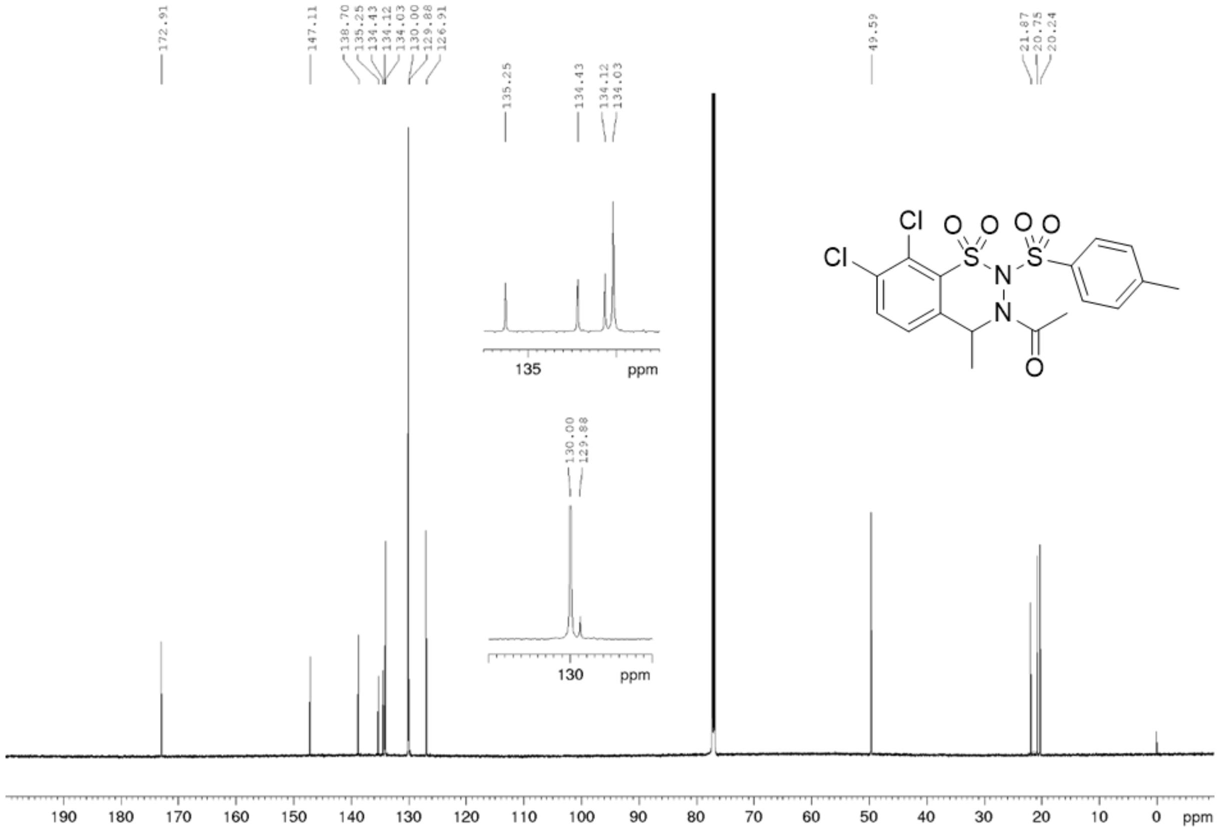

Compound **2a**, ^{1}H and ^{13}C NMR (600/150 MHz, [D$_6$]DMSO)

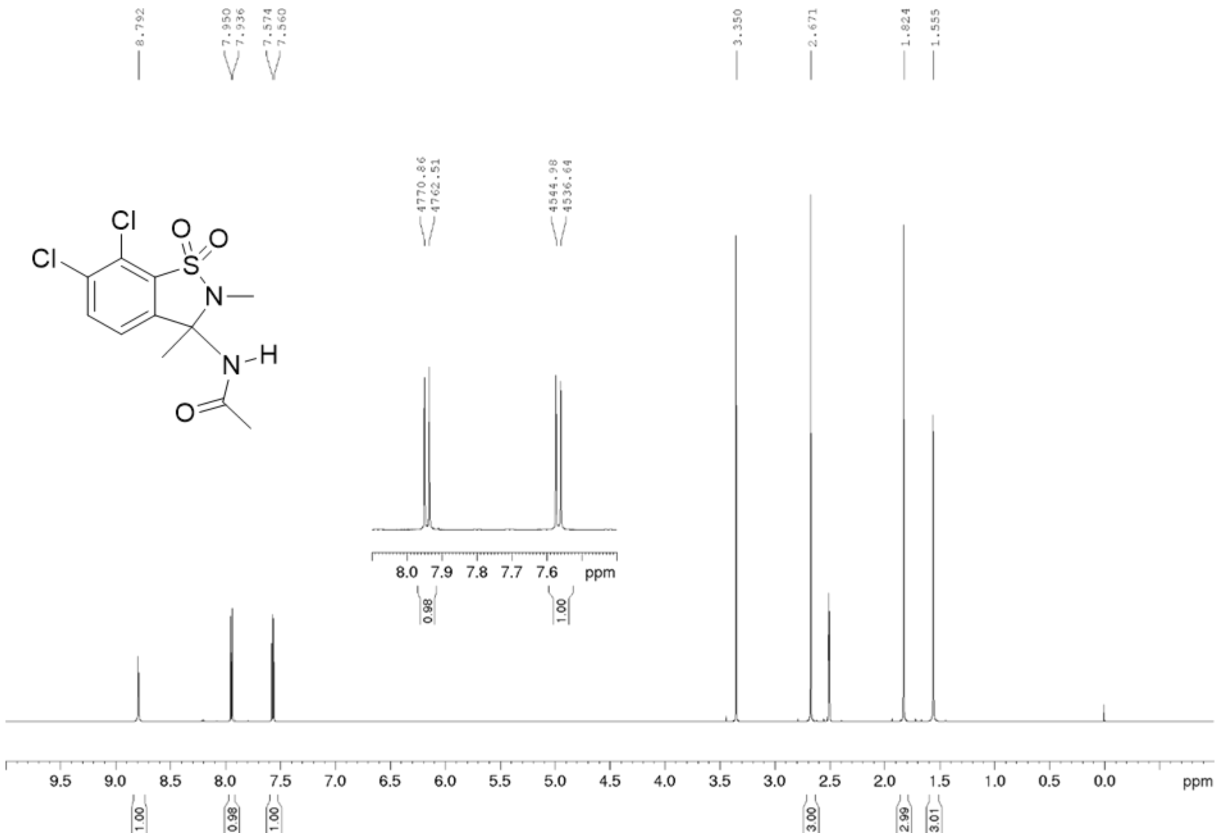

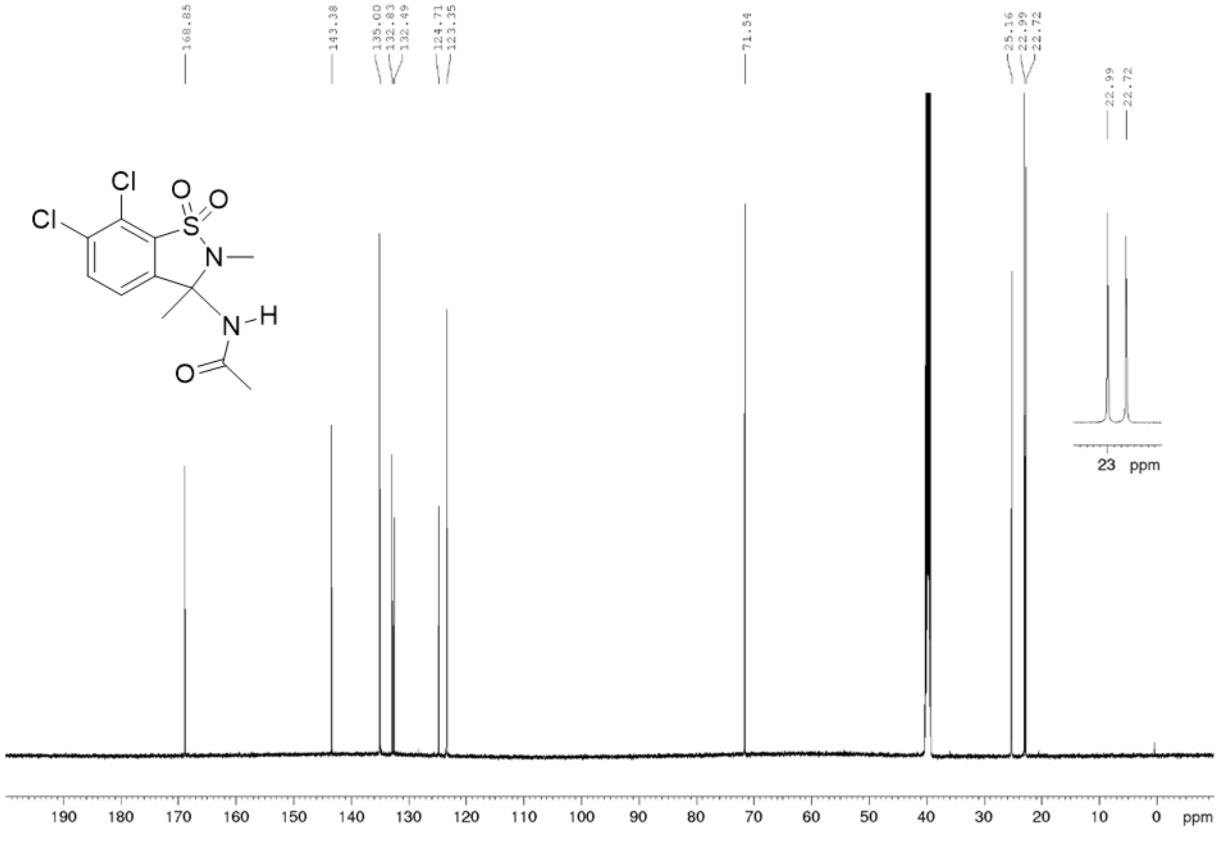

Compound **2b**, ^{1}H and ^{13}C NMR (600/150 MHz, [D_6]DMSO)

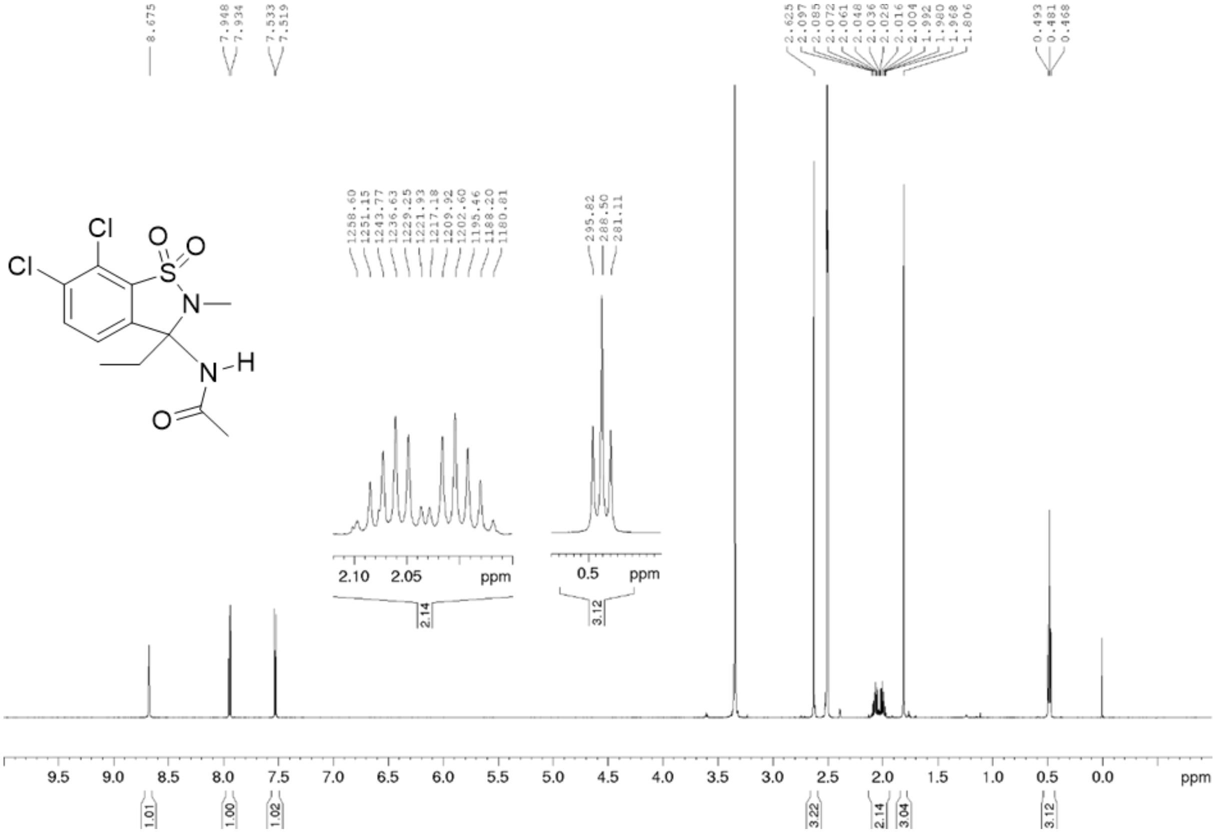

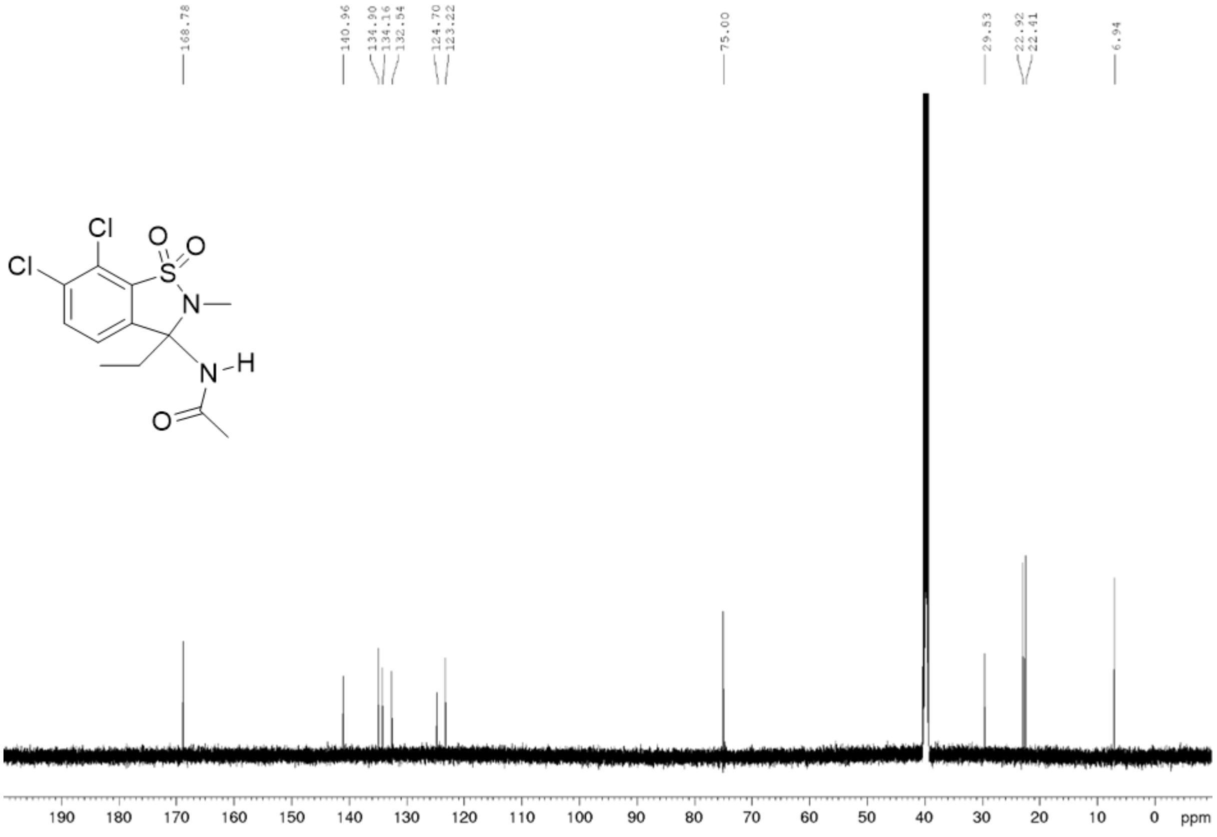

Compound **2c**, ^{1}H NMR and DEPTQ (600/150 MHz, [D$_6$]DMSO)

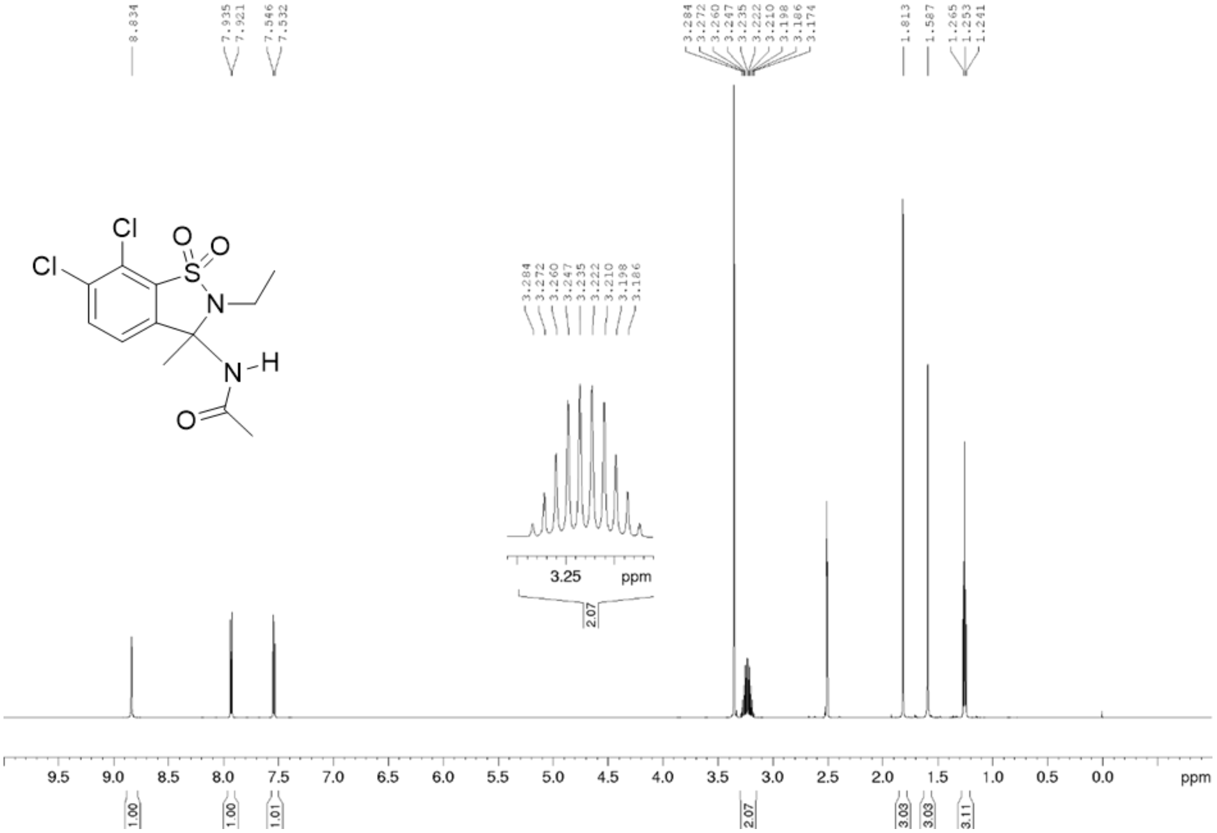

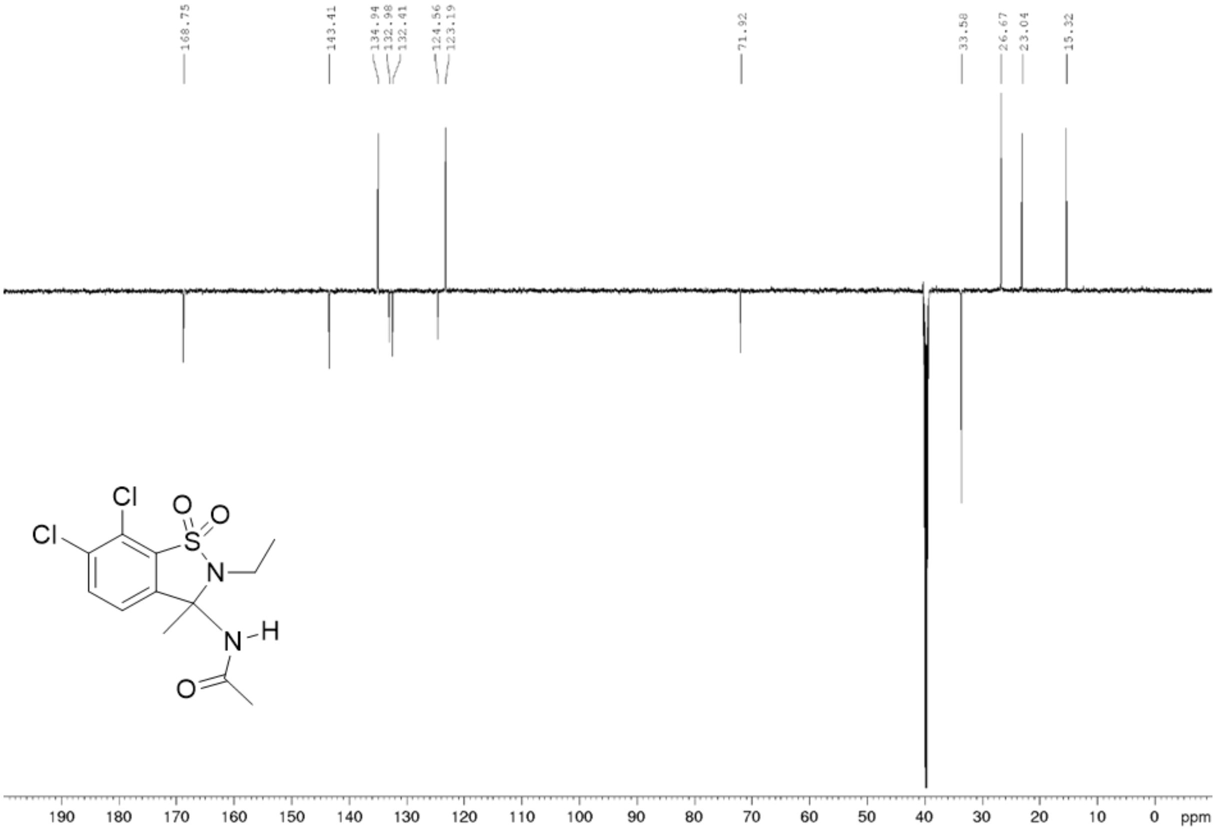

Compound **2d**, ^{1}H and ^{13}C NMR (600/150 MHz, [D_6]DMSO)

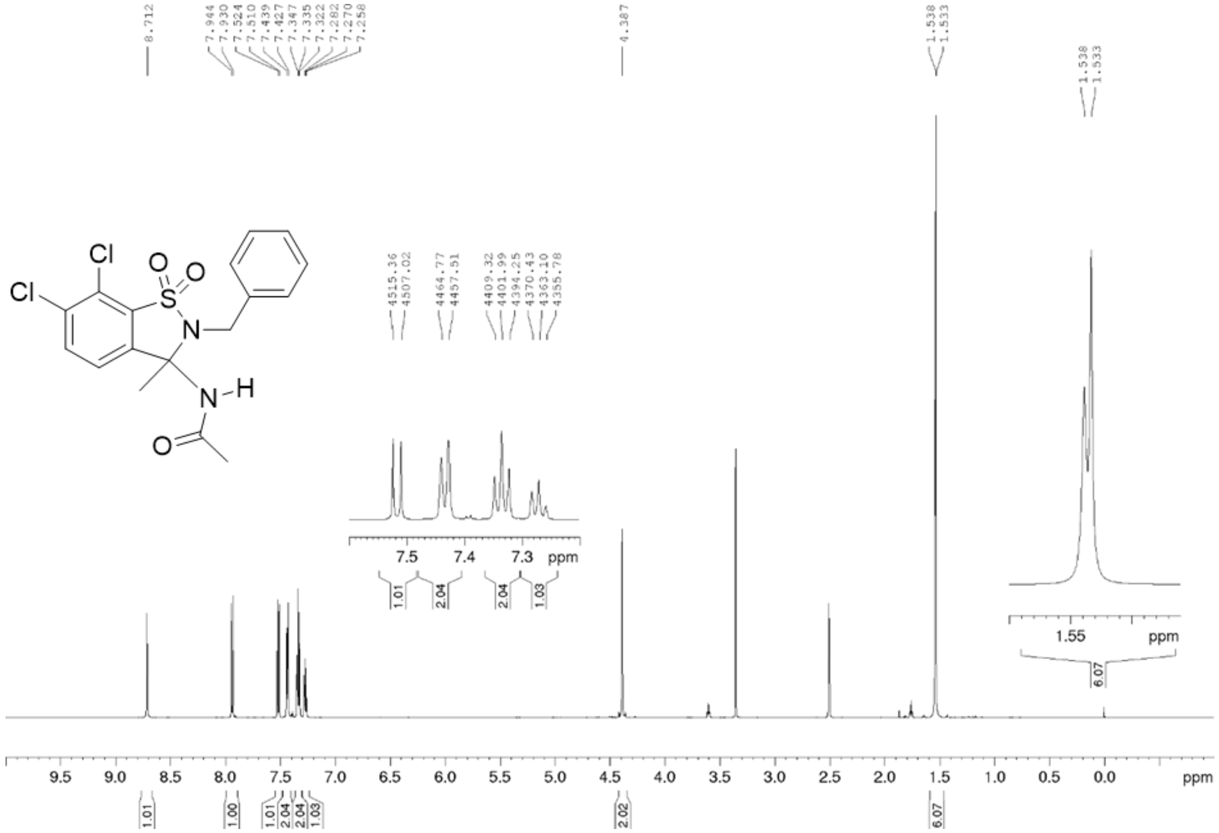

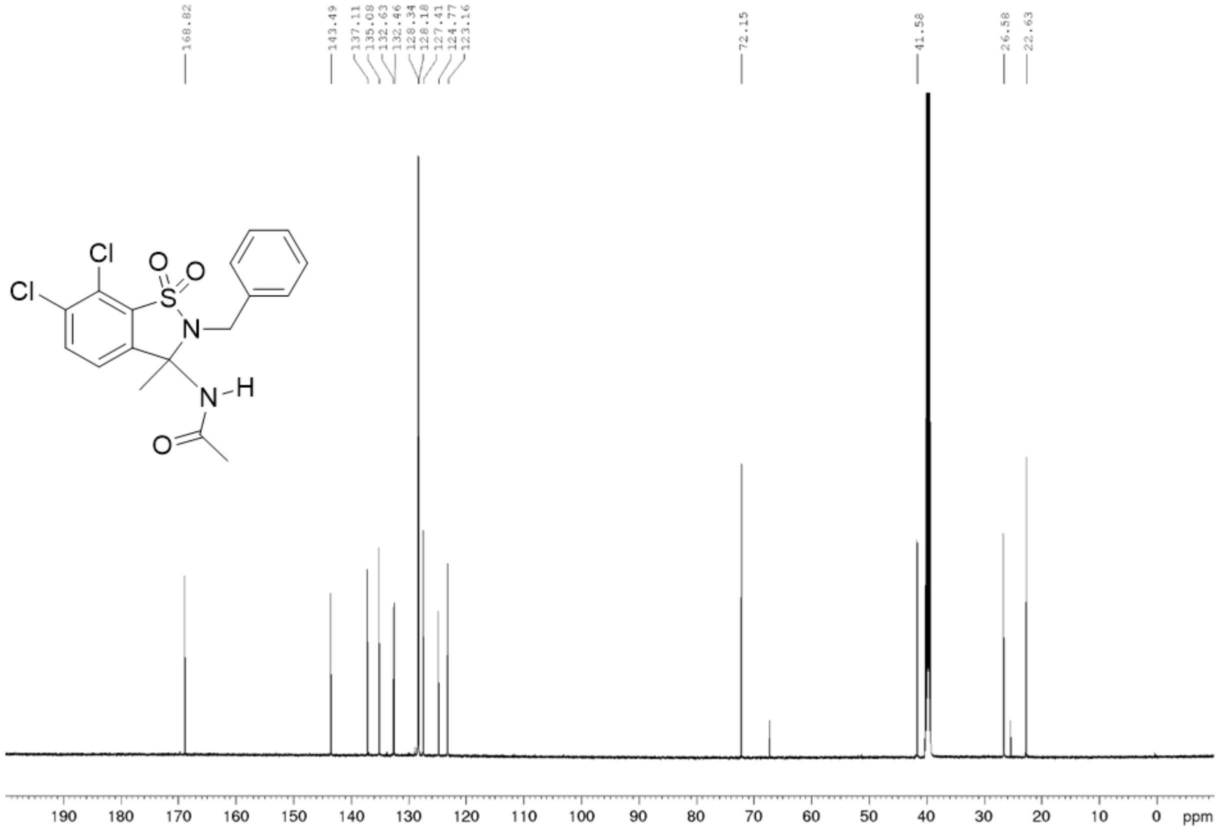

Compound **2e**, ^{1}H and ^{13}C NMR (600/150 MHz, [D_6]DMSO)

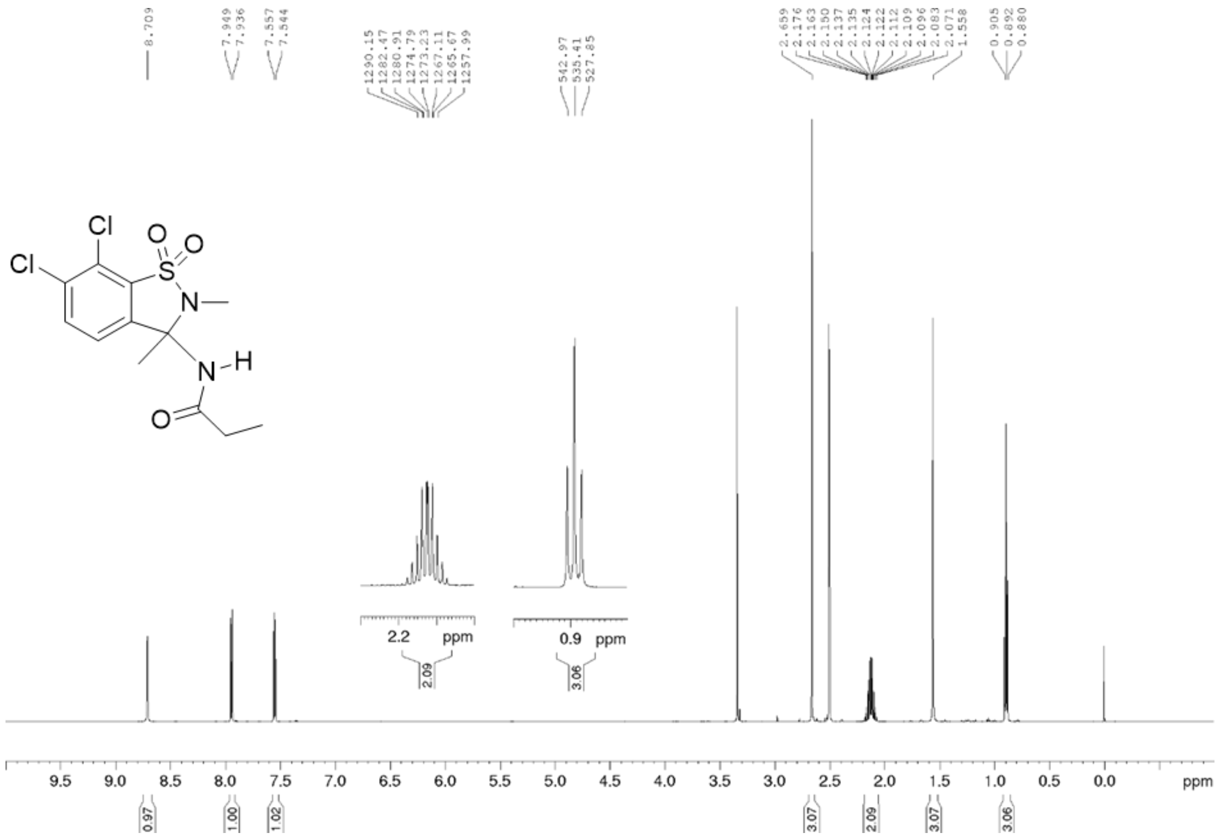

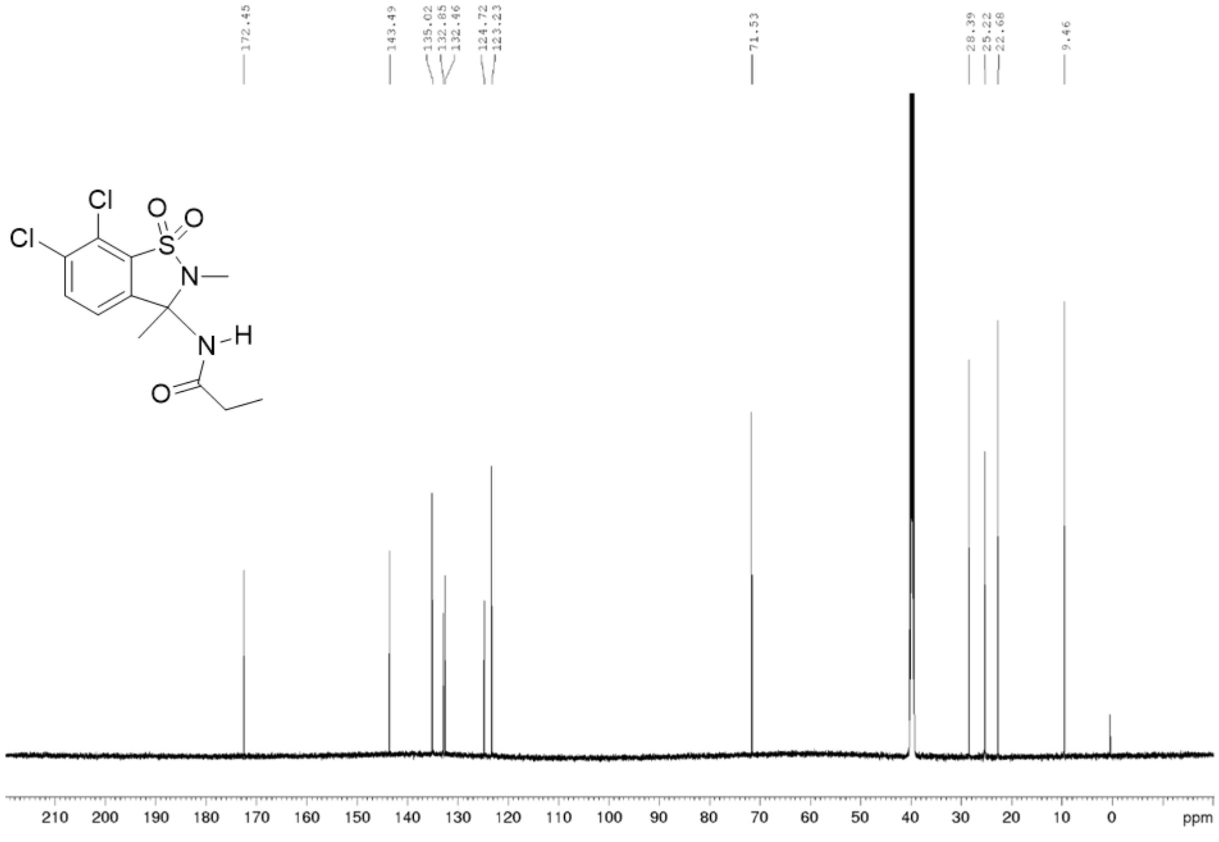

Compound **2f**, ^{1}H and ^{13}C NMR (600/150 MHz, [D$_6$]DMSO)

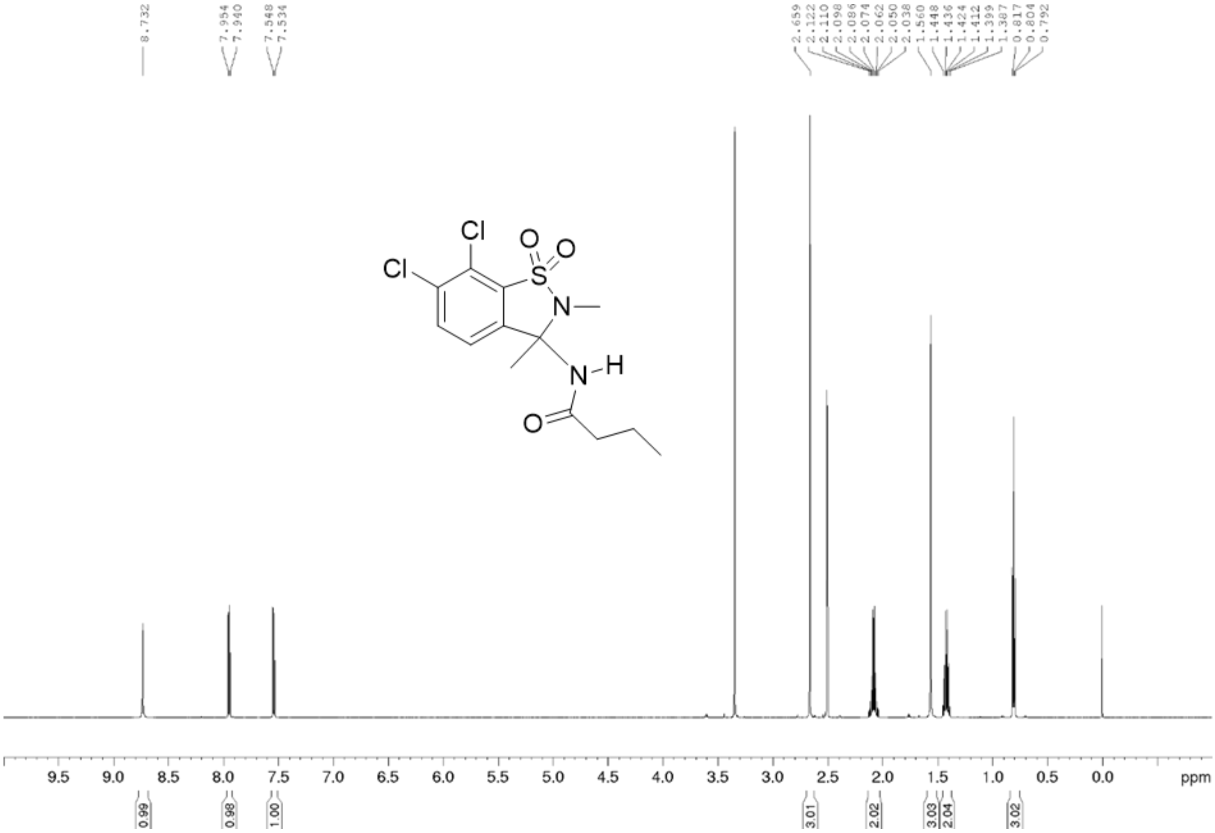

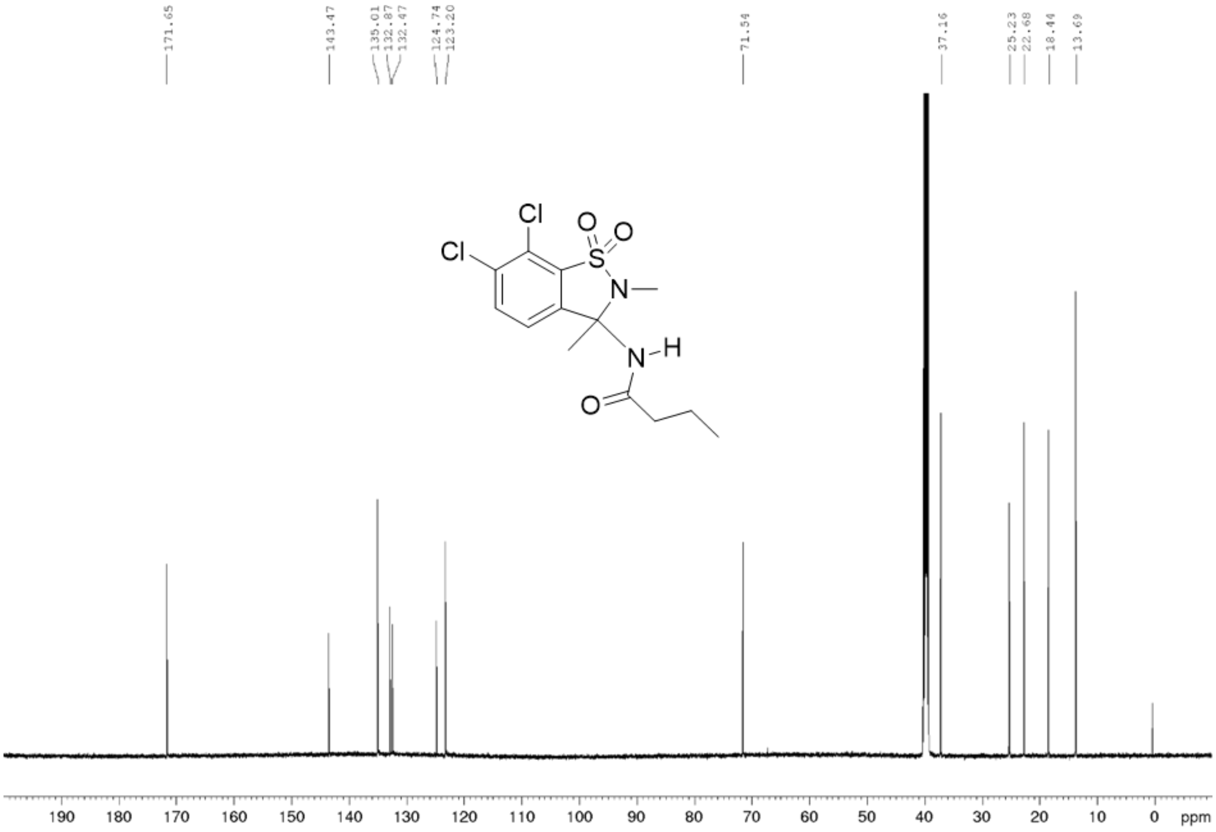

Compound **2g**, ^{1}H and ^{13}C NMR (600/150 MHz [D$_6$]DMSO)

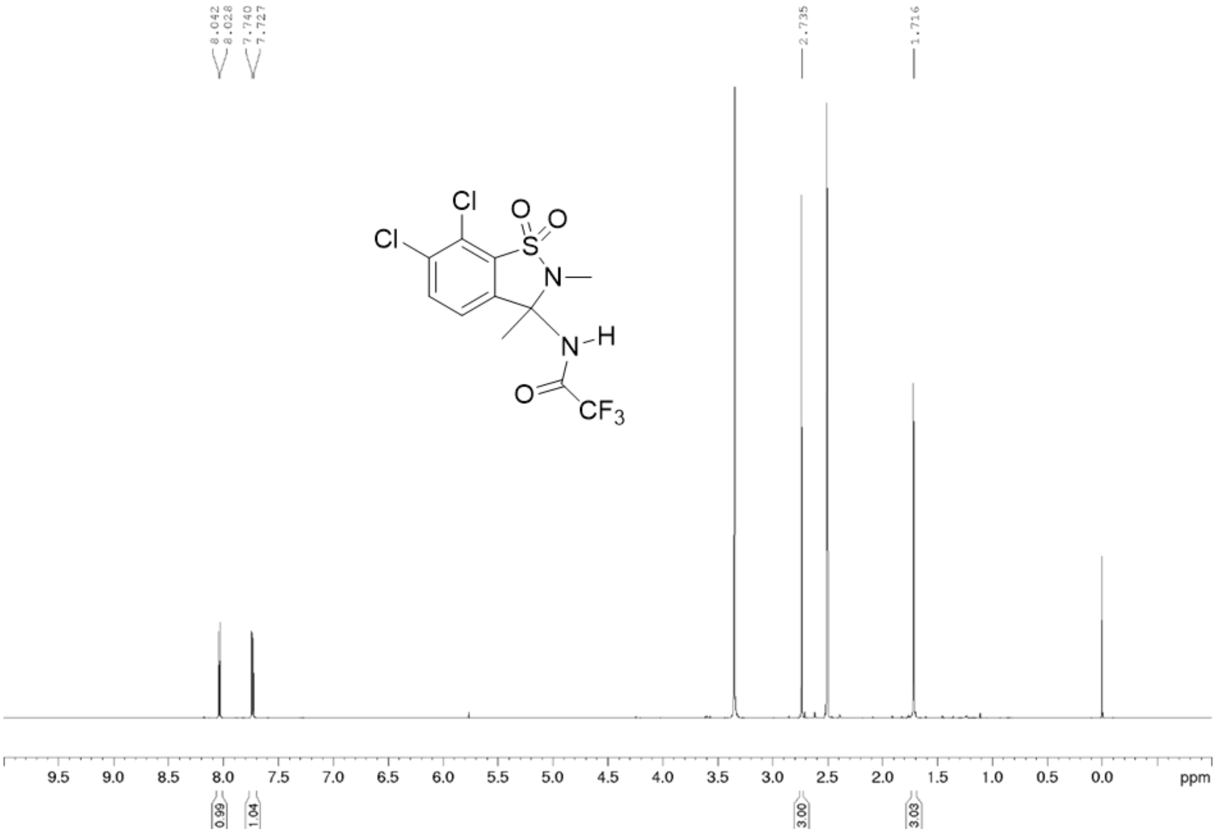

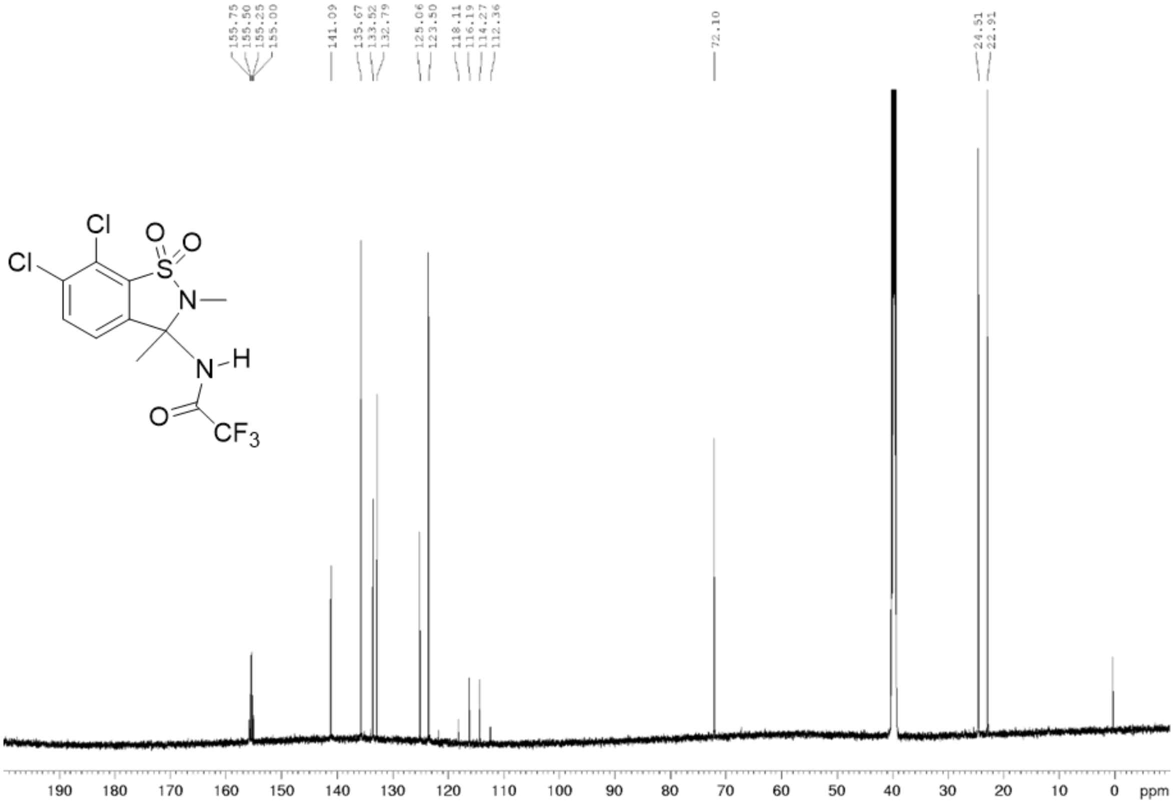

Compound **2h**, ^{1}H and ^{13}C NMR (600/150 MHz [D$_6$]DMSO)

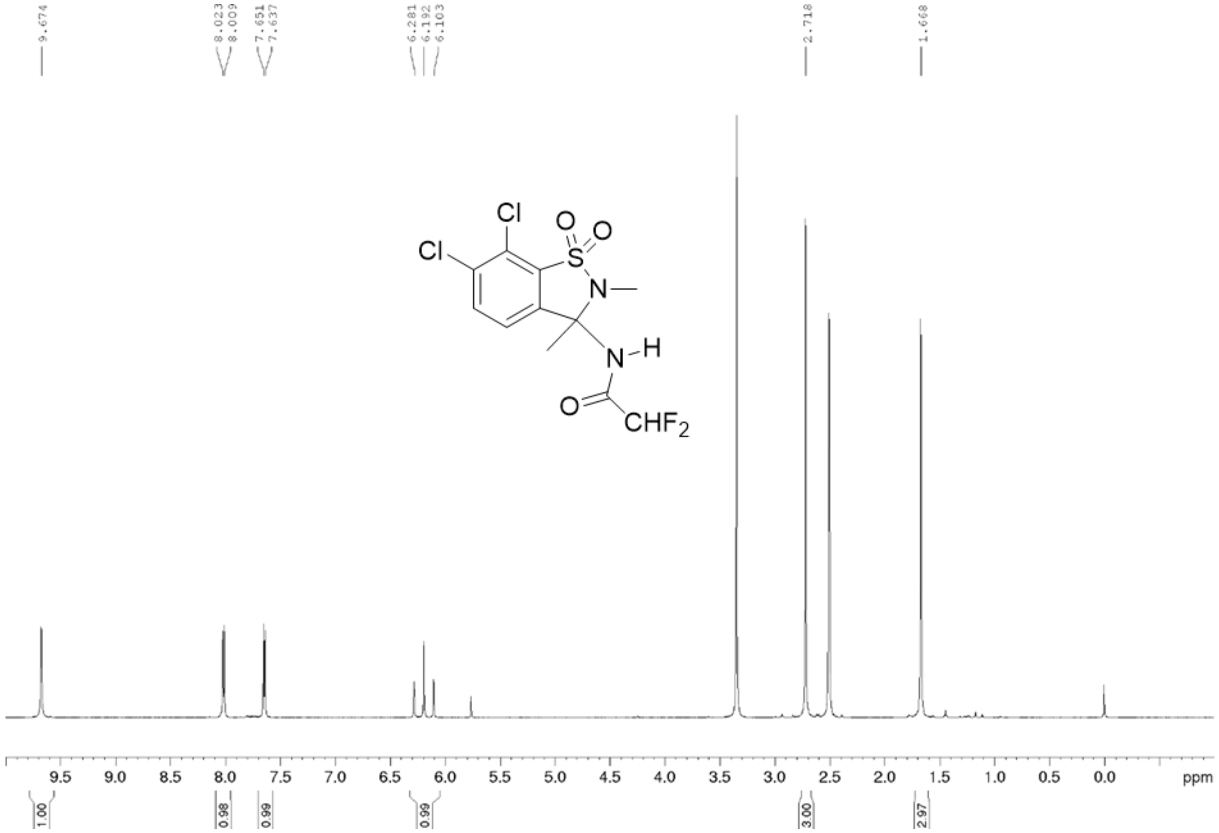

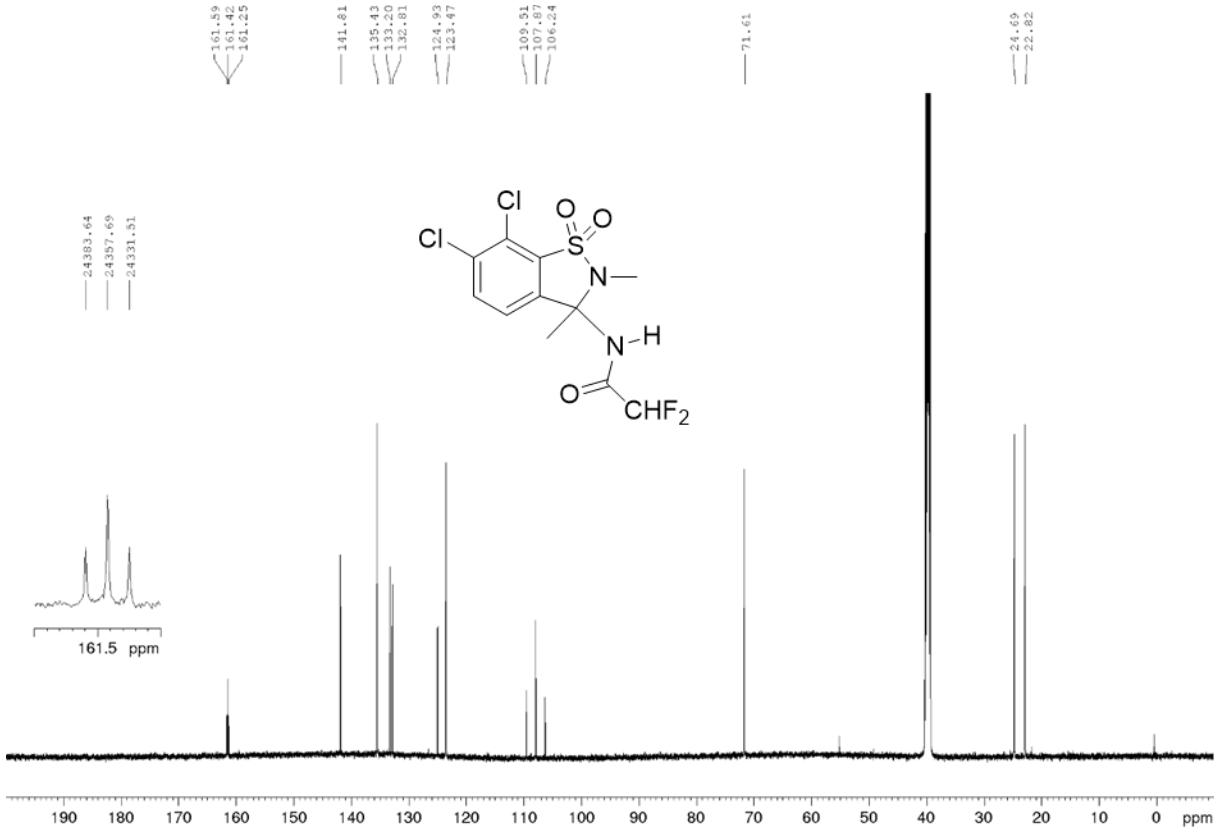

Compound **2i**, ^{1}H and ^{13}C NMR (600/150 MHz, [D_6]DMSO)

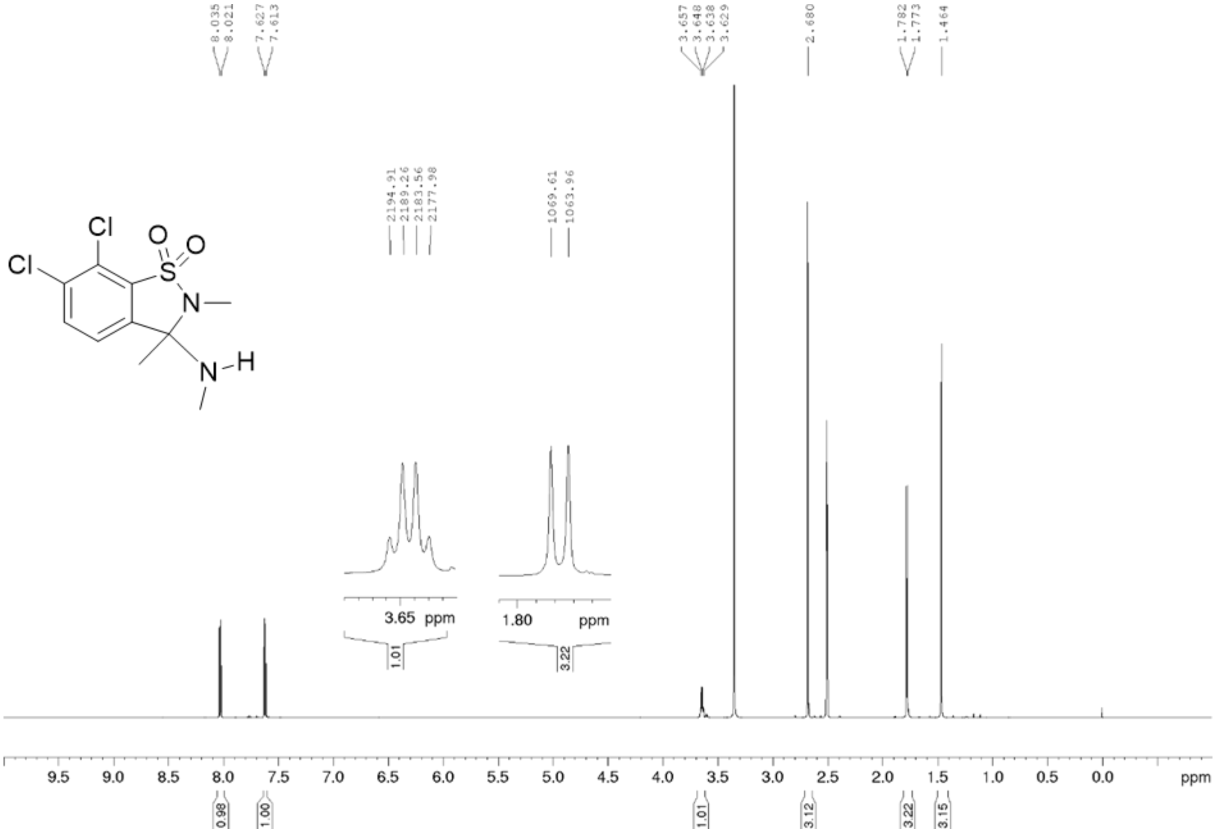

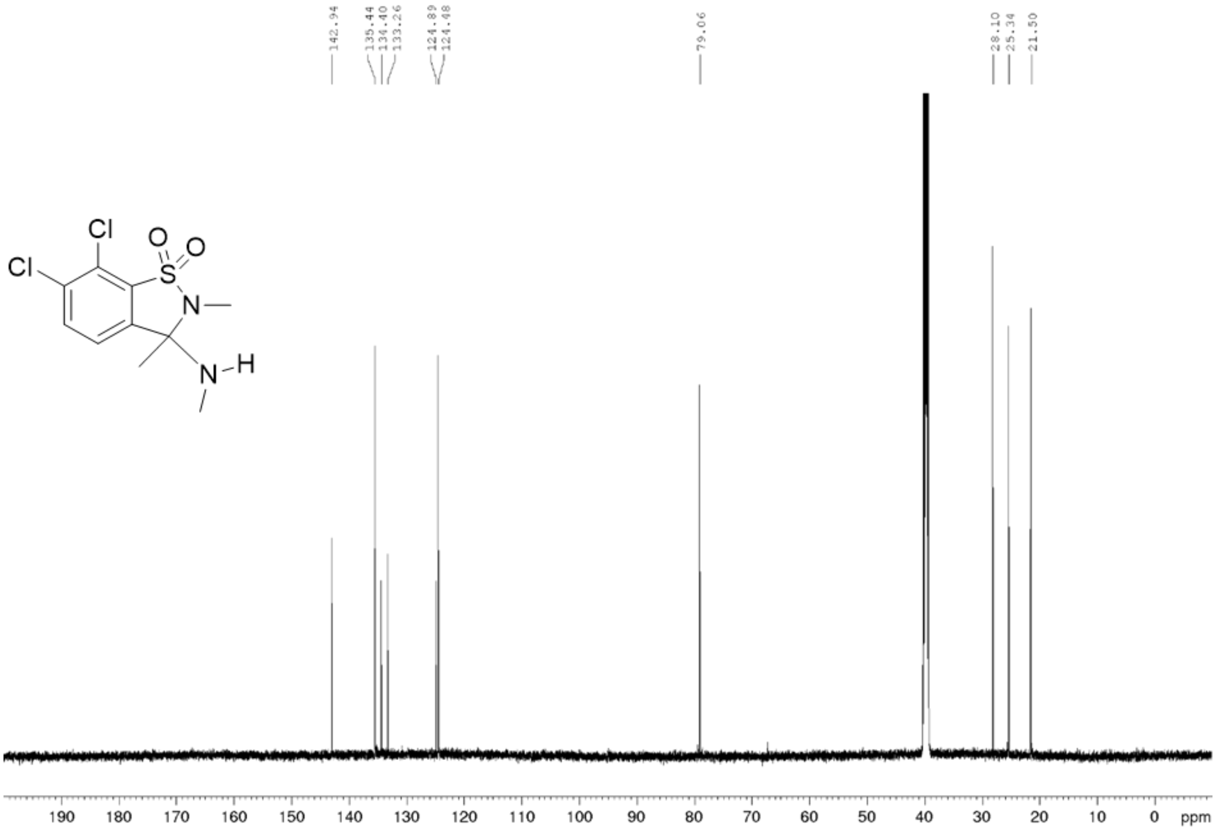

Compound **2j**, ^{1}H and ^{13}C NMR (600/150 MHz, [D$_6$]DMSO)

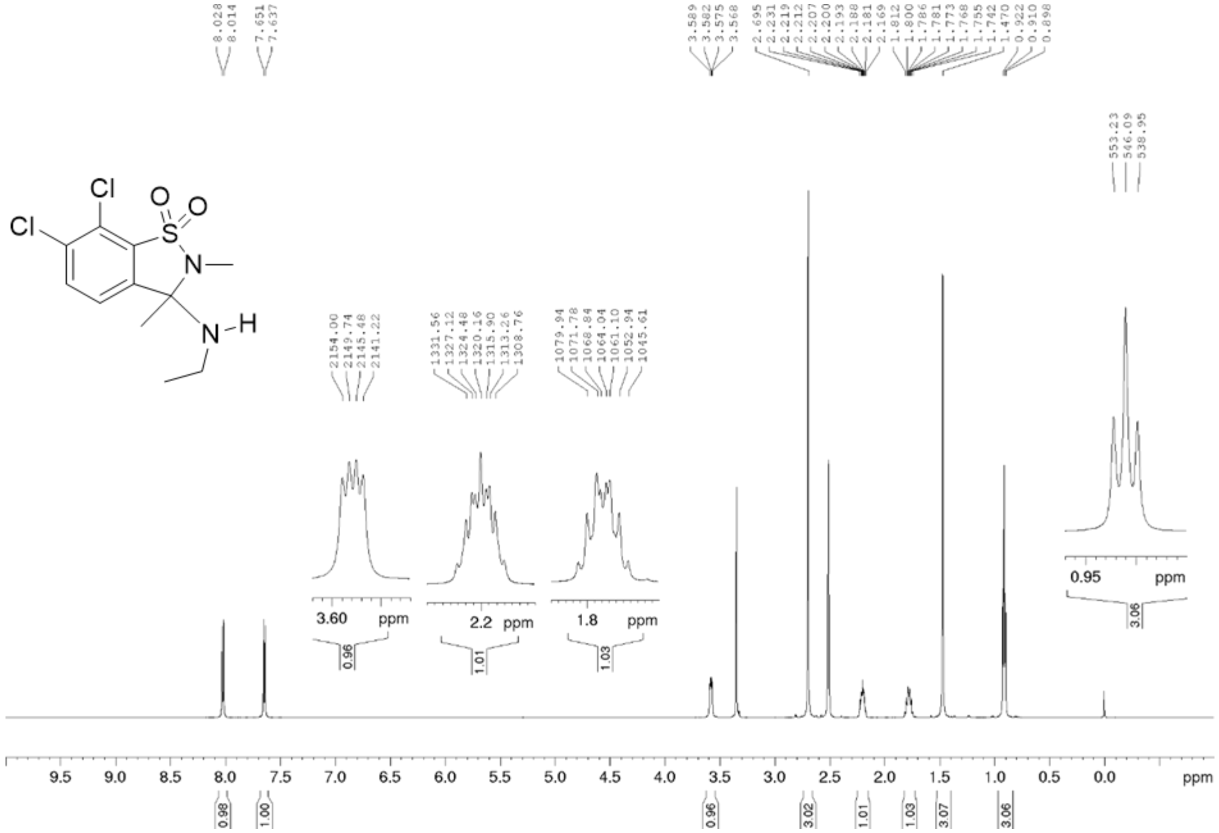

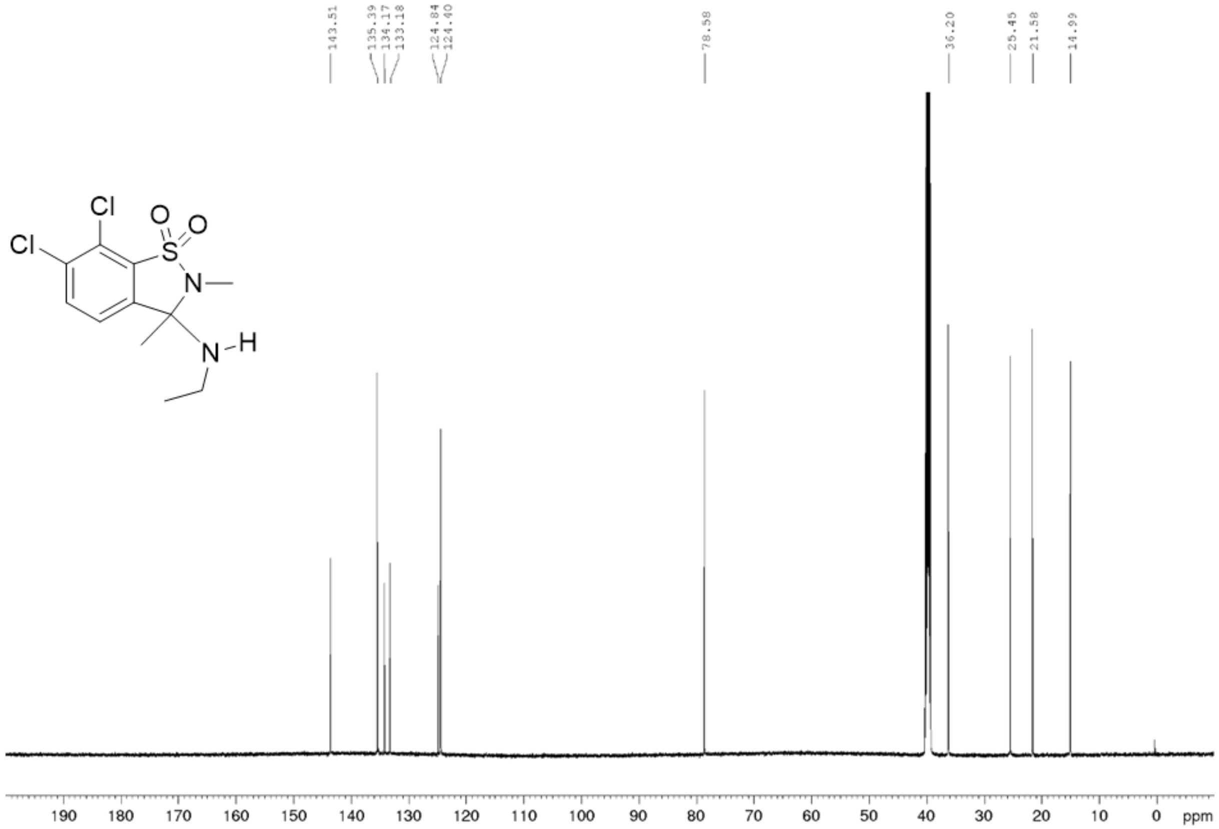

Compound **2k**, ^{1}H and ^{13}C NMR (600/150 MHz, [D_6]DMSO)

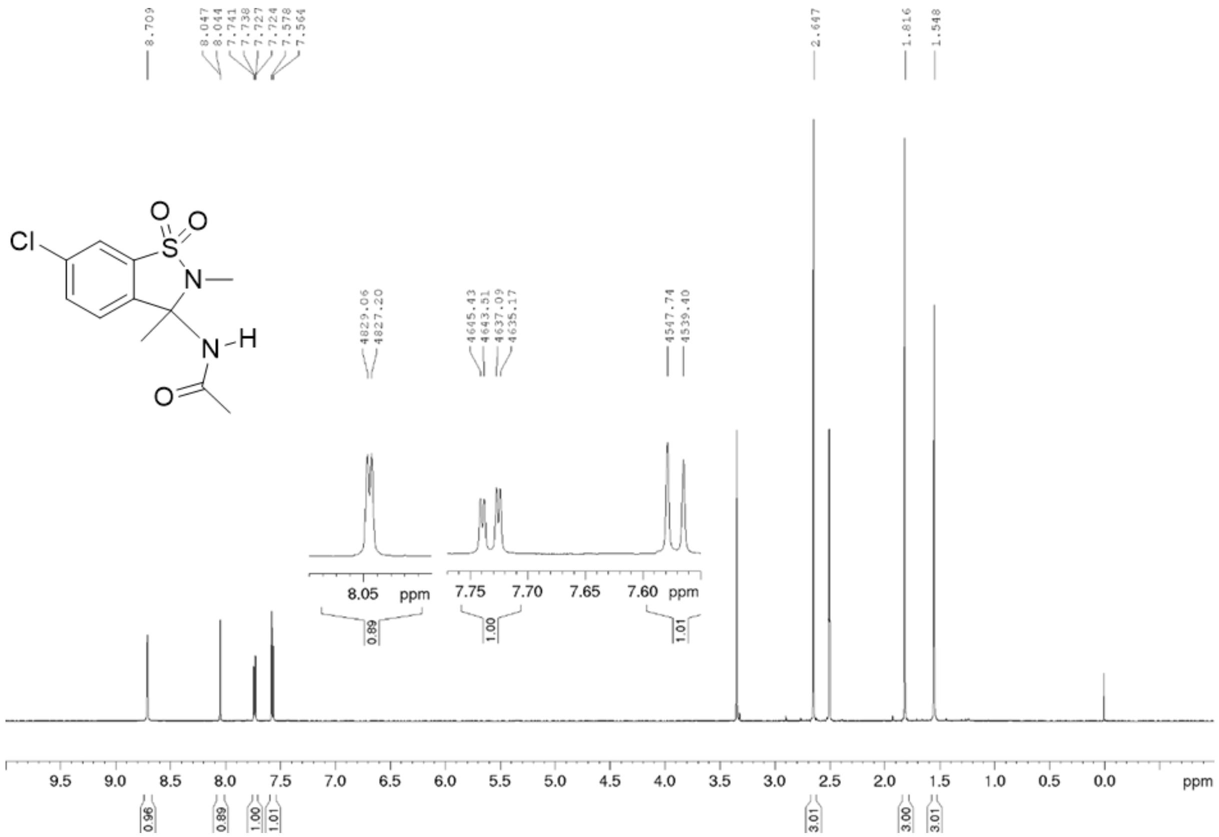

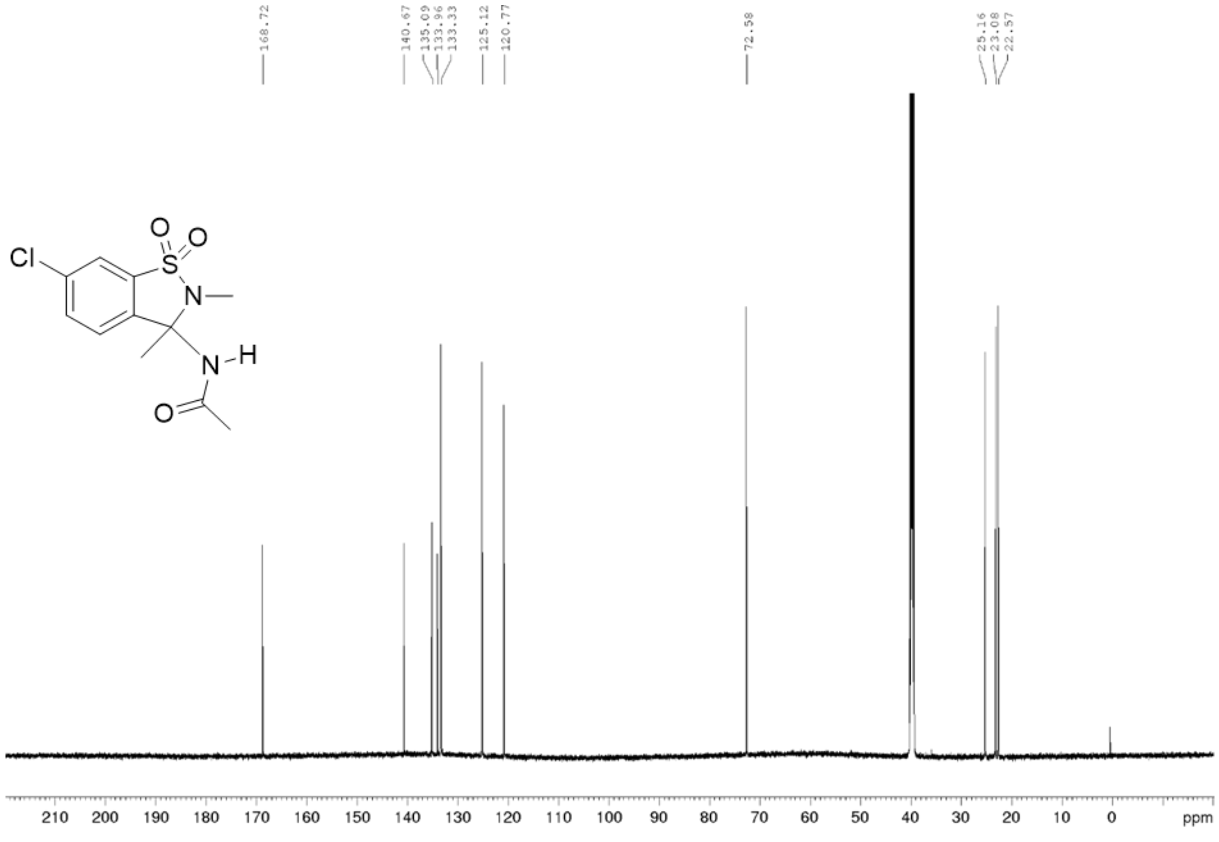

Compound **2l**, ^{1}H and ^{13}C NMR (600/150 MHz, [D$_6$]DMSO)

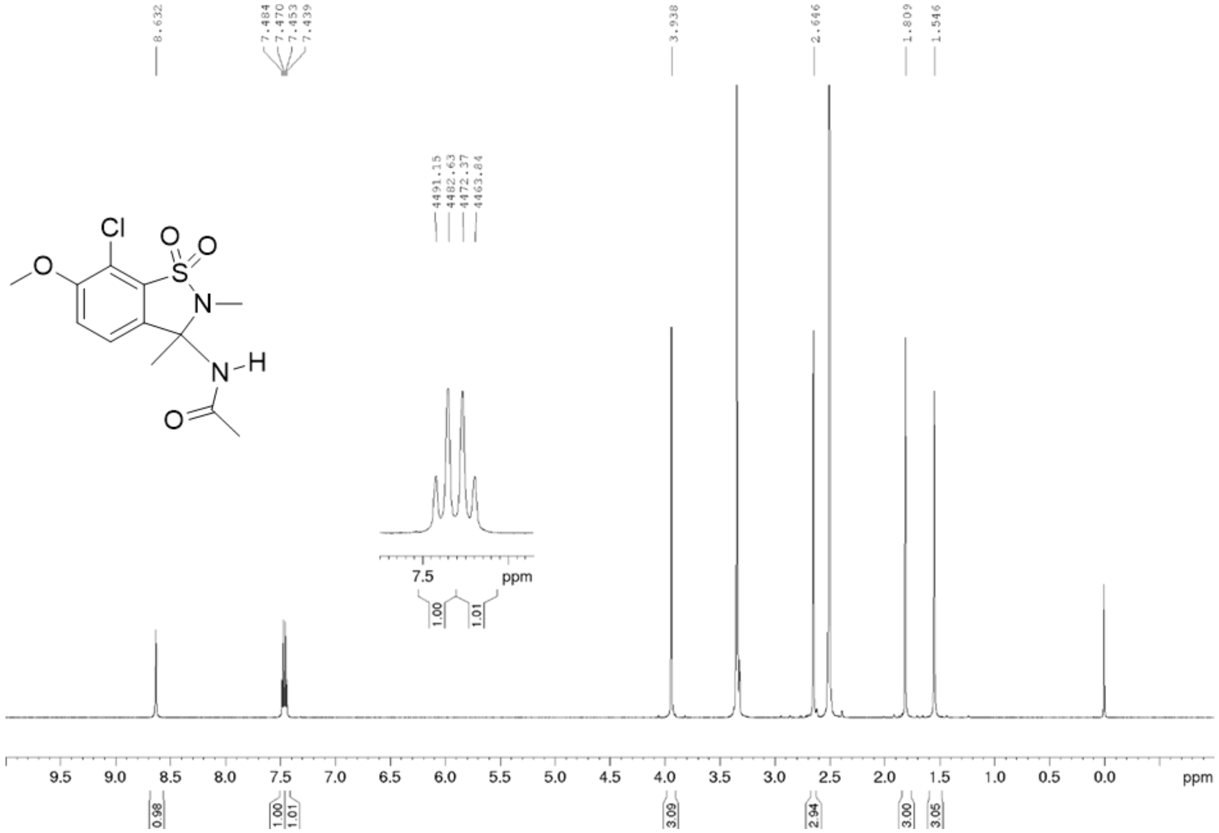

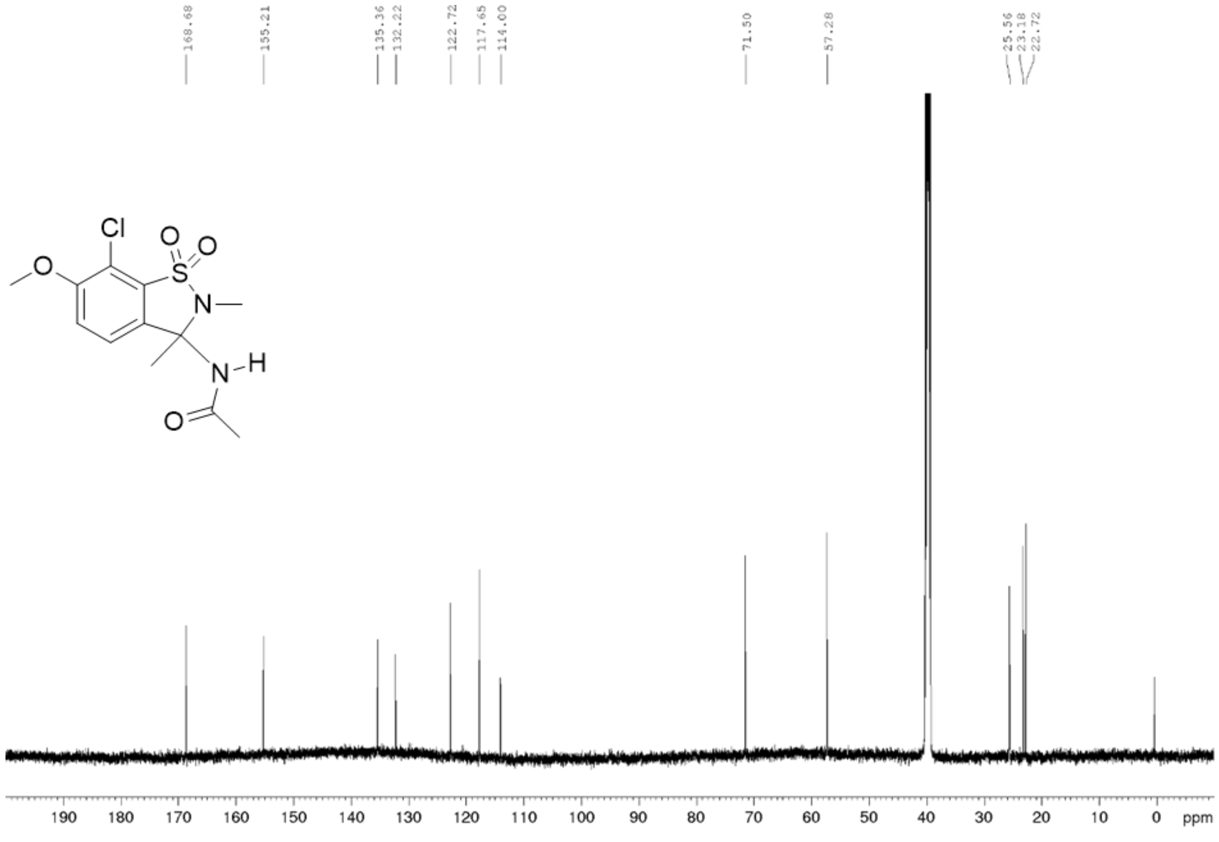

Compound **2m**, ^{1}H and ^{13}C NMR (600/150 MHz, [D$_6$]DMSO)

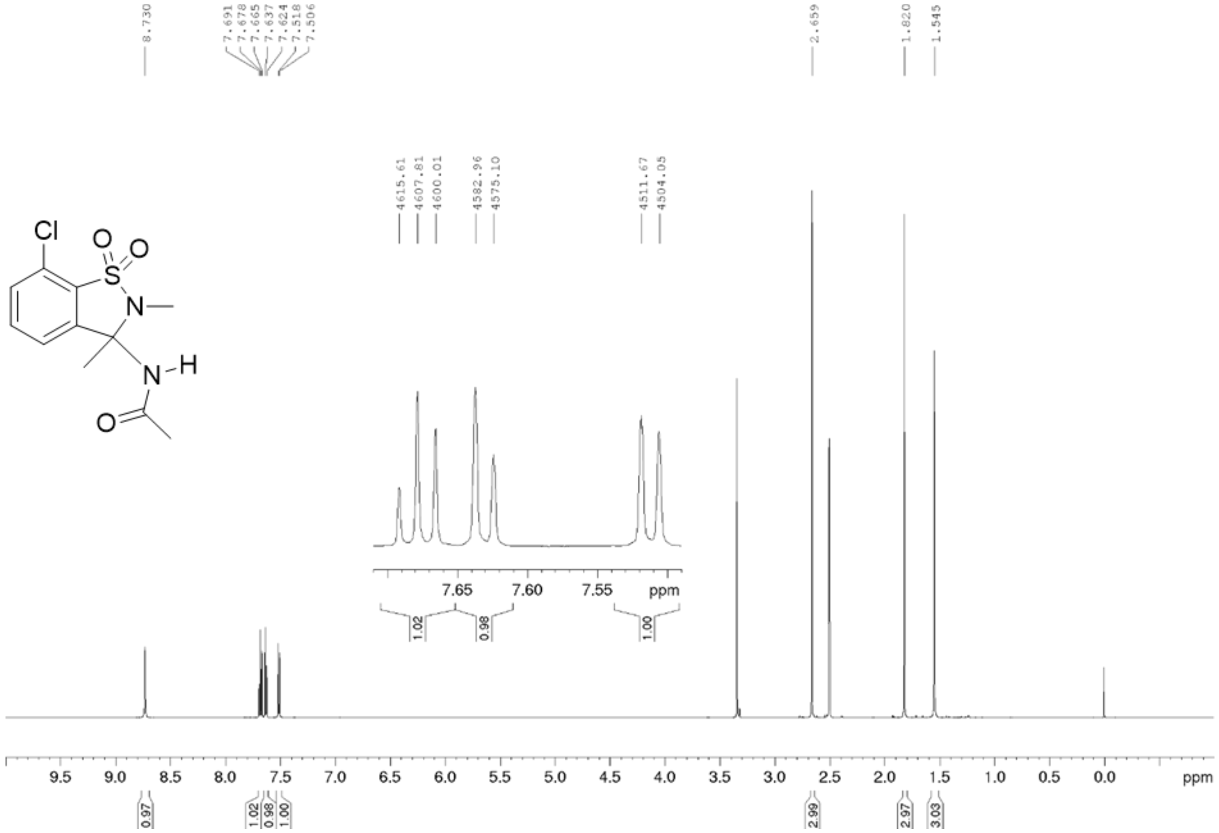

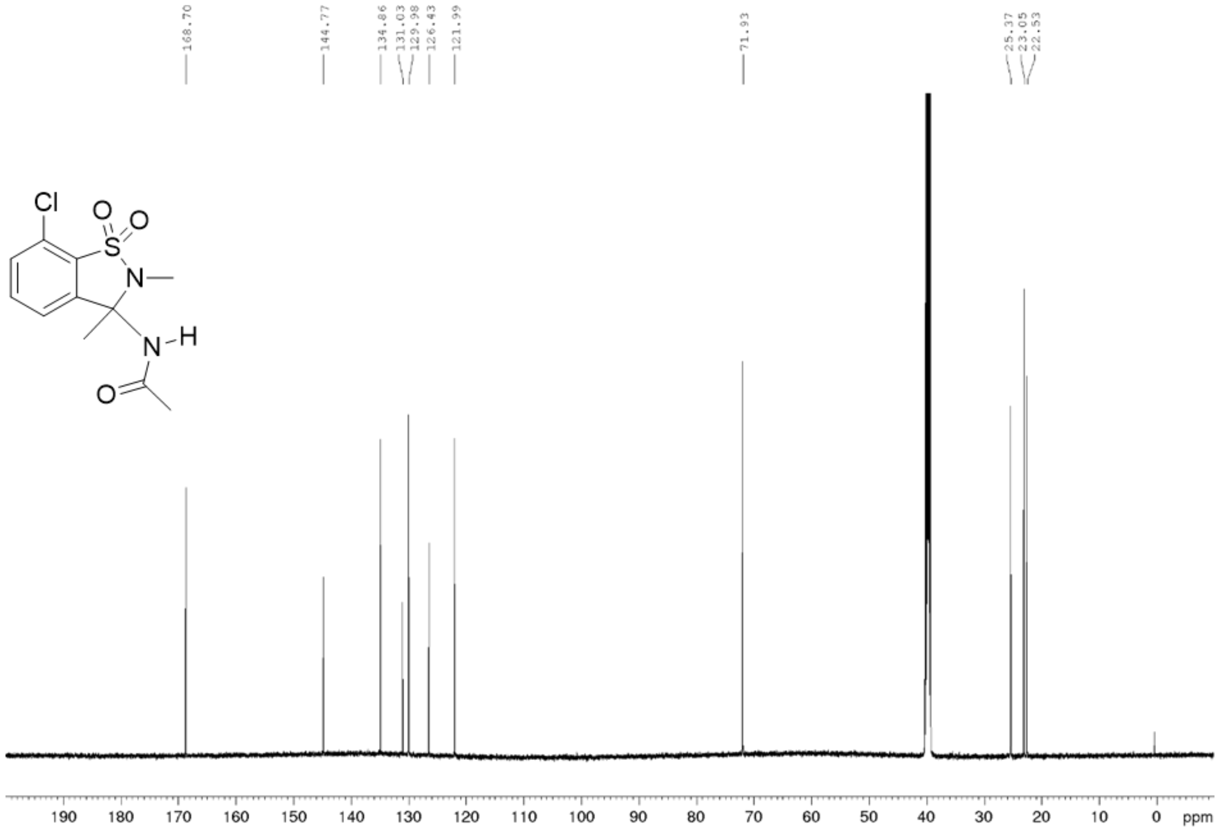

Compound **2n**, ^{1}H and ^{13}C NMR (600/150 MHz, [D$_6$]DMSO)

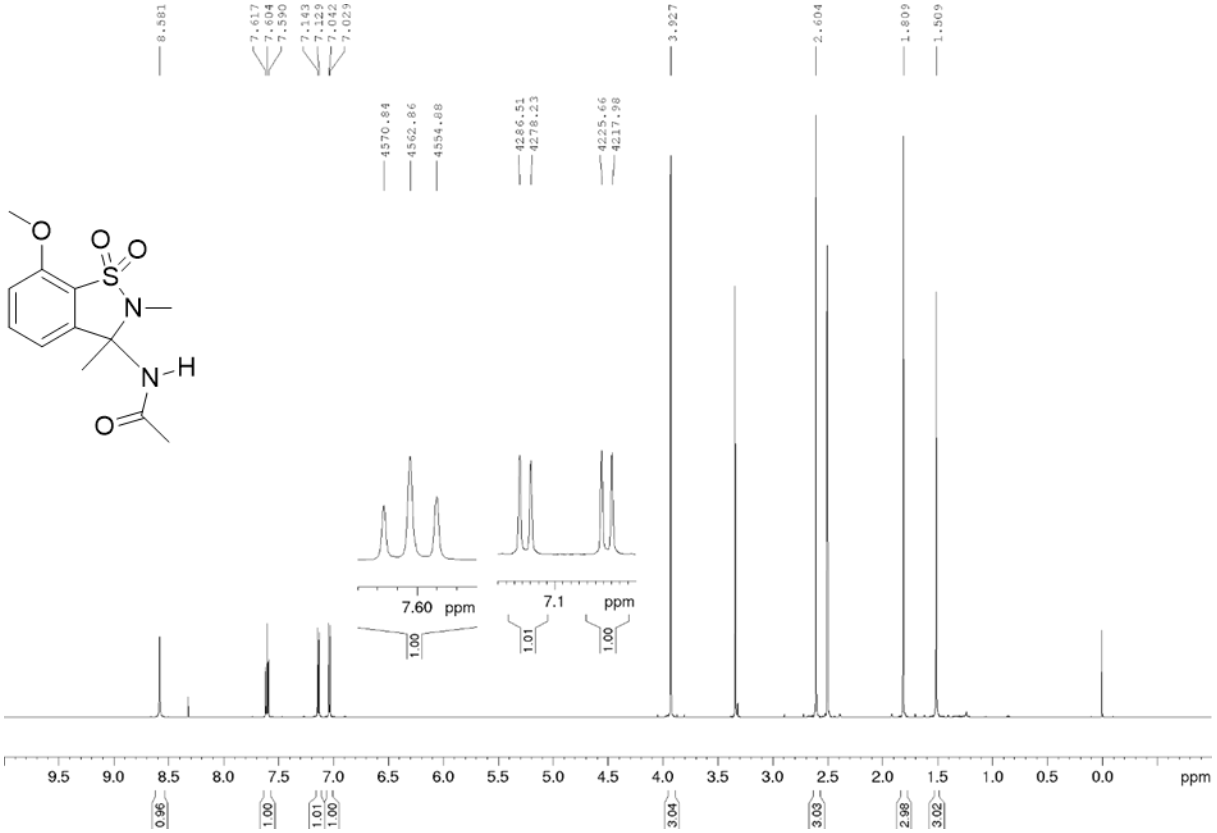

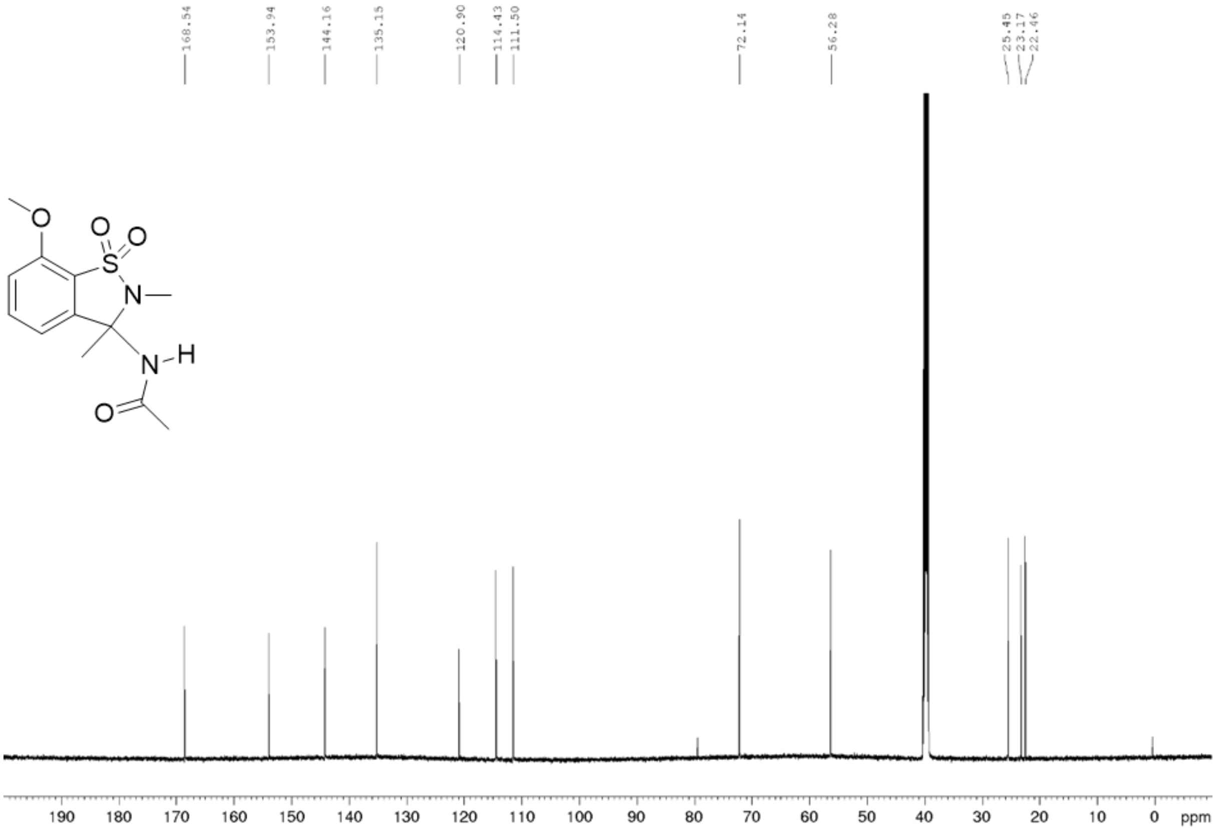

Compound **2o**, ^{1}H and ^{13}C NMR (600/150 MHz, [D$_6$]DMSO)

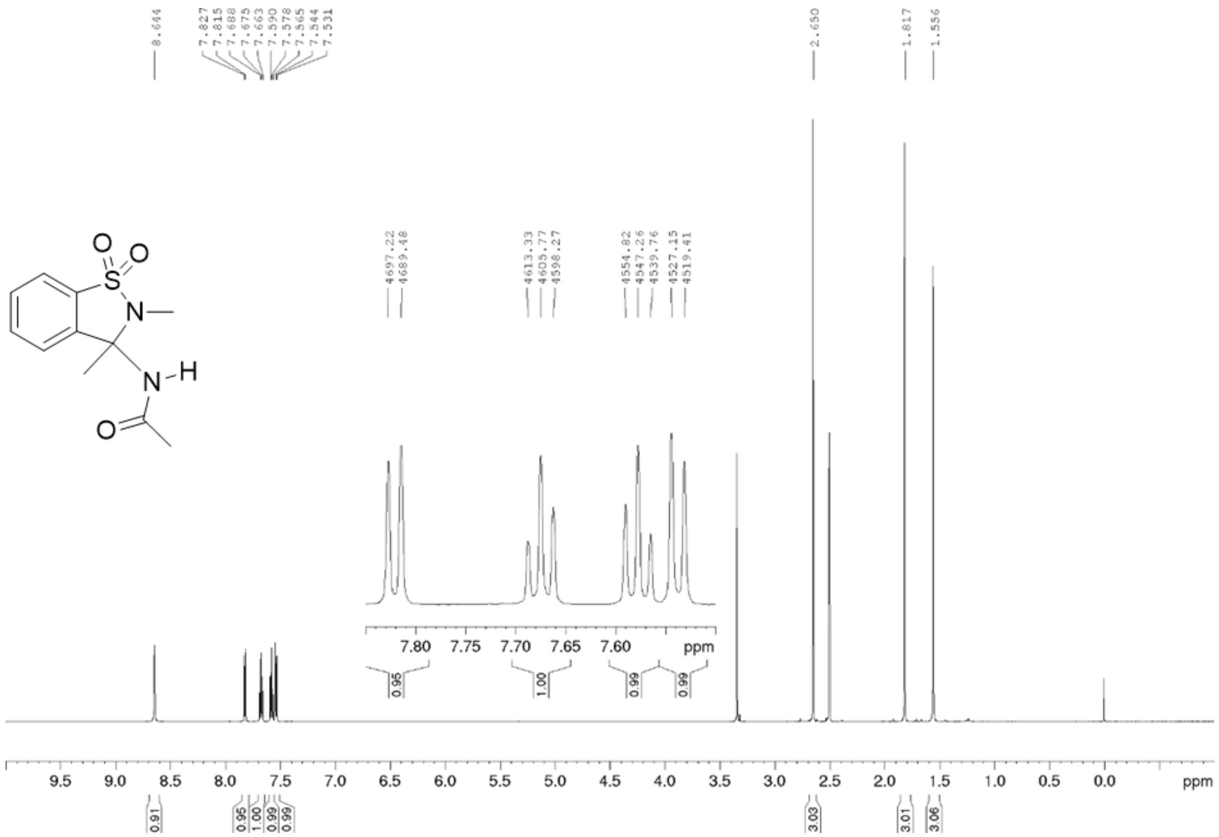

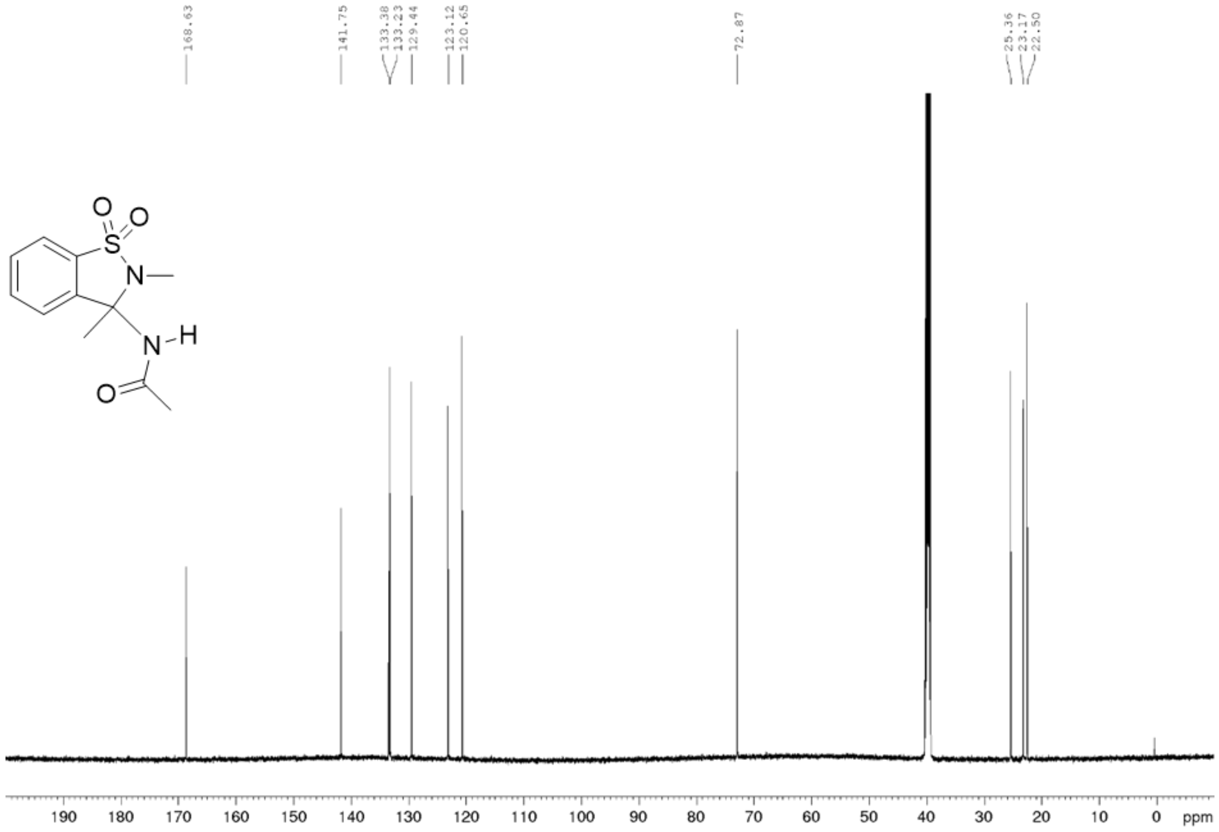

Compound **2q**, ^{1}H and ^{13}C NMR (600/150 MHz, [D$_6$]DMSO)

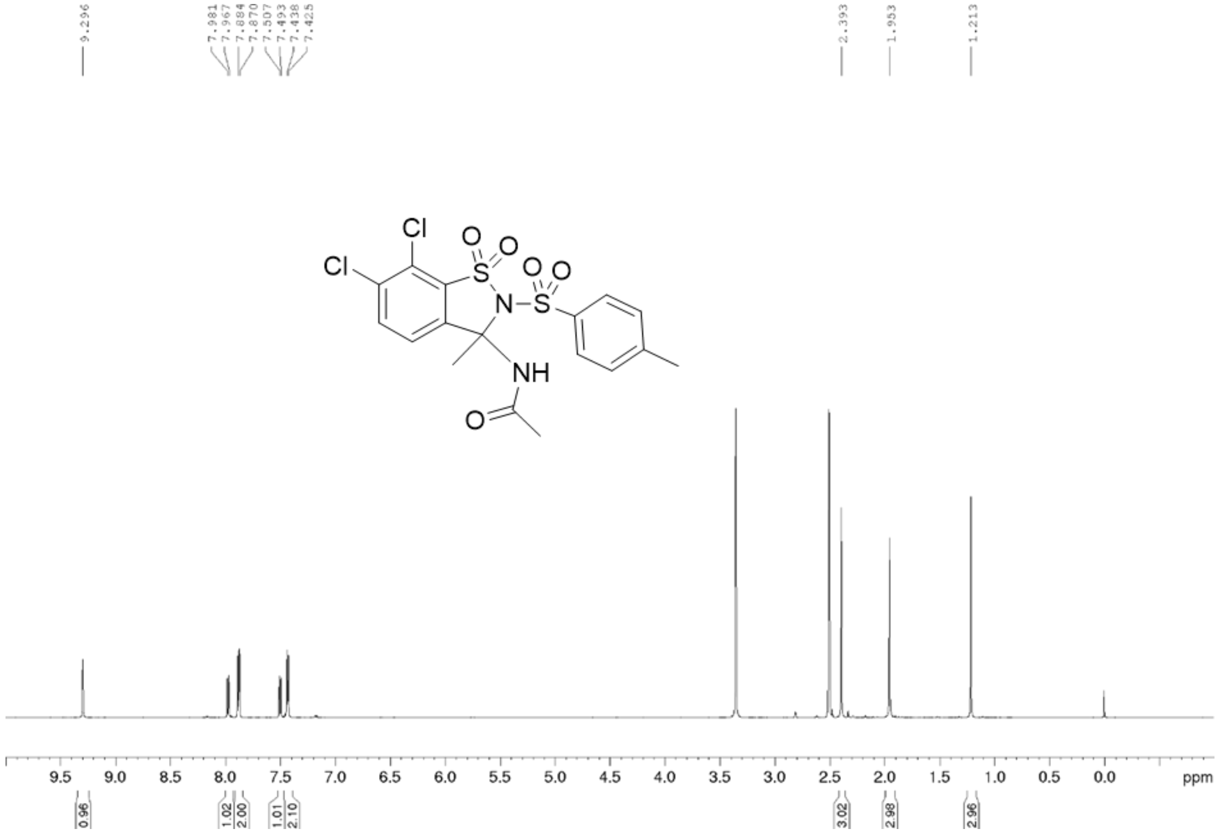

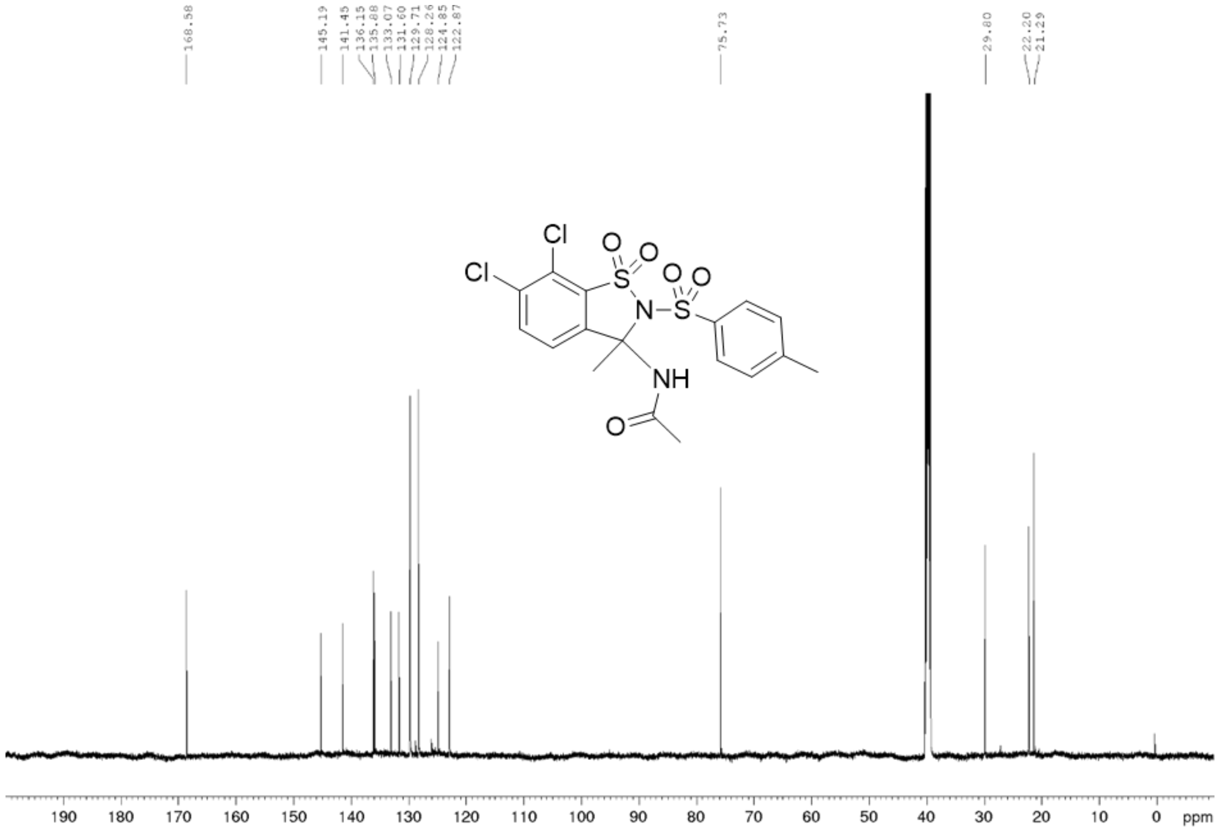

Compound **5q**, ^{1}H and ^{13}C NMR (600/150 MHz, [D$_6$]DMSO)

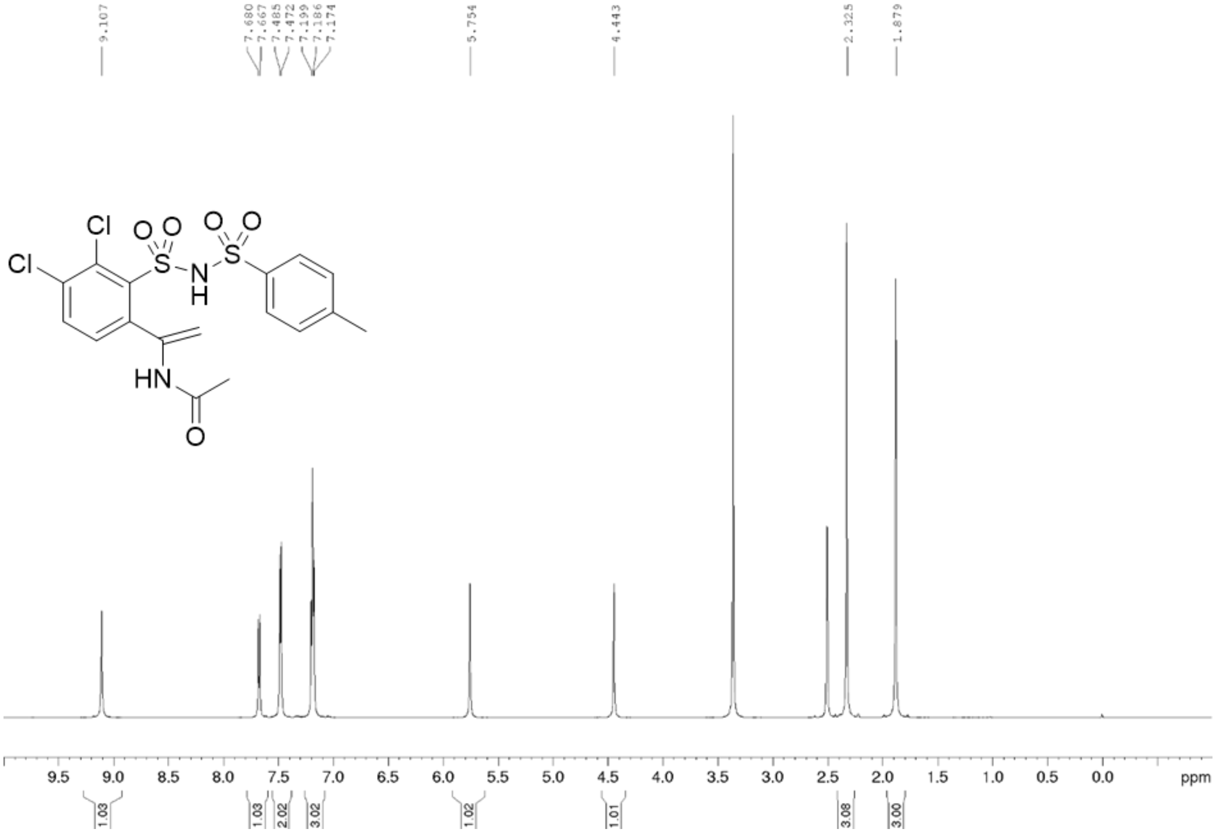

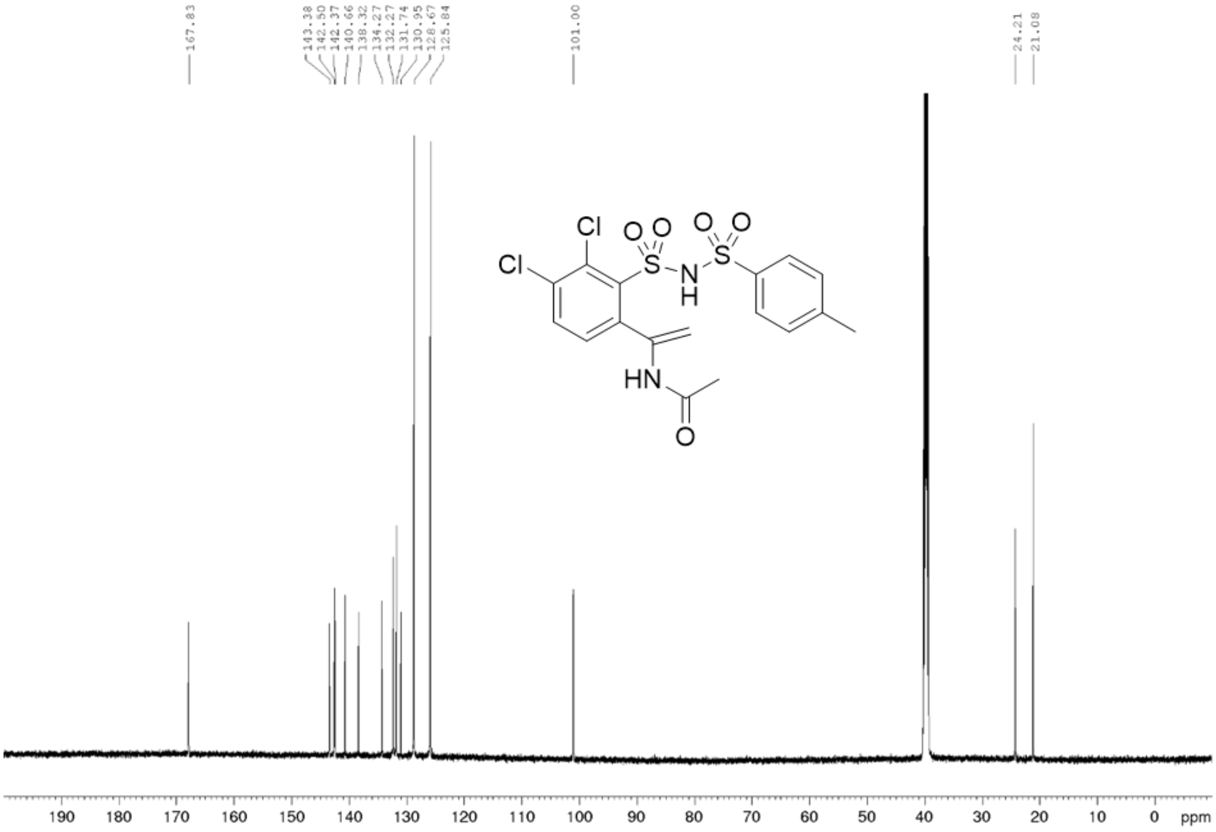

Compound **3a**, ^{1}H and ^{13}C NMR (600/150 MHz, [D$_6$]DMSO)

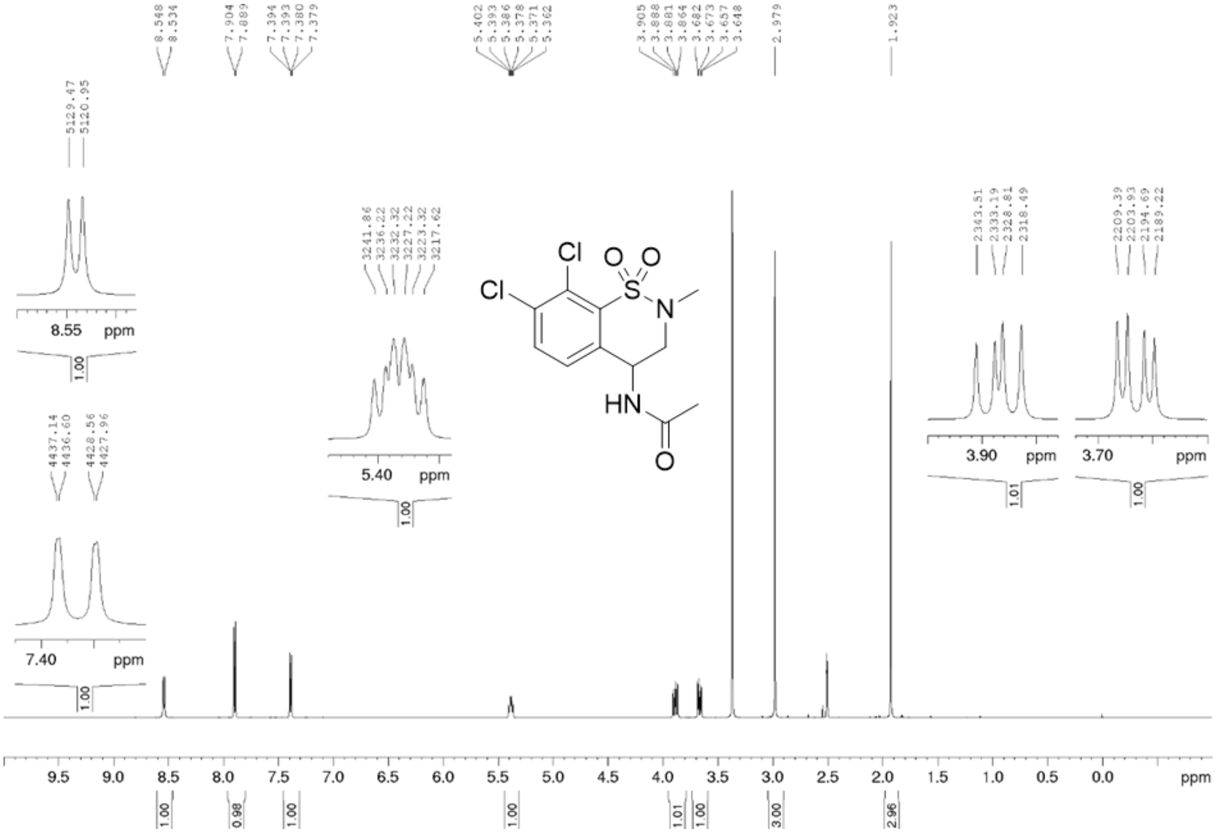

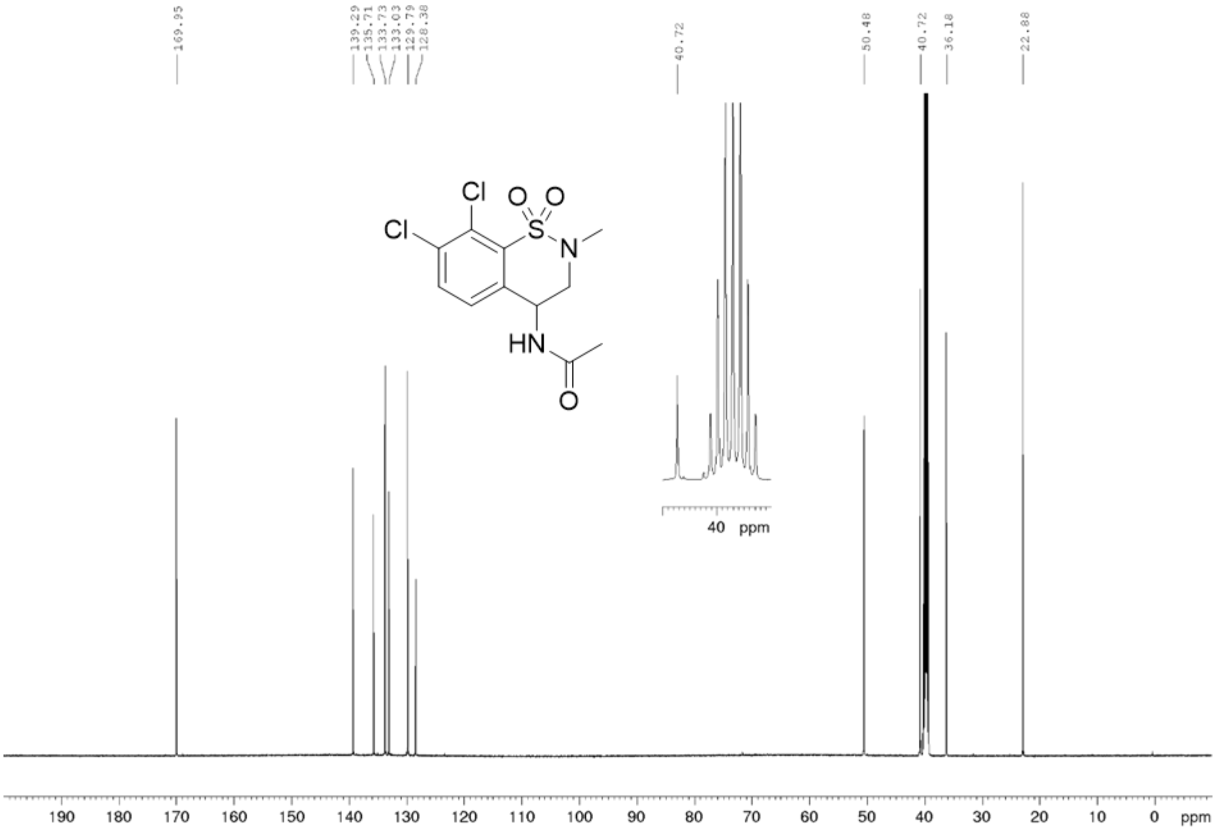

Compound ***trans*-3b**, ^{1}H NMR and DEPTQ (600/150 MHz, [D$_6$]DMSO)

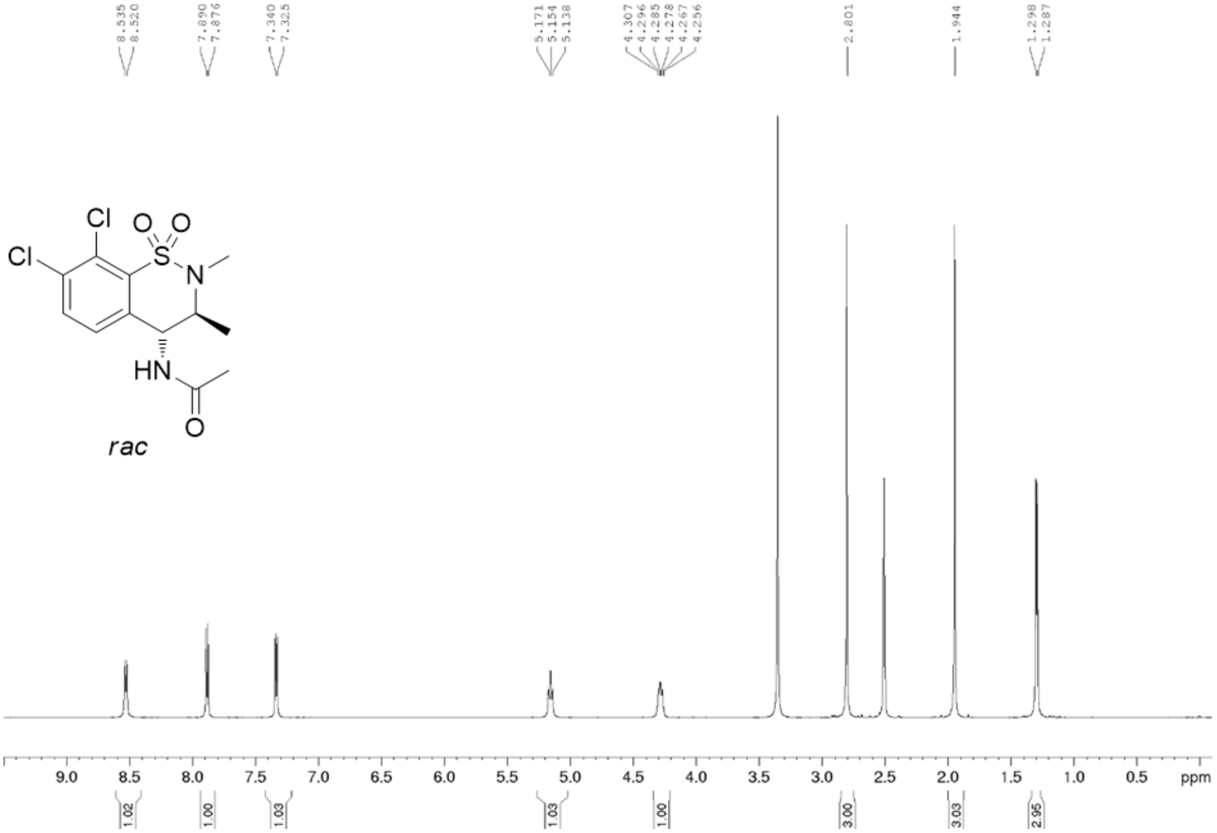

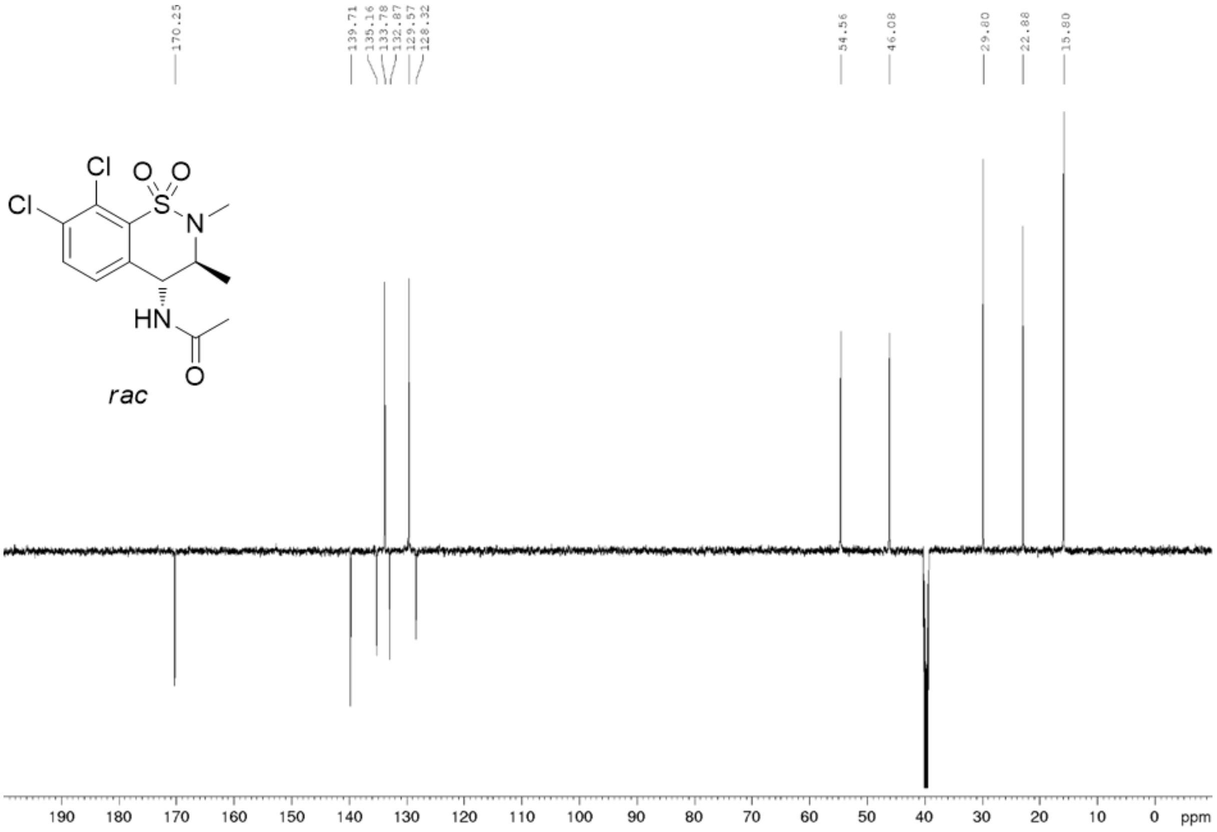

Compound **3c**, ^{1}H and ^{13}C NMR (600/150 MHz, [D$_6$]DMSO)

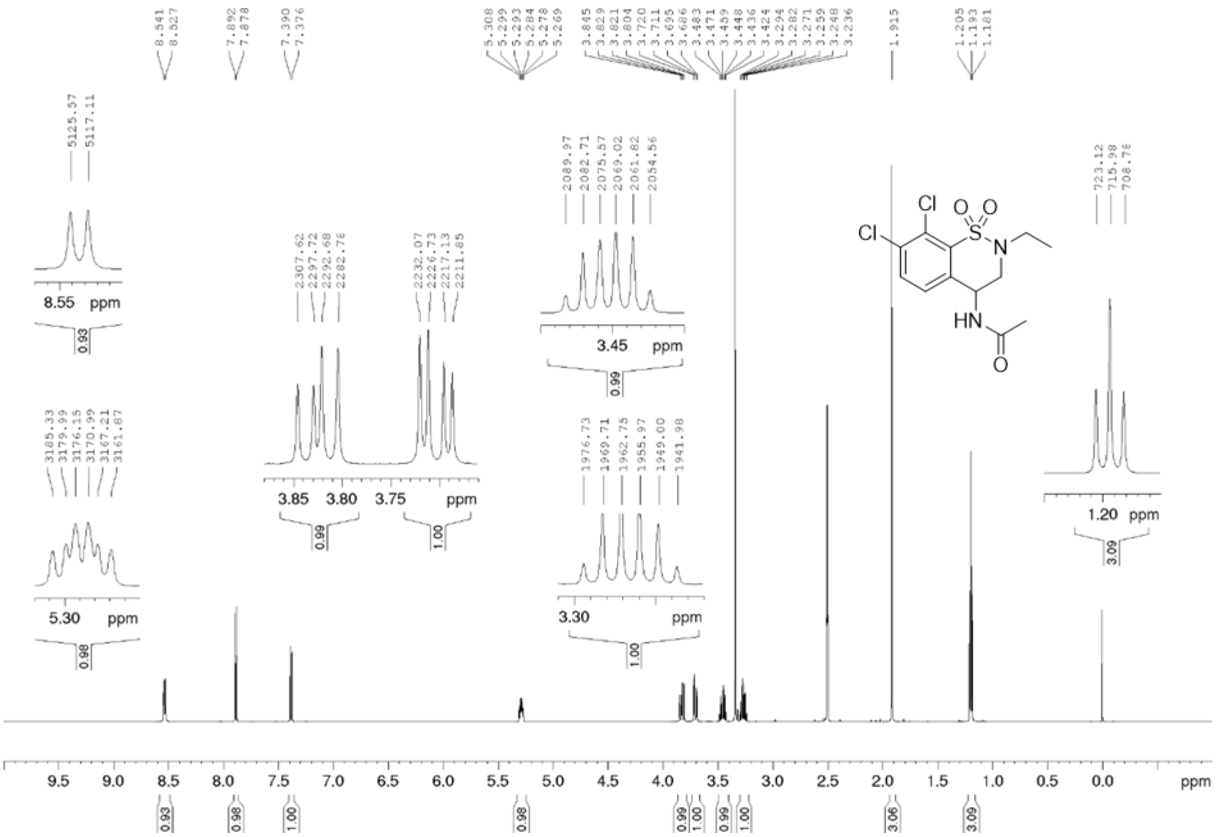

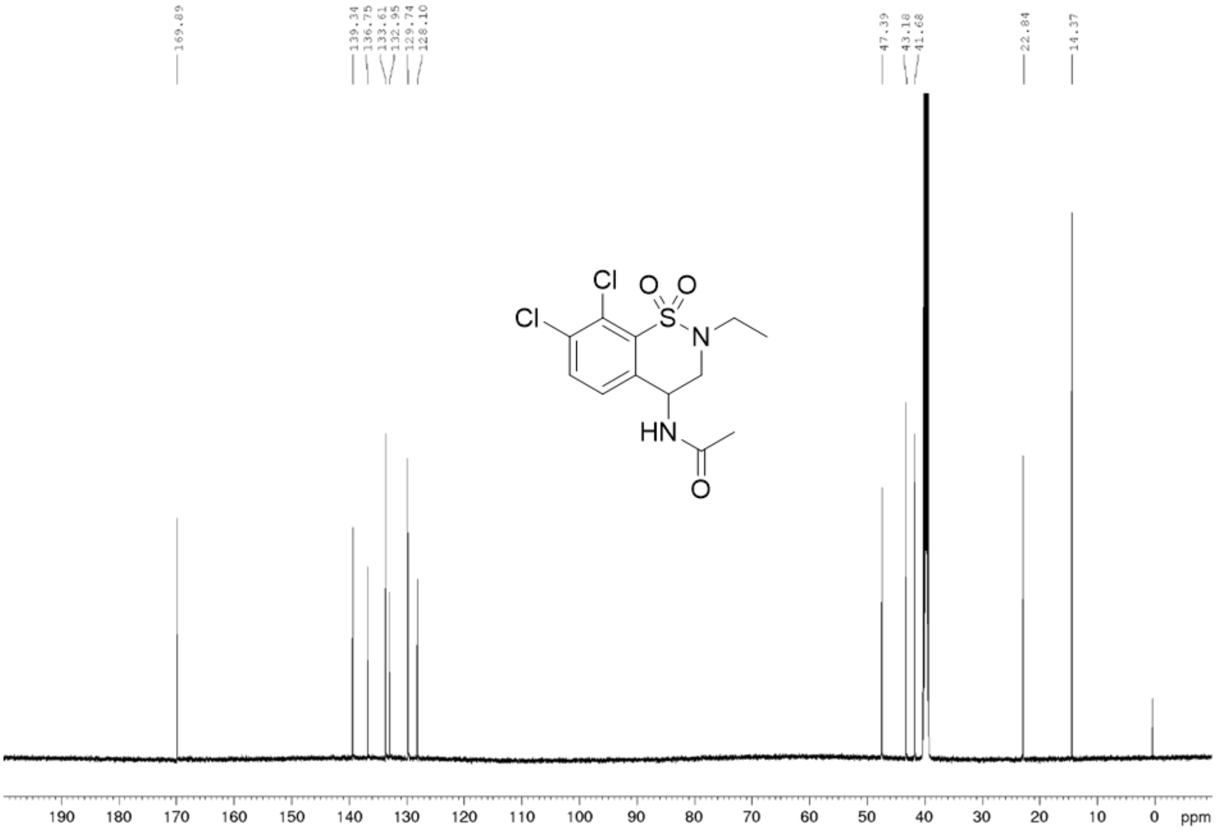

Compound **3d**, ^{1}H and ^{13}C NMR (600/150 MHz, [D$_6$]DMSO)

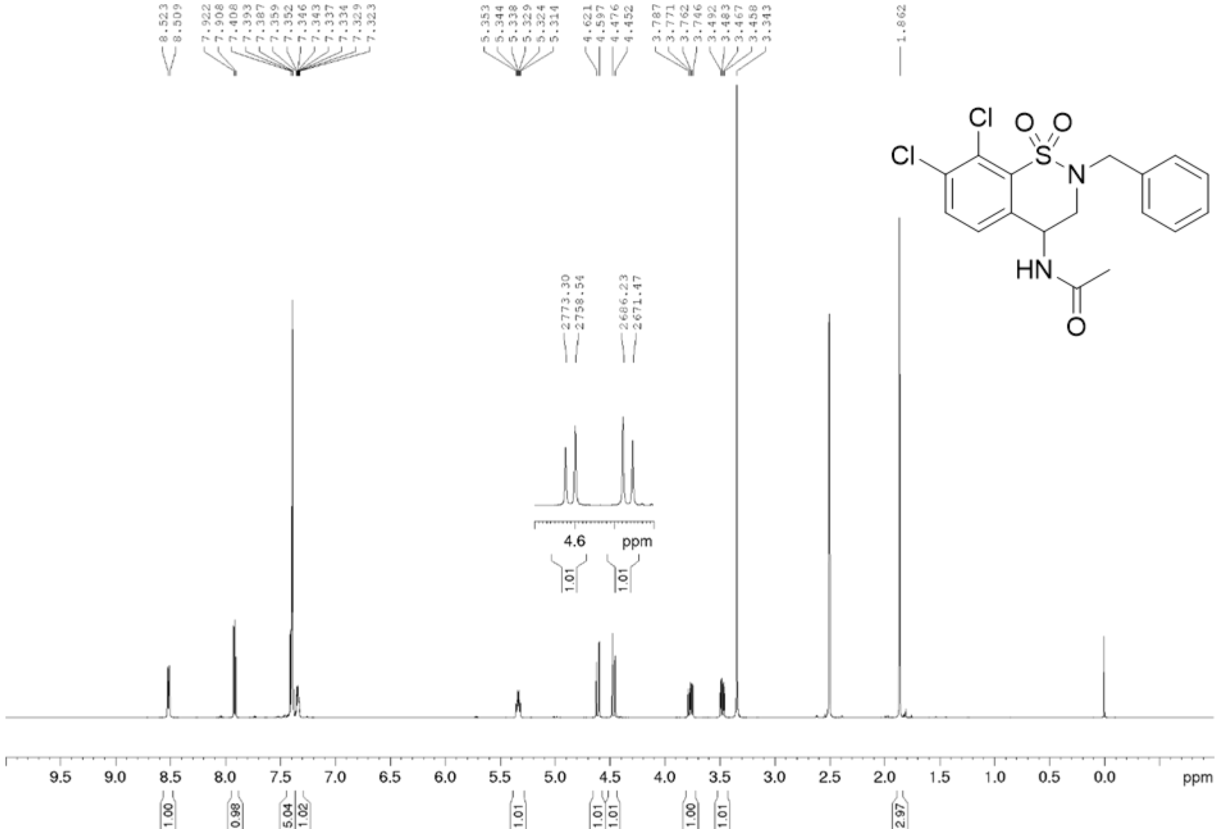

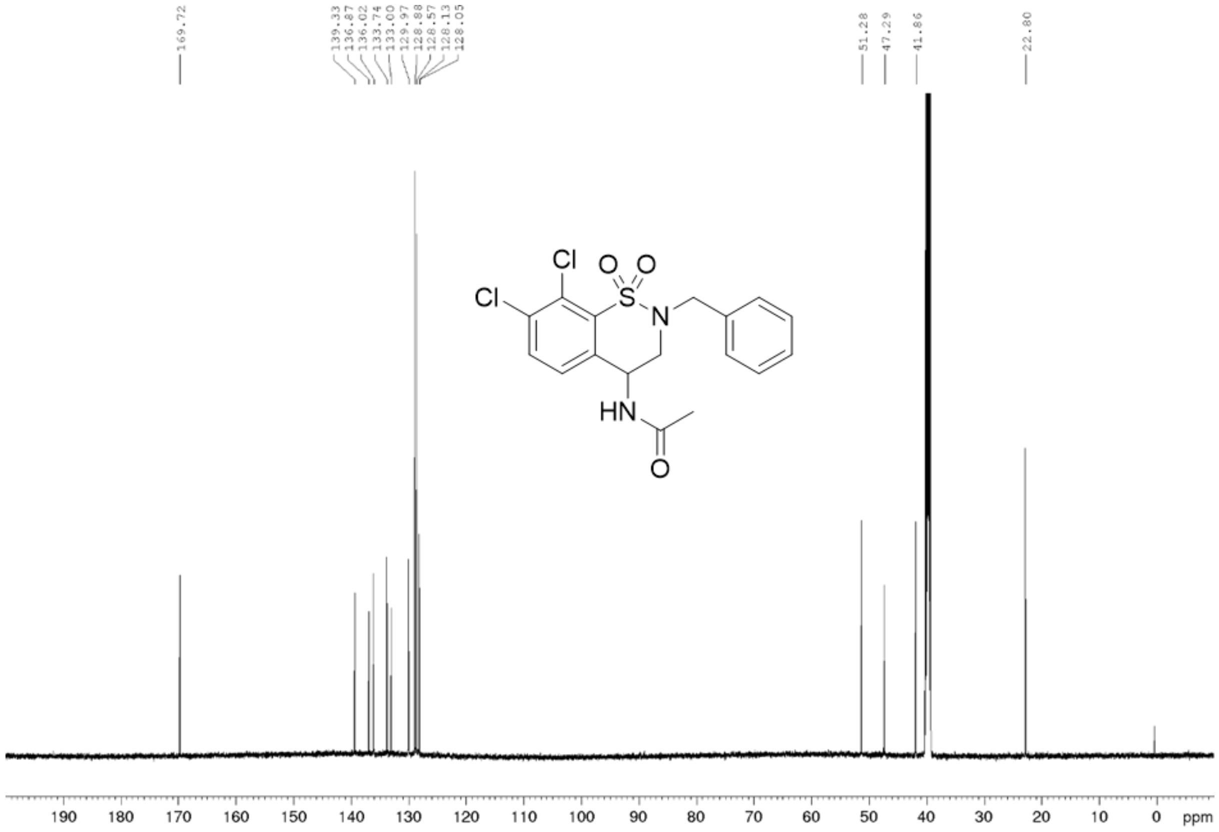

Compound **3e**, ^{1}H and ^{13}C NMR (600/150 MHz, [D$_6$]DMSO)

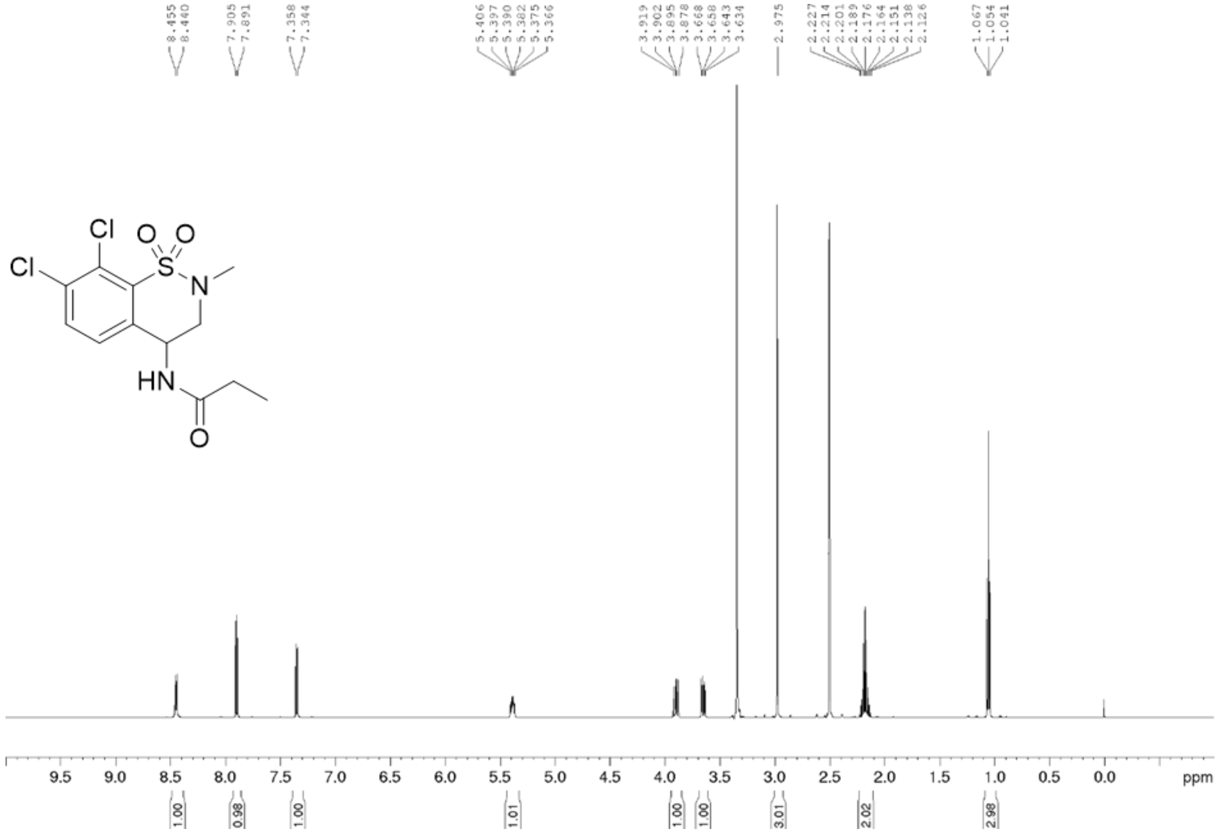

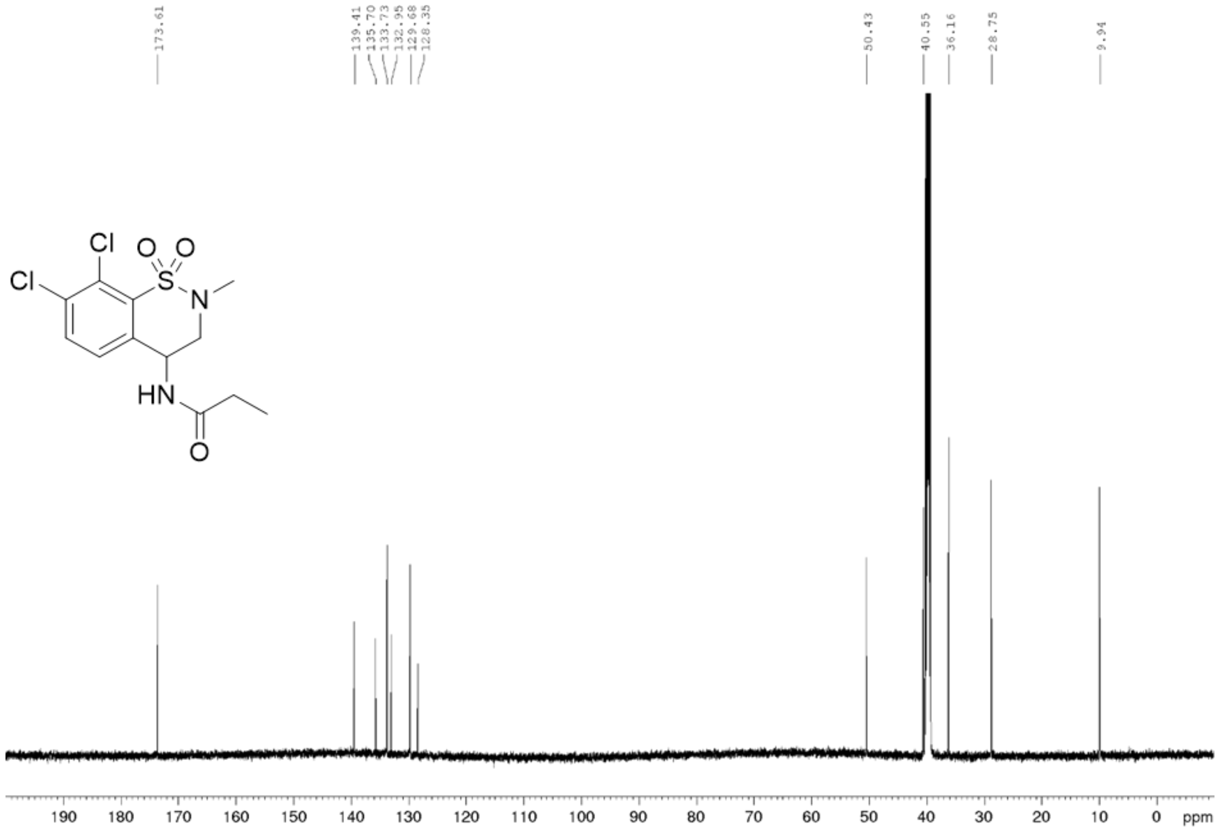

Compound **3f**, ^{1}H and ^{13}C NMR (600/150 MHz, [D$_6$]DMSO)

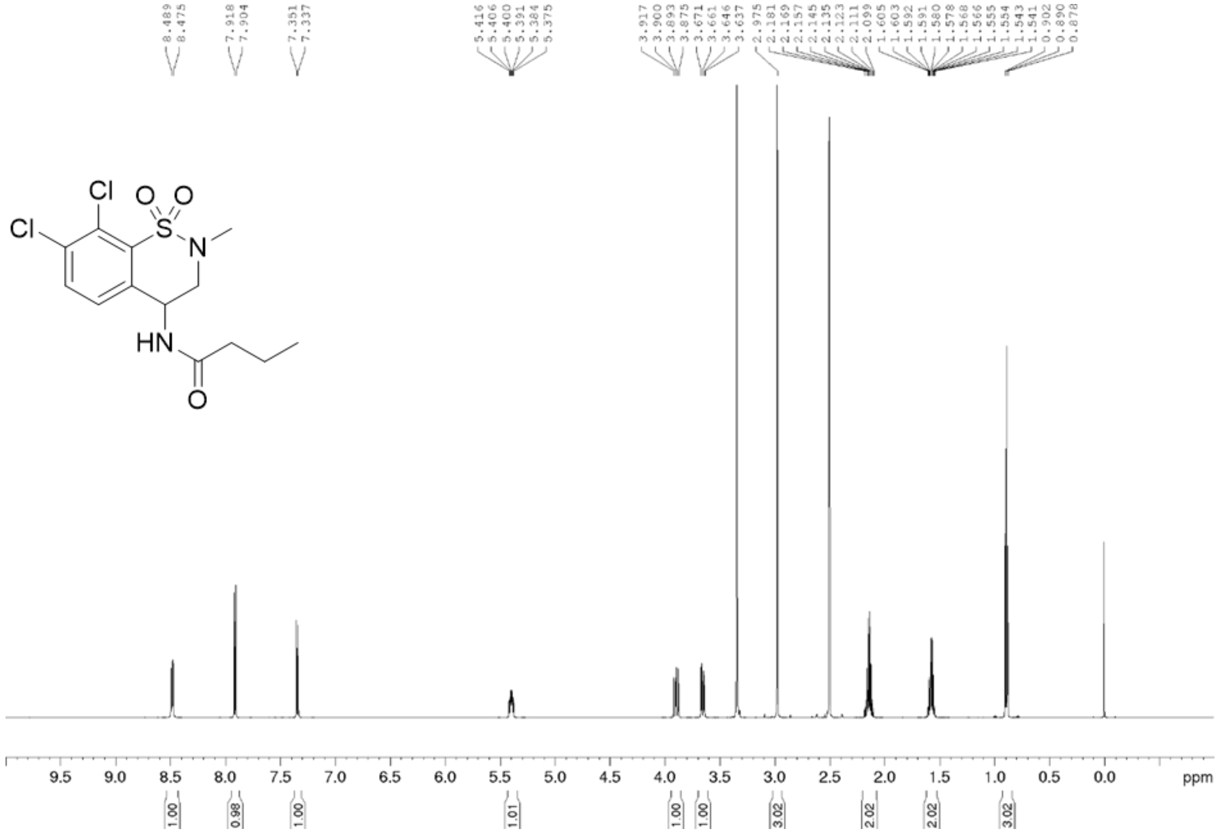

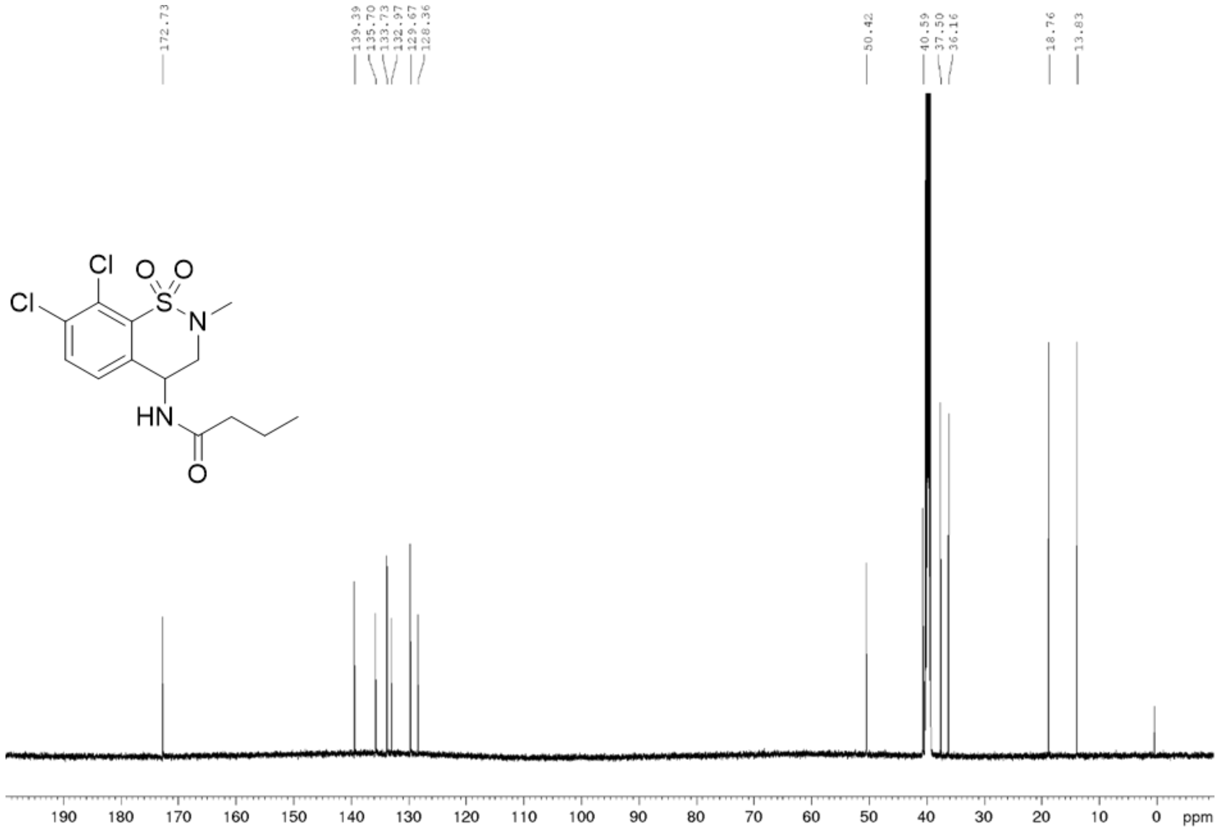

Calculated total energies and XYZ coordinates

M06-2X/6-31+G* (smd: THF) with ultrafine grid

1a

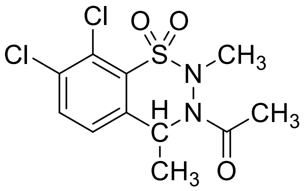

E=-2079.7081933

C	0.009067	-0.040296	0.008365
C	0.000090	-0.019833	1.529249
C	1.144447	-0.012415	2.339050
S	2.760229	0.138562	1.565958
N	2.341380	0.757175	0.064308
N	1.373019	-0.037576	-0.525034
C	1.053364	-0.119378	3.735920
C	-0.204563	-0.173199	4.332850
C	-1.351910	-0.146627	3.543059
C	-1.245820	-0.081377	2.164509
Cl	2.475627	-0.230919	4.714895
Cl	-0.376916	-0.290254	6.054228
O	3.545670	1.175093	2.215277
O	3.307340	-1.194286	1.374170
C	2.164486	2.217339	-0.030406
C	1.689230	-0.742363	-1.671617
C	3.113877	-0.643859	-2.149750
C	-0.858878	1.057835	-0.620733
O	0.827974	-1.396900	-2.239044
H	-2.326658	-0.198640	4.017512
H	-2.152398	-0.103717	1.567928
H	-0.401898	-1.009235	-0.291574
H	1.400912	2.598495	0.656969
H	3.120329	2.694976	0.181152
H	1.873740	2.427217	-1.061746
H	3.382685	0.395592	-2.361005
H	3.803511	-1.016870	-1.387565
H	3.205115	-1.240368	-3.057727
H	-1.915949	0.883888	-0.404261
H	-0.592328	2.051390	-0.252527
H	-0.731349	1.032043	-1.706952

2a

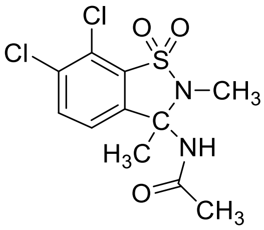

E=-2079.7458281

C	-0.007597	0.003330	-0.002162
C	-0.003354	0.007734	1.517908
C	1.257160	-0.011985	2.082484
S	2.496211	-0.071359	0.814782
N	1.370651	-0.398680	-0.368831
C	1.467088	0.041983	3.455501
C	0.337931	0.104084	4.273452
C	-0.944105	0.119990	3.715069
C	-1.123468	0.077342	2.337865
Cl	3.069603	0.036962	4.097838
Cl	0.503025	0.163846	5.998680
O	3.411727	-1.195541	0.962373
O	3.103622	1.250245	0.638107
C	1.824443	-0.179617	-1.739350
N	-0.956722	-0.942462	-0.542165
C	-1.009080	-2.263835	-0.177689
O	-0.339627	-2.712969	0.740100
C	-0.371903	1.395665	-0.526119
C	-1.943804	-3.113809	-1.004730
H	-1.802062	0.164359	4.378614
H	-2.123512	0.082352	1.913534
H	1.963620	0.881020	-1.977791
H	2.766465	-0.712756	-1.890281
H	1.079786	-0.615455	-2.410661
H	-2.787201	-2.542982	-1.402014
H	-1.382107	-3.533032	-1.847051
H	-2.311408	-3.938753	-0.392355
H	-0.380338	1.405358	-1.620786
H	-1.364936	1.677812	-0.164782
H	0.355879	2.130964	-0.171912
H	-1.488611	-0.648083	-1.353566

3a

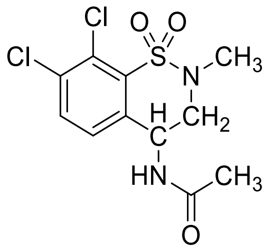

E=-2079.7441753

C	0.088191	-0.028368	-0.061538
C	0.022919	-0.014207	1.465732
C	1.146527	0.020327	2.304031
S	2.801461	0.096520	1.584545
N	2.504979	0.516518	0.011984
C	1.486336	-0.338193	-0.603005
C	1.008466	-0.035893	3.699975
C	-0.268203	-0.057356	4.260342
C	-1.392656	-0.041979	3.438252
C	-1.241883	-0.033152	2.062154
Cl	2.397611	-0.115546	4.729838
Cl	-0.492874	-0.110612	5.979410
O	3.548459	1.191297	2.184087
O	3.341015	-1.256582	1.605189
C	2.366713	1.957524	-0.253272
N	-0.859324	-0.972887	-0.613209
C	-1.848573	-0.613465	-1.478476
C	-2.690581	-1.746403	-2.013669
O	-2.045215	0.553353	-1.804474
H	-2.381244	-0.049298	3.886226
H	-2.124439	-0.047567	1.429200
H	1.525071	2.414629	0.282211
H	3.289296	2.465540	0.022842
H	2.208054	2.064563	-1.328791
H	-2.558691	-1.795006	-3.098752
H	-2.435005	-2.715366	-1.579036
H	-3.742791	-1.525607	-1.814779
H	1.510888	-0.171761	-1.683129
H	1.746845	-1.384669	-0.414896
H	-0.733407	-1.955698	-0.394400
H	-0.230737	0.955999	-0.422020

4a

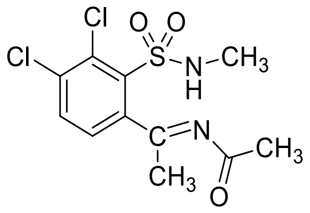

E=-2079.7051622

C	0.051073	0.022270	-0.000515
C	0.054284	0.021142	1.398943
C	1.271999	-0.020860	2.100203
C	2.466452	-0.169688	1.389195
C	2.458002	-0.246313	0.000523
C	1.259266	-0.133555	-0.684386
S	-1.496732	0.091442	2.332092
O	-1.386520	-0.866013	3.423723
Cl	1.343107	0.176326	3.818404
Cl	3.992188	-0.252119	2.211225
C	-1.156350	0.258193	-0.867097
N	-1.550475	1.460634	-0.984463
C	-2.590239	1.809144	-1.860812
C	-3.932479	1.965216	-1.197718
C	-1.677847	-0.921098	-1.642034
O	-1.804439	1.491872	2.595754
N	-2.611759	-0.469016	1.254513
C	-3.032316	-1.870292	1.355196
O	-2.381778	2.026663	-3.037584
H	3.393434	-0.364711	-0.536724
H	1.259856	-0.143040	-1.770761
H	-2.160669	-2.520707	1.252057
H	-3.538188	-2.091299	2.298860
H	-3.709242	-2.062595	0.520197
H	-4.295695	0.974258	-0.896444
H	-3.841729	2.578143	-0.295874
H	-4.644270	2.414157	-1.892140
H	-2.720582	-0.776876	-1.939351
H	-1.078376	-1.044041	-2.552726
H	-1.587775	-1.838972	-1.056535
H	-3.365876	0.209412	1.151355

5a

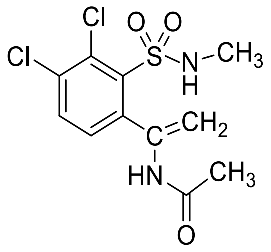

E=-2079.7148145

C	-0.006744	-0.002027	0.015107
C	0.004193	-0.015613	1.411502
C	1.240812	-0.008368	2.087621
C	2.431502	0.148238	1.359498
C	2.384472	0.165996	-0.041326
C	1.172820	0.057508	-0.711108
C	-1.313238	0.062193	2.107641
C	-2.289261	-0.828356	1.913634
S	1.313514	-0.311240	3.893241
N	0.064402	-1.283609	4.286348
C	0.010959	-2.634584	3.728212
Cl	3.959865	0.408293	2.130528
Cl	3.831022	0.338916	-0.984590
O	2.513549	-1.101706	4.119560
O	1.115072	0.959322	4.581348
N	-1.437538	1.194388	2.947644
C	-2.020416	1.222985	4.186373
O	-2.513166	0.221252	4.699019
C	-1.956793	2.547039	4.897802
H	1.155778	0.065714	-1.796059
H	-0.960732	-0.010306	-0.502935
H	-0.038538	-2.637504	2.631914
H	0.883734	-3.200769	4.056698
H	-0.890252	-3.107041	4.122959
H	-2.903555	2.716916	5.415055
H	-1.161465	2.490681	5.648638
H	-1.748555	3.379956	4.223022
H	-3.256963	-0.723114	2.391021
H	-2.113675	-1.695658	1.285078
H	-0.914494	2.020761	2.674337
H	-0.837874	-0.814821	4.395092

I⁻

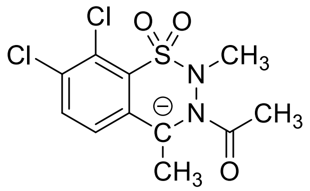

E=-2079.1997389

C	-0.938962	2.348753	0.159922
C	0.031347	1.271298	0.061767
C	-0.567407	-0.063916	-0.008676
C	-1.960216	-0.244046	-0.104158
C	-2.824876	0.830670	-0.050099
C	-2.283184	2.135108	0.103789
S	0.536452	-1.416574	0.027914
N	1.886218	-0.717590	0.822087
N	2.276662	0.420787	0.112428
C	1.377020	1.534318	0.103720
C	1.967271	2.896086	0.345695
O	0.091067	-2.507677	0.895782
O	1.024702	-1.759487	-1.309911
C	1.691125	-0.470248	2.255800
C	3.386943	0.325235	-0.695913
O	3.676594	1.231819	-1.468644
Cl	-2.617279	-1.842538	-0.354211
Cl	-4.556987	0.661178	-0.215656
C	4.238285	-0.913127	-0.540564
H	-2.968523	2.976827	0.161889
H	-0.573495	3.366507	0.253622
H	0.887368	0.249540	2.455302
H	1.467015	-1.420631	2.740059
H	2.635881	-0.079465	2.641859
H	4.472642	-1.108961	0.509573
H	3.709118	-1.787019	-0.930499
H	5.159021	-0.759548	-1.105730
H	1.491001	3.404717	1.197005
H	3.032148	2.802925	0.574216
H	1.883965	3.562990	-0.523289

II⁻

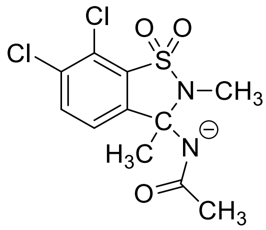

E=-2079.2467176

C	-1.755323	-0.212508	0.785915
C	-0.312557	-0.659415	0.582195
C	0.541442	0.315563	0.099156
S	-0.355118	1.820720	-0.185627
N	-1.816846	1.071100	-0.010607
C	1.905484	0.106027	-0.075248
C	2.408988	-1.152542	0.248667
C	1.560663	-2.149952	0.742025
C	0.205320	-1.907762	0.917814
Cl	2.925657	1.377236	-0.660660
Cl	4.100698	-1.509641	0.052066
O	-0.196514	2.337025	-1.545173
O	-0.070601	2.781662	0.891142
C	-2.950602	1.960501	0.207704
N	-2.771775	-1.132101	0.372091
C	-2.605517	-1.690693	-0.831825
O	-1.652313	-1.527226	-1.628830
C	-1.953703	0.084374	2.279660
C	-3.721210	-2.641983	-1.251600
H	1.982257	-3.121452	0.982609
H	-0.450650	-2.689339	1.291035
H	-2.904967	2.485450	1.170597
H	-2.998258	2.694831	-0.601505
H	-3.853266	1.346341	0.168976
H	-4.493579	-2.744756	-0.485324
H	-4.179151	-2.273414	-2.176629
H	-3.295390	-3.627708	-1.471226
H	-2.978954	0.426604	2.452675
H	-1.804806	-0.836772	2.851922
H	-1.251509	0.845222	2.638741

III⁻

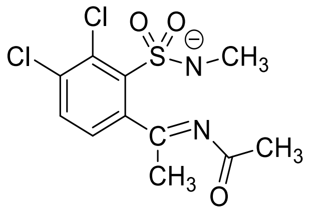

E=-2079.2208202

C	1.974512	-0.011257	-0.171359
C	2.573882	-1.251161	0.081773
C	1.809945	-2.343657	0.474854
C	0.433882	-2.205841	0.576308
C	-0.178898	-0.975634	0.329986
C	0.592767	0.149099	0.007835
Cl	4.293040	-1.480884	-0.093833
C	-1.683548	-0.975034	0.349908
N	-2.268504	-1.078459	-0.776133
C	-3.659968	-1.152519	-0.874720
C	-4.321763	0.175468	-1.130183
S	-0.270355	1.785288	-0.122581
N	-1.536710	1.675668	0.789315
C	-1.270907	1.936796	2.201859
Cl	2.973125	1.277525	-0.775258
C	-2.376173	-1.082641	1.682841
O	0.729732	2.758579	0.363409
O	-0.640376	1.873983	-1.545516
O	-4.255119	-2.215289	-0.850256
H	2.291562	-3.296728	0.669020
H	-0.174820	-3.068976	0.833476
H	-0.528554	1.250676	2.647418
H	-0.915319	2.959320	2.387544
H	-2.209876	1.800606	2.749041
H	-3.982568	0.910482	-0.392096
H	-4.008942	0.544723	-2.113371
H	-5.407365	0.066540	-1.104191
H	-3.345587	-0.574546	1.660291
H	-2.551095	-2.142238	1.909757
H	-1.766317	-0.657744	2.480966

III⁻ + *t*-BuO⁻

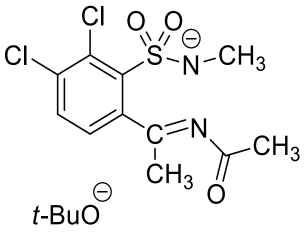

E=-2312.2586146

C	0.000000	0.000000	0.000000
C	0.000000	0.000000	1.402951
C	1.230813	0.000000	2.073928
C	2.421304	-0.119687	1.348228
C	2.398605	-0.251267	-0.034874
C	1.189653	-0.180029	-0.714442
S	-1.618816	-0.174889	2.292903
O	-1.277873	-1.055977	3.432982
Cl	1.329830	0.224930	3.797975
Cl	3.975268	-0.125826	2.145893
C	-1.214244	0.316785	-0.829552
N	-1.667537	1.505114	-0.735171
C	-2.747461	1.964986	-1.483901
C	-4.095661	1.723067	-0.852472
C	-1.637239	-0.705351	-1.839673
O	-2.028767	1.198709	2.634393
N	-2.610986	-0.828175	1.274707
C	-2.440947	-2.270591	1.128137
O	-2.597399	2.598873	-2.514808
O	0.964632	-1.086270	-3.669843
C	1.398565	-2.372708	-3.802263
C	2.820690	-2.553934	-3.213280
C	0.463027	-3.366159	-3.065682
C	1.453925	-2.797394	-5.290484
H	3.330782	-0.381542	-0.577120
H	1.168582	-0.295529	-1.808509
H	-1.440116	-2.561550	0.759153
H	-2.612899	-2.822009	2.063281
H	-3.171924	-2.620508	0.391142
H	-4.252249	0.647165	-0.719405
H	-4.120576	2.173099	0.145217
H	-4.882578	2.149460	-1.477409
H	-2.558118	-0.411822	-2.353143
H	-0.823988	-0.830746	-2.582726

H	-1.786932	-1.672902	-1.351978
H	3.217508	-3.572936	-3.336875
H	3.509886	-1.852079	-3.701155
H	2.808997	-2.322955	-2.140095
H	0.785875	-4.414155	-3.155254
H	0.418151	-3.111976	-1.997360
H	-0.554976	-3.284481	-3.470819
H	1.791376	-3.834574	-5.437282
H	0.457684	-2.691597	-5.739648
H	2.137431	-2.132318	-5.834556

IV^{2-} + *t*-BuOH

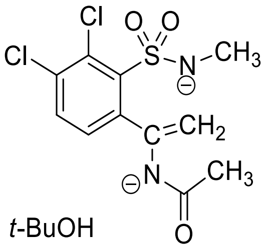

E=-2312.2910731

C	-0.006208	-0.015598	-0.002726
C	-0.003136	-0.004534	1.397389
C	1.217584	0.005155	2.087571
C	2.406708	-0.178793	1.354612
C	2.374888	-0.210188	-0.041576
C	1.169952	-0.092929	-0.728041
C	-1.351316	-0.117483	2.056446
C	-1.600807	-1.299588	2.671097
S	1.292764	0.438445	3.901766
N	0.050317	1.276478	4.306748
C	-1.031076	0.670133	5.065417
Cl	3.939366	-0.456725	2.136960
Cl	3.832957	-0.404002	-0.987562
O	1.447073	-0.869143	4.591669
O	2.484759	1.314595	3.908083
N	-2.165027	0.980986	1.828854
C	-3.447826	0.978713	2.234736
O	-4.077860	0.051843	2.788854
C	-4.187972	2.291742	1.999549
O	-1.372569	3.233895	0.361335

C	-0.591392	4.153815	1.112520
C	-1.306239	4.513942	2.418694
C	0.776798	3.547644	1.429122
C	-0.436017	5.391499	0.233945
H	1.163382	-0.101450	-1.813482
H	-0.960884	0.022997	-0.520588
H	-0.847658	-0.381317	5.323027
H	-1.161193	1.225174	6.006298
H	-1.978780	0.722255	4.514125
H	-5.208793	2.081808	1.664114
H	-4.259373	2.830260	2.953496
H	-3.681305	2.935215	1.277074
H	-2.556202	-1.520385	3.125784
H	-0.799996	-2.031498	2.745516
H	-1.569055	2.424807	0.915911
H	-0.719974	5.236435	2.999771
H	-2.289700	4.952101	2.209983
H	-1.442732	3.617272	3.035328
H	1.432085	4.279009	1.918631
H	0.665423	2.695330	2.107485
H	1.262581	3.204242	0.507174
H	0.153437	6.163019	0.742468
H	0.069618	5.130516	-0.703231
H	-1.419058	5.810199	-0.011833

V⁻

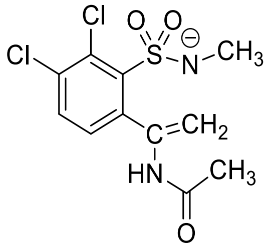

E=-2079.2256846

C	-0.547124	-0.048543	0.070327
C	-1.934466	-0.151709	-0.124605
C	-2.740448	0.993965	-0.100681
C	-2.178381	2.251042	0.073365
C	-0.801787	2.364416	0.175278
C	0.027683	1.236764	0.166401

Cl	-2.710340	-1.667329	-0.481587
Cl	-4.468865	0.898601	-0.306739
C	1.501888	1.509244	0.190862
C	2.027754	2.329002	1.114474
S	0.541644	-1.562117	0.226902
O	0.925652	-1.811313	-1.183562
N	2.201115	0.921323	-0.876178
C	3.546790	0.633617	-0.871211
O	4.352819	1.193549	-0.142072
C	3.934584	-0.444052	-1.853725
N	1.793719	-1.172678	1.056615
C	1.551768	-0.982059	2.481560
O	-0.334227	-2.565929	0.861766
H	-2.813081	3.131210	0.087262
H	-0.350255	3.349261	0.242909
H	0.794900	-0.205420	2.692567
H	1.232628	-1.900562	2.995111
H	2.489739	-0.645458	2.934921
H	5.019495	-0.464530	-1.964704
H	3.580133	-1.406275	-1.467256
H	3.463802	-0.283941	-2.829007
H	3.075346	2.594347	1.120555
H	1.384668	2.711413	1.901069
H	1.655450	0.261110	-1.426706

VI⁻

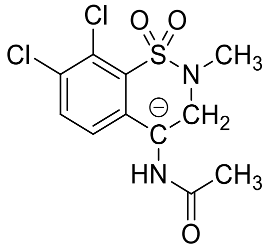

E=-2079.2316795

C	1.753746	0.144467	-0.466960
C	0.482244	0.615648	-0.241411
C	-0.699675	-0.236307	-0.121804
S	-0.417660	-1.967889	-0.277121
N	1.159780	-2.131299	0.266813
C	2.085504	-1.319262	-0.544309

C	-1.988182	0.308355	-0.003083
C	-2.182595	1.675481	0.105864
C	-1.049957	2.525730	0.082363
C	0.211248	2.033496	-0.084541
Cl	-3.389924	-0.731958	-0.052256
Cl	-3.768834	2.398730	0.248306
O	-1.254481	-2.746202	0.636092
O	-0.397816	-2.353185	-1.692219
C	1.303919	-1.925073	1.713797
N	2.831554	1.055651	-0.661581
C	3.936731	1.116223	0.125038
C	5.015693	2.074151	-0.333108
O	4.081331	0.425432	1.134287
H	-1.201938	3.596083	0.198107
H	1.053072	2.718990	-0.093602
H	1.056794	-0.901371	2.024415
H	0.665703	-2.634261	2.241616
H	2.347933	-2.125077	1.965831
H	5.882310	1.496523	-0.671331
H	4.691782	2.730519	-1.145326
H	5.330935	2.681273	0.519610
H	3.087395	-1.501002	-0.141394
H	2.061959	-1.720113	-1.567245
H	2.821808	1.653248	-1.482603

VII$^-$

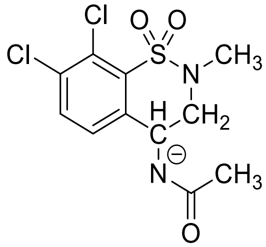

E=-2079.2436737

C	1.982904	-0.308673	0.060434
C	0.623929	0.396849	0.116976
C	-0.623083	-0.230272	-0.029549
S	-0.700344	-2.020824	-0.229841
N	0.820631	-2.512972	0.186693
C	1.857856	-1.722499	-0.499365

C	-1.817594	0.510779	-0.046509
C	-1.768581	1.887039	0.154020
C	-0.541452	2.520094	0.348889
C	0.629527	1.784463	0.316535
Cl	-3.345239	-0.258578	-0.343621
Cl	-3.215831	2.850802	0.162718
O	-1.616468	-2.587691	0.753010
O	-0.905531	-2.298988	-1.647232
C	1.042176	-2.726138	1.624813
N	2.936325	0.410692	-0.761157
C	3.771504	1.196486	-0.092230
C	4.829099	1.890596	-0.940644
O	3.777254	1.434866	1.148949
H	-0.516903	3.593294	0.511902
H	1.585173	2.280246	0.455165
H	2.339128	-0.362793	1.101900
H	0.869526	-1.825119	2.227048
H	0.392276	-3.526263	1.977562
H	2.084247	-3.034431	1.739084
H	4.740257	2.976450	-0.819692
H	5.825494	1.605475	-0.583605
H	4.740242	1.637325	-2.000305
H	2.806710	-2.254354	-0.386339
H	1.619098	-1.686336	-1.566697

VIII^{2-} + *t*-BuOH

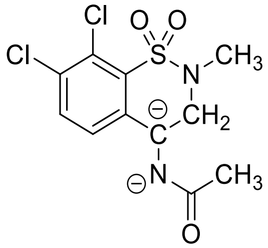

E=-2312.2910731

C	-0.006208	-0.015598	-0.002726
C	-0.003136	-0.004534	1.397389
C	1.217584	0.005155	2.087571
C	2.406708	-0.178793	1.354612
C	2.374888	-0.210188	-0.041576
C	1.169952	-0.092929	-0.728041

C	-1.351316	-0.117483	2.056446
C	-1.600807	-1.299588	2.671097
S	1.292764	0.438445	3.901766
N	0.050317	1.276478	4.306748
C	-1.031076	0.670133	5.065417
Cl	3.939366	-0.456725	2.136960
Cl	3.832957	-0.404002	-0.987562
O	1.447073	-0.869143	4.591669
O	2.484759	1.314595	3.908083
N	-2.165027	0.980986	1.828854
C	-3.447826	0.978713	2.234736
O	-4.077860	0.051843	2.788854
C	-4.187972	2.291742	1.999549
O	-1.372569	3.233895	0.361335
C	-0.591392	4.153815	1.112520
C	-1.306239	4.513942	2.418694
C	0.776798	3.547644	1.429122
C	-0.436017	5.391499	0.233945
H	1.163382	-0.101450	-1.813482
H	-0.960884	0.022997	-0.520588
H	-0.847658	-0.381317	5.323027
H	-1.161193	1.225174	6.006298
H	-1.978780	0.722255	4.514125
H	-5.208793	2.081808	1.664114
H	-4.259373	2.830260	2.953496
H	-3.681305	2.935215	1.277074
H	-2.556202	-1.520385	3.125784
H	-0.799996	-2.031498	2.745516
H	-1.569055	2.424807	0.915911
H	-0.719974	5.236435	2.999771
H	-2.289700	4.952101	2.209983
H	-1.442732	3.617272	3.035328
H	1.432085	4.279009	1.918631
H	0.665423	2.695330	2.107485
H	1.262581	3.204242	0.507174
H	0.153437	6.163019	0.742468
H	0.069618	5.130516	-0.703231
H	-1.419058	5.810199	-0.011833

TS1

E=-2079.1681609

n_{imag}=1

C	-0.894756	2.366837	0.222114
C	0.016950	1.292804	0.035950
C	-0.536199	-0.023995	-0.067585
C	-1.928979	-0.195486	-0.163585
C	-2.782813	0.898014	-0.068151
C	-2.255112	2.176929	0.165118
C	1.424682	1.502228	0.031582
C	2.070996	2.720475	0.646146
S	0.497854	-1.496251	0.200013
N	1.937328	-0.973160	0.821473
C	1.861452	-0.446580	2.171680
Cl	-2.620372	-1.765168	-0.477410
Cl	-4.510398	0.741689	-0.262052
O	-0.185909	-2.297445	1.227539
O	0.785799	-2.141820	-1.081353
N	2.125618	0.423431	-0.335282
C	3.394012	0.394836	-0.866147
O	3.818743	1.350858	-1.511488
C	4.196266	-0.875074	-0.722240
H	-2.934814	3.018057	0.266089
H	-0.501588	3.369331	0.364802
H	1.438557	0.572700	2.189209
H	1.238539	-1.083040	2.808043
H	2.877148	-0.414360	2.578014
H	3.588791	-1.747163	-0.972826
H	5.059482	-0.802982	-1.386815
H	4.541689	-1.002463	0.308582
H	3.096551	2.483853	0.947915
H	2.132744	3.561639	-0.058018
H	1.531642	3.073034	1.535432

TS2

E=-2079,2152398

n_{imag}=1

C	0.730563	0.194234	-0.019038
C	2.097298	-0.092238	-0.050941
C	2.514143	-1.427587	-0.047157
C	1.581552	-2.459410	-0.004776
C	0.226851	-2.161716	0.063030
C	-0.206629	-0.835940	0.076495
Cl	3.278834	1.178105	-0.070332
Cl	4.204625	-1.838446	-0.100771
C	-1.673652	-0.575087	0.302128
C	-2.020568	-0.159511	1.716210
S	0.065615	1.876296	-0.095983
O	0.259691	2.491013	1.239551
N	-2.492886	-1.158908	-0.522556
C	-3.836255	-1.335202	-0.230779
O	-4.269364	-1.749638	0.838724
C	-4.747327	-1.087341	-1.410697
N	-1.421407	1.461323	-0.429926
C	-2.471141	2.362207	0.011287
O	0.768860	2.578640	-1.187506
H	1.924270	-3.489323	-0.011934
H	-0.504550	-2.963104	0.118895
H	-2.365776	2.672360	1.061679
H	-2.537784	3.268221	-0.608157
H	-3.425256	1.828729	-0.091809
H	-4.538482	-0.107246	-1.851887
H	-4.546312	-1.837592	-2.183829
H	-5.793598	-1.151080	-1.105778
H	-3.014557	0.289692	1.771381
H	-2.029932	-1.057900	2.344925
H	-1.279124	0.540831	2.107637

TS3

E=-2312.2737222

n_{imag}=1

C	-1.775077	2.333922	-0.558666
C	-0.405193	2.462875	-0.752647
C	0.403592	1.341692	-0.645599
C	-0.140834	0.096387	-0.311852
C	-1.508687	-0.020230	-0.019920
C	-2.333232	1.101894	-0.202446
C	0.821808	-1.062556	-0.413440
C	1.975762	-1.037979	0.469227
S	-2.162279	-1.611272	0.682767
O	-3.163827	-1.152514	1.674690
Cl	-4.066915	0.996607	-0.073947
Cl	-2.767539	3.757283	-0.775720
N	0.652846	-1.812496	-1.452691
C	1.431614	-2.928771	-1.692774
O	2.267265	-2.974894	-2.586728
C	1.064853	-4.148814	-0.875231
O	-2.691984	-2.340244	-0.485194
N	-0.956100	-2.364309	1.323061
C	-0.563460	-1.853040	2.631000
O	3.597564	0.830278	-0.510359
C	4.276989	1.520388	0.482771
C	5.288195	2.476194	-0.169262
C	3.300636	2.348732	1.343258
C	5.043520	0.547992	1.402424
H	0.013776	3.430314	-1.013236
H	1.476908	1.412981	-0.829095
H	-0.315141	-0.776584	2.629636
H	-1.333117	-2.005094	3.401669
H	0.339606	-2.389671	2.942341
H	1.006754	-3.893855	0.187059
H	0.063889	-4.482623	-1.171815
H	1.786724	-4.950144	-1.047615
H	2.540008	-1.977332	0.481312
H	2.793436	-0.113448	-0.002943
H	1.725705	-0.709352	1.481949
H	5.854574	3.055778	0.573383
H	5.998271	1.905261	-0.780226
H	4.763289	3.177585	-0.829805

H	3.810865	2.893643	2.149550
H	2.774674	3.079377	0.715126
H	2.546017	1.693423	1.796678
H	5.623277	1.072283	2.174491
H	4.346943	-0.134390	1.905522
H	5.733456	-0.059390	0.803188

TS4

E=-2079.1863185

n_{imag}=1

C	-1.192994	-2.573707	-0.109250
C	0.116747	-2.147563	-0.229039
C	0.417880	-0.776512	-0.294953
C	-0.630056	0.158936	-0.075429
C	-1.963171	-0.271982	-0.026249
C	-2.234762	-1.642157	-0.063593
C	1.747329	-0.246915	-0.547194
N	2.791161	-0.638387	0.346082
C	3.966546	-1.219078	-0.003909
C	4.943780	-1.424117	1.136888
S	-0.021169	1.749680	0.639518
O	-1.182783	2.579499	0.985411
Cl	-3.308195	0.836351	0.036707
Cl	-3.873029	-2.251779	-0.067928
C	1.829321	0.944913	-1.267493
O	0.828894	1.284704	1.749398
N	0.938451	2.459711	-0.442844
C	0.202916	3.143677	-1.500632
O	4.259602	-1.549541	-1.152276
H	-1.432116	-3.633032	-0.101223
H	0.920981	-2.873041	-0.326537
H	2.677576	-0.363852	1.319354
H	-0.490849	2.479149	-2.047090
H	-0.374704	3.989026	-1.112308
H	0.937966	3.524406	-2.217371
H	5.376838	-2.424384	1.057241
H	5.756533	-0.697073	1.035126
H	4.487341	-1.301643	2.122602
H	1.175901	1.029893	-2.136788
H	2.809856	1.398403	-1.392348

TS5

E=-2078.6533059

n_{imag}=1

C	-1.725909	-1.888899	0.054929
C	-0.510224	-2.546605	0.330430
C	0.680763	-1.863033	0.276410
C	0.706974	-0.486816	-0.077458
C	-0.549324	0.222188	-0.106424
C	-1.750588	-0.509891	-0.118611
C	1.900364	0.247735	-0.311344
C	1.712477	1.435090	-1.031147
S	-0.453692	1.888743	0.680183
N	0.569659	2.816969	-0.164350
C	-0.073011	3.483227	-1.289160
Cl	-3.303969	0.249912	-0.418673
Cl	-3.167025	-2.878011	-0.096615
N	3.151327	-0.049811	0.295849
C	3.811922	-1.106135	-0.154215
O	3.478208	-1.915346	-1.068409
C	5.145677	-1.371700	0.542869
O	-1.810298	2.470882	0.709458
O	0.172571	1.604933	1.984637
H	-0.528063	-3.612483	0.541189
H	1.621866	-2.379326	0.434337
H	-0.587944	2.787796	-1.977796
H	-0.811140	4.221728	-0.958149
H	0.709687	3.999993	-1.856032
H	5.101073	-2.335813	1.064395
H	5.943398	-1.446035	-0.205629
H	5.397223	-0.590049	1.264639
H	0.976151	1.399539	-1.836136
H	2.578258	2.067614	-1.217495

Frequency calculation for gas-phase acidity was performed at M06-2X/6-31+G* using fine grid:

1a

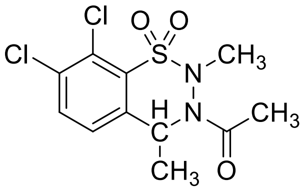

E=-2079.6818009

C	0.013346	-0.029703	-0.006280
C	0.013098	0.007869	1.515184
C	1.162812	-0.019608	2.318090
S	2.779499	0.123104	1.538127
N	2.337985	0.796246	0.055192
N	1.367108	0.025257	-0.557114
C	1.080708	-0.136163	3.714972
C	-0.175738	-0.166817	4.321975
C	-1.325410	-0.102423	3.538889
C	-1.228028	-0.025851	2.160021
Cl	2.502391	-0.285783	4.679079
Cl	-0.342200	-0.297332	6.038551
O	3.571109	1.139358	2.203287
O	3.293669	-1.207101	1.283778
C	2.125399	2.250798	0.041032
C	1.692096	-0.730799	-1.676018
C	3.129765	-0.681119	-2.125899
C	-0.900374	1.015949	-0.661040
O	0.826281	-1.382358	-2.229760
H	-2.295331	-0.134154	4.024067
H	-2.139045	-0.018489	1.570519
H	-0.356508	-1.019717	-0.295718
H	1.313453	2.566885	0.708419
H	3.051121	2.735613	0.347564
H	1.885285	2.522017	-0.989271
H	3.448975	0.347278	-2.315776
H	3.779587	-1.090653	-1.347301
H	3.210347	-1.273878	-3.036583
H	-1.950314	0.839038	-0.414979
H	-0.642342	2.032135	-0.351441
H	-0.795314	0.931895	-1.746111

I⁻

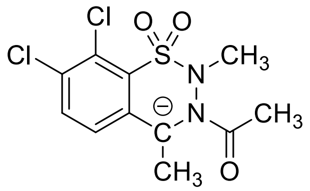

E=-2079.1307768

C	0.002314	0.009011	-0.012224
C	0.005078	0.001038	1.359275
C	1.200054	0.014535	2.206109
S	2.743479	0.072464	1.382870
N	2.278778	0.810372	-0.103414
N	1.261824	0.051934	-0.689447
C	1.112388	-0.018188	3.608685
C	-0.112917	0.000861	4.248822
C	-1.290562	0.046216	3.461402
C	-1.242866	0.048010	2.099619
Cl	2.560559	-0.142037	4.571935
Cl	-0.276754	-0.083176	5.988387
O	3.688075	1.005596	1.993709
O	3.216938	-1.254834	0.998333
C	1.932722	2.228980	0.020424
C	1.603398	-0.800009	-1.717566
C	3.008708	-0.667658	-2.267809
C	-1.233518	0.129346	-0.857166
O	0.790928	-1.588808	-2.179489
H	-2.249833	0.063394	3.972162
H	-2.172668	0.054767	1.540257
H	1.026400	2.386021	0.619294
H	2.777587	2.740401	0.481468
H	1.772472	2.608775	-0.992609
H	3.264251	0.375239	-2.473247
H	3.725931	-1.044290	-1.533581
H	3.057941	-1.260943	-3.182206
H	-1.890803	0.942300	-0.510519
H	-0.952823	0.345223	-1.891133
H	-1.827065	-0.795128	-0.880124

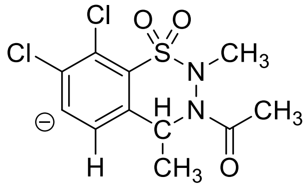

E=-2079.0950682

C	0.019765	-0.058017	-0.006569
C	0.026905	0.024384	1.516251
C	1.185124	-0.035368	2.315425
S	2.778932	0.035252	1.534073
N	2.354075	0.768600	0.051811
N	1.369363	0.028455	-0.584374
C	1.070461	-0.100875	3.715275
C	-0.198857	-0.033008	4.285765
C	-1.391273	0.048207	3.578246
C	-1.203988	0.055402	2.183818
Cl	2.518154	-0.321992	4.676798
Cl	-0.279340	-0.064970	6.076442
O	3.667320	1.000159	2.165792
O	3.265275	-1.295418	1.197570
C	2.086028	2.209562	0.128684
C	1.698535	-0.690599	-1.709881
C	3.142784	-0.626679	-2.151778
C	-0.912940	0.950347	-0.691536
O	0.848832	-1.336741	-2.305616
H	-2.094542	0.064250	1.551706
H	-0.326117	-1.062239	-0.274179
H	1.236404	2.445424	0.782532
H	2.981232	2.700149	0.509344
H	1.875960	2.549048	-0.888984
H	3.458343	0.406744	-2.319901
H	3.788308	-1.044412	-1.374287
H	3.229783	-1.203363	-3.073062
H	-1.956688	0.753399	-0.438203
H	-0.680508	1.976515	-0.392428
H	-0.799438	0.852597	-1.775674

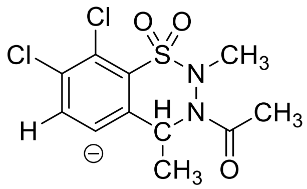

E=-2079.0943152

C	0.000000	0.000000	0.000000
C	0.000000	0.000000	1.527863
C	1.149884	0.000000	2.341123
S	2.781337	0.171128	1.560831
N	2.341044	0.825003	0.043938
N	1.358424	0.052028	-0.565670
C	1.059969	-0.137709	3.742246
C	-0.203239	-0.218322	4.341883
C	-1.351180	-0.189375	3.548672
C	-1.245386	-0.091332	2.168843
Cl	2.486265	-0.246995	4.727996
Cl	-0.387075	-0.371483	6.069107
O	3.567122	1.211629	2.222611
O	3.319285	-1.167434	1.325989
C	2.199368	2.291871	-0.041586
C	1.688178	-0.752886	-1.657248
C	3.122864	-0.716522	-2.132971
C	-0.920238	1.067637	-0.629319
O	0.821721	-1.440650	-2.182094
H	-2.324466	-0.263318	4.022395
H	-0.377805	-0.977801	-0.317624
H	1.384032	2.686006	0.577383
H	3.138456	2.746378	0.273444
H	2.011961	2.530261	-1.091274
H	3.455599	0.304467	-2.345141
H	3.788774	-1.125147	-1.366605
H	3.185304	-1.322021	-3.038538
H	-1.967565	0.893381	-0.364717
H	-0.655206	2.080490	-0.312730
H	-0.840641	1.001686	-1.718377

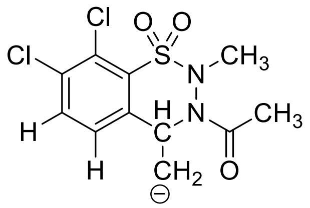

E=-2079.0648397

C	0.000000	0.000000	0.000000
C	0.000000	0.000000	1.527863
C	1.149884	0.000000	2.341123
S	2.781337	0.171128	1.560831
N	2.341044	0.825003	0.043938
N	1.358424	0.052028	-0.565670
C	1.059969	-0.137709	3.742246
C	-0.203239	-0.218322	4.341883
C	-1.351180	-0.189375	3.548672
C	-1.245386	-0.091332	2.168843
Cl	2.486265	-0.246995	4.727996
Cl	-0.387075	-0.371483	6.069107
O	3.567122	1.211629	2.222611
O	3.319285	-1.167434	1.325989
C	2.199368	2.291871	-0.041586
C	1.688178	-0.752886	-1.657248
C	3.122864	-0.716522	-2.132971
C	-0.920238	1.067637	-0.629319
O	0.821721	-1.440650	-2.182094
H	-2.324466	-0.263318	4.022395
H	-2.153356	-0.109475	1.575022
H	-0.377805	-0.977801	-0.317624
H	1.384032	2.686006	0.577383
H	3.138456	2.746378	0.273444
H	2.011961	2.530261	-1.091274
H	3.455599	0.304467	-2.345141
H	3.788774	-1.125147	-1.366605
H	3.185304	-1.322021	-3.038538
H	-0.655206	2.080490	-0.312730
H	-0.840641	1.001686	-1.718377

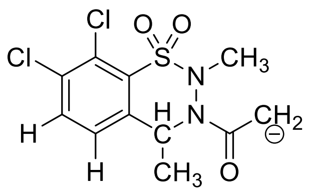

E=-2079.0986081

C	0.124135	-0.141636	0.097617
C	0.011018	-0.081968	1.608176
C	1.107569	0.017625	2.474644
S	2.763196	0.254299	1.780256
N	2.393973	0.648595	0.221796
N	1.512375	-0.285132	-0.316476
C	0.937355	-0.023234	3.867759
C	-0.348311	-0.113859	4.397876
C	-1.450850	-0.181160	3.548721
C	-1.266118	-0.170395	2.177647
Cl	2.301532	0.008880	4.932825
Cl	-0.623539	-0.156371	6.116905
O	3.343534	1.451633	2.381295
O	3.462744	-1.012118	1.868937
C	2.282095	2.074968	-0.112782
C	1.699498	-0.657890	-1.743670
C	2.886898	-0.297816	-2.333210
C	-0.659371	0.997483	-0.588337
O	0.693852	-1.251592	-2.219598
H	-2.444293	-0.259698	3.978619
H	-2.129174	-0.254442	1.522909
H	-0.335346	-1.078272	-0.233394
H	1.549683	2.605264	0.508822
H	3.258615	2.544947	0.010094
H	1.996437	2.100954	-1.166544
H	3.700253	0.143692	-1.772422
H	3.059235	-0.622229	-3.352456
H	-1.739056	0.839953	-0.484724
H	-0.422195	1.980813	-0.172732
H	-0.417619	0.972219	-1.654559

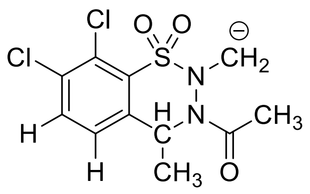

E=-2079.076127

C	0.039063	-0.093208	0.037635
C	0.009428	-0.029388	1.558364
C	1.150076	-0.038635	2.374901
S	2.780444	0.083595	1.563460
N	2.349430	0.781347	0.062498
N	1.403124	-0.103552	-0.473525
C	1.033784	-0.124368	3.768999
C	-0.232035	-0.147300	4.355333
C	-1.370631	-0.101940	3.557459
C	-1.244403	-0.052430	2.179285
Cl	2.437840	-0.250886	4.774991
Cl	-0.436440	-0.248085	6.083512
O	3.590699	1.047087	2.292218
O	3.283500	-1.282924	1.396665
C	2.106492	2.201593	-0.078672
C	1.671945	-0.717421	-1.665428
C	3.076388	-0.543512	-2.186793
C	-0.823335	0.978600	-0.646862
O	0.809948	-1.365178	-2.254022
H	-2.348403	-0.124623	4.027697
H	-2.142153	-0.052385	1.568614
H	-0.365086	-1.075312	-0.236165
H	1.411571	2.562721	0.704873
H	3.071998	2.699875	0.048715
H	3.288340	0.522804	-2.312081
H	3.797731	-0.940911	-1.467192
H	3.148777	-1.072504	-3.138067
H	-1.876758	0.880237	-0.362131
H	-0.454562	1.976804	-0.410120
H	-0.745306	0.831134	-1.727432

H_2O

E=-75.7523279

O	0.002039	0.000000	0.001553
H	-0.000718	-0.000000	0.967620
H	0.932611	-0.000000	-0.257926

OH^-

E=-75.7523279

O	0.000000	0.000000	0.001647
H	0.000000	0.000000	0.973227

MeOH

E=-115.6609312

C	0.000070	-0.000000	0.002523
O	0.001313	0.000000	1.422005
H	0.906690	-0.000000	1.751224
H	-0.523712	-0.890210	-0.358823
H	-0.523712	0.890210	-0.358823
H	1.013002	-0.000000	-0.413393

MeO^-

E=-115.0458769

C	-0.000043	0.000139	-0.002304
O	0.000023	-0.000141	1.334566
H	1.025462	-0.000020	-0.491240
H	-0.513063	-0.887773	-0.491136
H	-0.512768	0.888438	-0.490941

t-BuOH

E=-233.560657

C	-0.003353	-0.000001	0.007977
C	0.002976	-0.000001	1.531408
O	1.386275	-0.000001	1.894183
C	-0.672060	-1.258353	2.077316
C	-0.672059	1.258353	2.077315

H	-1.029051	-0.000001	-0.374258
H	0.514134	-0.887355	-0.369439
H	-1.723062	-1.306180	1.772269
H	0.514135	0.887352	-0.369440
H	-0.640916	-1.271011	3.174206
H	-0.157988	-2.151099	1.707949
H	-0.640915	1.271012	3.174205
H	-0.157985	2.151098	1.707947
H	-1.723060	1.306181	1.772268
H	1.453945	-0.000001	2.860597

t-BuO$^-$

E=-232.9543475

C	-0.004841	-0.000215	0.010064
C	-0.004309	0.000163	1.570567
O	1.240000	0.000475	2.069534
C	-0.813246	-1.253841	2.026479
C	-0.813850	1.254178	2.025584
H	-1.012918	0.000134	-0.438166
H	0.539057	-0.887201	-0.340144
H	-1.844463	-1.288405	1.636007
H	0.539715	0.886314	-0.340341
H	-0.848187	-1.267403	3.123649
H	-0.278327	-2.154131	1.697019
H	-0.849835	1.268299	3.122741
H	-0.278951	2.154584	1.696361
H	-1.844713	1.288242	1.634155

AcOH

E=-228.9820965

C	0.001312	0.000000	0.001645
C	0.002190	0.000000	1.517701
O	1.210085	-0.000000	2.130911
O	-1.001584	0.000000	2.173663
H	0.998068	-0.000000	-0.445678
H	-0.544271	-0.881952	-0.341892
H	-0.544271	0.881952	-0.341892
H	1.931393	-0.000000	1.486280

AcO$^-$

E=-228.4341997

C	0.000674	-0.000000	0.001819
C	0.000438	0.000000	1.559417
O	1.124940	0.000000	2.115411
O	-1.139083	-0.000000	2.086382
H	1.017902	0.000000	-0.401840
H	-0.539935	-0.882032	-0.362440
H	-0.539935	0.882032	-0.362440

***trans*-3b** and ***cis*-3b** were calculated at B3LYP[7]/6-31G (pcm[8,9]: DMSO)

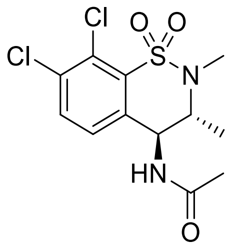

***trans*-3b**

E=-2118.9937322

C	0.037409	0.183665	-0.034060
C	0.042526	0.068245	1.368455
C	1.300529	-0.009517	1.989493
C	2.497230	0.093328	1.270276
C	2.446429	0.210038	-0.116728
C	1.215716	0.244415	-0.771027
S	1.391194	-0.213322	3.870205
N	-0.332555	-0.586533	4.322782
C	-1.253517	0.342849	3.607924
C	-1.315018	0.014176	2.090277
N	-2.280750	0.878727	1.413367
C	-3.472652	0.403459	0.925809
O	-3.776954	-0.814854	0.995123
O	2.284808	-1.517814	4.290996
O	1.774774	1.218641	4.564386
C	-0.638691	-2.032967	4.393522
Cl	4.095437	0.092751	2.114758
Cl	3.953676	0.323451	-1.113312
C	-4.399245	1.433740	0.324352
H	1.182871	0.328822	-1.849549
H	-0.912275	0.238370	-0.549216
H	-1.722787	-0.998627	1.975532
H	-0.844538	-2.498445	3.422201
H	0.206089	-2.539327	4.859714
H	-1.509772	-2.144225	5.042683
H	-4.833361	1.033173	-0.594946
H	-5.222398	1.630508	1.020787
H	-3.899374	2.379683	0.103576
C	-2.629309	0.296567	4.285088
H	-0.834581	1.347059	3.747570
H	-2.080852	1.868564	1.352938
H	-2.508839	0.409942	5.365664

H	-3.250097	1.118008	3.919635
H	-3.152068	-0.642644	4.081462

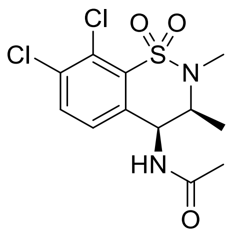

***cis*-3b**

E=-2118.9920237

C	0.103790	0.478742	-0.053957
C	0.086540	0.185029	1.320200
C	1.326881	-0.066854	1.931538
C	2.533060	-0.011725	1.223691
C	2.505390	0.283555	-0.138537
C	1.291771	0.526848	-0.778239
S	1.399437	-0.469045	3.785364
N	-0.348551	-0.454743	4.326774
C	-1.192090	0.501689	3.552529
C	-1.257313	0.098658	2.051431
N	-2.303101	0.848063	1.362730
C	-3.464099	0.249308	0.936094
O	-3.660156	-0.984679	1.073037
O	1.966886	-1.987889	4.023674
O	2.175923	0.733890	4.576685
C	-0.896321	-1.805914	4.590414
Cl	4.114066	-0.326740	2.040011
Cl	4.024563	0.356546	-1.121374
C	-4.491372	1.167194	0.316628
H	1.278089	0.752773	-1.836646
H	-0.833525	0.672862	-0.557711
H	-1.598795	-0.943829	2.005406
H	-1.209235	-2.347942	3.689752
H	-0.149307	-2.397208	5.117605
H	-1.759417	-1.661402	5.245793
H	-4.927500	0.678796	-0.558034
H	-5.300021	1.344628	1.034918
H	-4.075448	2.132740	0.018806
H	-2.196992	0.343920	3.960100

C	-0.793180	1.959037	3.812071
H	-2.197973	1.847348	1.252383
H	-0.761683	2.140877	4.888955
H	0.185295	2.215026	3.396852
H	-1.537009	2.632662	3.375815

5o

E=-1160.584351

C	-0.051277	-0.066852	-0.092053
C	-0.118922	-0.129319	1.302261
C	1.037260	-0.101805	2.079688
C	2.288209	-0.027542	1.470422
C	2.381284	0.007790	0.081245
C	1.217462	-0.013805	-0.684728
S	1.410284	0.008462	-2.465117
O	2.806870	-0.331811	-2.736441
C	-1.331136	-0.075248	-0.870933
N	-1.942137	-1.346406	-0.951703
C	-3.258519	-1.704950	-0.813840
C	-4.259704	-0.661099	-0.385163
N	0.431183	-1.246609	-2.946723
C	-0.507527	-1.010841	-4.051117
O	0.860633	1.244842	-3.011707
C	-1.846045	1.036087	-1.399390
O	-3.591528	-2.868170	-1.016935
H	-1.095433	-0.193074	1.776607
H	0.958394	-0.140606	3.162301
H	3.192690	-0.009253	2.070750
H	3.347916	0.038055	-0.411278
H	0.995451	-2.088378	-3.065321
H	-1.112292	-1.915091	-4.149410
H	-1.158721	-0.174921	-3.792369
H	-0.006292	-0.802245	-5.001990
H	-2.766108	1.023910	-1.975986
H	-1.341886	1.987038	-1.264318
H	-1.317883	-2.123805	-1.156674
H	-3.823800	0.106064	0.258394
H	-4.684409	-0.170124	-1.266935
H	-5.067127	-1.176837	0.137789

Io$^+$

E=-1161.014911

C	0.010679	0.098132	0.027007
C	0.031700	0.053905	1.420959
C	1.249183	0.004412	2.101188

C	2.442944	-0.063134	1.360970
C	2.420505	-0.059112	-0.026242
C	1.198691	0.045363	-0.693943
C	1.198227	0.031823	3.582025
C	0.401868	-0.990723	4.302508
S	4.029612	-0.251324	2.170607
O	4.345248	0.971100	2.915532
N	3.637371	-1.385274	3.316197
C	3.513473	-2.774760	2.849015
O	4.973867	-0.736746	1.174889
N	1.826491	1.007685	4.179922
C	2.002954	1.208743	5.614843
O	1.468217	0.470001	6.392319
C	2.894615	2.365715	5.917123
H	3.350608	-0.154687	-0.578106
H	1.182237	0.060082	-1.779099
H	-0.941553	0.159455	-0.490216
H	-0.897929	0.079710	1.983108
H	2.342884	1.665713	3.589203
H	2.518491	3.269027	5.424678
H	2.939456	2.513310	6.995709
H	3.898903	2.162715	5.526435
H	4.260572	-1.278496	4.120704
H	3.173758	-3.370980	3.697587
H	2.754681	-2.825886	2.062662
H	4.458327	-3.174427	2.470230
H	-0.057803	-1.674727	3.590233
H	1.064408	-1.550247	4.973670
H	-0.358453	-0.517271	4.931998

TS between **Io⁺** and **IIo⁺**

E=-1161.011215

n_{imag}=1

C	-2.816925	0.382303	-0.685777
C	-3.246034	1.700852	-0.544564
C	-2.381099	2.675778	-0.046256
C	-1.074550	2.353524	0.324121
C	-0.632124	1.041595	0.178249
C	-1.510290	0.084284	-0.327478
C	0.767949	0.655338	0.561636
C	1.208711	0.958236	1.959481
S	-0.840252	-1.547300	-0.466605
N	0.262853	-1.370936	0.833635
C	-0.289513	-1.714929	2.159838
N	1.629838	0.654406	-0.466797
C	3.044721	0.459216	-0.419620
O	3.608507	0.273670	0.629651
C	3.688551	0.516419	-1.770128
O	-1.862797	-2.536399	-0.181122
O	-0.000143	-1.668666	-1.650872

H	-3.484557	-0.394208	-1.048325
H	-4.264879	1.964334	-0.812895
H	-2.729514	3.697387	0.067869
H	-0.407321	3.112063	0.725773
H	1.230415	0.702903	-1.403322
H	4.755796	0.327879	-1.665248
H	3.239864	-0.231530	-2.434203
H	3.527769	1.504086	-2.216423
H	1.115303	-1.905580	0.619060
H	0.501544	-1.568535	2.896736
H	-1.127661	-1.051907	2.384135
H	-0.622622	-2.754928	2.182327
H	0.342613	0.966330	2.622184
H	1.966466	0.264694	2.319181
H	1.654339	1.962630	1.947993

IIo$^+$

E=-1161.029160

C	-0.008063	0.024232	0.010188
C	-0.002421	-0.017532	1.398685
C	1.219863	-0.014229	2.061843
C	2.450335	0.022196	1.420152
C	2.427924	0.067368	0.027124
C	1.214527	0.067829	-0.664437
S	1.015552	-0.070627	3.809785
N	-0.733680	-0.608608	3.622892
C	-1.230201	0.007076	2.296517
C	-0.865588	-2.090329	3.750316
N	-1.550708	1.396696	2.576306
C	-2.335163	1.800618	3.615976
C	-2.614142	3.270975	3.714479
C	-2.427833	-0.754792	1.752302
O	1.737852	-1.159061	4.425635
O	0.948488	1.231690	4.434098
O	-2.743504	0.986413	4.448107
H	3.384397	0.010254	1.974094
H	3.364101	0.094789	-0.520915
H	1.218860	0.098510	-1.749648
H	-0.941977	0.021541	-0.543866
H	-1.154390	2.098310	1.958173
H	-2.339748	3.815680	2.809628
H	-3.677715	3.409508	3.923203
H	-2.047928	3.671359	4.561938
H	-1.306224	-0.161364	4.372934
H	-1.927964	-2.333131	3.741389
H	-0.345019	-2.578983	2.925419
H	-0.429712	-2.382612	4.704849
H	-2.160423	-1.770619	1.454736
H	-3.234562	-0.778156	2.489257
H	-2.782742	-0.212850	0.872101

2o

E=-1160.614135

C	0.005699	0.001276	-0.003233
C	0.003814	-0.000907	1.385808
C	1.157059	0.001269	2.151696
C	2.394115	-0.000726	1.513591
C	2.427616	0.004192	0.119267
C	1.247672	0.008700	-0.633776
S	-1.411402	-0.077177	2.440491
N	-0.518006	0.327452	3.803163
C	0.921159	-0.014136	3.657624
N	1.232634	-1.365673	4.119780
C	1.463724	-1.769426	5.407815
O	1.542253	-1.000172	6.356805
C	1.754651	1.063249	4.350550
O	-2.385278	0.973523	2.156826
O	-1.928754	-1.447359	2.513829
C	-1.178827	-0.006425	5.064920
C	1.588911	-3.267359	5.575775
H	-0.920123	0.001966	-0.571399
H	1.298806	0.018656	-1.718212
H	3.386100	0.007893	-0.391334
H	3.314633	-0.003236	2.090958
H	1.093177	-2.106051	3.439802
H	1.972062	-3.763455	4.680490
H	2.243634	-3.475906	6.423660
H	0.595982	-3.674389	5.799088
H	-1.212610	-1.088354	5.240773
H	-0.634948	0.476101	5.878306
H	-2.197719	0.389729	5.043941
H	1.461722	1.183941	5.392451
H	2.813485	0.789559	4.321608
H	1.610355	2.006167	3.817072

conformer of **5a**, structure was obtained from the scan calculations

E= -2079.704733

C	-0.008283	-0.000614	-0.064469
C	-0.039985	-0.209636	1.321363
C	1.164069	-0.307343	2.021858
C	2.378715	-0.128611	1.380703
C	2.399965	0.190529	0.026723
C	1.212161	0.269384	-0.708161
C	-1.297843	-0.223888	2.132283
C	-1.989006	0.902010	2.327096
S	-1.539764	-0.135347	-1.030967
O	-2.238889	1.142291	-0.987662
O	-1.187614	-0.740083	-2.309769
N	-2.408591	-1.256685	-0.175876

C	-3.796309	-0.943142	0.188466
N	-1.606285	-1.463913	2.722864
C	-2.064182	-1.755513	3.985383
O	-2.372591	-2.910045	4.257889
C	-2.141608	-0.651614	5.009789
H	1.138216	-0.497316	3.091823
H	3.312007	-0.196103	1.930600
Cl	3.937683	0.495050	-0.719642
Cl	1.291197	0.772324	-2.362696
H	-2.305165	-2.166170	-0.626579
H	-4.137360	-1.739422	0.854287
H	-3.821937	0.004007	0.727297
H	-4.456676	-0.885744	-0.682436
H	-2.895550	0.919494	2.923795
H	-1.648901	1.833938	1.887693
H	-1.499173	-2.279987	2.124698
H	-1.377712	0.115348	4.864383
H	-3.124352	-0.170802	4.964070
H	-2.032459	-1.109273	5.994565

Ia$^+$

E= -2080.132097

C	0.031841	0.259228	0.116888
C	0.149683	0.102638	1.491514
C	1.408268	-0.073418	2.061423
C	2.545880	-0.135506	1.247772
C	2.439376	0.057655	-0.132545
C	1.165007	0.251607	-0.688183
C	1.467953	-0.126872	3.555284
C	0.741571	-1.211505	4.264896
S	4.116049	-0.477795	2.069716
O	4.596139	0.764248	2.665189
N	3.513141	-1.398786	3.326892
C	3.297848	-2.825789	3.035113
O	4.970462	-1.267744	1.205880
N	1.924539	0.955881	4.142309
C	2.024968	1.220670	5.570689
O	1.618594	0.415719	6.361170
C	2.657699	2.540338	5.862864
Cl	3.827635	0.097293	-1.152775
Cl	0.969526	0.479174	-2.390245
H	-0.944774	0.399996	-0.334714
H	-0.737058	0.134071	2.117468
H	2.311819	1.686715	3.540714
H	2.087188	3.340948	5.379106
H	2.678734	2.696219	6.940970
H	3.677055	2.560187	5.460507
H	4.076433	-1.233259	4.167395
H	2.849603	-3.273613	3.923406
H	2.599708	-2.918374	2.198053

H	4.231968	-3.338773	2.792384
H	0.540989	-2.030430	3.574220
H	1.288621	-1.562835	5.139902
H	-0.212155	-0.800001	4.624018

IIa⁺

E= -2080.152661

C	-0.005795	0.013314	0.007742
C	0.002871	0.001152	1.395480
C	1.219661	-0.010084	2.062997
C	2.448736	0.004643	1.413602
C	2.424503	0.012680	0.014502
C	1.209238	0.015047	-0.672460
S	1.011216	-0.058183	3.819239
N	-0.746641	-0.549631	3.617494
C	-1.223111	0.072614	2.291941
N	-1.496925	1.473321	2.565458
C	-2.268675	1.905732	3.604511
C	-2.532257	3.379848	3.675466
C	-2.443243	-0.653050	1.748133
O	1.686838	-1.174034	4.432290
O	0.990426	1.251127	4.426121
C	-0.916370	-2.029336	3.738734
Cl	3.915383	0.021101	2.311375
Cl	3.900229	0.020550	-0.881021
O	-2.676753	1.112299	4.456928
H	1.221224	0.017663	-1.757791
H	-0.938042	0.021147	-0.548393
H	-1.117525	2.157966	1.917935
H	-2.283974	3.898776	2.747823
H	-3.586922	3.532813	3.916453
H	-1.934953	3.796246	4.493289
H	-1.316205	-0.085607	4.364319
H	-1.986382	-2.236428	3.719645
H	-0.405679	-2.525084	2.912058
H	-0.497316	-2.336816	4.695698
H	-2.210562	-1.678848	1.456376
H	-3.251660	-0.644274	2.483932
H	-2.779538	-0.103828	0.865090

isomer of **2a**

E= -2079.743264

C	0.001775	-0.005684	-0.001929
C	0.001069	-0.002371	1.387897
C	1.161286	-0.002132	2.140209
C	2.394848	0.006235	1.497098
C	2.426393	0.022373	0.107238
C	1.244350	0.017165	-0.639256

S	-1.414219	-0.094020	2.456881
N	-0.500214	0.290809	3.806962
C	0.934506	-0.054637	3.646795
N	1.238427	-1.419756	4.066708
C	1.486965	-1.858566	5.342448
O	1.605081	-1.112472	6.304418
C	1.777775	1.001414	4.360716
O	-2.384633	0.963900	2.209182
O	-1.917056	-1.467812	2.498146
C	-1.151387	-0.048304	5.073745
Cl	1.345170	0.031366	-2.369587
Cl	-1.482617	-0.045299	-0.879332
C	1.581316	-3.362260	5.471028
H	3.377046	0.038305	-0.416632
H	3.321330	0.000550	2.063267
H	1.056065	-2.142523	3.377191
H	1.957778	-3.840008	4.562907
H	2.229105	-3.606569	6.314434
H	0.579777	-3.756322	5.678872
H	-1.177169	-1.130200	5.249670
H	-0.606880	0.440423	5.882601
H	-2.172620	0.341344	5.059602
H	1.485611	1.103014	5.404559
H	2.834058	0.719248	4.328289
H	1.640591	1.955922	3.846276

Single crystal X-ray diffraction data

1a (CCDC 1995299)

The crystal was grown from ethyl acetate by allowing a solution of the compound in a vial to evaporate slowly.

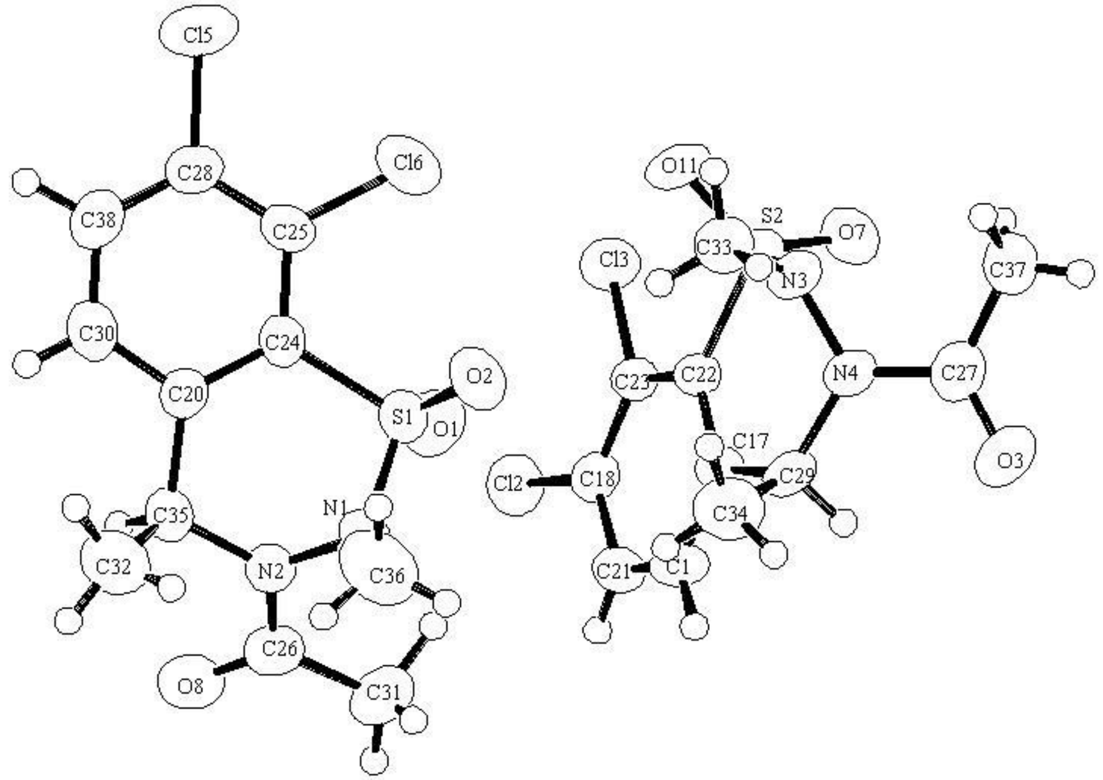

Figure S11. ORTEP plot of **1a** with 50% probability

Table S4. Crystal parameters of **1a**

Space group	P-1 (#2)
a [Å]	9.8457(7)
b [Å]	10.1492(7)
c [Å]	15.5653(10)
α [°]	100.491(4)
β[°]	100.007(4)
γ[°]	109.544(4)
V [Å^3]	1394.27(16)
density [g/cm^3]	1.540

3a · H_2O (CCDC 1995298)

The crystal was grown from methanol by allowing a solution of the compound in a vial to evaporate slowly.

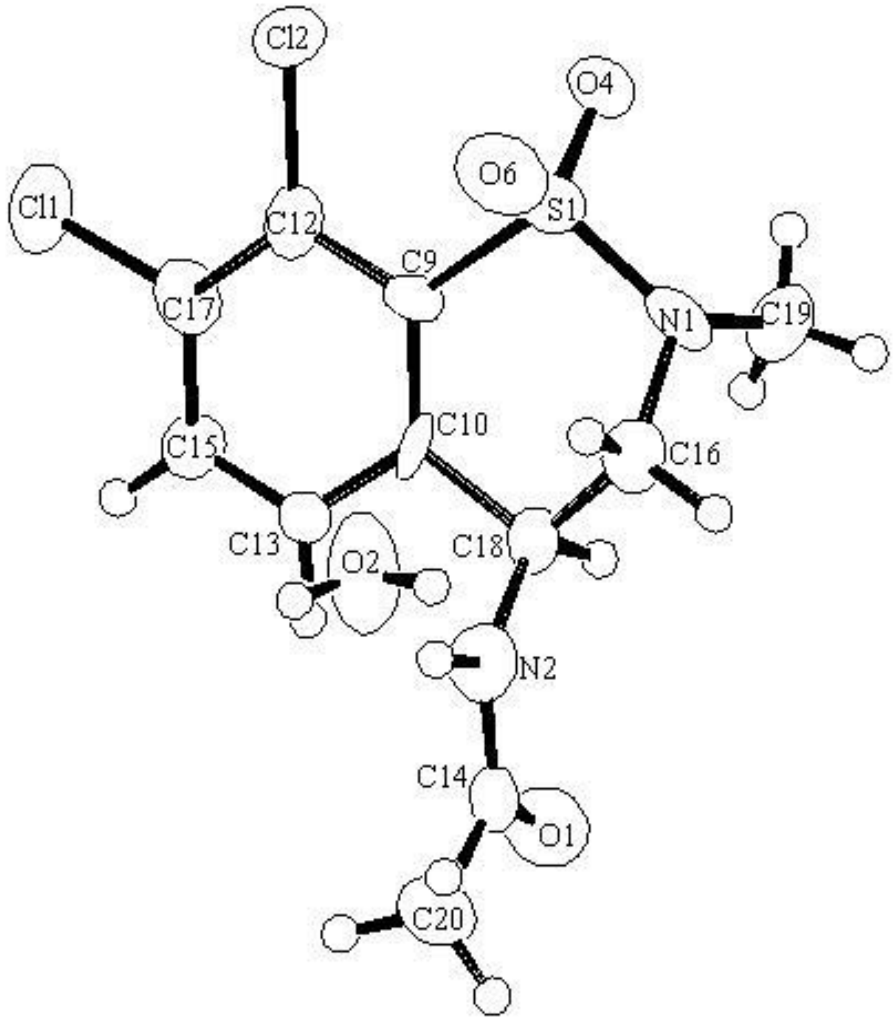

Figure S12. ORTEP plot of **3a · H_2O** with 50% probability

Table S5. Crystal parameters of **3a · H_2O**

Space group	$P4_2bc$ (#106)
a [Å]	20.0729(4)
c [Å]	7.2451(2)
V [Å^3]	2919.21(12)
density [g/cm^3]	1.553
